# Do children born to teenage parents have lower adult intelligence? A prospective birth cohort study

**DOI:** 10.1371/journal.pone.0167395

**Published:** 2017-03-09

**Authors:** Mohsina Khatun, Abdullah Al Mamun, James Scott, Gail M. William, Alexandra Clavarino, Jake M. Najman

**Affiliations:** 1 School of Public Health, Faculty of Medicine and Biomedical Sciences, The University of Queensland, Brisbane, Australia; 2 UQ Centre for Clinical Research, Faculty of Medicine and Biomedical Sciences, The University of Queensland, Brisbane, Australia; 3 Metro North Mental Health Service, Royal Brisbane and Women’s Hospital, Queensland, Australia; 4 School of Pharmacy, The University of Queensland, Brisbane, Australia; 5 School of Social Science, The University of Queensland, Brisbane, Australia; Hopital Robert Debre, FRANCE

## Abstract

Teenage motherhood has been associated with a wide variety of negative offspring outcomes including poorer cognitive development. In the context of limitations of previous research, this paper assesses the contemporary relevance of this finding. In this study we investigate the long-term cognitive status (IQ) among 21 year adult offspring born to teenage parents using the Mater University Study of Pregnancy- a prospective birth cohort study, which recruited all pregnant mothers attending a large obstetrical hospital in Brisbane, Australia, from 1981 to 1983. The analyses were restricted to a sub-sample of 2643 mother-offspring pair. Offspring IQ was measured using the Peabody Picture Vocabulary Test at 21 year. Parental age was reported at first clinic visit. Offspring born to teenage mothers (<20 years) have -3.0 (95% Confidence Interval (CI): -4.3, -1.8) points lower IQ compared to children born to mothers ≥20 years and were more likely to have a low IQ (Odds Ratio (OR) 1.7; 95% CI: 1.3, 2.3). Adjustment for a range of confounding and mediating factors including parental socioeconomic status, maternal IQ, maternal smoking and binge drinking in pregnancy, birthweight, breastfeeding and parenting style attenuates the association, though the effect remains statistically significant (-1.4 IQ points; 95% CI: -2.8,-0.1). Similarly the risk of offspring having low IQ remained marginally significantly higher in those born to teenage mothers (OR 1.3; 95% CI: 1.0, 1.9). In contrast, teenage fatherhood is not associated with adult offspring IQ, when adjusted for maternal age. Although the reduction in IQ is quantitatively small, it is indicative of neurodevelopmental disadvantage experienced by the young adult offspring of teenage mothers. Our results suggest that public policy initiatives should be targeted not only at delaying childbearing in the population but also at supporting early life condition of children born to teenage mothers to minimize the risk for disadvantageous outcomes of the next generation.

## Introduction

Globally, adolescent (10–19 years) birth rates have declined significantly over the last few decades though adolescent mothers still account for 11% of all births globally. It has been estimated that a disproportionate 23% of the overall burden of disease (disability-adjusted life years) due to pregnancy and child birth [1] is attributable to adolescent motherhood. In Australia, the teenage birth rate has declined considerably from over 50 per 1000 females in 1970s to 16 per 1000 births in 2012[2]. This rate is still relatively high compared to many other economically developed countries [1, 3].Teenage pregnancy and childbirth remains an important health and social issue in many countries including Australia due to its association with higher risk of maternal morbidity and mortality[4–7] and long-term psychological, social and economic consequences both for the young mother and her child[8–10].

While a good deal is known about the impact of teenage parenting on offspring childhood and adolescent cognitive development [11–14], relatively little research has especially addressed the long term cognitive outcomes of offspring born to teenage parents. Evidence of an association between young parental age and offspring IQ is mixed; some studies report lower IQ score in children with both parents being teenagers [3, 15–17], and others report either younger maternal [16, 18, 19] or paternal [15, 20, 21] age but not both are associated with lower offspring IQ compared to parents older than 20 years. Many studies reported in the literatures are retrospective, and do not adjust for maternal IQ, which is the strongest predictor of offspring IQ [22, 23]. Many of the available studies do not take into account a range of other potentially important confounding and mediating factors including parental socio-economic status, child birth weight, breastfeeding, and child rearing that may play a role in the relationship between teen parental age and lower offspring IQ.

Early life socioeconomic status, particularly educational status, income support and breastfeeding are important factors that may mediate the association between young parental age and cognitive development of offspring. For instance, by age five those offspring with degree-educated parents may be as much as 18 months ahead on vocabulary and 13 months ahead on problem solving ability[22]. Similarly, mothers who breastfeed their children are more likely to have an offspring with better educational achievement and higher IQ by young adulthood [24]. Although the direct effects of teenage pregnancy on reduced offspring IQ may be modest, the indirect effects may be important due to a multitude of psychosocial risk factors [25, 26]. Teenagers who experience pregnancy are often socially disadvantaged with low levels of educational attainment and income. Many have left school early; have no partner, have reduced rates of breastfeeding their child and engage in less mother-offspring-interaction [27, 28]. It may be the different home environment and social support and family interactions that account for any reduction in offspring IQ of teenage mothers rather than some aspects of biological age of the mother *per se*[26].

Intellectual differences in children born to teenage parents may become more pronounced as children develop. Small differences are seen in studies in the preschool years while larger differences have been found by the elementary school years [29]. By adolescence, school achievement among the offspring of teenage mothers is markedly lower. For example, in the Baltimore study and in the National Survey of Children, about half of the African American ethnic adolescents born to teenage mothers had failed a grade. In comparison, only 20% of those adolescents born to later child bearers in the National Survey of Children had repeated a grade[29].These educational challenges in adolescence increase the risk for early initiation of sexual activity and adolescent pregnancy[30], perpetuating the intergenerational cycle [31] of disadvantage in life course outcomes including poor health with subsequent increased mortality[32, 33], and poorer social, economic and environmental circumstances [34].

This study investigates the association between teenage parenthood and young adult offspring IQ at 21 years, adjusting for parental psychosocial, economic, and biological confounders. We also test whether parental educational status, duration of breastfeeding, and child rearing practices- known to influence offspring IQ development, mediate the association between teenage parenting and offspring cognitive development.

## Materials and methods

### Study sample and design

Data for this study were drawn from the Mater-University of Queensland Study of Pregnancy (MUSP) cohort. MUSP is a mother-offspring pair birth cohort study which commenced in 1981–83 at the Mater Misericordiae Mothers’ Hospital in Brisbane, Australia. Some 7223 mothers gave birth to a live singleton baby at the study hospital. Baseline data were collected at the mother’s first obstetric clinic visit (mean 18 weeks gestation) and follow up data were gathered prospectively on mothers and offspring at 3–5 days postpartum, six months, five, 14, and 21 years of age. Of the 7223 offspring who constituted the original sample, a sub-sample of 2643 had their intelligence assessed at the 21 year follow-up. This latter group constitutes the analytical sample for this study. Details of the study design, sampling and response rates are provided elsewhere [35, 36].

Informed consent from the mothers was obtained at all data collection phases of the study and from the offspring at 14 and 21 years. Ethics committees at the Mater Hospital and the University of Queensland approved each phase of the study.

### Measurements

#### Maternal and paternal factors

Maternal age at first clinic visit (FCV) was calculated based on the maternal date of birth and FCV survey date. Paternal age was reported by the mothers. Parental age was categorised as teenage parents (pregnancy delivered <20 years of age) vs. all other aged parents (pregnancy delivered 20+ years of age). Mothers and fathers were stratified and those older than 20 years were the reference group in all the comparisons. To assess level of education, mothers, at the time of recruitment, were asked the level at which they and their partner had completed their education. The more detailed original responses were stratified into ‘incomplete high school’, ‘high school completed’ and ‘post-high school’. Maternal smoking during pregnancy was classified as nil, 1–9 cigarettes/day and 10 or more cigarettes/day. Family income in the year of pregnancy was categorised as low (<$A10,400), middle ($A10,400-$A15,599) or high (≥$A15,600). At FCV the mother was asked whether her pregnancy was planned with response options ‘yes’, ‘no’ or ‘unsure’. As the number in the “unsure” group is small we combined this with ‘no’. At 14 years, mothers reported if there had been a change in partner since the birth of the child. Maternal depression was measured using the seven-item depression subscale from the delusions-symptoms-states inventory: state of anxiety and depression (DSSI/SAD)[37]. The measure was developed to detect signs and symptoms of psychopathology that limit a person’s capacity to function and to maintain relationships. This measure has high internal validity[37], correlates well, and shares items, with other measures of depression and anxiety such as the Edinburgh Postnatal Depression Scale and the Hospital Anxiety and Depression Scale [38].The Cronbach’s alpha for the scale was 0.79. Maternal depression was categorised into two groups- depressed and not depressed.

Birth weights were converted into z scores representing birth weight standardized to gender and gestational age, calculated as birth weight minus birth weight mean divided by the SD adjusted for gender and gestational age. A gender and gestational age (in weeks) standardised birth weight z-score was computed to give a measure of intrauterine growth [39]. Breastfeeding was self-reported by the mothers at the 6-month follow-up. Duration of breastfeeding was categorised into never, <4 or ≥4 months. At the 5 year follow-up mothers responded to items assessing pattern of mother-child interaction and maternal response to misbehaviour, as well as reporting the child’s attendance at pre-school. Mother-child interaction was assessed using four items (“try to encourage baby to be interested in what going on”; “my baby likes me talking to him/her”; “spend a lot of time teaching baby to recognise things”; “love to play with my baby”) each with five response options (strongly agree, agree, neutral, disagree, strongly disagree) allocated scores from 1 to 5 and Cronbach’s alpha was 0.654. Summed average means of the items were calculated and multiplied by 10. The total score varied from 10 to 50. On the basis of this score, mother-child interaction was categorised into two groups–always (10.0 to 39.9) and not always (40.0 to 50.0).

Maternal response to misbehaviour was assessed by asking the mother how she would respond to five situations involving her child-(“child refuses to clean up room”; “child takes something belonging to another child and punches”; “child makes fun of a crippled person”; “child touches hot stove”; and “child breaks something indoors after told to play outdoors”) to which the mother could respond in one of three possible ways: always, sometimes and never. Summed average means of the items were multiplied by 10. Total score was regrouped into two categories: always (10.0 to 15.0) and not always (15.1 to 30.0). The Cronbach alpha for this scale is 0.819.

#### Measurement of intelligence

For both mothers and offspring, intelligence was measured at the 21 year follow-up using the Peabody Picture Vocabulary Test (PPVT–R)[40]; the PPVT-R is a norm-referenced measure of verbal comprehension and/or receptive vocabulary for individuals aged 2 years 6 months through to 90 years. The test comprises a set of stimulus words. The respondent must identify which of four pictures shown on a series of cards depicts the word spoken by the interviewer. Interviews were conducted either at a central facility or in the respondent’s home under controlled conditions.

The Peabody Picture Vocabulary test has been found to be a reliable measure of verbal comprehension [41, 42]. In one study the reliability coefficients for various age groups ranged from 0.59–0.90 (median = 0.77) indicating good stability[40]. The PPVT-R has been found to yield consistent scores for children with mental retardation over a 7 month interval [41]. The Peabody test also correlates well with other measures of intelligence in both early childhood and later life[41] and is highly correlated with school success. In the supplementary analysis, low IQ has been defined for PPVT score below one standard from the mean and otherwise normal IQ.

### Statistical analysis

Parental demographic and socio-economic characteristics and off-spring IQ at 21yrs are separately presented by parental age (<20y vs 20+y) at FCV. The chi-square or Fishers exact test and t-test are used for categorical and continuous variables as appropriate. Simple linear regressions are applied to test the trend of systematic increase or decrease of off-spring mean IQ score over the level of parental age groups. The non-zero slope (rate of increment or decrement of mean IQ) of the regression with p-value <0.05 for t-test is used for linear trend in off-spring IQ across parental age groups. A trend test was also applied for the mutually adjusted parental ages using multiple linear regressions. The normality of the outcome variable is tested applying histogram and discerning whether off-spring IQ approximates the bell curve of a normal distribution.

Three regression models are constructed to examine the independent association of parental age and offspring IQ at 21 years. Residual diagnostics are used to examine the regression assumptions and to identify influential and outlier cases. The variance inflation factors for the predictors are screened to avoid multicollinearity. The first model is mutually adjusted for maternal and paternal age. The second model is adjusted for the potential confounders including maternal IQ, income, birth weight, child sex, living with same partner at 14 years as at the birth of child, planned pregnancy, depression, smoking and binge drinking. The final model was additionally adjusted for contextual mediators including parental education, breastfeeding and child rearing factor. All the regression analyses are performed using the multiple imputed data for the missing values on covariates [43] assuming data are missing at random. Similarly, we have conducted additional analyses using logistic regression models where the outcome was two categories- low IQ when standardised IQ was below 1SD of mean, otherwise normal IQ. Results were presented in adjusted odds ratio (OR) with 95% confidence interval.

To evaluate the extent to which maternal education, paternal education, breastfeeding and child rearing factor (e.g. physical punishment/smacking) mediates the relationship between maternal age and offspring IQ at 21years, the total effect was decomposed into direct and indirect effect using STATA user-written program *KHB* command. The Karlson-Holm-Breen (KHB) method[44] disaggregates the total effect into individual indirect effect for four mediators and also for combined effect. The KHB method can be used with various types of independent variables, mediators, and dependent variables, including continuous ones; it does not rely on the distributional assumptions of the control variables and interpretation of the results are simple[44]. However, KHB method could not be used for multiple imputed data. Analyses are performed using STATA version 13.0 (StataCorp, College Station,TX) and a P-value less than 0.05 is set for statistical significance.

## Results

Participant’s characteristics are presented in Table 1. Mothers were on average 2.8 years younger than fathers (mean age 25.4±SD 5.1 years vs. 28.2±6.1 years). Compared to 20+ years old mothers, a greater proportion of teenage mothers: were not living with same partner at 14 years as they had at the birth of child, had experienced unplanned pregnancy, did not complete high school education, had low family income, smoked cigarettes, did not breastfeed or breastfed for a shorter duration, had experienced depressed mood, and gave birth with relatively lower birth weight (all p-values<0.05), more likely to use physical punishment and less likely to explain the reasons for child’s bad behaviour when responding to a child’s misbehaviour. However, the maternal IQ was not significantly different between teenage and mothers older than 20 years (p-value 0.17). These differences were very similar when comparing teenage fathers with fathers older than 20 years.

**Table 1:** Parental and offspring characteristics by parental age categories <20 years vs. ≥20 years

selected characteristics		Maternal age			Paternal age^€^		
	Overall^Ѱ^ (n = 2643)	<20 years (n = 363)	≥20 years (n = 2280)	p-value	<20 years (n = 73)	≥20 years (n = 2518)	p-value
**Living with same partner as birth of child**							
No	769 (31.0)	52.7	27.6	<0.001	66.7	28.1	<0.001
Yes	1712 (69.0)	47.3	72.4		33.3	71.9	
**Planned Pregnancy**							
Yes	1218 (47.8)	22.1	51.9	<0.001	16.7	50.1	<0.001
No or unsure	1333 (52.3)	77.9	48.1		83.3	49.9	
**Child gender**							
Male	1300 (49.2)	48.5	49.3	0.773	65.8	48.8	0.004
Female	1343 (50.8)	51.5	50.7		34.3	51.2	
**Maternal education**							
Incomplete high	420 (16.0)	20.2	15.3	<0.001	15.1	15.9	0.058
Complete high	1682 (64.1)	69.3	63.3		75.3	63.7	
Post high	523 (19.9)	10.5	21.4		9.6	20.5	
**Paternal education**							
Incomplete high	439 (17.4)	17.1	17.4	<0.001	16.4	17.4	0.001
Complete high	1517 (60.0)	70.9	58.4		78.1	59.3	
Post high	574(22.7)	12.0	24.2		5.5	23.3	
**Family Income**							
<$10400	754(30.0)	54.4	26.3	<0.001	61.8	27.8	<0.001
$10400-$15599	995 (39.6)	30.1	41.0		22.1	40.7	
>$15599	763(30.4)	15.5	32.6		16.2	31.5	
**Smoking during pregnancy**							
Never smoked	1693(64.6)	50.8	66.9	<0.001	50.7	65.6	<0.001
1–9 cigarettes/day	417(15.9)	27.1	14.1		34.3	15.5	
10+ cigarettes/day	509 (19.4)	22.1	19.0		15.1	19.0	
**Binge drinking**							
Never binge	2082 (79.5)	80.0	79.5	0.810	78.1	79.9	0.705
Binge	536 (20.5)	20.0	20.6		21.9	20.1	
**Maternal depression**							
Not-Depressed	2107 (80.8)	64.1	83.5	<0.001	68.5	81.9	0.004
Depressed	500 (19.2)	35.9	16.5		31.5	18.1	
**Breastfeeding**							
Never	468 (18.4)	24.9	17.4	<0.001	20.3	17.7	0.002
< 4 months	954 (37.5)	50.0	35.6		55.1	37.0	
≥ 4 months	1124 (44.2)	25.2	47.0		24.6	45.3	
**Child attended at preschool**							
Yes	1498 (64.5)	62.5	64.8	0.439	62.5	64.7	0.735
No	823 (35.5)	37.5	35.2		37.5	35.3	
**Mother-child interaction**							
Always	2198 (86.0)	88.5	85.7	0.166	91.4	85.7	0.175
Not always	357 (14.0)	11.5	14.3		8.5	14.3	
**Physical punishment**							
Always	158 (7.5)	6.6	7.6	0.001	5.6	7.5	0.390
Sometimes	1556 (73.6)	82.2	72.3		81.5	73.2	
Never	401 (19.0)	11.3	20.1		13.0	19.4	
**Explaining for child bad behaviour**							
Always	1205 (53.7)	46.5	54.8	0.009	42.9	53.9	0.102
Not always	1038 (46.3)	53.5	45.2		57.1	46.1	
**Maternal IQ**	2141	256	1885		50	1992	
Mean±SD	96.9±10.4	96.2±8.4	97.0±10.6	0.170	96.7±9.6	97.0±10.4	0.824
**Off-spring IQ at 21y**	2643	363	2280		73	2445	
Mean±SD	103.3±10.3	100.5±10.0	103.8±10.3	<0.001	100.3±9.8	103.5±10.3	0.009
**Birth weight (kg)**							
Mean±SD	3.4±0.5	3.3±0.5	3.4±0.5	0.001	3.5±0.5	3.4±0.5	0.290

Ѱ Prevalence of variables might not sum to 2643 because of missing data.

€ There are 125 missing cases in paternal age.

While 13.7% of mothers were teenagers, only 2.9 percent of fathers were less than 20 years of age at the time of the child’s birth. Unadjusted mean IQ shows a clear trend towards increasing offspring IQ score as parental age increases (p-values<0.05, Table 2). However, after adjusting for maternal or paternal age, the association of parental age and offspring IQ remains statistically significant only for maternal age (p-value<0.001).

**Table 2:** Offspring mean (95% CI) IQ at age 21 year by maternal and paternal age at first clinic visit (cohort N = 2643).

			Offspring IQ at 21years	
Parental age	(%)	Mother’s IQ	Unadjusted Mean(95% CI)	Adjusted Mean(95% CI)^†^
** Maternal age at FCV (year) (n = 2643)**				
<20	13.7	96.2	100.5 (99.5, 101.6)	101.1 (99.8, 102.4)
20–24	38.3	96.1	102.8 (102.2, 103.5)	102.8 (101.9, 103.7)
25–29	29.6	97.8	104.0 (103.3, 104.7)	103.5 (102.5, 104.5)
30–34	13.6	98.0	105.7 (104.6, 106.8)	105.3 (104.0, 106.6)
35+	4.8	96.4	103.9 (102.1, 105.7)	103.4 (101.3, 105.5)
**P-trend**			**<0.001**	**0.001**
**Paternal age at FCV**^**€**^ **(year) (n = 2518)**				
<20	2.9	96.7	100.3 (98.0, 102.7)	102.2 (99.6, 104.8)
20–24	26.9	95.8	101.8 (101.0, 102.5)	102.6 (101.6, 103.7)
25–29	35.1	97.6	103.9 (103.2, 104.6)	103.9 (103.0, 104.8)
30–34	22.0	97.0	104.4 (103.6, 105.3)	103.8 (102.8, 104.8)
35+	13.2	97.7	104.5 (103.4, 105.6)	103.7 (102.4, 104.9)
**P-trend**			**<0.001**	**0.185**
**Overall mean IQ**		**96.9**	**103.3**	

€ There are 125 missing cases in paternal age.

† Parental age adjusted for each other.

Table 3 shows the mean difference in IQ at age 21 between offspring born to teenage parents compared with offspring born to parents older than 20 years (as the reference group), with adjustment for potential confounders and mediators in a series of multiple regression models. Results are presented for the 2643 young adults with multiple imputed data for the missing values in all the multivariable models. In the mutually adjusted parental age model (model 1), we have found that offspring of teenage mothers have an IQ lower by -3.0 points (95 percent confidence interval: -4.3, -1.8), on average, than offspring of mothers older than 20 years. Adjustments for potential confounders reduce the mean IQ difference to -2.2points (95% confidence interval: -3.6, -0.8). After adjusting for both confounders and mediators in the final model (model 3) the association has attenuated to -1.4 points (95%CI: -2.8, -0.01), but remains significant. The adjustment for mediators suggests that of the mean 2.2 IQ points that distinguish teenage mother an additional 0.8 IQ points reflects patterns of child rearing, breastfeeding and parental education. This leaves 1.4 IQ points that represents the disadvantage experienced by the children of the teenage mothers. Paternal age is not associated with offspring IQ at 21year in any of these models. Similarly, when we repeated this analysis for categorical outcome (low IQ vs. normal IQ), we found the odds ratio of being low IQ was 1.7 times (95% CI: 1.3, 2.3) of those offspring born to teenage mothers compared to their counterpart. This reduces to 1.3 (95% CI: 1.0, 1.9), when adjusted for confounders and mediators (S2 Table).

**Table 3:** Adjusted mean difference in Offspring IQ at 21year with 95% confidence interval (CI) by the parental age groups <20 years vs. 20+ years at first clinic visit using multiple imputed data (N = 2643).

	Maternal age <20 years vs. 20+ years		Paternal age <20 years vs. 20+ years	
	(20+ years as reference)		(20+ years as reference)	
Models	Mean difference (95% CI)	p-value	Mean difference (95% CI)	p-value
**Model 1**:				
Adjusted for parental age	-3.0 (-4.3,-1.8)	<0.001	-0.9 (-3.4,1.6)	0.491
**Model 2:**				
Model 1+ confounders^α^	-2.1 (-3.3,-0.9)	0.001	-0.9 (-3.3, 1.5)	0.469
**Model 3:**				
Model 1+confounders+ mediators^β^	-1.4 (-2.8, -0.1)	0.041	-0.7 (-3.5,2.1)	0.618

α Confounders: living with same partner as birth to child, planned pregnancy, gender of child, income, smoking status, binge drinking, mother’s depression, birth weight and maternal IQ.

β Mediators: breastfeeding, parental education, child rearing practices includes physical punishment, explain reasoning during parenting for child’s bad behaviour, child attend at preschool, and spending time teaching baby.

To further illustrate the association between maternal age and adult offspring IQ, we have stratified maternal IQ into four categories (< = 85, 86–95, 96–105, and 106+) (Table 4). For every category of maternal IQ, lower IQ is observed for offspring of adolescent mother compared to offspring of mothers older than 20 years although for some differences there is only a trend towards statistical significance. However, the overall IQ score for young adult children born to teenage mothers is on average 3.3 points lower than the children born to mothers older than 20 years (100.5 vs. 103.3; p-value <0.001) (Table 4).

**Table 4:** Mean (95% CI) offspring IQ at 21 year by mother's IQ and the age of pregnancy.

Mother’s IQ^£^	Mother's mean IQ	Teenage pregnancy		Other pregnancy		
		n	Offspring IQ at 21y Mean (95% CI)	n	Offspring IQ at 21y Mean (95% CI)	P-value
< = 85	80.4	23	93.3 (89.9, 96.7)	210	97.4 (95.9,98.9)	0.087
86–95	90.8	94	99.5 (97.4, 101.6)	658	101.3 (100.6, 102.0)	0.092
96–105	100.0	114	101.7 (100.0, 103.4)	638	105.0 (104.2, 105.7)	<0.001
106+	112.3	25	105.4 (101.5, 109.2)	379	109.6 (108.7, 110.5)	0.025
Total	96.9^Ω^	256	100.5^‡^(99.5,101.6)	1885	103.8^‡^(103.3,104.2)	<0.001

£ Mother’s IQ was missing for 502 cases in the sample.

Ω Overall mean of mother’s IQ.

‡ Mean IQ of offspring at their 21 year.

**Fig 1:**
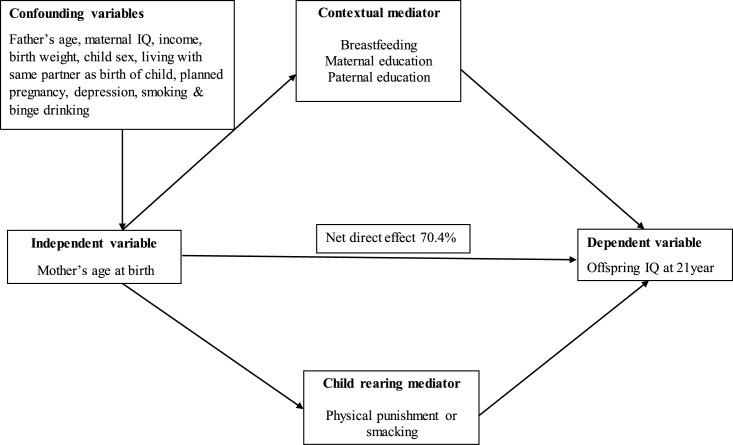
Direct and indirect effect of mother’s age on offspring IQ at 21 year, mediated by breastfeeding and parental education and child rearing.

In the additional analysis, we have explored what proportion of the total association between teenage parenthood and adult offspring IQ was due to direct and indirect effect through the pathways of parental education, breastfeeding and child rearing (Fig 1 and Table 5).

**Table 5:** Proportion of total effect of maternal age (<20years vs. 20+years, reference category 20+years) at pregnancy on offspring IQ at 21 year mediated by breastfeeding, parental education and child rearing.

Mediators^π^	Proportion (%) of total effect mediated
Breastfeeding	14.1%
Maternal education	5.9%
Fathers education	7.7%
Child rearing-Physical punishment/smacking^Ω^	2.0%
Combined effect of breast feeding, parental education and child rearing	29.6%

π Mediation effects have been adjusted for the confounders: father’s age, maternal IQ, income, birth weight, child sex, living with same partner as birth of child, planned pregnancy, depression, smoking, and binge drinking.

^Ω^ Child rearing includes physical punishment or child smacking only.

The results in Table 5 show that 7.7% of the total effect of teenage pregnancy on young adult offspring IQ was due to confounding of paternal education whereas maternal education contributes 5.9%. The degree of mediation was much larger for breastfeeding (14.1%) than for parental education and child rearing style (2.0%).The overall explanatory contribution for all four mediators was 29.6%.

## Discussion

This study shows that offspring of teenage mothers and fathers have small but significant mean reductions in cognitive ability as young adults. However, this relationship only remained statistically significant for teenage mothers after controlling for a range of potential confounders and mediators prospectively collected around pregnancy and in early childhood period. We also found a significant linear trend of increasing cognitive outcome with maternal age after adjusting for paternal age. Offspring of teenage mothers were at increased risk of low IQ at 21 years after adjusting for confounding and mediating factors.

Direct comparisons with other studies of offspring cognitive development born to teenage parents are difficult because researchers have used a wide range of cognitive measures and have different study designs, age groups and adjusted for different confounding factors. However, the finding that young maternal age is associated with a lower mean IQ of young adult offspring is consistent with three large studies [3, 15, 45]. McGrath et al.[45] using a cross-sectional study (n = 169,009) found that the offspring at age 18 years who born to teenage mothers had a lower mean IQ of -1.69 (95% CI: -2.03 to -1.34) after adjusting for parental age and education, birth order, multiple birth status, birth weight and gestational age. McGrath and colleagues reported offspring of teenage fathers also had a lower IQ (Mean difference -0.69; 95% CI: -1.34, -0.04). The other two studies reported similar associations both for teenage mothers and fathers although the main focus of these studies were to examine the association between advanced parental age and offspring cognitive development [3, 15]. Compared to McGrath et al., the estimate of our effect size in the fully adjusted model is slightly lower (IQ mean difference -1.4; 95% CI: -2.8,-0.1) for teenage mother and similar for teenage father (IQ mean difference -0.7;-3.5, 2.1). When we considered low (1SD below mean IQ) vs. normal IQ of offspring at 21 year, we found similar direction and strength (unadjusted OR 1.7; adjusted OR 1.3) (S2 Table) of association with teenage mothers but not with fathers. In our study, we have additionally adjusted for important confounding factors including maternal IQ, life style in pregnancy, breast feeding and child rearing practices. It is likely that our small sample size (n = 73) of teenage fathers underlies the non-significant association between young paternal age and offspring IQ.

There are a variety of confounding or mediating factors hypothesised to influence the association between teen parental age and offspring intelligence [46]. In this study, we have determined the direct and indirect pathways of teenage parenthood through their educational attainment, breastfeeding and parenting style. Three mediators in combination only explain approximately 30% of the age effect on offspring IQ. After adjusting for a range of potential confounders around pregnancy and early childhood, we found the association of teenage maternal age with offspring cognitive development remains statistically significant. Although there may be unknown biological factors for this association, it is important to consider that psychosocial mechanisms whose complexities are not able to be measured in entirety (e.g. parenting behaviours, mother-child interactions, maternal well-being, and parental conflict) underlie this association [47]. Although we have considered several confounding factors including maternal IQ, maternal life style in pregnancy, mental health, living with partner, birth weight, gestational age and child rearing factors, other residual confounders may play a role for this association.

Results of this and other studies reporting the significant association between teenage mothers and increased risk of offspring cognitive impairment at 21 years and lower mean cognitive development reinforces the importance of interventions which supports young and socially disadvantaged parents during pregnancy and the early years. The Family Nurse Partnership is one such intervention that has demonstrated a positive impact on early learning development, readiness to school and academic performance of offspring of young mothers [48, 49]. In addition to the effects on the offspring, it is important to consider teenage mothers who are likely to have missed educational and vocational opportunities placing them on a disadvantaged trajectory [50]. Together, it is clear that pregnancy of young mothers has substantial adverse psychosocial consequences to both mother and offspring where supporting programmes shown to be effective in preventing adolescent pregnancy [51].

The findings of this study need to be interpreted in the context of some caveats. Firstly, similar to other longitudinal studies, this study has loss to follow up. Initially, the survey recruited 7223 mothers/children pairs however; only 2643 cases are available for this analysis when offspring IQ is measured after 21 years of postpartum (S1 Table). Mothers of offspring lost to follow up were disproportionately of low levels of educational attainment and income, experiencing a number of psychosocial problem including depression as well as marital distress [36, 52]. Generally, more residentially mobile participants were prone to being lost to follow-up and simply cannot be located. We have previously used multiple strategies to assess the impact of attrition on our estimates of association [35] and found attrition does not substantially bias findings. In this study, we have used multiple imputations and found that our estimates of association are only minimally affected by loss to follow-up. Secondly, mother’s IQ is not measured at birth but at 21years of postpartum. We have assumed this has remained constant over the previous 21 years as there is relative stability in IQ after ten years of age [5, 7] peaking between 25 and 29 years before declining more steeply after age 70 [6]. The measures for parent-child interactions used in this study would not capture the intricacies of these complex relationships and at best serve as proxy measures. Finally, although home environment, supportive social welfare and family structure are identified as positive predictors for child rearing and child’s cognitive development particularly among the teenage parents [8, 9], they were not considered in the analysis.

## Conclusion

This study shows teen maternal child birth is associated with lower offspring IQ and increased risk of offspring cognitive impairment which persists into young adulthood. The small but significant decrease in offspring IQ combined with other challenges often faced by children of teenage mothers may contribute to increased risk of poor educational performance and intergenerational transfer of psychosocial and health disadvantage. With this consequence in mind, our results suggest public policy initiatives should be targeted not only at delaying childbearing in the population but also at supporting early life condition of children born to teenage mothers to minimize the risk for disadvantageous outcomes of the next generation.

## Supporting information

S1 Table Comparison of the respondents’ characteristics who have been included versus those who have been excluded at 21 years post-partum. (DOCX)

S2 Table Adjusted odd ratio (95% confidence interval) in offspring IQ at 21years by the parental age groups<20 years vs. 20+ years at first clinic visit using multiple imputed data (N = 2643). (DOCX)

S3 Table Mean (95% CI) and p-value of offspring IQ at 21 years for the various medical characteristics of pregnancy. (DOCX)

S4 Table Input data. (DOCX)

S1 Data (PDF)
