# Supplementary material for: Do children born to teenage parents have lower adult intelligence? A prospective birth cohort study
Source: PLoS One. 2017 Mar 9;12(3):e0167395. doi: 10.1371/journal.pone.0167395 (PMC5344312; doi:10.1371/journal.pone.0167395)
Supplement: S1 Table — (DOCX) [file pone.0167395.s001.docx]

**Supplementary material**

**S1 Table.** Comparison of the respondents’ characteristics who have been included versus those who have been excluded at 21 years post-partum

| **Selected characteristics** | Women who have been included in the analysis  (n=2643) | Women who have  been excluded from the analysis (n=4580) | p-value |
| --- | --- | --- | --- |
| **Maternal age** |  |  |  |
| **<20 years** | 363 (13.7) | 821 (17.9) | <0.001 |
| **20+ Years** | 2280 (86.3) | 3759 (82.1) |  |
| **Paternal age** |  |  |  |
| **<20 years** | 73 (2.9) | 233 (5.5) | <0.001 |
| **20+ Years** | 2445 (97.1) | 4014 (94.5) |  |
| **Planned Pregnancy** |  |  |  |
| Yes | 1218 (47.8) | 1836 (41.9) | <0.001 |
| No or unsure | 1333 (52.3) | 2550 (58.1) |  |
| **Child gender** |  |  |  |
| Male | 1300 (49.2) | 2458 (53.7) | <0.001 |
| Female | 1343 (50.8) | 2122 (46.3) |  |
| **Maternal education** |  |  |  |
| Incomplete high | 420 (16) | 885 (19.5) | <0.001 |
| Complete high | 1682 (64.1) | 2927 (64.4) |  |
| Post high | 523 (19.9) | 733 (16.1) |  |
| **Paternal education** |  |  |  |
| Incomplete high | 439 (17.4) | 787 (18.5) | <0.001 |
| Complete high | 1517 (60) | 2726 (64) |  |
| Post high | 574 (22.7) | 748 (17.6) |  |
| **Family Income** |  |  |  |
| <$10400 | 754 (30) | 1554 (36.7) | <0.001 |
| $10400-$15599 | 995 (39.6) | 1572 (37.1) |  |
| >$15599 | 763 (30.4) | 1111 (26.2) |  |
| **Smoking status** |  |  |  |
| Never smoked | 1693 (64.6) | 2702 (59.6) | <0.001 |
| 1-9 cigarettes/day | 417 (15.9) | 774 (17.1) |  |
| 10+ cigarettes/day | 509 (19.4) | 1057 (23.3) |  |
| **Binge drinking** |  |  |  |
| Never binge | 2082 (79.5) | 3539 (78.3) | 0.234 |
| Binge | 536 (20.5) | 979 (21.7) |  |
| **Maternal depression** |  |  |  |
| Not-Depressed | 2107 (80.8) | 3354 (74.9) | <0.001 |
| Depressed | 500 (19.2) | 1124 (25.1) |  |
| **Breastfeeding** |  |  |  |
| Never | 468 (18.4) | 972 (23.6) | <0.001 |
| < 4 months | 954 (37.5) | 1645 (39.9) |  |
| ≥ 4 months | 1124 (44.2) | 1502 (36.5) |  |
| **Birthweight**, mean±SD | 3.4±0.5 | 3.4±0.5 | 0.363 |

| **Gestation** |  |  |  |
| --- | --- | --- | --- |
| <37 weeks (Preterm birth) | 110 (4.2) | 186 (4.1) | 0.835 |
| 37+ weeks (Not preterm) | 2533 (95.8) | 4394 (95.9) |  |
| **Foetal distress** |  |  |  |
| No | 1921 (72.7) | 3404 (74.3) | 0.127 |
| Yes | 722 (27.3) | 1176 (25.7) |  |
| **Duration of 1^st^ stage (to full cervical dilation)** |  |  |  |
| <3 | 540 (20.4) | 911 (19.9) | 0.641 |
| 3-5 | 865 (32.7) | 1501 (32.8) |  |
| 6-8 | 617 (23.3) | 1035 (22.6) |  |
| >8 | 621 (23.5) | 1132 (24.7) |  |
| **Duration of 2^nd^ stage (to full cervical dilation)** |  |  |  |
| <10 | 765 (29.1) | 1341 (29.4) | 0.371 |
| 10-14 | 461 (17.5) | 727 (15.9) |  |
| 15-30 | 721 (27.4) | 1289 (28.3) |  |
| >30 | 683 (26.0) | 1203 (26.4) |  |
