# Supplementary material for: Do children born to teenage parents have lower adult intelligence? A prospective birth cohort study
Source: PLoS One. 2017 Mar 9;12(3):e0167395. doi: 10.1371/journal.pone.0167395 (PMC5344312; doi:10.1371/journal.pone.0167395)
Supplement: S2 Table — (DOCX) [file pone.0167395.s002.docx]

**Supplementary material**

**S2 Table.** Adjusted odd ratio (95% confidence interval) in offspring IQ at 21years by the parental age groups<20 years vs. 20+ years at first clinic visit using multiple imputed data (N=2643)

| **Models** | **Maternal age <20 years vs. 20+ years**  **(20+ years as reference)** | | | **Paternal age <20 years vs. 20+ years**  **(20+ years as reference)** | | |
| --- | --- | --- | --- | --- | --- | --- |
|  | Odd ratio (95%CI) | p-value | Odd ratio (95%CI) | | p-value |  |
| **Model 1**: |  |  |  | |  |  |
| Adjusted for parental age | 1.7  (1.3, 2.3) | <0.001 | 1.0  (0.6, 1.9) | | 0.919 |  |
| **Model 2:** |  |  |  | |  |  |
| Model 1+ confounders^α^ | 1.5  (1.1,2.1) | 0.009 | 0.9  (0.5,1.8) | | 0.874 |  |
| **Model 3:** |  |  |  | |  |  |
| Model 1+confounders+ mediators^β^ | 1.3  (1.0, 1.9) | 0.086 | 0.9  (0.5,1.7) | | 0.727 |  |

α- confounders: living with same partner as birth to child, planned pregnancy, gender of child, income, smoking status, binge drinking, mother’s depression, birth weight and maternal IQ

β- mediators: breastfeeding, parental education, child rearing practices includes physical punishment, explain reasoning during parenting for child’s bad behaviour, child attend at preschool, and spending time teaching baby
