# Supplementary material for: Do children born to teenage parents have lower adult intelligence? A prospective birth cohort study
Source: PLoS One. 2017 Mar 9;12(3):e0167395. doi: 10.1371/journal.pone.0167395 (PMC5344312; doi:10.1371/journal.pone.0167395)
Supplement: S3 Table — (DOCX) [file pone.0167395.s003.docx]

**Supplementary material**

**S3 Table**. Mean (95% CI) and p-value of offspring IQ at 21 years for the various medical characteristics of pregnancy

|  |  |  |  | Mother’s age at pregnancy | | |
| --- | --- | --- | --- | --- | --- | --- |
| Medical characteristics | n (2643) | Off spring IQ at 21 years Mean (95% CI) | p-value | <20 years (%) | 20+ years (%) | p-value |
| Admitted to ICU |  |  |  |  |  |  |
| No | 2460 | 103.4 (103.0, 103.8) | 0.203 | 92.6 | 93.2 | 0.909 |
| Yes | 183 | 102.4 (100.8, 104.0) |  | 7.4 | 6.8 |  |
| Foetal distress |  |  |  |  |  |  |
| No | 1921 | 103.3 (102.8, 103.7) | 0.772 | 64.5 | 74.0 | <0.001 |
| Yes | 722 | 103.3 (102.8, 103.7) |  | 35.5 | 26.0 |  |
| Duration of 1^st^ stage (to full cervical dilation) | |  |  |  |  |  |
| <3 | 540 | 102.8 (101.9, 103.7) | 0.412 | 11.9 | 21.8 | <0.001 |
| 3-5 | 865 | 103.3 (102.7, 104) |  | 27.0 | 33.6 |  |
| 6-8 | 617 | 103.3 (102.5, 104.1) |  | 25.9 | 22.9 |  |
| >8 | 621 | 103.8 (103, 104.6) |  | 35.3 | 21.6 |  |
| Duration of 2^nd^ stage (to full cervical dilation) | |  |  |  |  |  |
| <10 | 765 | 103.4 (102.6, 104.1) | 0.088 | 16.3 | 31.1 | <0.001 |
| 10-14 | 461 | 102.3 (101.4, 103.2) |  | 12.2 | 18.4 |  |
| 15-30 | 721 | 103.5 (102.7, 104.3) |  | 32.1 | 26.7 |  |
| >30 | 683 | 103.8 (103, 104.5) |  | 39.3 | 23.8 |  |
| Mode of delivery |  |  |  |  |  |  |
| Spontaneous vaginal | 2056 | 103.1 (102.7, 103.6) | 0.097 | 80.7 | 77.3 | 0.149 |
| Other | 587 | 103.9 (103.1, 104.8) |  | 19.3 | 22.7 |  |
| Gestational age (week) |  |  |  |  |  |  |
| <37 | 110 | 101.8 (99.6, 104.1) | 0.126 | 4.7 | 4.1 | 0.592 |
| 37+ | 2533 | 103.4 (103.0, 103.8) |  | 95.3 | 95.9 |  |
| Apgar score at 1 min | |  |  |  |  |  |
| >8 | 1358 | 103.2 (102.7, 103.8) | 0.947 | 49.3 | 54.8 | 0.057 |
| ≤8 | 1157 | 103.2 (102.6, 103.8) |  | 50.7 | 45.2 |  |
| Apgar score at 5 min | |  |  |  |  |  |
| >8 | 2356 | 103.2 (102.8, 103.6) | 0.807 | 93.4 | 95.0 | 0.216 |
| ≤8 | 131 | 103.4 (101.3, 105.6) |  | 6.7 | 5.0 |  |

Summation of the prevalence is not equal to 2643 when there are missing cases
