# Supplementary material for: Do children born to teenage parents have lower adult intelligence? A prospective birth cohort study
Source: PLoS One. 2017 Mar 9;12(3):e0167395. doi: 10.1371/journal.pone.0167395 (PMC5344312; doi:10.1371/journal.pone.0167395)
Supplement: S4 Table — (DOCX) [file pone.0167395.s004.docx]

**Supplementary material**

**S4 Table. Input data**

**Data Dictionary**

| Variable name | Label | Value label |
| --- | --- | --- |
| mage_FCV_5CAT | Maternal age at first clinic visit | 13-19=0  20-24=1  25-29=3  30-34=4  35+=5 |
| fage_FCV_5CAT | Paternal age (reported by mother at her first clinic visit) | 13-19=0  20-24=1  25-29=3  30-34=4  35+=5 |
| offiq21 | peabody-standard score |  |
| mumiq21 | peabody-standard score |  |
| samepart | same partner as birth of child (reported at 14y FU) | 1 No  2 Yes |
| planned | Planned to get pregnant | 1 No  2 Yes |
| sex | Child gender | 1 Male  2 Female |
| mumedu | Maternal education at first clinic visit | 1 Incomplete high  2 Complete high  3 Post-high |
| dadedu | Paternal education | 1 Incomplete high  2 Complete high  3 Post-high |
| familyincome | Family income at first clinic visit | 1 (<AU$10400)  2 (AU$10400-15599)  3 >(AU$15599) |
| mumsmoking | Smoking during pregnancy | 0 Never smoked  1 1 to 9 cigarettes/day  2 10+ cigarettes/day |
| bingepreg | Binge drinking in pregnancy | 1 No  2 yes |
| mumdep | Depression at first clinic visit | 1 No  2 Yes |
| breastfeeding6 | Breastfeeding (reported at 6-m FU) | 1 Never  2 <4 months  3 4+ months |
| childattend | Child attended at preschool | 0 Yes  1 No |
| Mother_child | **Mother-child interaction** | 1 No  2 Yes |
| csmack | Physical punishment | 1 Always  2 Sometime  3 Never |
| childbehab | **Explaining for child bad behaviour** | 0 Always  1 Not always |
| **birthwet** | Birth weight in (kg) |  |

**Input data**

mage_FCV_5CAT fage_FCV_5CAT offiq21 mumiq21 samepart planned sex mumedu dadedu familyincome mumsmoking bingepreg mumdep breastfeeding6 childattend mother_child csmack childbehab birthwet

=35+ =35+ 111 103 2 1 1 2 2 2 0 1 1 2 0 1 2 0 3.38

<20y 25-<30 109 101 1 2 2 1 3 2 2 2 1 2 0 1 0 2.87

<20y <20y 105 117 2 2 2 2 2 2 0 1 1 0 0 1 2 4.22

25-<30 30-<35 105 2 2 2 3 3 3 0 2 1 2 0 1 3 0 3.96

25-<30 30-<35 123 107 1 2 2 2 2 3 1 2 1 1 1 1 3 1 3.02

<20y 25-<30 117 87 2 2 1 3 3 3 0 1 1 2 1 3.64

<20y 25-<30 94 88 2 1 1 2 2 2 1 1 1 1 1 2 1 3.5

20+yr 105 97 1 2 1 2 2 0 1 1 2 0 1 2 0 3.74

<20y <20y 100 86 1 1 2 2 1 1 2 1 1 0 1 2 1 2.96

20+yr 20+yr 104 95 2 2 2 2 1 1 0 2 1 1 0 1 2 1 3.755

20+yr <20y 105 100 1 2 1 1 0 1 1 0 0 1 2 1 2.66

20+yr <20y 113 92 2 1 1 2 1 0 1 1 0 2 1 4.28

<20y <20y 87 101 2 1 2 3 1 0 1 1 2 0 2 2 3.3

<20y <20y 94 97 2 1 1 1 3 3 0 2 1 1 0 1 2 0 3.14

<20y 25-<30 108 2 2 2 2 2 1 0 1 1 2 0 2 2 0 3.14

25-<30 30-<35 104 108 2 2 2 2 1 0 1 1 2 1 4.38

25-<30 25-<30 101 1 1 1 2 2 2 0 1 1 2 0 1 2 1 3.81

<20y <20y 102 95 2 1 2 2 2 3 0 1 1 1 0 1 2 0 2.75

<20y 30-<35 103 98 2 1 2 1 3 1 0 1 1 2 0 1 2 1 3.5

25-<30 30-<35 93 101 2 2 1 1 2 1 0 1 1 2 0 1 3 1 3.71

<20y <20y 105 1 2 1 2 2 1 1 1 1 0 3 1 4.39

25-<30 =35+ 91 104 2 2 2 2 2 3 2 1 1 1 0 2 3 1 4.27

<20y <20y 102 2 1 1 1 1 1 0 1 1 1 0 1 2 0 3.82

25-<30 25-<30 102 91 2 1 2 2 2 1 1 1 1 1 0 1 2 1 2.34

25-<30 30-<35 96 2 2 1 2 1 2 0 1 1 2 0 2 2 1 3.06

30-<35 =35+ 91 83 2 1 1 1 3 2 0 1 1 2 0 2 3 0 3.18

<20y 25-<30 109 89 2 1 2 1 2 0 1 1 1 0 1 3 0 4.11

20+yr 30-<35 102 1 2 1 1 3 2 2 1 1 0 1 0 2.73

<20y 25-<30 101 104 2 1 2 3 3 3 0 1 1 2 0 1 3 0 3.08

<20y 25-<30 94 94 2 1 1 2 2 3 2 2 1 1 0 1 2 1 3.56

20+yr 25-<30 100 90 2 2 2 2 2 3 0 1 1 1 0 1 2 0 3.86

30-<35 30-<35 124 2 1 2 3 3 1 1 1 2 0 1 3 1 3.62

<20y 25-<30 123 110 2 2 1 3 2 3 1 1 1 2 0 1 2 1 2.69

20+yr 20+yr 118 115 1 2 1 2 2 3 0 1 1 1 0 1 2 1 3.47

<20y 25-<30 100 101 2 1 2 3 2 2 2 1 1 2 0 1 1 1 3.75

<20y <20y 99 81 2 2 1 2 2 3 1 1 1 1 0 1 1 2.98

25-<30 =35+ 100 87 1 2 1 3 2 2 1 1 1 1 0 1 2 1 3.63

25-<30 30-<35 84 87 2 2 2 2 2 2 0 1 1 1 0 1 2 1 5.05

25-<30 30-<35 105 100 2 2 2 2 2 2 0 1 1 2 0 1 2 0 3.64

<20y 25-<30 100 97 2 2 1 3 3 1 1 2 1 1 0 1 2 0 3.76

<20y <20y 103 92 1 2 2 2 2 1 1 2 1 1 0 1 3 0 2.88

<20y <20y 90 86 2 2 2 2 2 1 0 2 1 0 1 2 1 3.42

25-<30 25-<30 97 117 2 2 1 3 2 2 0 1 1 1 0 1 2 0 2.8

<20y 25-<30 114 94 2 1 1 3 3 1 0 1 1 2 0 1 2 0 4.29

<20y <20y 111 94 2 2 1 2 2 2 0 1 1 1 0 1 2 0 3.425

25-<30 30-<35 107 103 2 1 1 3 3 3 0 1 1 2 0 1 2 0 3.96

20+yr 25-<30 101 96 2 2 2 1 2 1 0 1 1 0 0 1 2 1 3.07

20+yr 25-<30 102 2 1 1 2 2 2 1 1 1 2.62

20+yr <20y 104 92 1 2 2 2 2 2 2 1 1 1 2 2 0 3.38

25-<30 =35+ 102 117 2 2 2 3 3 2 0 1 1 1 0 1 2 1 3.65

25-<30 30-<35 88 86 2 1 1 2 1 2 1 1 1 0 0 1 0 3

<20y 25-<30 96 92 2 2 2 2 1 2 0 1 1 1 0 1 1 3.83

<20y 25-<30 80 96 2 1 1 2 1 1 1 1 2 0 1 2 1 2.375

20+yr <20y 91 76 2 2 2 1 1 2 2 1 1 1 0 1 2 1 3.17

30-<35 =35+ 104 105 2 2 2 2 2 2 0 1 1 2 0 1 2 1 2.93

30-<35 =35+ 85 1 1 2 2 2 1 0 1 1 0 1 1 3.41

<20y 30-<35 103 98 1 2 1 1 2 0 1 2 0 1 2 1 2.57

25-<30 30-<35 118 98 2 1 2 2 2 2 0 2 1 2 0 1 2 1 3.09

30-<35 =35+ 111 102 2 1 2 1 3 2 0 1 1 1 0 1 0 3.09

30-<35 <20y 118 2 1 2 3 3 1 0 1 1 2 1 2 0 4.2

<20y 25-<30 107 106 2 1 2 2 3 3 0 1 1 1 0 1 2 1 3.75

=35+ =35+ 99 1 2 2 2 1 3 2 2 1 0 1 2.54

=35+ =35+ 101 98 1 2 2 1 1 3 2 1 1 0 0 2 2 0 3.13

<20y <20y 90 78 1 2 1 2 2 1 2 1 1 0 1 2 1 2.75

=35+ =35+ 110 2 2 1 3 3 2 0 1 1 2 0 1 3 0 3.3

20+yr <20y 107 91 1 2 1 3 3 1 0 1 1 0 0 1 2 0

20+yr <20y 94 88 1 2 2 2 1 2 1 1 2 0 2 1 2.38

20+yr 20+yr 103 87 2 1 1 1 1 1 0 1 1 1 0 1 2 1 3.99

20+yr <20y 108 101 2 2 2 3 2 2 0 1 1 0 0 2 2 1 2.74

30-<35 =35+ 114 81 1 2 1 1 2 1 0 1 1 2 1 1 0 3.5

20+yr <20y 100 94 1 2 2 2 1 1 0 1 1 1 0 1 3 1 2.94

<20y <20y 103 92 2 2 1 2 3 2 1 1 1 2 0 1 2 1 3.48

<20y 25-<30 104 88 1 2 1 1 2 2 1 1 1 0 1 2 1 3.12

20+yr 20+yr 114 95 1 2 1 2 2 1 1 1 1 0 1 1 1 3.73

25-<30 30-<35 105 76 2 1 1 1 3 2 0 1 1 2 0 1 2 1 2.85

25-<30 =35+ 120 2 2 1 2 1 0 1 1 1 0 1 2 1 3.53

<20y 25-<30 111 110 1 1 1 3 3 1 0 1 1 2 1 1 1 0 3.47

<20y 25-<30 99 93 2 2 1 3 2 3 0 1 1 0 0 1 2 1 4.53

30-<35 30-<35 102 85 2 1 1 2 2 3 0 1 1 1 0 1 3 0 3.61

<20y 25-<30 85 97 2 1 2 2 2 2 1 1 1 1 0 1 2 1 3.06

20+yr <20y 88 87 1 2 2 1 3 1 2 2 1 0 1 2.98

25-<30 25-<30 118 114 1 2 2 2 2 2 1 1 1 2 0 1 3 0 2.95

20+yr <20y 110 86 1 2 1 1 1 2 2 1 2 1 0 2 2 1 3.29

<20y 25-<30 94 92 1 2 2 1 1 1 0 1 1 2 0 1 2 1 3.47

25-<30 25-<30 92 78 2 1 1 2 2 2 0 2 1 0 0 1 3 1 2.39

<20y <20y 97 93 2 2 2 1 2 1 0 1 1 1 0 1 2 1 3.503

20+yr <20y 110 109 2 2 2 2 2 1 0 1 1 2 1 3.35

25-<30 30-<35 120 106 2 1 1 2 3 2 0 1 1 2 0 1 2 0 3.02

25-<30 30-<35 108 2 1 1 3 3 1 0 1 2 1 0 2 3 1 3.885

<20y 25-<30 118 94 2 1 1 2 3 3 0 1 1 2 0 1 2 0 3.36

<20y <20y 122 108 1 2 2 2 2 3 1 2 1 1 0 1 2 1 3.7

20+yr 20+yr 98 1 2 1 2 2 3 1 1 1 1 3.56

25-<30 30-<35 94 90 2 1 2 2 3 2 0 1 1 2 0 1 0 3.46

20+yr <20y 97 101 2 2 1 2 2 1 2 1 1 1 0 1 2 0 3.12

25-<30 =35+ 90 87 2 2 1 1 1 1 2 1 1 1 1 2.71

20+yr <20y 114 103 2 2 1 3 3 1 0 1 1 3.39

25-<30 25-<30 117 2 1 1 2 2 1 2 2 1 0 0 1 2 1 3.06

=35+ =35+ 94 91 2 1 2 1 1 2 0 1 1 1 0 1 0 2.46

25-<30 30-<35 103 88 2 2 2 1 1 2 0 1 1 2 0 2 2 0 3.1

<20y <20y 110 2 2 1 2 2 2 1 1 1 0 1 2 0 3

25-<30 25-<30 85 103 2 1 2 2 2 3 1 1 1 1 0 1 1 3.25

<20y 25-<30 100 104 1 2 1 2 2 2 0 1 1 2 0 1 2 1 4.01

<20y 30-<35 107 2 1 2 2 1 3 0 1 1 2 0 1 2 1 3.8

=35+ 111 96 2 3 1 2 1 1 0 1 4.075

<20y 85 2 2 1 1 2 2 1 0 0 1 3 0 3.42

<20y 25-<30 102 2 2 1 1 1 2 1 1 2 0 0 1 2 1 2.83

25-<30 =35+ 122 91 2 1 2 2 2 2 0 1 1 2 0 1 1 3.9

<20y 30-<35 111 1 1 1 1 2 1 0 1 1 2 0 1 2 1 4.32

30-<35 25-<30 117 97 2 1 1 1 2 2 1 2 1 2 0 1 2 0 3.84

30-<35 25-<30 101 120 2 2 2 3 3 2 0 1 1 2 0 1 3 0 3.26

25-<30 =35+ 115 2 1 2 3 1 1 0 1 1 2 2 3.6

20+yr 20+yr 91 1 2 1 2 1 1 0 2 1 1 0 1 2 1 3.255

25-<30 25-<30 108 98 2 1 1 2 2 2 0 1 1 1 0 1 2 1 3.82

25-<30 25-<30 100 91 2 1 1 2 2 3 0 2 1 2 0 1 2 1 3.435

<20y 25-<30 100 91 1 2 1 1 1 1 1 1 1 0 1 2 1 3.79

25-<30 30-<35 103 101 2 1 1 3 3 2 1 1 1 1 0 1 2 0 3.43

25-<30 30-<35 99 98 2 1 2 2 2 3 1 2 1 2 0 1 2 1 3.4

<20y 25-<30 99 2 1 2 3 2 2 2 1 1 2 0 1 2 1 3.155

25-<30 25-<30 102 88 1 2 1 1 2 0 1 1 1 0 1 2 0 2.26

25-<30 <20y 110 93 2 2 2 2 3 2 1 2 1 2 0 1 3 0 3.21

30-<35 =35+ 110 1 2 2 3 1 2 1 2 1 2 0 1 2 0 3.5

<20y 110 90 2 1 2 2 0 2 1 0 1 3 1 4.35

25-<30 25-<30 110 106 2 1 2 2 3 2 0 1 1 2 0 2 3 0 3.25

<20y 25-<30 107 85 2 1 1 2 3 2 0 1 1 1 0 1 2 1 3.5

25-<30 <20y 86 86 1 2 1 2 2 0 1 1 1 0 1 2 1 3.86

25-<30 25-<30 100 1 2 1 2 2 1 0 1 1 1 0 1 2 0 3.24

<20y 25-<30 103 88 1 1 2 2 2 2 0 2 1 0 0 2 2 1 3.6

<20y <20y 97 2 1 1 1 1 1 2 1 2 1 0 2 2 1 2.68

25-<30 104 93 1 2 3 2 1 0 1 1 2 1 1 2 1 3.34

25-<30 30-<35 111 110 2 1 2 2 1 1 0 1 1 2 0 2 2 1 3.46

30-<35 30-<35 102 109 2 2 2 2 2 2 0 1 1 0 0 1 3.77

<20y 25-<30 101 88 2 1 1 2 2 2 1 2 1 0 1 2 0 3.27

<20y 30-<35 113 100 2 1 2 2 2 3 2 1 1 1 0 1 1 0 3.58

25-<30 103 98 1 1 2 3 1 0 1 2 2 0 1 3 1 3.48

20+yr <20y 95 77 2 1 1 1 2 1 2 2 1 1 1 3.51

30-<35 30-<35 109 1 1 2 2 2 2 0 1 1 2 0 1 1 3.34

20+yr <20y 122 109 2 2 2 2 2 3 0 1 1 1 0 1 2 0 3.26

<20y <20y 101 1 2 1 2 2 1 1 2 1 0 1 1 2 0 2.95

20+yr =35+ 101 100 1 1 1 2 2 1 0 1 1 0 0 2 0 2.96

25-<30 <20y 111 96 1 1 2 2 1 3 0 1 2 2 0 2 2 0 3.285

<20y <20y 80 1 2 1 1 1 0 1 1 0 1 3.53

25-<30 25-<30 102 1 1 2 2 2 1 0 1 1 1 0 2 1 0 3.5

<20y <20y 99 92 2 2 1 2 2 3 0 1 1 0 0 1 3 1 3.65

25-<30 25-<30 97 2 2 1 2 2 3 2 1 2 0 0 2 2 0 3.9

25-<30 25-<30 103 96 2 1 1 2 2 1 0 1 2 1 1 2 1 0 2.75

30-<35 =35+ 110 98 2 1 2 2 2 2 0 2 1 0 2 0 2.8

30-<35 30-<35 110 117 2 1 1 3 3 1 0 1 1 2 0 1 3 0 3

25-<30 25-<30 111 2 1 2 2 2 1 0 1 1 2 0 1 2 1 3.93

25-<30 25-<30 100 108 1 1 2 2 2 2 1 1 1 2 0 1 3 1 3.71

30-<35 30-<35 117 94 2 1 1 2 2 2 2 1 1 2 0 1 3 0 3.62

<20y 25-<30 100 86 2 2 2 2 1 0 1 1 2 0 1 2 1 3.56

=35+ =35+ 84 1 2 2 1 1 0 1 1 0 0 1 3 1 3.04

<20y 25-<30 109 2 1 2 3 2 2 0 1 1 2 0 2 2 1 2.97

25-<30 25-<30 110 92 2 1 2 2 2 3 0 2 1 1 0 1 2 1 4.11

<20y 25-<30 89 95 1 1 1 2 2 2 0 2 1 1 0 1 2 1 3.18

30-<35 30-<35 115 117 2 1 2 3 3 2 2 1 1 1 1 2 0 3

30-<35 30-<35 97 100 1 2 2 2 2 2 0 1 1 1 0 1 3 0 3.71

30-<35 =35+ 103 97 2 2 2 1 2 3 0 2 1 1 1 1 2 0 3.19

25-<30 25-<30 111 1 2 1 1 2 2 0 1 1 1 0 1 2 1 4.08

<20y 100 91 2 2 2 2 2 1 0 1 1 2 0 1 2 0 2.56

25-<30 30-<35 100 84 2 2 2 2 1 2 2 1 1 2 0 1 2 1 3.25

20+yr 107 112 1 2 2 3 1 2 1 2 1 0 1 3 0 2.79

25-<30 25-<30 115 101 2 1 1 2 2 2 0 1 1 2 0 1 2 0 3.36

20+yr 25-<30 96 82 2 2 2 1 3 2 2 1 1 0 1 1 2 1 2.83

20+yr 25-<30 110 2 1 2 2 2 1 0 1 1 1 1 1 1 0 3.37

<20y 25-<30 115 98 2 1 2 2 2 3 0 2 1 1 1 1 2 1 3.68

=35+ =35+ 117 109 1 2 2 2 3 1 0 1 1 2 0 1 3 0 3.8

25-<30 30-<35 110 108 2 1 1 3 3 1 2 1 1 2 0 1 2 0 3.98

<20y 25-<30 108 2 1 2 3 2 3 0 2 1 1 0 1 1 3.65

20+yr 25-<30 94 2 2 2 1 2 1 1 0 2 0 3.02

<20y <20y 112 97 2 2 1 3 3 1 0 1 1 1 1 3.73

20+yr <20y 117 2 2 1 2 2 1 1 1 1 1 1 1 2 1 4.43

25-<30 25-<30 114 106 2 1 1 3 3 3 0 1 1 0 0 1 2 0 3.8

20+yr <20y 89 2 2 2 2 2 1 1 2 1 2 0 1 0 2.99

20+yr 25-<30 120 107 2 2 1 2 2 3 0 1 1 2 0 1 3 0 2.92

25-<30 <20y 112 101 2 2 1 2 1 2 1 1 2 0 1 1 2.5

25-<30 25-<30 100 2 1 1 2 3 3 2 1 1 2 0 1 2 0 3.48

<20y 25-<30 101 93 2 1 2 2 1 0 1 1 2 0 2 3 1 3.83

<20y 25-<30 107 106 2 2 1 2 2 2 2 2 1 2 0 1 2 0 4.32

<20y <20y 104 96 2 2 2 2 2 2 0 1 1 2 0 1 2 0 3.925

25-<30 25-<30 100 94 2 2 1 2 2 2 0 1 1 1 0 1 2 0 3.9

20+yr 20+yr 105 101 1 2 2 2 2 1 2 1 1 1 2 2.67

<20y <20y 108 95 1 1 1 2 2 1 1 1 1 2 0 1 3 1 3.43

<20y 25-<30 103 90 1 2 1 1 1 1 0 1 1 2 1 1 2 0 4.34

<20y 25-<30 91 101 1 1 1 2 1 0 1 1 1 0 1 2 1 3.085

<20y 25-<30 80 112 2 1 2 1 1 1 1 1 1 1 1 2 0 2.805

20+yr 20+yr 78 82 2 2 2 1 1 2 2 1 1 0 0 1 2 1 2.86

25-<30 <20y 99 93 2 2 1 1 1 1 0 1 1 0 0 1 2 0 2.99

<20y =35+ 100 2 2 2 1 1 2 1 1 2 2 0 1 3 0 4.08

<20y 30-<35 97 96 2 2 2 2 2 1 0 1 1 0 0 1 3 0 3.17

<20y 30-<35 90 98 2 2 3 1 2 0 2 1 0 2 2.97

25-<30 25-<30 98 83 2 1 2 2 2 3 1 1 1 1 1 1 2 0 2.86

25-<30 25-<30 105 96 2 2 2 2 3 1 0 1 1 0 0 1 2 0 3.31

20+yr 20+yr 99 1 2 2 2 2 1 2 1 1 1 0 1 2 0 3.34

20+yr <20y 107 2 2 3 2 1 2 1 1 1 0 1 2 0 2.7

25-<30 30-<35 94 93 2 2 2 2 2 3 1 2 1 1 0 1 2 0 3.28

<20y <20y 94 95 1 2 1 2 2 1 0 2 1 1 0 1 2 1 3.09

30-<35 =35+ 110 120 1 2 2 1 1 0 1 1 1 0 2 0 4.13

25-<30 25-<30 122 114 2 1 1 3 2 1 0 1 1 2 0 1 2 0 3.65

30-<35 25-<30 99 2 2 1 2 2 3 1 1 2 2 0 1 1 0 3.07

<20y 25-<30 100 1 1 2 2 2 3 0 2 1 2 0 1 2 0 3.63

=35+ =35+ 108 88 2 2 2 2 2 1 0 1 1 2 1 1 2 0 3.25

25-<30 30-<35 102 95 2 1 1 3 3 3 2 1 1 1 0 1 2 1 3.16

<20y 25-<30 88 86 2 1 1 2 2 1 0 2 1 1 0 2 2 0 3.4

30-<35 =35+ 99 99 2 1 2 3 2 2 1 2 0 1 2 0 3.62

25-<30 25-<30 109 108 2 2 2 2 1 3 0 1 1 2 0 2 2 1 3.83

25-<30 25-<30 122 119 2 2 1 2 3 2 0 1 1 2 1 1 1 1 2.82

<20y 25-<30 110 90 2 2 1 1 2 2 0 1 1 1 1 1 1 1 3.37

20+yr <20y 106 99 2 2 2 3 3 2 1 1 1 2 0 1 2 0 3.76

25-<30 30-<35 96 103 2 2 1 2 2 1 2 1 1 0 1 1 3 0 3.38

30-<35 =35+ 100 93 2 2 1 3 2 3 0 1 1 2 0 1 2.94

20+yr <20y 90 1 2 2 1 1 1 2 1 2 2 0 1 2 1 3.43

=35+ 30-<35 103 1 2 1 1 1 1 2 1 1 0 0 1 2 0 2.94

<20y <20y 104 104 2 1 2 2 1 0 2 1 1 0 1 2 1 3.57

25-<30 =35+ 82 100 2 1 1 3 2 1 1 2 0 1 2 0 2.52

20+yr <20y 84 111 1 1 2 3 1 1 1 2 1 1 0 1 3 0 2.93

25-<30 <20y 110 94 1 2 2 3 3 2 0 1 1 0 0 1 2 1 3.14

<20y 25-<30 108 100 2 1 2 2 2 3 2 1 1 2 0 2 2 0 3.04

20+yr 99 96 1 2 2 2 2 1 1 1 1 0 0 1 2 1 3.33

<20y <20y 107 97 2 2 2 2 2 1 2 1 1 1 0 1 2 3.34

<20y 25-<30 111 1 1 2 2 2 3 0 1 1 0 0 1 2 1 3.84

<20y 25-<30 93 86 2 1 1 1 2 2 0 2 1 2 0 1 2 1 3.17

<20y 30-<35 106 114 1 1 3 1 2 0 1 1 2 0 1 2 0 4.6

30-<35 30-<35 102 90 1 2 2 2 2 2 0 1 1 1 1 3 1 3.87

30-<35 30-<35 101 81 2 1 1 2 3 2 0 1 1 1 1 2 3 1 3.6

25-<30 25-<30 102 2 1 1 3 3 2 0 1 1 2 0 1 3 0 2.43

<20y <20y 114 103 2 1 2 2 2 3 0 1 1 2 0 1 1 3.125

25-<30 30-<35 94 100 2 2 2 2 2 0 1 1 1 1 1 2 1 3.4

<20y 25-<30 103 108 2 1 1 2 2 2 0 2 1 2 0 1 2 0 3.83

25-<30 =35+ 107 89 2 1 1 2 2 0 1 1 2 0 1 2 0 3.87

25-<30 25-<30 101 2 2 2 3 2 2 0 1 1 2 0 1 2 0 3.66

20+yr <20y 89 2 2 1 2 2 2 0 1 1 0 0 2 2 1 3

=35+ 30-<35 104 2 2 2 3 3 2 0 1 1 1 0 1 3 0 3.63

25-<30 30-<35 103 105 2 2 1 2 2 2 0 1 1 0 1 4.43

<20y <20y 111 96 2 2 2 2 1 2 0 1 1 2 1 1 1 3.17

25-<30 25-<30 103 81 2 1 2 1 1 1 2 2 1 1 0 2 2 1 3.62

<20y 25-<30 104 93 2 1 2 2 2 1 0 1 1 0 0 1 2 1 3.24

25-<30 30-<35 105 98 2 1 1 2 2 2 0 1 1 1 1 1 2 0 4.49

<20y 89 2 1 1 2 1 1 0 1 1 0 0 1 2 0 2.835

25-<30 30-<35 117 2 2 2 3 3 3 0 1 1 2 0 1 2 0 3.5

<20y <20y 94 84 2 2 2 2 2 3 1 2 1 1 0 1 2 0 2.65

25-<30 30-<35 109 98 2 2 1 2 2 1 0 2 1 0 0 1 2 1 3.18

<20y 30-<35 103 87 2 1 2 2 2 2 2 2 2 1 3 0 3.38

25-<30 30-<35 93 96 2 1 2 2 1 2 0 1 1 0 1 1 2 3.44

25-<30 25-<30 110 106 2 2 2 2 2 2 0 1 1 2 1 1 2 1 3.37

25-<30 30-<35 94 119 2 2 1 3 3 2 0 1 1 2 0 1 2 0 3.95

25-<30 106 93 2 2 1 2 0 1 1 0 1 2 2 0 4.25

20+yr <20y 107 115 2 2 1 2 2 2 0 1 1 2 0 1 2 0 3.08

25-<30 30-<35 98 101 2 1 1 1 2 3 2 2 1 1 0 1 2 0 4.71

20+yr 115 1 2 2 2 0 1 1 1 0 1 3 0 3.03

<20y 25-<30 112 106 2 1 2 2 2 2 0 1 1 1 1 2 0 3.46

20+yr <20y 102 83 2 2 1 2 2 1 1 2 1 0 0 1 1 0 2.4

30-<35 30-<35 109 88 2 2 1 2 1 2 0 1 1 2 0 1 2 0 3.26

<20y <20y 100 2 2 1 2 1 2 1 1 1 1 0 1 2 0 4.03

30-<35 =35+ 115 107 2 2 2 2 2 2 0 1 1 0 0 1 2 0 3.71

25-<30 30-<35 114 98 2 1 1 1 2 3 0 1 1 1 2 0 3.34

25-<30 =35+ 99 92 1 1 1 2 1 2 0 1 1 0 0 1 2 0 3.25

20+yr <20y 108 99 2 2 1 2 2 2 2 1 1 0 1 3.71

<20y <20y 104 89 2 1 1 2 2 2 0 1 1 1 0 1 3 0 3.87

25-<30 30-<35 108 96 1 1 2 2 0 1 1 2 1 1 3 0 3.44

<20y <20y 100 92 2 2 1 2 2 1 0 1 1 2 0 1 2 0 3.38

<20y <20y 105 106 1 1 2 3 1 2 0 1 1 1 0 1 2 1 3.27

<20y 25-<30 98 101 2 2 2 3 2 2 0 1 1 2 0 1 2 1 3.49

25-<30 25-<30 110 104 2 2 2 3 2 2 0 1 1 1 0 1 2 1 3.24

25-<30 25-<30 101 100 2 1 2 2 2 1 1 2 1 2 0 2 2 1 2.54

30-<35 =35+ 102 97 2 2 2 2 2 2 2 2 1 2 0 3.96

25-<30 25-<30 122 90 2 1 1 2 2 2 0 2 2 1 0 2 2 1 3.97

30-<35 30-<35 123 103 2 1 2 3 2 2 0 2 1 2 0 1 2 1 4.32

25-<30 =35+ 85 86 2 1 1 1 1 2 0 1 1 2 0 1 2 0 3.88

30-<35 30-<35 115 109 2 2 2 3 2 0 1 1 1 1 1 1 0 4.32

30-<35 =35+ 102 99 2 2 1 3 1 2 0 1 1 2 0 1 2 1 1.98

30-<35 =35+ 109 114 2 2 2 2 3 2 0 1 1 1 0 1 3 0 3.67

25-<30 30-<35 81 2 2 2 2 2 2 2 1 2 0 1 2 1 3.77

30-<35 <20y 115 107 2 1 2 2 2 3 2 1 1 1 1 1 2 1 3.305

25-<30 30-<35 105 100 2 2 2 3 3 3 0 1 1 0 0 2 2 0 3.03

30-<35 =35+ 111 80 2 2 1 3 3 1 0 1 1 1 2 3 0 3.02

25-<30 30-<35 109 98 2 1 2 3 3 3 0 1 1 2 0 1 0 3.29

25-<30 30-<35 110 98 1 2 2 1 2 1 0 1 1 2 1 1 3.48

25-<30 25-<30 104 101 2 2 2 2 2 3 1 1 1 2 0 1 3 0 2.96

25-<30 25-<30 96 91 1 2 1 2 1 2 0 1 1 2 2 1 0 3.46

<20y 25-<30 103 92 2 1 1 2 1 2 0 1 2 1 1 1 2 1 3.82

25-<30 25-<30 114 81 2 2 2 2 1 1 2 2 1 1 0 1 0 2.74

25-<30 25-<30 100 94 1 1 1 2 2 2 0 1 1 0 2 0 3.51

30-<35 =35+ 118 102 2 1 2 3 2 2 0 1 1 2 0 1 3 1 3.08

30-<35 25-<30 120 147 2 1 2 3 3 1 0 1 1 2 0 1 2 0 3.51

25-<30 30-<35 100 108 2 1 1 2 1 2 0 1 1 2 0 2 2 0 3

<20y <20y 102 91 2 1 2 2 2 1 0 1 1 1 0 1 2 0 3.42

<20y 25-<30 83 80 2 1 1 2 2 3 1 2 1 1 1 2.98

<20y 30-<35 105 117 1 2 2 3 2 1 0 1 1 2 1 2 2 1 3.36

30-<35 =35+ 125 2 1 2 2 2 0 1 1 2 0 1 3 0 3.28

20+yr <20y 108 1 1 2 2 2 1 0 1 1 2 0 1 2 0 3.17

25-<30 25-<30 100 92 2 2 2 2 2 2 0 1 1 2 0 1 3 1 3.1

30-<35 30-<35 101 98 2 2 2 2 2 2 0 2 1 1 0 1 1 1 3.19

<20y <20y 109 88 1 2 1 3 2 1 0 1 1 1 0 1 3.32

=35+ =35+ 92 95 2 2 2 2 1 2 0 1 1 0 1 1 2 0 4.55

30-<35 =35+ 114 114 1 2 2 2 3 3 1 1 1 2 0 1 2 0 2.9

25-<30 30-<35 122 117 2 1 1 3 3 3 0 1 1 2 0 1 3 0 4

25-<30 25-<30 109 103 2 2 1 3 3 2 0 1 1 1 0 1 2 0 3.72

<20y 30-<35 92 2 1 1 2 2 2 1 2 1 0 2 1 3.16

<20y 30-<35 98 96 2 2 1 2 2 2 0 1 1 2 0 1 3 3.35

<20y <20y 93 85 2 2 2 1 2 1 1 1 1 0 1 1 2 0 3.97

<20y <20y 100 112 2 2 2 3 3 3 0 1 1 2 0 1 2 0 3.02

30-<35 =35+ 91 2 2 2 1 2 2 2 1 1 0 1 3.41

30-<35 30-<35 108 103 1 2 3 2 1 0 1 1 1 1 2.49

20+yr <20y 96 88 2 2 1 2 1 0 2 1 2 0 1 2 1 3.49

<20y <20y 111 92 2 1 1 3 2 3 0 1 1 2 0 1 2 1 3.39

25-<30 25-<30 120 95 2 1 1 1 2 2 0 1 1 2 0 1 2 1 3.51

25-<30 25-<30 105 2 1 2 2 2 2 0 1 1 2 1 1 2 1 3.53

<20y <20y 83 1 2 1 2 2 2 1 1 1 0 2 3.71

25-<30 30-<35 117 112 1 2 1 2 2 3 0 1 1 1 0 1 2 0 3.75

20+yr 20+yr 97 100 2 1 2 2 3 1 0 1 1 2 1 1 2 1 4.38

30-<35 =35+ 100 103 2 1 1 3 1 2 0 1 1 2 1 2 2 1 4.51

<20y <20y 108 100 2 1 1 2 2 3 0 1 1 2 0 1 2 0 3.565

25-<30 25-<30 105 2 1 2 2 3 3 0 1 1 2 0 1 1 1 3.48

25-<30 25-<30 115 94 2 2 1 2 2 2 0 1 1 0 0 1 2 1 3.07

<20y 25-<30 89 98 2 2 2 2 2 2 0 1 1 0 0 1 2 0 3.195

30-<35 30-<35 98 2 2 2 1 2 2 0 1 1 1 1 1 3 1 3.1

25-<30 25-<30 83 92 2 2 2 1 2 2 2 1 2 0 1 2 1 4.11

25-<30 30-<35 107 97 2 2 1 3 1 2 0 1 1 2 0 2 2 1 4.12

30-<35 =35+ 106 95 2 1 1 3 1 3 0 1 1 2 1 2 2 0 3.78

30-<35 30-<35 134 99 2 2 1 2 2 2 2 2 1 2 0 1 2 0 2.45

30-<35 25-<30 84 84 1 2 1 2 1 0 1 1 1 0 1 2 0 3.18

30-<35 30-<35 110 107 2 2 1 2 2 2 0 1 1 2 0 1 2 0 4.3

<20y 97 95 2 1 2 1 1 2 2 0 3.04

<20y 30-<35 103 2 2 2 2 2 3 0 1 1 1 1 3.45

<20y <20y 104 93 1 2 2 2 3 3 0 1 1 2 0 1 2 0 4.25

30-<35 =35+ 90 105 2 2 2 2 2 1 0 1 1 2 0 1 2 0 3.503

<20y 25-<30 118 110 2 2 1 2 3 2 0 1 1 2 0 1 3 0 3.36

20+yr 110 104 1 2 1 2 2 1 1 1 1 0 1 2 0 3.31

30-<35 =35+ 115 107 2 1 2 2 1 0 1 1 0 0 1 2 0 3.3

<20y 25-<30 92 85 2 2 2 1 2 3 1 1 1 0 0 1 2 1 3.27

<20y 25-<30 115 1 2 1 3 2 2 1 1 1 1 1 2 0 3.68

20+yr 20+yr 107 1 2 1 2 2 2 0 1 1 2 0 1 2 0 3.42

<20y <20y 106 2 1 2 2 2 2 0 1 1 2 1 3.17

<20y 25-<30 94 92 2 1 1 2 2 1 0 1 2 0 1 2 0 3.36

<20y 105 88 1 2 1 2 2 1 2 1 1 2 1 1 1 3.105

30-<35 =35+ 117 87 2 1 1 2 2 0 1 1 2 0 2 3 1 3.92

<20y <20y 97 81 2 1 2 1 1 2 0 1 1 0 1 1 2 1 3.01

20+yr <20y 111 2 2 2 2 1 0 1 1 0 1 1 2.9

<20y 25-<30 104 2 2 1 2 2 2 1 2 1 2 1 3.245

<20y <20y 110 98 2 2 2 2 2 3 0 2 1 2 0 1 2 0 2.62

25-<30 30-<35 103 81 2 1 3 3 3 0 1 1 1 0 1 2 4.16

<20y <20y 105 89 2 1 1 2 2 2 0 1 1 1 0 1 2 0 2.54

<20y <20y 100 2 1 2 2 2 2 0 1 1 0 2 1 3.3

30-<35 30-<35 90 89 2 2 2 2 2 1 2 1 1 1 1 1 2 0 3.44

=35+ =35+ 101 88 1 2 1 1 1 1 2 1 2 0 0 2 0 3.37

<20y <20y 87 83 1 1 1 1 2 1 1 2 1 1 1 2 2 1 3.81

20+yr <20y 102 88 2 1 2 2 2 2 0 1 1 0 0 1 2 1 3.64

<20y <20y 111 1 2 2 2 2 2 1 2 0 0 1 2 1 2.68

25-<30 25-<30 127 122 2 1 2 2 3 0 1 1 2 0 1 2 0 3.65

<20y 25-<30 87 84 1 1 1 1 1 1 2 1 1 1 1 1 3 0 3.39

25-<30 30-<35 94 81 1 1 2 1 3 2 2 2 1 1 1 3.31

25-<30 30-<35 114 103 2 1 1 2 2 2 0 1 1 2 0 1 2 1 3

<20y <20y 101 90 1 2 3 3 1 2 2 1 3.39

25-<30 =35+ 88 94 2 1 2 2 2 2 0 1 1 2 2 3.49

<20y 25-<30 80 52 1 2 2 3 3 1 0 1 1 0 0 1 3 0 4.24

<20y <20y 117 87 1 2 2 2 2 1 1 2 1 0 1 1 0 3.27

<20y 25-<30 123 101 2 1 2 2 3 1 0 1 1 0 1 1 3.475

<20y 30-<35 91 86 2 2 1 2 1 1 0 2 1 1 0 1 2 1 3.48

25-<30 25-<30 103 2 2 2 2 3 3 0 1 1 2 0 1 2 1 4.17

20+yr <20y 96 101 1 2 2 2 2 1 1 1 0 2 1 3.28

25-<30 25-<30 122 104 2 1 1 2 2 2 2 2 1 2 0 1 2 1 3.3

20+yr 20+yr 115 1 2 1 2 2 1 0 1 1 2 1 1 2 0 3.5

20+yr 25-<30 113 2 2 2 2 2 1 0 1 1 2 0 2 2 0 3.14

20+yr <20y 115 2 1 1 2 3 1 0 1 1 1 3.43

<20y 25-<30 88 2 1 1 2 2 2 1 1 1 0 1 0 2.8

25-<30 25-<30 114 92 2 1 1 1 1 1 0 1 1 1 2 3.58

<20y <20y 99 87 2 2 2 2 1 1 2 2 1 0 1 1 2 1 3.69

20+yr 102 96 1 2 2 1 1 0 1 1 2 1 3.13

=35+ =35+ 82 90 2 2 1 2 2 3 0 1 1 0 2 1 3.24

<20y 25-<30 92 88 2 2 1 2 2 2 1 2 1 1 1 1 2 1 2.78

=35+ =35+ 105 2 2 1 2 1 3 2 2 1 2 1 1 0 2.82

=35+ =35+ 103 99 2 2 2 2 2 2 0 1 1 1 0 1 3 0 4.37

<20y 25-<30 98 93 2 2 2 3 3 3 0 1 1 2 0 1 3 1 3.3

<20y 25-<30 111 101 1 2 2 1 2 1 1 1 1 1 1 3.61

<20y <20y 94 83 2 1 1 2 2 1 0 1 1 2 0 1 1 3.49

25-<30 25-<30 102 108 2 2 2 2 3 2 2 1 1 1 0 1 2 1 2.79

25-<30 25-<30 105 2 2 2 2 2 2 0 1 1 2 0 1 2 0 3.08

25-<30 25-<30 114 2 2 2 3 3 2 2 1 1 2 0 1 2 0 2.94

25-<30 25-<30 113 1 1 1 3 3 2 0 1 1 1 1 1 2 0 4.15

30-<35 30-<35 104 102 1 2 2 1 1 2 1 1 1 0 0 1 1 0 2.95

20+yr 20+yr 92 111 2 1 1 3 2 2 0 1 2 2 1 3.47

25-<30 25-<30 100 89 2 2 2 2 2 2 0 1 1 2 0 2 1 1 2.96

25-<30 30-<35 106 2 1 1 3 2 3 0 1 1 2 1 1 3 0 3.86

25-<30 30-<35 112 95 2 2 1 2 2 1 0 1 1 1 0 1 2 0 3.66

<20y 25-<30 108 100 2 2 2 3 3 2 0 1 1 2 0 1 2 0 3.94

25-<30 30-<35 103 103 1 2 2 2 1 3 2 2 1 0 1 2 0 2.92

<20y 25-<30 81 1 1 2 1 2 1 0 1 1 0 0 1 3 1 3.14

25-<30 25-<30 107 89 2 2 2 3 2 2 0 2 1 2 1 2 2 2.98

25-<30 30-<35 111 1 2 3 3 2 0 2 1 2 2 3.31

20+yr <20y 98 115 1 2 2 3 2 3 1 1 1 1 1 1 3 1 2.83

25-<30 25-<30 108 96 2 2 2 1 1 1 0 1 1 1 0 1 0 3.38

25-<30 102 2 1 1 1 3 2 0 1 1 2 0 1 2 1 3.35

<20y 25-<30 106 2 2 1 2 1 1 0 2 1 2 1 2 3 1 3.44

=35+ =35+ 87 93 2 1 2 1 1 2 0 1 1 1 0 1 3.64

<20y <20y 104 88 2 2 1 1 1 1 1 2 1 1 1 1 2 1 3.23

20+yr <20y 91 96 1 2 2 1 1 1 1 2 1 0 1 1 2 0 3.33

25-<30 30-<35 81 88 2 2 1 2 1 1 1 1 1 1 1 1 2 1 3.38

25-<30 30-<35 111 98 2 2 1 3 1 1 0 2 1 2 1 1 2 1 3.355

30-<35 <20y 108 106 1 2 2 2 2 2 0 1 1 0 0 1 2 1 2.86

25-<30 =35+ 103 1 2 2 3 3 3 0 1 2 2 1 2 2 1 3.2

<20y 25-<30 99 98 2 1 2 2 3 2 0 1 1 2 1 1 3 1 3.76

20+yr <20y 76 81 2 2 1 1 2 1 0 1 1 1 0 1 2 1 3.96

=35+ 30-<35 124 103 2 1 1 2 3 1 0 1 1 1 0 2 2 0 3.5

20+yr <20y 100 1 2 1 3 3 2 1 2 1 2 1 1 2 0 2.93

<20y <20y 102 84 2 2 1 1 2 1 1 2 1 0 1 1 2 1 3.73

<20y <20y 88 2 1 2 1 1 1 0 2 1 0 0 1 2 1 2.78

<20y <20y 114 2 1 3 2 3 0 1 1 2 0 1 3 1 3.6

<20y 25-<30 105 108 2 2 2 3 3 2 0 1 1 2 0 2 2 0 3.38

25-<30 30-<35 105 1 1 1 3 2 0 1 1 2 0 1 3 1 3.405

25-<30 30-<35 101 2 2 1 2 2 2 0 1 1 1 1 1 2 1 3.33

25-<30 30-<35 115 86 2 2 1 2 2 1 2 2 1 1 2 3.29

<20y <20y 110 2 2 1 3 2 1 0 1 0 0 1 2 1 3.78

20+yr 20+yr 101 1 2 1 2 3 0 1 1 1 1 1 2 0 4.56

25-<30 25-<30 80 96 2 2 2 2 1 1 0 2 1 1 2 0 3.44

=35+ =35+ 114 91 2 2 1 1 2 1 0 1 1 2 0 2 2 1 3.62

<20y 25-<30 112 100 2 2 2 2 3 3 2 1 1 2 1 1 2 0 3.29

<20y 25-<30 85 93 2 2 1 1 1 1 2 1 1 1 0 1 2 1 3.57

<20y 25-<30 104 94 2 2 2 3 2 3 0 2 1 2 1 1 2 1 3.11

<20y <20y 105 1 1 1 2 2 3 0 1 1 0 0 1 3 0 3.09

20+yr 84 92 2 2 1 2 1 0 1 2 0 0 1 1 1 3.39

20+yr <20y 103 98 2 2 1 2 2 1 2 2 1 1 0 1 2 1 3.8

25-<30 25-<30 114 104 2 1 2 1 2 0 1 1 0 1 1 3 0 3.45

25-<30 25-<30 101 90 2 1 2 2 2 2 0 1 1 2 0 1 2 0 3.84

<20y 25-<30 114 2 2 1 3 3 2 0 2 1 2 0 2 2 0 3.37

<20y <20y 96 93 1 1 2 2 2 1 1 2 1 0 1 3.74

<20y 86 104 1 2 2 2 1 1 1 1 1 0 2 2 0 3.91

25-<30 25-<30 86 88 2 1 2 3 3 2 0 1 1 2 0 1 2 1 2.73

20+yr <20y 104 2 2 2 1 2 2 0 2 1 1 1 1 2 1 3.63

<20y 25-<30 85 82 2 2 1 2 2 2 1 1 1 1 0 2 2 1 3.34

<20y <20y 79 85 2 2 1 2 2 2 0 1 1 0 0 1 3 0 3.14

30-<35 =35+ 103 100 1 2 1 1 1 1 2 2 1 1 0 1 2 1 2.81

30-<35 =35+ 110 98 2 1 1 2 1 1 2 2 1 0 0 1 0 2.685

<20y 25-<30 99 2 1 1 2 2 2 0 1 1 2 0 1 0 3.485

<20y 25-<30 99 2 2 1 3 2 1 0 1 1 1 0 1 2 0 3.73

25-<30 25-<30 107 93 2 1 2 2 3 1 2 1 1 2 0 1 2 0 2.66

30-<35 30-<35 114 2 1 2 2 2 2 0 1 1 2 1 1 1 3.63

30-<35 25-<30 117 95 1 1 1 1 1 3 1 2 1 0 1 3.53

30-<35 25-<30 104 94 1 2 1 3 2 2 1 1 1 1 0 1 2 1 2.48

20+yr 25-<30 97 2 2 2 2 2 1 1 1 0 1 1 2 1 3.31

30-<35 30-<35 102 91 2 1 2 2 1 1 0 1 1 2 0 1 0 3.33

25-<30 25-<30 107 2 1 2 2 2 0 1 1 0 1 1 2 0 3.52

25-<30 30-<35 112 2 2 2 3 3 2 0 1 1 2 1 1 2 0 3.08

30-<35 =35+ 122 102 1 2 1 3 1 3 2 1 1 1 0 1 3 1 3.1

25-<30 25-<30 82 89 2 2 1 2 2 2 2 2 1 2 1 2 2 1 3.27

<20y <20y 99 97 2 1 2 2 2 2 0 1 1 0 0 1 0 3.78

<20y 25-<30 97 2 2 2 2 2 1 0 2 1 1 1 1 2 0 3.19

30-<35 =35+ 94 98 1 2 1 2 2 2 0 1 1 2 0 1 2 1 3.74

<20y 30-<35 112 92 1 1 2 3 3 0 1 1 1 1 1 3 0 4.07

<20y <20y 98 89 2 2 2 2 3 3 0 1 1 2 1 1 1 0 4.12

30-<35 25-<30 114 2 2 2 3 3 3 2 1 1 1 0 1 2 1 3.49

25-<30 =35+ 111 94 1 1 2 1 1 2 0 1 1 1 1 1 2 0 3.36

<20y 25-<30 101 103 2 2 2 2 2 3 2 2 1 2 0 1 1 2.2

20+yr 20+yr 114 94 1 2 1 1 1 1 1 2 1 0 1 1 2 1 4.05

<20y 25-<30 102 2 2 2 2 2 3 2 1 1 2 0 1 2 1 3.09

=35+ =35+ 107 100 2 2 2 1 1 2 0 1 1 0 0 1 2 0 3.34

<20y 25-<30 107 104 2 2 1 2 2 1 0 1 1 2 0 1 2 0 2.98

<20y 25-<30 104 2 1 1 2 3 2 0 1 1 1 1 1 2 1 2.95

20+yr 20+yr 94 117 1 2 2 2 2 1 1 2 2 2 1 1 2 0 3.795

<20y 25-<30 109 90 2 2 1 2 2 3 0 1 1 2 0 1 2 1 2.93

<20y 111 94 2 1 1 2 2 2 0 1 1 0 2 1 3.35

20+yr 25-<30 85 2 2 2 1 1 2 2 1 1 0 2 2 1 3.2

30-<35 30-<35 92 1 2 2 1 2 2 2 1 1 1 1 1 2 0 3.26

20+yr 20+yr 106 2 2 1 2 2 3 1 1 1 1 0 1 2 1 4.38

<20y <20y 113 110 2 2 1 2 2 2 0 1 1 2 0 1 2 0 3.66

25-<30 30-<35 111 2 2 1 3 2 1 0 1 1 2 0 2 2 1 3.39

20+yr <20y 91 90 2 2 1 2 3 1 0 1 1 2 0 1 1 0 3

20+yr <20y 98 90 2 2 2 3 2 2 0 2 1 1 0 1 2 1 4.04

20+yr 30-<35 114 90 2 2 2 2 3 2 1 1 1 1 1 3.48

20+yr 20+yr 101 92 1 1 2 1 2 1 0 1 1 1 1 1 3 0 3.48

25-<30 25-<30 110 88 2 1 2 2 3 2 0 1 1 2 1 2 3 0 3.08

25-<30 30-<35 97 97 2 1 2 3 2 2 0 1 1 1 0 1 2 0 3.01

<20y 25-<30 98 2 1 2 2 2 2 2 2 1 1 2 1 3.2

<20y 25-<30 126 88 2 1 1 2 2 2 0 2 1 1 1 1 2 1 2.66

25-<30 30-<35 95 83 2 2 2 1 1 1 0 2 1 2 0 1 3 0 3.41

<20y 25-<30 107 101 1 1 2 2 2 1 0 1 1 2 1 2 2 1 3.16

<20y <20y 103 96 2 1 1 2 2 2 0 1 1 1 1 3.5

<20y 25-<30 104 88 2 2 1 2 3 2 0 1 2 0 0 1 2 1 3.72

25-<30 30-<35 85 90 2 1 1 2 3 2 0 1 1 0 0 1 3 1 2.89

25-<30 25-<30 91 93 2 2 1 2 1 1 0 1 1 2 0 1 2 1 3.74

20+yr 25-<30 78 76 1 1 1 1 1 2 1 1 1 0 1 2 1 2.29

<20y <20y 95 101 2 2 2 3 2 3 0 1 1 1 0 1 1 0 3.31

<20y 25-<30 100 88 2 1 1 3 3 2 2 1 1 1 1 2 0 3.45

20+yr <20y 98 97 1 2 2 2 2 1 0 1 1 1 0 1 2 0 3.11

<20y <20y 114 108 2 2 1 3 3 3 0 1 1 2 1 1 2 1 3.52

<20y <20y 100 50 2 2 1 1 1 2 1 1 1 0 2 1 3.09

30-<35 25-<30 114 2 2 1 2 2 1 0 1 1 2 0 1 2 0 3.75

<20y 25-<30 91 97 2 1 2 2 2 2 2 1 1 2 1 1 1 3.91

20+yr <20y 98 102 1 1 2 2 2 3 0 1 1 1 0 1 0 3.51

25-<30 30-<35 118 101 1 1 2 2 1 2 2 1 1 1 2 1 2.5

25-<30 <20y 96 81 2 2 2 2 2 1 2 2 1 1 0 1 2 1 2.27

20+yr 102 99 1 2 2 3 1 2 1 1 2 0 1 2 0 3.48

<20y <20y 123 117 1 1 1 2 2 3 2 2 1 0 0 1 2 1 3.18

<20y <20y 94 2 1 2 1 1 3 1 1 1 0 1 1 2 1 3.17

25-<30 30-<35 100 77 1 1 2 2 1 0 2 1 0 0 1 2 0 3.69

30-<35 =35+ 110 2 1 2 3 3 2 0 1 1 1 1 1 1 0 3.38

30-<35 30-<35 94 87 1 1 2 1 2 1 0 1 1 1 1 1 3 1 3.13

<20y <20y 111 110 2 2 2 2 2 1 1 2 1 1 2 0 3.51

<20y 25-<30 59 90 1 1 2 2 2 2 1 1 1 0 1 2 0 3.44

<20y 25-<30 82 88 2 2 1 2 2 1 0 1 1 2 1 1 2 1 3.94

<20y <20y 108 97 2 2 1 3 2 1 2 2 1 1 1 1 1 0 3.44

=35+ =35+ 117 147 2 1 1 3 2 3 0 1 1 2 1 1 2 0 4.07

25-<30 25-<30 104 100 2 1 2 2 2 1 0 1 1 0 1 2 2 0 3.25

<20y =35+ 101 103 1 2 2 2 2 1 2 1 1 2 1 1 3 0 2.82

<20y <20y 86 2 2 1 1 2 2 0 1 2 1 1 2.61

<20y <20y 113 95 1 1 2 2 2 3 2 1 1 1 0 1 2 1 3.3

25-<30 25-<30 115 119 2 1 2 2 2 2 0 1 1 2 0 1 3 0 3.83

30-<35 30-<35 114 98 2 2 1 2 1 3 0 1 1 2 1 1 3 1 3.54

<20y 25-<30 108 106 1 2 1 2 2 3 2 2 1 0 1 2.93

25-<30 25-<30 105 106 2 1 2 2 2 1 1 2 1 1 1 1 2 0 2.91

<20y <20y 118 1 2 1 2 3 1 0 1 1 2 1 1 3.73

<20y 97 103 1 2 2 2 1 2 2 1 0 0 1 2 0 3.01

25-<30 =35+ 105 105 2 2 2 3 1 1 0 1 1 1 1 2 3 0 2.88

<20y <20y 114 117 2 2 1 2 2 1 1 1 1 1 0 1 3 0 3.67

30-<35 30-<35 102 103 2 1 1 2 2 2 0 1 1 2 1 1 0 3.31

<20y 25-<30 122 114 1 1 1 2 3 3 0 2 1 1 0 1 2 0 4.12

25-<30 30-<35 108 95 1 2 2 2 3 2 0 1 1 2 1 1 2 0 3.56

<20y 25-<30 100 98 2 2 2 2 1 2 1 1 0 0 2 1 1 3.45

<20y <20y 107 101 1 1 2 3 2 2 0 1 1 2 1 1 1 0 3.46

20+yr <20y 110 87 2 2 2 1 2 1 0 2 1 1 1 1 2 1 2.96

<20y 25-<30 110 2 2 1 2 3 2 1 1 1 2 1 1 2 1 2.97

<20y <20y 92 1 2 1 2 2 2 0 1 1 0 1 2 2 1 3.39

<20y <20y 111 101 2 1 2 2 2 0 2 1 2 1 1 2 1 3.82

<20y <20y 97 79 2 2 1 2 2 2 2 1 1 2 1 1 2 1 2.96

<20y <20y 94 84 2 1 1 2 3 1 2 2 1 1 0 1 1 1 3.46

25-<30 25-<30 106 92 2 2 1 2 1 2 0 1 1 2 0 1 2 0 3.17

<20y <20y 92 81 1 1 2 1 2 2 2 1 1 2 2 2.86

<20y 25-<30 107 103 2 2 2 2 1 2 2 1 1 1 0 1 2 1 3.61

25-<30 25-<30 103 100 2 1 1 2 2 2 0 1 1 2 1 1 2 0 4.83

20+yr 20+yr 115 1 2 2 2 2 1 1 1 2 2 0 1 2 0 3.84

<20y 30-<35 94 87 2 1 2 2 1 1 0 1 1 0 1 1 2 0 3.01

<20y <20y 112 110 1 1 1 2 3 3 0 1 1 2 0 1 2 1 3.71

30-<35 30-<35 117 78 2 2 1 3 0 2 1 2 1 1 2 0 4.04

20+yr <20y 101 100 2 2 2 2 2 2 0 1 1 1 1 1 2 0 3.16

30-<35 30-<35 120 102 2 2 1 2 3 3 0 1 1 2 1 1 2 1 3.92

25-<30 25-<30 91 88 2 2 1 1 1 1 0 1 2 0 0 2 2 3.355

25-<30 30-<35 125 112 2 1 1 2 2 2 0 1 1 1 0 1 2 0 3.29

20+yr 84 1 2 2 2 2 1 1 2 0 2 1 3.02

30-<35 =35+ 107 93 2 2 2 1 1 1 0 1 1 2 0 1 2 1 3.02

20+yr <20y 94 1 1 2 3 1 2 1 1 1 1 1 1 2 1 3.56

<20y <20y 102 88 2 2 2 3 2 1 0 2 1 2 1 1 3 1 3.75

<20y 25-<30 112 101 1 2 2 2 1 1 0 1 1 2 0 1 2 0 1.585

25-<30 30-<35 108 106 2 2 2 3 2 1 0 1 1 2 1 1 2 1 3.74

<20y <20y 105 97 1 2 3 1 1 2 1 1 0 1 3.08

<20y <20y 118 117 2 1 1 2 2 3 2 1 1 1 0 1 2 1 4.08

<20y 25-<30 96 2 2 2 1 0 1 1 2 1 3.9

30-<35 30-<35 105 80 2 1 1 3 1 0 1 1 0 1 2 2 0 2.95

<20y 25-<30 100 80 1 1 1 2 1 1 0 1 1 0 1 1 2 1 4.25

20+yr <20y 71 90 2 1 2 2 2 2 1 1 1 1 2.178

<20y 85 89 1 2 2 2 1 2 2 1 0 1 2.3

25-<30 25-<30 96 88 2 1 2 1 2 2 2 1 1 0 1 1 2 0 2.61

25-<30 25-<30 98 98 2 2 2 2 2 2 0 1 1 0 1 1 2 0 4.28

25-<30 25-<30 99 94 2 1 1 2 3 3 0 1 1 2 0 1 2 1 3.75

25-<30 30-<35 91 85 2 1 2 2 1 1 2 2 1 1 1 1 2 0 3.36

25-<30 30-<35 115 100 2 2 2 2 2 2 0 1 1 2 0 1 2 0 3.48

30-<35 30-<35 72 40 2 1 2 1 1 2 0 1 1 2 1 3.59

20+yr 25-<30 118 109 2 2 2 3 1 2 0 1 1 1 1 1 2 1 3.27

<20y <20y 92 87 2 2 2 1 2 1 2 2 1 0 0 1 2 2.99

<20y 30-<35 90 85 1 2 2 1 1 3 0 1 1 1 0 1 2 0 2.94

<20y 25-<30 113 1 1 3 3 2 1 1 1 1 1 1 2 0 3.05

30-<35 25-<30 118 2 1 1 2 2 2 0 1 1 2 0 1 0 3.79

=35+ =35+ 104 102 2 2 1 2 1 2 0 1 1 2 1 2 0 3.19

20+yr <20y 100 2 1 2 2 2 2 0 2 1 0 0 1 2 1 2.78

25-<30 25-<30 101 2 1 2 2 3 3 0 1 1 2 1 1 2 0 3

=35+ =35+ 103 95 2 1 2 1 1 2 0 1 1 2 1 1 3 1 3.01

<20y 25-<30 105 2 2 1 3 1 1 0 1 1 1 0 1 2 1 2.8

25-<30 30-<35 113 87 2 1 1 2 2 1 0 1 1 2 1 1 2 0 3.6

25-<30 25-<30 122 125 2 1 2 3 2 2 1 1 1 2 1 1 2 1 3.7

<20y 30-<35 104 90 2 2 2 1 1 1 0 1 1 1 0 1 2 1 3.92

25-<30 25-<30 115 108 1 2 2 2 3 0 2 1 2 1 1 3 1 2.98

25-<30 <20y 106 91 2 1 2 2 3 2 0 1 1 1 1 3.44

30-<35 =35+ 96 2 1 2 2 3 0 2 1 0 0 2 3.64

30-<35 =35+ 120 128 2 1 1 2 3 3 0 1 1 2 0 1 2 0 2.92

25-<30 =35+ 111 106 2 2 1 3 3 3 0 1 1 2 1 1 2 1 3.35

<20y 25-<30 101 95 2 2 1 2 2 1 0 1 1 1 0 1 2 0 3.72

25-<30 25-<30 100 97 2 1 2 2 2 3 2 2 1 1 0 1 2 3.88

25-<30 25-<30 100 78 2 2 2 1 2 2 0 1 1 0 1 2 2 1 2.435

25-<30 30-<35 94 114 1 2 2 3 3 2 0 1 1 2 1 1 2 0 3.67

<20y <20y 103 95 1 2 2 2 1 3 0 1 1 1 1 1 3 1 3.81

30-<35 30-<35 102 105 2 2 1 2 2 2 0 1 1 0 0 1 0 4.93

20+yr 86 2 2 1 1 0 1 1 0 1 1 2 1 2.95

<20y <20y 100 84 2 1 1 2 1 0 1 1 0 0 1 2 1 3.62

<20y <20y 103 101 2 1 1 2 3 2 0 1 1 2 0 1 2 0 3.49

25-<30 25-<30 117 108 2 1 2 2 2 3 0 1 1 1 1 1 2 1 3.94

25-<30 25-<30 102 90 2 2 2 1 2 1 2 1 1 1 1 1 0 3.28

30-<35 =35+ 111 99 1 2 1 2 2 1 0 1 1 2 1 1 2 1 3.187

25-<30 25-<30 98 96 2 2 2 2 3 1 0 2 1 1 0 1 2 0 3.69

<20y 25-<30 104 103 1 2 1 3 3 3 1 1 1 1 3 0 3.36

30-<35 30-<35 102 93 2 1 3 3 3 0 1 1 1 0 1 2 0 3.5

25-<30 25-<30 100 87 2 1 1 2 3 1 2 1 1 1 1 1 2 1 3.22

25-<30 25-<30 122 101 2 2 1 3 3 2 0 1 1 2 0 1 2 0 3.53

30-<35 30-<35 100 1 1 2 2 3 3 0 1 1 2 1 2 3 1 3.63

25-<30 =35+ 94 2 2 2 2 3 3 0 1 1 2 1 1 2 1 3.06

30-<35 =35+ 84 81 2 2 1 1 1 2 1 1 1 1 1 1 3.58

30-<35 30-<35 95 2 2 2 2 2 2 0 1 1 2 1 2 2 1 3.09

30-<35 30-<35 107 2 2 2 2 2 2 0 1 2 2 0 2 2 1 3.69

<20y 25-<30 122 110 1 1 1 2 2 2 0 1 1 2 0 1 2 0 3.41

25-<30 30-<35 108 76 2 1 2 2 2 2 1 1 1 2 1 1 2 1 2.535

=35+ =35+ 97 2 2 2 3 0 1 1 0 0 1 3 1 3.23

<20y 25-<30 90 101 1 1 2 2 3 2 0 1 1 0 1 1 2 1 2.74

=35+ =35+ 108 112 2 2 1 3 1 2 0 1 1 2 0 1 2 1 3.12

25-<30 30-<35 96 95 2 2 2 2 2 1 0 1 1 2 1 3 1 2.96

<20y 25-<30 102 86 2 2 2 2 3 3 1 1 1 1 0 1 3 1 2.89

25-<30 25-<30 92 88 1 2 2 2 2 0 2 1 0 0 1 2 1 3.15

20+yr <20y 110 99 1 2 2 3 2 1 1 2 1 2 1 3.675

<20y 30-<35 81 100 1 1 1 2 3 2 2 2 1 2 2 2.84

20+yr 20+yr 89 83 2 2 1 1 1 1 1 1 1 1 1 1 2 4.06

25-<30 25-<30 83 2 2 2 1 1 0 1 1 0 0 2 3 1 2.62

30-<35 30-<35 97 102 2 1 1 2 2 3 0 1 1 2 1 1 3 0 3.79

20+yr 20+yr 86 1 2 1 2 2 2 1 2 1 1 1 1 2 1 3.4

<20y 25-<30 84 79 1 2 1 2 2 1 1 1 3

<20y 25-<30 89 86 2 1 1 2 2 1 1 1 1 1 1 1 2 1 3.82

20+yr <20y 94 1 2 2 2 2 1 0 1 2 1 1 2 1 3.08

20+yr <20y 85 105 2 2 2 2 3 2 0 1 2 2 1 1 2 1 3.02

<20y <20y 114 100 2 1 1 1 2 1 0 1 1 1 1 1 2 1 3.45

25-<30 25-<30 115 94 2 2 2 2 2 3 2 1 1 2 0 2 3 0 3.37

25-<30 25-<30 89 94 2 1 2 1 0 1 1 1 0 1 2 1 3.94

<20y 25-<30 94 88 2 1 1 2 2 3 0 1 1 2 1 1 3 1 3.45

<20y 103 88 2 1 2 0 1 1 2 0 1 3 0 3.13

25-<30 30-<35 111 117 2 1 1 2 2 2 0 1 1 2 0 1 2 1 3.7

<20y 98 88 1 2 2 3 3 0 1 1 0 0 1 2 0 3.38

<20y 25-<30 120 1 2 1 1 3 2 1 1 1 2 1 1 2 0 3.49

=35+ =35+ 100 109 2 2 1 3 3 2 0 1 1 0 0 1 2 1 3.98

<20y <20y 103 94 2 1 2 2 1 1 2 2 1 1 1 1 2 1 2.54

30-<35 =35+ 110 117 1 2 2 3 1 2 0 2 1 2 1 1 2 1 3.88

<20y 25-<30 102 95 2 1 1 2 3 1 0 1 1 1 0 1 2 1 3.54

25-<30 25-<30 94 98 2 1 2 2 3 2 0 1 1 1 1 2 2 1 3.27

<20y <20y 102 1 1 1 2 2 1 2 2 1 1 1 1 1 1 3.39

<20y <20y 78 100 1 2 1 2 2 1 0 1 1 0 2 0 4.06

<20y 100 1 2 1 2 2 0 1 1 1 1 1 2 1 3.39

=35+ =35+ 114 2 2 1 1 1 2 0 1 1 2 0 1 3 0 3.85

<20y <20y 88 81 2 2 2 2 2 1 0 1 1 1 0 2 1 1 3.48

25-<30 30-<35 104 104 2 1 1 3 3 2 2 1 1 1 1 1 3 0 3.73

<20y =35+ 103 112 2 2 2 2 2 2 0 2 1 1 0 1 2 4.15

30-<35 30-<35 104 107 2 2 1 2 2 1 2 1 1 1 0 1 0 3.25

<20y 25-<30 114 96 2 2 2 2 2 2 0 1 1 1 1 1 2 0 3.25

25-<30 25-<30 122 117 2 1 1 2 2 3 0 1 1 2 1 1 3 0 3.71

25-<30 30-<35 95 91 2 1 1 1 2 0 1 1 2 1 1 2 0 3.47

<20y <20y 104 98 2 2 1 2 3 2 0 1 1 2 0 1 2 1 2.8

<20y 25-<30 105 92 2 2 2 2 3 3 0 2 1 2 1 1 2 0 4.43

<20y 25-<30 115 97 2 1 2 3 3 3 0 1 1 2 1 1 0 4.08

30-<35 30-<35 104 105 2 1 1 2 2 2 0 1 1 1 0 2 2 0 3.54

=35+ =35+ 105 89 1 2 1 3 3 2 0 1 1 2 1 1 3 0 4.82

20+yr 20+yr 105 108 1 2 2 2 2 2 0 1 2 2 1 1 2 1 3.5

<20y 25-<30 102 2 2 2 2 3 2 0 1 1 1 1 1 2 0 3.51

<20y 25-<30 97 98 1 1 1 2 1 1 2 1 1 0 1 2 2 0 2.87

20+yr 20+yr 122 91 1 1 1 1 2 3 1 1 1 1 1 1 2 1 3.24

<20y <20y 84 84 1 2 1 1 1 2 2 2 1 0 1 2.98

25-<30 30-<35 97 91 1 1 2 2 1 3 2 2 2 1 1 1 2 1 2.81

25-<30 25-<30 84 79 2 1 1 1 2 2 1 2 1 0 1 1 2 1 3.87

20+yr <20y 111 99 2 2 2 2 1 1 1 1 1 1 3.24

<20y 25-<30 108 1 1 1 2 2 2 1 1 1 1 1 1 2 0 3.35

<20y <20y 85 80 2 2 2 1 1 1 0 1 1 0 1 1 2 0 3.4

25-<30 25-<30 110 100 2 2 2 3 3 2 0 1 1 0 0 1 2 0 3.72

30-<35 30-<35 99 2 2 1 2 2 1 0 1 1 1 1 3 1 3.24

25-<30 25-<30 117 94 2 1 1 2 2 1 2 1 1 0 1 2 2 0 2.34

25-<30 25-<30 94 100 1 2 2 2 2 1 2 2 2 1 1 1 2.89

<20y 25-<30 88 93 2 2 1 2 2 1 0 1 1 0 1 2 2 0 4.32

25-<30 30-<35 107 95 2 2 1 2 2 3 0 1 1 1 1 1 2 0 3.64

<20y 25-<30 114 98 2 1 2 2 2 1 2 1 1 2 1 1 2 0 3.5

<20y <20y 99 91 1 2 2 3 3 3 2 2 1 2 1 1 2 0 3.32

<20y 30-<35 94 82 2 2 2 1 2 1 2 2 1 0 0 1 0 3.5

30-<35 30-<35 104 117 2 1 1 3 3 2 0 1 1 2 1 1 2 0 3.58

25-<30 30-<35 113 110 2 2 2 3 3 1 1 1 1 2 1 1 2 1 3.68

20+yr 20+yr 106 101 1 2 2 2 2 2 0 1 1 2 0 1 3 0 2.96

<20y 25-<30 106 95 2 2 2 3 3 2 0 2 1 0 1 1 2 0 3.64

25-<30 30-<35 94 80 2 1 2 3 3 0 1 1 2 1 1 3 1 3.65

<20y 111 98 1 2 1 2 0 1 1 2 1 1 2 0 2.75

<20y 25-<30 115 104 2 1 1 3 3 2 0 1 1 2 0 1 2 1 2.46

25-<30 25-<30 108 97 1 2 1 3 2 1 2 1 1 2 1 1 2 1 3.46

<20y 103 101 1 2 1 2 1 3 0 1 1 2 1 1 2 1 2.93

20+yr <20y 108 98 2 2 2 3 2 0 1 1 2 0 1 1 1 3.18

<20y 25-<30 100 89 2 2 2 3 3 1 0 1 1 1 1 1 2 1 3.96

25-<30 25-<30 96 96 2 2 1 2 2 2 0 2 1 2 1 1 2 0 3.12

<20y <20y 80 81 1 1 2 2 2 1 0 1 1 2 1 2.92

20+yr <20y 85 94 2 2 1 1 3 2 2 2 1 0 0 1 1 2.81

<20y <20y 108 1 2 2 2 2 1 1 1 1 1 1 1 2 1 2.89

20+yr <20y 107 1 2 2 1 2 1 1 1 1 1 1 1 2 1 2.63

30-<35 30-<35 101 94 2 1 2 2 2 1 0 1 1 2 0 1 2 0 3.38

<20y 25-<30 115 106 2 1 2 2 2 1 2 1 1 0 0 1 2 0 2.89

30-<35 30-<35 97 112 1 2 2 2 3 1 1 1 1 2 1 1 3 1 3.38

30-<35 =35+ 115 93 2 1 1 1 2 3 0 1 1 1 0 2 2 0 3.99

20+yr 30-<35 110 101 1 2 1 2 2 2 0 1 1 2 1 2 2 0 3.78

<20y 25-<30 97 100 2 2 2 2 2 2 0 1 1 2 1 1 1 1 3.38

20+yr <20y 103 95 1 2 2 2 2 1 1 1 2 1 1 1 2.72

<20y <20y 100 95 1 2 2 2 2 3 1 1 1 2 1 1 1 1 2.61

25-<30 25-<30 108 104 2 1 1 2 3 3 0 1 1 2 1 1 2 0 3.7

25-<30 25-<30 106 104 2 1 2 2 3 3 2 1 1 1 1 1 2 0 3.53

<20y 30-<35 102 95 2 2 1 2 3 1 2 1 1 1 1 1 4.17

20+yr 113 105 1 2 2 2 3 0 2 1 2 0 1 2 0 3.735

20+yr 98 111 1 1 2 2 1 2 2 1 2 0 2 2 1 2.29

30-<35 30-<35 114 112 2 1 1 2 2 3 0 2 1 1 1 1 2 0 2.84

25-<30 =35+ 104 104 2 1 1 3 3 3 1 2 1 1 1 1 2 0 3.3

<20y <20y 111 2 1 2 2 2 1 2 1 1 2.62

<20y 20+yr 99 2 2 1 2 2 3 0 1 1 1 2 3.71

25-<30 25-<30 102 98 2 2 1 2 2 3 2 1 1 1 1 1 0 4.49

<20y <20y 101 87 1 2 1 2 2 2 2 1 2 1 1 2 1 4.04

30-<35 =35+ 85 90 1 1 1 1 2 1 2 2 1 1 1 2 0 3.43

<20y 25-<30 102 88 2 1 1 2 2 1 0 2 1 2 0 1 2 1 3.39

<20y <20y 108 101 1 1 1 2 1 3 2 1 1 1 1 1 2 1 4.03

25-<30 25-<30 106 95 1 1 1 2 2 2 0 1 1 2 1 4.16

30-<35 25-<30 108 128 2 2 1 2 2 1 1 2 1 2 1 1 2 1 3.08

<20y <20y 123 112 2 1 2 3 2 3 0 1 1 1 1 1 2 1 3.16

25-<30 25-<30 104 1 1 2 2 2 1 0 1 1 0 0 1 3 0 3.9

25-<30 30-<35 102 104 2 2 2 2 3 2 0 1 1 0 1 1 0 3.625

30-<35 30-<35 115 86 1 2 2 2 3 2 0 1 1 2 0 1 1 3.12

25-<30 <20y 109 95 1 2 1 2 3 2 0 1 1 1 1 2 0 3.67

30-<35 =35+ 115 91 2 2 1 1 1 1 2 1 1 1 1 1 1 3.76

25-<30 30-<35 105 94 2 1 1 2 2 2 2 1 1 2 1 1 2 1 4.12

25-<30 30-<35 120 117 2 1 1 2 2 2 1 1 1 2 1 1 2 0 3.94

20+yr 99 86 2 2 1 1 2 1 0 1 1 1 0 2 2 1 3.36

25-<30 =35+ 102 105 2 1 1 1 1 1 0 1 1 0 1 1 2 1 3.07

25-<30 25-<30 117 110 1 1 1 2 2 1 0 2 1 1 1 1 2 0 3.9

<20y <20y 105 88 2 1 2 2 2 2 0 2 1 1 1 1 2 0 3.07

25-<30 25-<30 108 89 2 2 2 2 2 2 1 2 1 0 1 1 2 0 2.52

30-<35 30-<35 96 89 2 2 1 2 1 3 0 2 1 1 0 1 2 1 3.76

<20y <20y 99 104 2 1 1 2 2 2 1 1 1 1 0 2 2 1 4.04

30-<35 <20y 105 91 2 2 2 2 2 1 2 2 2 1 1 1 2 1 3.51

<20y <20y 95 88 2 2 1 2 2 1 0 1 1 2 0 1 0 3.55

30-<35 =35+ 99 99 2 2 2 2 1 1 0 1 1 1 0 2 2 0 3.41

25-<30 30-<35 108 100 2 1 1 2 3 2 1 2 1 1 2 0 3.64

<20y <20y 120 94 2 1 1 2 2 2 0 1 1 1 1 1 0 3.4

<20y <20y 107 95 2 2 1 2 2 1 0 1 1 2 1 1 2 0 3.09

25-<30 25-<30 99 1 1 1 3 3 2 0 1 1 0 1 1 2 0 3.71

25-<30 25-<30 89 87 2 1 2 1 2 0 2 1 0 0 1 0 3.55

25-<30 25-<30 99 108 1 1 1 3 3 3 0 1 1 2 1 1 2 0 4.64

20+yr 20+yr 104 100 2 2 3 2 1 0 1 1 1 1 3.65

20+yr <20y 81 86 2 2 1 1 2 1 0 1 1 1 0 2 2 1 3.82

30-<35 30-<35 103 1 2 1 1 1 3 0 1 1 1 0 1 3 1 3.06

20+yr 20+yr 111 1 2 1 2 2 1 1 2 1 1 1 3.74

25-<30 25-<30 97 110 2 1 2 2 2 1 2 1 1 1 1 1 2 0 2.77

<20y 25-<30 99 2 1 2 2 2 3 0 1 1 2 1 1 2 1 3.08

<20y 25-<30 110 101 2 1 1 3 3 3 0 1 1 2 1 1 3 0 3.06

<20y 25-<30 77 83 1 1 2 2 2 1 0 1 1 0 1 1 2 1 2.34

20+yr 20+yr 115 101 2 2 1 2 2 3 1 1 1 4.02

<20y 25-<30 97 84 1 1 1 2 2 1 2 1 1 1 1 0 2.85

20+yr 117 1 1 1 0 1 1 0 1 3

25-<30 30-<35 107 66 2 2 1 2 3 2 1 1 1 1 0 1 2 1 4.31

<20y 25-<30 86 91 2 2 1 2 2 2 2 1 1 1 1 2 0 3.22

<20y 25-<30 101 94 1 1 1 3 3 2 0 2 1 1 1 1 2 1 3.71

30-<35 30-<35 103 1 1 1 1 1 2 2 1 1 0 3 1 2.79

20+yr 25-<30 102 87 2 2 2 2 2 1 2 1 1 0 2 2 1 3.88

<20y <20y 100 81 2 2 2 2 2 3 1 1 1 1 0 1 1 3.26

25-<30 25-<30 92 88 2 2 2 1 2 2 0 1 1 0 0 1 2 0 2.76

<20y 25-<30 99 100 2 1 1 2 3 2 0 2 1 2 0 1 1 0 3.39

=35+ =35+ 109 112 1 1 1 3 3 3 0 1 1 2 1 3 1 4.32

<20y <20y 78 1 1 2 2 1 1 2 1 1 1 1 1 1 1 3.64

<20y 25-<30 90 85 1 2 1 2 2 1 2 1 1 0 0 2 2 1 4.42

25-<30 25-<30 100 101 2 1 1 3 2 2 1 1 1 2 0 1 2 0 3.1

<20y <20y 118 1 2 2 2 3 0 1 1 1 1 1 2 1 2.48

25-<30 25-<30 112 110 2 1 2 2 2 2 0 2 1 2 1 2 2 1 3.35

<20y 25-<30 110 110 2 2 1 2 2 3 0 1 1 1 1 1 2 1 3.41

25-<30 25-<30 122 106 2 1 2 1 3 3 0 1 1 2 0 1 3 0 1.96

<20y 25-<30 104 93 1 1 2 2 2 2 0 1 1 1 1 2 1 3.295

<20y <20y 105 88 2 2 2 2 3 1 1 1 1 1 1 1 0 3.68

20+yr 20+yr 102 88 1 1 1 2 2 2 1 1 2 1 0 1 2 1 3.4

20+yr <20y 100 94 2 1 1 2 1 1 1 1 1 1 2 1 3.16

20+yr <20y 107 2 2 2 2 1 0 1 1 2 1 3.43

20+yr <20y 108 109 1 2 1 2 2 1 0 1 2 1 1 1.48

<20y <20y 118 101 2 2 2 2 3 3 0 1 1 2 0 1 2 0 3.36

<20y 25-<30 89 91 1 2 2 2 1 1 2 1 1 0 1 3.04

20+yr <20y 109 107 1 2 1 2 2 1 1 1 1 1 1 1 3.09

30-<35 100 1 1 1 1 1 1 0 1 2 1 1 3.5

20+yr 20+yr 123 2 1 2 2 1 0 2 1 1 0 1 2 0 2.89

<20y 25-<30 105 103 2 2 2 2 2 3 2 1 1 1 0 1 3 0 3.11

<20y <20y 112 96 1 2 1 3 2 1 0 1 1 1 1 1 2 0 2.23

<20y <20y 113 88 2 1 1 3 3 1 0 1 1 2 0 1 2 1 3.83

<20y 25-<30 89 82 1 1 1 2 2 2 0 1 2 1 1 0 3.42

<20y 30-<35 118 2 2 1 2 2 1 0 2 1 0 1 1 4.01

30-<35 25-<30 103 2 1 2 2 2 1 0 1 1 2 1 2 1 0 3.51

30-<35 =35+ 120 89 2 2 1 2 2 2 2 1 1 2 1 1 2 1 3.63

25-<30 25-<30 89 106 2 1 2 1 1 2 1 1 1 1 0 1 1 1 2.72

30-<35 30-<35 123 103 2 2 1 2 2 2 0 1 1 2 0 1 2 1 3.95

<20y 25-<30 102 2 1 1 2 2 2 1 1 1 0 1 1 1 1 3.3

25-<30 25-<30 81 2 2 2 2 2 1 2 2 1 1 2 0 3.91

25-<30 25-<30 112 114 2 2 2 2 1 3 0 1 1 2 0 1 2 1 3.57

30-<35 30-<35 108 100 2 2 2 1 3 0 1 1 2 1 1 3.19

25-<30 <20y 100 89 1 1 2 2 2 2 0 1 1 1 1 2 0 3.51

20+yr 25-<30 109 92 2 2 1 2 1 1 0 1 1 2 1 3

<20y <20y 110 2 2 1 1 2 1 1 1 1 1 1 1 2 0 3.11

<20y <20y 89 1 1 2 2 1 1 0 1 1 1 1 1 2 1 3.38

<20y =35+ 94 101 1 1 2 2 2 2 0 1 1 0 0 1 3 0 3.43

25-<30 30-<35 118 77 2 1 2 3 2 2 0 1 1 2 0 1 2 1 2.68

25-<30 25-<30 83 93 2 1 3 2 2 0 1 2 1 1 2 1 3.92

<20y 25-<30 104 2 2 2 2 2 2 0 1 1 1 1 1 2 0 4.33

30-<35 =35+ 108 2 2 1 2 1 1 0 1 1 2 1 3.93

25-<30 30-<35 118 2 1 1 3 3 2 0 2 1 2 0 1 0 3.935

30-<35 30-<35 97 81 2 2 1 2 2 1 0 1 1 0 1 1 2 1 2.93

25-<30 25-<30 101 88 1 1 2 3 2 2 0 1 1 2 1 3.45

<20y 25-<30 105 2 2 2 2 2 3 0 1 1 1 1 1 3 1 3.32

<20y 25-<30 114 98 2 2 2 2 2 3 0 2 1 1 1 1 1 0 3.86

<20y 25-<30 105 117 2 2 1 2 2 2 0 2 1 1 0 1 2 1 3.42

30-<35 25-<30 107 102 2 1 1 2 2 3 0 2 1 2 0 1 0 2.41

<20y <20y 127 112 2 2 1 3 3 3 0 1 1 2 1 2 3 1 3.03

25-<30 25-<30 95 91 2 2 2 2 2 1 0 1 1 2 1 2 2 1 3.68

<20y =35+ 92 1 2 2 2 1 1 2 1 1 0 0 1 2 1 4.68

<20y 109 100 2 2 1 2 2 2 0 1 1 2 1 1 2 0 3.29

<20y <20y 96 1 1 1 2 2 1 2 1 1 0 2 3.08

25-<30 <20y 97 76 2 1 2 2 2 0 1 1 0 0 1 3 0 3.85

30-<35 30-<35 97 93 1 2 1 3 3 1 0 1 1 1 1 1 3 1 2.38

<20y 25-<30 108 1 1 2 3 2 1 0 1 1 1 0 1 2 0 4.56

25-<30 30-<35 99 103 1 1 2 2 1 2 2 2 1 2 1 1 2 1 3.7

<20y 25-<30 100 100 2 1 1 3 3 2 0 1 1 2 0 1 2 1 3.21

<20y <20y 89 101 2 1 2 1 1 1 2 1 1 0 1 1 2 0 3.6

25-<30 =35+ 101 105 2 2 3 3 3 0 1 1 2 0 1 2 1 3.89

25-<30 25-<30 97 94 2 2 2 2 1 2 0 2 1 1 1 3.25

25-<30 25-<30 90 106 2 1 1 2 2 1 0 1 1 2 1 1 3 0 3.14

<20y 25-<30 99 81 2 1 2 1 2 1 0 2 1 1 0 1 3 1 3.19

<20y 25-<30 108 97 1 1 2 2 3 3 2 2 1 0 1 1 3 0 3.48

<20y 30-<35 91 106 1 2 2 1 2 3 0 2 1 2 1 2 2 1 3.2

<20y <20y 98 94 2 2 1 1 1 2 2 1 1 0 0 1 2 0 2.66

30-<35 25-<30 117 95 1 2 2 2 2 2 2 2 1 1 1 1 2 1 2.79

<20y 25-<30 123 2 1 2 2 3 3 0 1 1 2 1 1 3.85

<20y <20y 103 97 2 2 2 3 2 1 0 1 1 2 1 1 3 1 3.22

25-<30 <20y 104 94 1 1 2 1 3 0 2 1 1 0 1 3 0 2.73

<20y 25-<30 98 100 2 1 1 2 1 1 0 1 1 1 1 1 2 0 3.1

25-<30 =35+ 111 103 2 1 2 2 1 2 0 1 1 1 1 1 2 1 3.11

20+yr <20y 92 99 1 1 2 2 1 1 2 1 1 0 1 1 2 1 2.76

30-<35 =35+ 93 93 2 2 2 2 2 2 0 1 1 1 0 1 3 0 3.49

25-<30 =35+ 100 112 1 1 2 2 2 1 0 2 1 2 1 1 0 3.05

<20y 25-<30 105 103 2 1 2 2 2 3 0 1 1 1 1 1 3 1 2.91

25-<30 25-<30 101 112 2 1 1 3 3 2 1 1 1 1 0 1 3 0 3.45

=35+ =35+ 111 128 2 1 2 3 3 3 0 1 1 2 0 1 2 1 3.59

20+yr <20y 103 1 2 1 1 3 1 0 1 1 1 1 1 2 1 4.64

20+yr <20y 97 90 2 1 1 2 2 2 0 1 1 1 0 1 2 1 3.51

25-<30 30-<35 126 98 2 1 1 2 2 3 0 1 1 2 1 2 2 1 3.1

20+yr 20+yr 89 105 1 2 1 3 2 2 0 1 1 1 0 1 2 0 3.55

25-<30 25-<30 93 94 2 1 1 2 2 2 0 1 1 2 0 1 2 1 2.28

<20y <20y 98 92 1 1 1 1 2 1 1 1 1 1 1 3.97

30-<35 =35+ 75 93 1 2 2 3 3 3 0 1 1 1 0 1 2 0 2.76

<20y 30-<35 105 101 2 1 1 2 2 3 0 1 1 0 0 1 2 0 3.09

=35+ =35+ 106 98 1 2 1 2 3 3 0 1 1 2 0 1 1 3.34

<20y <20y 127 88 1 1 1 2 2 3 0 1 1 2 0 1 2 0 3.99

30-<35 30-<35 109 100 2 2 2 2 2 2 0 1 1 2 0 1 3 1 3.51

<20y <20y 102 97 2 2 1 1 1 1 2 1 1 1 1 1 2 1 3.66

<20y 25-<30 114 89 1 1 2 2 2 1 0 1 1 1 1 3.45

20+yr <20y 102 2 1 2 3 2 1 1 1 1 1 0 1 2 1 3.19

20+yr <20y 96 97 1 1 2 2 2 2 0 1 1 1 0 1 2 0 3.4

<20y 103 96 1 2 2 1 1 2 1 1 1 0 1 2 1 3.48

<20y <20y 104 2 1 2 1 1 1 0 1 1 2 0 1 2 0 3.42

20+yr <20y 91 1 1 1 2 1 1 1 2 1 2 1 1 1 0 3.29

30-<35 30-<35 97 97 2 2 2 2 1 2 0 1 1 2 1 1 3 1 3.57

<20y 30-<35 108 1 1 2 2 1 2 2 1 1 0 1 1 2 1 2.41

<20y <20y 105 98 2 1 2 2 2 1 0 1 1 2 0 1 3 1 2.99

20+yr <20y 97 97 2 1 2 2 2 1 0 1 1 1 0 1 2 1 3.26

<20y 25-<30 102 88 2 2 2 2 2 1 0 1 1 0 0 1 3 0 2.1

30-<35 30-<35 103 94 1 1 2 2 2 1 0 2 1 0 1 1 2 0 3.26

20+yr 84 85 1 2 2 2 1 1 0 1 1 0 0 2 2 1 3.44

<20y 25-<30 84 85 1 1 1 2 2 1 0 2 1 1 0 1 1 2.85

=35+ =35+ 95 93 2 1 2 2 1 2 1 1 0 1 3.67

20+yr 20+yr 87 1 2 2 2 1 1 2 2 1 1 3 1 2.74

30-<35 30-<35 110 83 2 2 1 2 1 2 0 1 1 0 0 1 0 3.9

25-<30 30-<35 114 119 2 1 1 2 3 3 0 1 1 2 0 1 3 0 3.37

<20y 25-<30 115 88 2 2 1 1 2 3 0 1 1 2 1 1 2 0 3.25

<20y <20y 109 90 1 1 1 2 2 2 0 2 1 2 0 1 2 0 4.48

<20y 25-<30 102 95 2 2 2 2 3 3 0 2 1 2 0 1 0 3.84

<20y 20+yr 107 122 1 2 1 2 3 2 0 1 1 2 0 1 2 0 3.915

<20y 25-<30 117 2 1 2 1 3 0 1 1 2 0 1 2 1 3.81

<20y <20y 92 97 2 2 1 2 2 3 0 2 1 1 1 1 2 0 3.98

<20y 25-<30 100 81 2 1 2 1 1 1 2 1 1 0 0 1 3 1 3.6

<20y <20y 106 108 2 2 2 2 2 3 0 1 1 1 0 1 3 0 3.02

25-<30 25-<30 107 106 1 1 2 2 2 2 2 1 1 1 2 2 0 4.46

25-<30 30-<35 115 80 2 2 1 2 3 3 0 1 1 1 0 1 2 1 4.74

<20y <20y 115 1 2 1 2 2 3 1 1 1 0 0 1 2 1 3.7

30-<35 =35+ 109 94 2 1 2 2 2 2 0 1 1 2 1 1 0 4.12

<20y <20y 107 96 1 1 1 2 2 1 0 2 1 2 1 4.07

25-<30 25-<30 97 101 2 1 1 2 3 2 0 1 1 2 0 2 2 0 3.71

25-<30 30-<35 107 104 2 1 2 2 1 3 0 1 1 2 0 1 2 0 3.58

30-<35 =35+ 105 95 1 2 2 1 3 3 0 2 1 1 1 2 3.78

30-<35 =35+ 97 109 2 1 1 2 1 2 0 1 1 1 0 1 2 1 4.33

30-<35 30-<35 103 98 2 1 2 1 1 1 1 1 1 2 1 1 2 0 2.97

<20y <20y 101 1 1 2 2 2 1 2 2 1 0 0 2 2 1 2.91

20+yr <20y 100 1 2 2 1 2 2 1 1 2 1 1 1 1 3.61

20+yr 20+yr 100 1 1 2 2 2 0 1 1 0 1 1 2 1 3.37

30-<35 =35+ 105 105 2 1 1 2 2 1 0 2 1 2 0 1 2 1 3.69

<20y 25-<30 108 97 2 1 1 2 2 2 0 1 1 0 0 2 2 1 3.61

<20y <20y 89 96 1 1 2 2 2 0 1 1 1 1 1 2 1 3.55

20+yr 101 1 2 1 2 2 1 0 1 1 0 1 1 2 0 3.28

20+yr <20y 110 114 1 2 1 1 2 3 1 1 1 0 2 2 0 4.1

<20y 25-<30 118 101 2 2 2 2 2 2 0 2 1 1 1 1 2 0 3.53

<20y 25-<30 114 129 2 1 2 3 2 2 0 1 1 2 0 1 2 0 2.82

<20y 25-<30 111 88 2 2 1 2 3 3 0 2 1 2 0 1 2 3.11

25-<30 25-<30 92 119 2 1 2 2 2 2 0 2 1 2 0 1 1 0 2.47

<20y <20y 88 1 2 2 3 3 0 1 1 1 0 1 2 1 2.99

30-<35 25-<30 98 97 2 1 1 2 3 2 2 2 1 1 0 1 4.34

<20y 25-<30 101 108 2 1 2 3 1 2 0 1 1 2 1 1 2 0 3.34

<20y 25-<30 107 94 2 1 2 3 3 2 0 1 1 2 0 1 3 1 3.19

=35+ 30-<35 122 117 2 2 2 3 3 3 0 1 1 2 0 2 3 0 3.22

20+yr <20y 112 2 2 1 2 3 3 0 1 1 1 0 1 2 1 4.19

20+yr <20y 112 2 2 1 2 3 0 1 1 2 0 1 2 0 3.36

30-<35 30-<35 113 93 2 1 2 1 2 2 0 1 1 2 0 1 1 3.505

20+yr <20y 112 1 1 1 2 1 1 1 1 1 1 2 1 3.16

<20y 30-<35 113 96 2 1 1 2 2 2 0 1 1 1 1 2 2 1 3.99

25-<30 30-<35 114 98 2 1 2 3 3 3 0 2 1 2 0 1 2 0 3.34

20+yr <20y 93 98 2 2 1 1 2 2 1 1 1 2 1 1 3 0 3.4

<20y 25-<30 100 2 2 2 1 2 1 2 2 1 0 2 1 2.89

<20y <20y 110 98 2 2 1 2 1 2 2 1 1 2 1 1 2 1 3.27

<20y <20y 102 106 2 1 1 2 2 3 0 1 1 2 0 1 2 1 3.24

20+yr <20y 83 89 2 2 1 2 2 1 1 1 1 0 0 1 1 1 2.56

<20y <20y 96 97 2 2 2 2 2 1 0 1 1 1 0 1 2 0 4.075

25-<30 25-<30 97 89 1 3 2 2 0 2 1 1 2 3.25

<20y 25-<30 114 92 2 1 1 2 3 1 1 1 1 2 1 3.46

25-<30 <20y 91 90 2 1 2 2 2 2 0 1 1 0 2 1 3.57

30-<35 30-<35 117 97 2 2 2 0 1 1 2 0 1 0 3.19

20+yr <20y 105 94 2 2 2 2 2 1 0 1 1 1 1 2.99

25-<30 30-<35 102 1 1 1 1 3 2 0 1 1 2 0 1 3 1 3.68

<20y 30-<35 104 91 2 1 1 1 2 3 0 1 1 1 0 1 2 1 3.5

<20y 25-<30 113 101 2 1 2 1 2 1 0 1 1 1 1 1 2 0 3.45

25-<30 30-<35 101 1 1 1 1 1 0 2 1 2 0 1 3 0 3.79

=35+ =35+ 89 92 2 2 1 2 3 2 0 1 1 2 1 2 2 0 3.27

=35+ 25-<30 101 95 2 1 2 2 2 1 1 1 2 0 1 2 0 3.08

25-<30 =35+ 107 82 2 1 3 3 1 0 1 1 2 2 1 1 3.23

30-<35 30-<35 115 109 2 1 1 2 2 3 2 1 1 1 0 1 2 0 3.76

30-<35 =35+ 91 88 1 2 2 2 2 2 0 2 1 1 0 3.26

<20y =35+ 98 2 1 2 2 1 2 2 2 1 1 0 1 0 3.34

<20y 25-<30 117 97 2 1 2 3 1 2 0 1 1 2 0 1 2 0 3.69

20+yr 20+yr 103 93 1 2 1 2 2 3 1 1 1 1 1 2 1 1.98

20+yr 122 92 2 2 1 2 2 0 1 1 2 1 1 2 0 3.18

=35+ =35+ 104 98 2 1 1 1 0 1 1 2 0 1 0 3.53

=35+ =35+ 106 107 2 1 3 1 2 2 2 1 0 1 1 0 3.91

25-<30 =35+ 114 2 2 2 2 2 1 1 1 1 2 0 1 0 2.48

<20y 25-<30 83 93 2 2 1 2 2 2 2 2 1 0 0 2 3.5

25-<30 30-<35 106 97 2 1 2 2 3 0 2 1 2 1 3.57

20+yr <20y 91 85 2 2 2 2 2 1 2 2 2 1 1 3 1 3.2

=35+ 30-<35 97 77 2 1 2 1 2 1 0 1 1 1 0 1 2 1 3.57

25-<30 25-<30 98 90 1 1 2 2 3 2 2 1 1 0 1 2 1 1 3.43

20+yr <20y 103 105 1 1 1 2 1 1 0 1 1 1 0 1 3 1 3.52

30-<35 =35+ 112 93 2 2 1 2 2 1 0 1 1 0 0 1 2 0 3.95

<20y <20y 95 2 1 2 2 2 1 1 1 1 1 0 1 2 0 3.15

25-<30 30-<35 91 88 2 2 2 2 3 1 0 1 2 0 0 1 1 0 3.7

25-<30 107 1 2 1 3 3 1 0 1 2 2 1 2.8

30-<35 25-<30 97 93 2 1 2 2 2 2 0 1 1 2 0 1 2 0 4.15

<20y 106 97 1 2 1 2 2 0 2 1 1 0 1 3 0 3.78

<20y <20y 114 1 1 2 2 3 1 0 2 1 1 0 2 1 0 2.938

<20y <20y 103 106 1 1 3 3 3 0 1 1 1 0 1 1 0 3.81

20+yr <20y 108 109 2 2 1 2 3 3 0 1 1 2 0 1 2 0 2.93

<20y 25-<30 105 103 2 2 2 2 2 1 2 1 2 2 0 1 2 0 3.47

20+yr 20+yr 95 96 2 2 2 2 2 1 1 1 1 0 0 1 2 1 2.85

25-<30 30-<35 115 2 1 2 3 3 3 2 1 1 1 0 1 3 1 2.95

25-<30 30-<35 110 96 2 1 2 3 3 2 0 1 1 1 0 2 2 0 3.08

=35+ =35+ 117 93 2 1 2 2 2 0 1 1 2 1 1 2 0 3.59

<20y 25-<30 106 104 1 1 1 2 2 1 0 1 1 1 0 1 2 0 4.21

<20y <20y 98 88 2 1 1 2 2 2 1 1 1 0 0 1 2 0 2.53

25-<30 25-<30 115 98 2 1 1 2 2 3 0 1 1 2 1 1 1 2.6

<20y 25-<30 85 87 1 1 2 1 1 1 2 1 0 0 1 0 3.13

30-<35 =35+ 105 102 2 1 1 1 2 2 0 1 1 1 0 1 2 1 3.49

<20y 25-<30 117 2 1 2 2 2 2 0 1 1 1 0 2 2 0 3.115

<20y <20y 108 89 2 1 2 2 2 1 2 1 1 2 0 1 2 0 4.06

25-<30 25-<30 88 98 2 1 2 2 1 0 1 1 1 1 1 3 1 3.52

20+yr <20y 125 1 2 2 2 2 1 0 1 1 1 1 3.14

=35+ =35+ 103 2 1 2 3 3 2 2 1 1 1 0 2 3 0 3.83

25-<30 25-<30 110 95 2 1 2 1 2 1 0 1 1 2 0 1 2 0 3.53

25-<30 30-<35 135 117 2 2 1 2 2 3 0 1 1 2 0 2 2 0 3.02

20+yr <20y 100 97 2 2 1 2 3 1 1 1 1 1 0 2 2 0 3.59

25-<30 25-<30 103 95 2 1 2 2 2 3 0 1 1 2 1 1 0 2.63

20+yr <20y 92 101 2 2 1 1 1 1 0 1 1 0 1 1 2 0 2.65

25-<30 107 108 1 1 2 2 1 0 1 1 0 1 3.15

25-<30 25-<30 123 97 2 2 1 2 2 3 1 1 1 1 0 1 3 1 3.78

20+yr <20y 86 1 2 2 2 3 1 0 1 1 1 0 1 2 1 3.625

25-<30 =35+ 115 89 2 1 2 2 1 2 0 1 1 2 0 1 2 1 3.37

<20y 25-<30 104 1 1 2 2 3 0 1 1 1 0 1 3 1 2.97

<20y <20y 92 80 1 1 2 2 2 1 0 1 1 0 0 2 2 1 3.19

<20y 25-<30 115 106 2 1 1 2 3 2 0 1 1 1 0 1 3 0 3.8

25-<30 30-<35 102 2 2 2 1 2 1 1 1 1 2 0 1 2 0 2.99

<20y 25-<30 91 88 2 1 1 2 2 2 0 1 1 1 0 1 2 1 3.23

25-<30 25-<30 96 93 2 1 2 2 1 2 1 1 1 1 2 1 3.38

25-<30 =35+ 114 103 1 1 2 2 3 2 0 2 1 2 0 1 2 0 3.09

<20y 25-<30 117 110 2 2 2 3 2 3 0 1 1 1 0 1 3 1 3.4

<20y 25-<30 102 110 2 1 2 3 2 2 0 1 1 1 0 1 2 0 3.91

20+yr <20y 103 105 2 2 1 3 3 1 1 1 1 1 0 1 1 3.41

<20y =35+ 110 2 1 2 2 1 2 1 1 3.49

=35+ =35+ 103 109 2 2 2 3 1 2 0 1 1 2 0 2 3 1 3.74

25-<30 30-<35 106 86 2 1 2 1 3 2 2 2 1 1 1 2 1 3.15

20+yr 99 2 2 2 1 2 2 1 0 0 1 2 1 3.64

<20y <20y 94 97 2 1 2 2 2 2 0 1 1 2 0 1 0 3.59

=35+ =35+ 115 75 2 1 1 1 1 1 0 1 1 2 0 2 1 1 3.17

25-<30 <20y 110 95 2 1 2 1 2 0 1 1 2 0 1 2 1 3.06

<20y <20y 92 91 2 2 2 2 2 1 0 2 1 1 0 1 1 0 3.46

<20y <20y 104 1 2 2 2 1 2 1 1 1 2 0 1 2 0 3.73

<20y 25-<30 114 1 2 1 3 3 3 0 1 1 0 2 3.93

20+yr 30-<35 113 2 2 1 2 3 2 1 1 1 0 1 1 2.83

=35+ =35+ 102 103 2 2 2 1 1 0 1 1 2 0 1 2 0 3.92

25-<30 30-<35 103 95 2 1 2 2 2 2 0 1 1 1 0 1 2 0 3.64

25-<30 30-<35 101 92 1 2 1 2 1 2 0 1 1 0 0 2 1 1 3.88

<20y <20y 100 1 2 2 2 2 1 2 2 1 0 1 3.59

=35+ =35+ 101 87 2 2 1 2 2 1 1 1 1 2 1 1 3.25

30-<35 30-<35 89 95 2 1 2 2 2 3 0 1 1 1 0 1 2 1 3.91

25-<30 25-<30 117 106 2 1 2 2 2 2 0 1 1 2 0 1 2 1 3.02

20+yr 108 122 1 2 1 2 1 1 1 1 1 0 1 2 0 2.78

<20y <20y 107 2 1 1 2 1 1 2 1 1 1 0 1 2 1 2.97

<20y <20y 83 90 1 1 2 2 2 1 1 1 1 0 0 1 2 1 3.29

<20y <20y 112 101 2 2 1 2 2 1 0 1 1 2.77

20+yr <20y 101 1 2 2 2 1 0 1 1 2 1 3.6

30-<35 30-<35 107 93 2 1 1 2 2 2 1 2 1 0 0 1 3 1 3.61

<20y <20y 87 94 1 2 1 2 2 2 0 1 1 1 0 1 2 1 3.36

<20y 25-<30 108 97 2 1 2 3 2 2 0 2 1 2 0 1 3.39

25-<30 =35+ 74 79 2 2 2 2 2 2 2 1 0 0 1 2 1 2.6

<20y <20y 118 93 2 2 2 1 2 3 0 1 1 2 0 1 2 1 3.05

25-<30 25-<30 85 82 2 2 2 1 2 1 2 1 1 1 1 1 2 1 3.02

<20y <20y 106 1 2 2 3 2 2 1 1 1 0 1 2.9

20+yr 25-<30 91 82 2 2 2 2 2 2 0 1 1 2 1 1 3 0 3.9

<20y 30-<35 115 2 2 2 2 2 1 2 1 1 0 0 1 3 0 3.14

<20y =35+ 125 1 2 2 3 3 1 0 1 1 2 0 1 3 0 3.19

<20y <20y 92 88 2 1 2 1 2 2 1 1 1 1 2 2 1 3.82

20+yr <20y 88 1 2 2 2 2 1 1 1 1 1 1 3.26

25-<30 30-<35 105 103 2 1 1 2 2 2 0 2 1 2 0 1 2 1 3.17

20+yr 30-<35 118 1 1 1 2 2 1 0 1 1 0 1 1 2 0 2.94

25-<30 25-<30 97 110 2 2 2 2 3 1 1 1 1 1 0 1 2 1 3.73

=35+ =35+ 110 97 2 2 2 2 3 2 0 1 1 2 0 1 3 1 3.2

20+yr 20+yr 90 2 2 1 2 2 1 1 1 1 3.6

25-<30 25-<30 88 2 2 1 3 3 3 0 1 1 2 0 1 2 1 3.1

<20y 25-<30 104 110 2 2 2 2 2 2 2 1 1 1 0 1 1 1 3.4

<20y 25-<30 103 87 1 2 2 1 2 1 1 1 1 0 2 1 3.205

25-<30 30-<35 115 115 2 1 1 3 2 3 0 1 1 1 0 2 2 0 4.18

30-<35 30-<35 96 114 1 1 2 2 2 1 0 1 1 0 1 1 2 1 4.6

<20y 30-<35 95 89 2 2 1 2 2 1 1 1 1 1 0 1 3.92

20+yr <20y 98 94 1 2 1 2 1 2 0 1 1 0 0 2 2 1 3.35

20+yr 25-<30 84 96 1 1 2 2 2 2 0 1 1 1 0 1 2 0 4.4

<20y <20y 89 1 2 2 2 3 1 0 1 1 1 1 2.335

25-<30 <20y 114 1 2 2 2 1 1 2 2 1 0 0 1 2 0 2.76

<20y <20y 99 93 2 1 1 1 3 1 1 1 2 0 1 2 1 3.12

30-<35 30-<35 98 85 2 1 1 1 2 2 2 2 1 0 0 1 1 0 2.99

<20y 25-<30 118 2 2 2 2 2 2 2 2 1 0 1 0 3.08

<20y <20y 120 100 1 2 2 3 3 1 1 1 2 1 1 2 1 0 3.81

<20y <20y 117 2 1 2 2 2 2 0 1 1 2 0 1 2 1 3.41

<20y <20y 103 1 1 1 2 2 3 0 1 1 2 1 3.625

25-<30 25-<30 109 96 2 2 2 2 2 2 0 2 1 2 0 1 2 1 3.92

30-<35 30-<35 99 2 1 2 2 3 3 0 1 1 2 0 1 3 0 3.95

25-<30 25-<30 78 2 2 1 2 2 3 2 1 1 0 2 1 4.81

<20y 98 89 1 2 2 2 1 2 2 1 0 1 2 0 3.55

20+yr 20+yr 100 1 2 1 2 2 1 0 2 1 1 0 1 2 1 3.82

30-<35 =35+ 105 117 1 1 1 2 1 2 0 1 1 2 1 1 2 0 3.34

25-<30 25-<30 108 2 1 2 3 3 3 0 1 1 2 0 1 2 0 3.46

=35+ =35+ 108 83 2 2 1 2 3 3 1 1 1 0 2 2 0 3.54

25-<30 25-<30 93 93 2 2 1 2 2 2 0 2 1 2 0 1 3 0 4.11

<20y <20y 100 2 1 1 1 2 2 2 2 1 1 0 1 3 1 1.904

<20y 20+yr 95 100 2 2 2 3 2 2 0 1 1 2 0 1 2 0 4

25-<30 30-<35 102 84 1 1 2 1 1 2 2 1 1 1 0 1 3 1 2.51

20+yr 101 2 2 2 2 2 0 1 1 0 1 1 2 0 3.6

20+yr <20y 91 92 1 2 1 2 1 1 1 2 1 1 0 1 2 0 3.33

25-<30 25-<30 105 112 2 2 1 2 2 2 1 1 1 0 1 2 0 2.75

20+yr <20y 85 2 1 2 2 1 2 0 2 1 1 0 1 2 1 3.36

<20y <20y 132 103 2 1 1 3 3 3 0 1 1 2 0 1 2 0 4.2

30-<35 30-<35 96 87 1 1 1 1 1 2 0 1 1 1 1 1 2 1 4.22

<20y 25-<30 120 2 2 2 2 2 3 0 1 1 2 0 2 2 0 3.17

30-<35 25-<30 129 97 2 1 1 2 3 3 0 1 1 0 1 3.02

<20y 25-<30 111 100 2 1 1 2 2 1 0 1 1 2 0 1 2 1 2.8

20+yr <20y 114 104 2 1 1 3 2 2 0 1 1 2 0 1 2 0 4.33

<20y 30-<35 103 1 1 2 3 2 3 0 1 1 2 1 1 2 1 3.49

=35+ =35+ 110 100 2 2 2 2 3 2 0 1 1 2 0 2 3 1 3.47

30-<35 =35+ 112 2 2 2 2 2 0 1 0 0 1 2 1 3.76

<20y <20y 105 100 1 2 2 3 1 0 1 2 1 1 1 2 1 3.14

<20y <20y 120 108 2 2 2 3 2 1 1 1 1 1 3.92

30-<35 25-<30 115 2 1 1 2 2 2 0 1 1 2 0 1 2 0 3.58

<20y 25-<30 112 85 2 2 2 2 2 2 0 1 1 2 1 3.16

20+yr <20y 95 2 2 2 1 1 2 1 1 1 0 1 2 0 2.58

25-<30 25-<30 95 117 2 2 1 2 1 2 0 1 1 1 1 1 2 0 3.02

=35+ =35+ 96 61 2 1 1 3 1 2 0 1 1 2 1 3.24

<20y 25-<30 122 100 1 1 2 1 1 0 1 1 2 0 1 0 4.265

30-<35 30-<35 107 2 1 2 2 2 1 1 1 1 0 1 1 2 1 2.67

<20y 120 1 2 2 2 2 1 2 1 1 1 0 1 3 1 3.65

30-<35 =35+ 111 2 2 1 3 3 2 0 1 1 2 0 1 3 1 3.9

<20y 25-<30 103 1 1 2 2 2 1 0 1 1 1 0 1 1 3.72

<20y <20y 100 88 1 1 1 2 2 2 0 2 1 0 1 3.63

<20y <20y 110 95 1 2 2 2 2 2 0 1 1 1 1 1 2 0 2.89

25-<30 30-<35 109 92 2 1 1 1 1 2 0 2 1 1 1 4.25

30-<35 30-<35 110 107 2 1 2 3 3 3 0 2 1 2 0 1 3 0 3.67

<20y 25-<30 114 86 2 1 1 1 2 3 1 2 1 1 0 1 0 3.65

25-<30 30-<35 118 117 2 1 1 3 3 3 1 2 1 2 0 1 2 0 3.08

25-<30 30-<35 104 106 2 2 2 2 3 2 1 2 1 2 1 1 2 1 3.09

25-<30 30-<35 114 119 2 2 2 2 2 1 0 1 1 0 1 2.85

25-<30 25-<30 114 2 1 1 2 3 1 0 1 1 2 0 1 2 1 3.81

30-<35 =35+ 109 87 2 1 2 1 1 0 1 1 2 0 1 2.31

30-<35 30-<35 114 100 2 1 2 2 2 1 0 1 1 1 0 2 3 1 3.68

25-<30 25-<30 115 2 1 1 2 1 2 2 1 1 1 0 1 2 0 3.39

30-<35 =35+ 91 86 1 1 1 2 3 2 1 1 0 0 1 1 0 3.12

25-<30 =35+ 96 91 2 2 2 2 2 2 2 2 1 0 0 1 3 0 3.64

<20y 25-<30 120 101 2 2 2 3 2 3 0 1 1 2 1 1 1 0 3.4

<20y <20y 100 2 2 1 2 2 2 0 2 1 2 0 1 2 1 3.58

25-<30 25-<30 108 88 2 2 1 3 3 3 0 2 1 1 0 1 2 0 3.17

25-<30 30-<35 109 92 2 2 2 3 2 2 0 1 1 1 2 1 2.88

<20y <20y 82 88 2 2 1 2 1 1 2 1 2 1 0 2 2 0 3.08

25-<30 25-<30 97 95 2 1 1 2 1 2 0 1 1 1 0 1 2 1 3.645

=35+ =35+ 102 102 2 1 2 2 2 2 0 1 1 2 0 1 2 0 4.33

25-<30 25-<30 111 112 2 2 2 3 3 2 0 1 1 1 0 1 2 0 3.14

25-<30 30-<35 97 1 2 1 2 3 3 0 1 1 2 0 1 2 0 3

30-<35 30-<35 107 103 2 2 2 3 3 0 1 1 2 0 1 2 1 3.69

20+yr <20y 92 92 2 2 1 1 1 1 1 1 1 0 1 1 2 1 2.86

20+yr 93 105 1 2 1 1 1 0 1 2 0 3.89

25-<30 30-<35 107 108 2 2 1 3 3 2 0 1 1 1 0 2 2 1 3.92

<20y <20y 102 94 1 1 3 3 1 1 2 1 2 0 1 3 0 3.61

25-<30 30-<35 112 104 2 2 1 2 2 3 0 1 1 2 1 1 2 1 3.88

<20y 25-<30 120 108 2 2 1 2 2 3 0 1 1 1 1 1 2 1 3.52

<20y 25-<30 100 100 2 1 2 3 2 2 0 2 1 1 0 1 2 1 3.43

<20y <20y 100 2 1 2 3 2 2 0 1 1 1 0 1 2 0 4.17

30-<35 =35+ 94 88 2 2 2 2 1 3 0 1 1 2 0 1 0 3.78

<20y 25-<30 112 92 2 1 2 2 2 1 2 1 1 1 0 1 2 0 2.69

20+yr <20y 105 102 1 2 2 2 2 3 0 1 1 1 0 1 2 1 3.24

25-<30 25-<30 114 93 2 1 2 3 3 3 0 1 1 2 0 1 2 0 3.3

20+yr <20y 95 100 2 2 2 2 2 2 2 2 1 1 0 1 3.1

30-<35 =35+ 113 100 2 1 2 3 3 3 0 1 1 2 0 1 2 1 3.25

<20y 25-<30 103 95 2 1 1 2 2 2 0 1 1 0 0 1 3 0 3.62

25-<30 30-<35 114 93 1 1 1 2 1 3 1 1 1 0 1 3.96

<20y =35+ 115 114 1 2 1 2 1 2 2 1 1 2 0 1 2 0 3.23

<20y <20y 107 110 2 1 1 2 1 2 1 2 1 0 0 1 2 1 1.24

25-<30 30-<35 111 101 2 2 2 2 2 2 0 1 1 1 0 1 1 3.14

<20y 25-<30 118 1 1 1 1 2 2 2 2 1 0 2 1 2.05

<20y 25-<30 101 2 1 2 3 3 3 0 1 1 2 0 1 2 1 3.01

<20y 25-<30 115 112 1 2 1 2 1 2 0 1 1 2 0 1 2 0 3.39

<20y 20+yr 100 93 1 2 1 2 2 1 1 1 1 2 0 1 3 0 3.49

25-<30 25-<30 91 90 1 1 1 3 3 2 0 1 1 0 1 1 2 1 3.85

30-<35 25-<30 123 102 2 1 1 2 3 3 0 1 1 2 0 1 0 3.55

=35+ =35+ 91 2 1 2 1 2 2 0 1 1 2 1 1 2 0 3.63

20+yr 25-<30 94 98 2 1 1 2 2 1 2 2 1 1 0 1 2 1 2.5

30-<35 30-<35 105 107 2 2 2 2 2 1 0 1 1 2 0 1 2 1 3.54

<20y 30-<35 106 114 1 2 2 2 2 3 1 1 1 1 1 3.16

25-<30 25-<30 108 104 1 2 2 3 3 1 2 1 1 2 0 1 3 0 3.36

25-<30 <20y 92 83 2 1 2 1 2 2 0 1 1 0 0 1 3 1 2.72

25-<30 =35+ 114 112 2 2 1 2 3 3 0 2 1 1 1 2 0 3.16

<20y <20y 100 97 2 1 2 2 2 3 2 2 1 2 0 1 2 1 3.09

25-<30 25-<30 114 104 2 1 1 2 3 2 1 1 1 2 1 1 2 0 2.87

<20y <20y 95 86 2 2 2 2 2 1 2 1 1 1 0 1 1 4.055

25-<30 25-<30 101 106 2 1 2 3 2 3 0 1 1 2 0 1 3 1 3.23

30-<35 =35+ 100 95 2 2 2 2 3 2 0 1 1 1 1 1 0 3.01

<20y <20y 92 90 2 2 1 2 2 2 1 1 1 1 0 1 2 0 2.27

25-<30 25-<30 99 80 1 2 2 2 2 1 2 1 2 0 0 2 2 1 3.04

25-<30 30-<35 109 95 2 1 1 2 2 3 0 1 1 1 0 1 3 0 2.82

25-<30 =35+ 92 97 2 2 1 2 3 3 1 1 1 2 1 3.42

25-<30 30-<35 105 69 1 2 3 3 1 0 1 1 2 1 2.55

<20y 30-<35 117 110 1 2 2 3 2 2 0 1 1 2 0 1 2 1 4.08

25-<30 =35+ 83 92 1 2 2 3 2 1 1 1 2 1 1 0 3.97

20+yr 110 84 1 1 2 1 0 1 1 0 2 2 1 3.84

25-<30 25-<30 93 98 2 1 1 2 3 2 0 1 1 1 0 1 2 1 3.07

<20y <20y 110 103 2 1 2 2 2 1 0 1 1 2 0 1 1 0 3.08

30-<35 =35+ 105 107 2 2 1 2 2 3 0 2 1 1 0 1 2 0 3.79

<20y 25-<30 105 88 2 1 2 2 2 2 0 1 1 0 0 1 0 3.51

25-<30 30-<35 104 88 2 1 2 2 2 2 0 1 1 0 0 2 2 1 3.75

=35+ =35+ 106 90 1 2 2 2 1 1 0 1 1 0 0 1 2 1 3.19

25-<30 25-<30 132 104 2 2 1 3 3 2 0 1 1 2 0 2 2 1 4.09

<20y 25-<30 99 101 1 1 1 2 2 2 2 2 1 1 0 1 2 0 3.5

30-<35 =35+ 105 2 2 2 1 2 2 0 1 1 2 0 2 2 0 3.37

20+yr <20y 101 103 1 2 2 2 1 1 1 1 1 1 0 1 2 0 3.71

25-<30 =35+ 101 110 2 2 2 2 1 2 0 1 1 2 0 1 3 1 3.97

<20y <20y 97 101 1 1 2 2 2 2 2 1 1 1 1 3.85

=35+ =35+ 100 103 2 2 1 2 3 2 0 1 1 2 0 1 1 2.025

25-<30 25-<30 99 106 2 2 1 3 2 1 0 1 1 0 1 3.62

25-<30 25-<30 103 98 2 1 1 3 3 2 0 1 1 2 0 2 2 0 3.98

<20y <20y 101 101 2 1 1 2 2 2 1 2 1 1 1 1 3 1 3.57

<20y <20y 103 2 1 1 2 2 3 0 1 1 0 1 2.47

<20y 25-<30 94 90 2 2 2 2 2 2 0 1 1 0 0 1 3 1 3.53

25-<30 25-<30 123 91 2 1 1 2 2 3 1 1 1 0 1 2 0 2.86

<20y 25-<30 94 82 2 1 2 2 2 1 0 1 2 0 0 1 2 1 2.56

25-<30 =35+ 94 94 2 1 1 2 2 3 0 1 1 2 0 2 2 1 3.82

20+yr <20y 95 1 2 2 2 2 3 0 1 1 1 1 2.78

30-<35 25-<30 103 85 2 2 1 2 1 0 1 1 0 0 1 2 1 3.15

25-<30 30-<35 114 108 2 1 1 2 3 1 2 2 1 2 0 1 2 0 3.28

20+yr <20y 91 101 2 1 2 2 2 3 0 1 1 2 1 1 2 0 3.42

<20y 25-<30 101 103 2 2 2 2 1 2 2 1 1 0 0 1 2 0 3.03

25-<30 25-<30 94 91 2 1 2 2 2 1 0 1 1 1 0 1 1 4.13

20+yr 20+yr 100 1 2 2 2 2 1 0 1 1 1 0 2 3 0 3.76

=35+ 30-<35 94 90 1 2 1 1 3 2 0 1 1 1 1 1 2 0 4.62

<20y 30-<35 99 93 2 1 1 2 2 3 0 1 1 2 1 3.09

<20y 25-<30 106 85 2 2 2 3 1 3 0 1 1 2 1 1 2 0 3.16

20+yr <20y 103 2 1 1 2 2 2 1 1 3.05

=35+ =35+ 112 90 2 2 2 2 2 2 0 1 1 0 0 1 3 0 4.16

20+yr 102 1 2 2 2 2 0 1 1 0 1 1 3 1 3.98

<20y 86 2 1 1 1 1 0 1 3.04

20+yr <20y 96 102 2 1 1 2 2 1 1 1 1 2 0 1 2 0 3.5

25-<30 30-<35 114 101 2 2 2 0 1 1 0 1 2 1 3.98

<20y 25-<30 114 100 2 1 1 2 2 2 2 1 1 0 1 3.13

<20y <20y 96 104 1 2 2 3 3 3 0 1 1 1 1 3.09

25-<30 30-<35 100 96 2 1 1 2 2 3 0 2 1 2 0 1 2 0 2.92

25-<30 25-<30 102 110 2 1 2 2 2 2 0 1 1 2 1 1 2 0 3.16

30-<35 =35+ 120 1 2 2 1 1 1 0 1 1 2 0 2 0 3.2

<20y <20y 90 86 1 1 2 1 1 2 2 1 1 1 0 1 2 0 3.07

<20y 25-<30 98 82 2 1 1 1 3 2 0 2 1 1 0 1 1 1 3.68

30-<35 25-<30 90 92 2 1 1 2 2 2 0 2 1 2 0 1 2 0 3.72

25-<30 25-<30 104 101 2 2 2 2 3 2 1 2 1 1 1 1 2 0 2.715

<20y 30-<35 103 2 2 2 2 2 3 2 1 1 1 1 3.51

20+yr 25-<30 111 100 1 2 1 2 3 3 0 1 1 0 3 0 3.35

<20y 25-<30 118 88 1 1 2 2 1 3 0 1 1 1 1 1 3 0 4.04

<20y <20y 87 81 1 2 2 1 1 1 2 1 1 1 2 2 1 2.68

<20y <20y 111 2 1 2 3 3 2 0 1 1 2 0 1 2 0 4.06

20+yr 25-<30 92 1 1 1 2 2 1 0 1 1 1 0 1 2 1 2.78

<20y <20y 85 2 2 1 2 2 1 0 1 1 1 0 1 3 1 2.69

=35+ =35+ 104 124 1 2 1 2 2 3 0 1 1 2 0 1 2 0 3

<20y 30-<35 108 88 2 2 1 2 2 2 2 1 1 1 0 1 2 0 3.58

20+yr <20y 108 1 2 2 2 2 3 0 1 1 1 1 1 1 2.65

25-<30 30-<35 90 87 2 1 1 2 2 1 2 1 1 0 0 1 2 0 3.46

<20y 25-<30 100 95 2 2 1 2 2 1 1 2 0 2 2 1 3.03

<20y <20y 102 2 1 2 2 2 2 0 2 1 1 0 1 2 0 3.07

30-<35 30-<35 105 2 1 2 2 2 3 0 1 1 0 0 1 2 1 2.95

20+yr <20y 109 99 1 2 2 2 2 2 0 2 1 2 1 1 1 0 3.86

25-<30 30-<35 90 88 2 2 2 2 2 3 0 1 1 1 1 2 2 1 4.15

<20y <20y 120 1 2 1 2 1 3 1 2 1 1 1 3.37

30-<35 =35+ 112 1 2 1 2 2 2 0 2 1 0 1 1 2 0 4.2

<20y 25-<30 110 110 2 1 1 2 1 2 0 1 1 1 0 1 2 0 2.92

20+yr <20y 86 2 2 2 2 1 2 0 1 1 1 1 1 2 1 3.9

25-<30 25-<30 112 110 1 2 1 2 2 3 2 2 1 1 2 2.28

<20y <20y 100 103 2 2 1 2 2 2 1 1 1 1 0 1 2 1 3.82

<20y 25-<30 94 1 2 1 2 2 1 2 1 2 1 1 3.82

20+yr <20y 95 104 1 2 2 3 2 2 0 1 1 1 0 1 2 1 3.1

30-<35 30-<35 117 79 2 1 1 1 1 0 1 1 2 0 1 2 1 3.26

<20y 25-<30 117 2 1 1 3 2 3 1 1 1 2 0 1 2 1 2.79

<20y 30-<35 114 85 1 2 1 3 1 1 0 1 1 1 0 1 3 0 4.19

20+yr 20+yr 109 102 1 2 2 2 1 1 1 1 1 1 0 1 1 1 3.45

25-<30 30-<35 113 98 2 2 1 3 3 1 0 1 1 2 1 1 2 1 4.15

30-<35 30-<35 111 95 2 1 2 2 1 0 1 2 0 1 3 1 3.38

25-<30 =35+ 68 2 1 3 1 1 0 1 2 1 2 1 4.13

<20y 30-<35 97 89 1 1 1 1 1 2 1 0 0 2 0 2.92

20+yr 99 100 2 2 2 1 0 1 1 2 1 3.34

<20y 25-<30 97 97 2 2 2 1 2 2 0 1 1 1 1 1 3 0 3.19

<20y 25-<30 115 108 2 2 2 3 3 3 0 1 1 2 0 1 2 1 4.2

25-<30 =35+ 106 77 1 1 2 3 2 2 1 0 1 3.47

30-<35 =35+ 103 91 2 2 3 2 2 1 1 1 1 1 2 0 3.13

30-<35 =35+ 99 91 2 1 1 2 2 2 0 2 1 2 0 1 2 0 3.55

<20y 25-<30 107 91 2 2 1 1 1 1 0 1 1 2 0 1 2 1 2.486

30-<35 <20y 100 103 2 2 2 2 2 1 2 1 2 2 0 1 2 0 3.5

25-<30 25-<30 98 98 2 2 2 3 3 3 0 1 1 1 0 1 0 3.6

<20y =35+ 100 2 1 1 2 2 3 0 1 1 1 1 2.08

<20y <20y 101 92 2 2 1 2 2 1 1 1 1 1 1 1 2 1 3.26

20+yr <20y 80 92 1 2 1 2 2 1 1 2 1 1 0 1 2 1 2.34

<20y <20y 97 110 2 2 2 2 3 3 1 1 1 1 1 1 2 1 3.06

25-<30 25-<30 109 96 2 2 2 2 2 2 0 1 1 1 2 1 4

<20y 25-<30 115 101 2 1 2 2 2 2 0 1 1 1 0 1 2 0 3.46

30-<35 30-<35 105 95 2 2 2 2 1 1 0 1 1 2 0 1 1 1 3.89

25-<30 =35+ 82 108 2 2 1 2 3 3 0 1 1 1 0 1 2 1 3.72

25-<30 =35+ 114 2 1 2 1 3 3 0 1 1 1 1 2 2 1 3.07

30-<35 30-<35 94 85 2 1 2 2 2 3 0 1 1 1 0 1 2 0 3.11

<20y <20y 99 1 1 1 2 2 1 0 2 1 0 1 1 2 1 4.11

30-<35 30-<35 104 91 1 1 2 2 3 2 1 1 1 0 1 2 1 3.22

<20y <20y 115 112 2 2 2 3 3 1 0 1 1 1 0 1 2 0 2.89

25-<30 30-<35 117 100 2 1 2 2 2 3 0 2 1 2 1 1 2 0 2.855

30-<35 30-<35 117 2 2 2 1 1 3 0 1 1 1 0 1 2 1 3.49

20+yr 20+yr 91 101 1 2 1 2 2 2 0 1 1 1 0 1 1 1 3.12

30-<35 30-<35 104 81 2 2 1 1 3 2 1 1 1 2 0 1 3 0 4.27

20+yr <20y 106 2 2 2 1 2 1 2 2 1 2.97

25-<30 30-<35 94 80 2 1 2 2 2 1 0 1 1 0 0 1 3 1 3.69

25-<30 30-<35 107 106 2 1 1 2 2 0 1 1 1 1 3.27

<20y <20y 117 108 1 1 1 3 3 2 2 1 1 2 0 1 2 0 3.78

=35+ =35+ 96 2 2 2 1 1 2 0 1 1 1 0 1 3.55

25-<30 25-<30 85 2 2 2 2 2 0 1 1 2 0 1 2 0 3.45

25-<30 25-<30 110 2 2 2 2 3 3 0 1 1 2 0 1 2 0 2.75

<20y <20y 90 82 1 1 1 2 2 2 0 2 1 0 1 2.45

<20y 25-<30 111 1 2 2 2 1 1 0 1 1 2 1 3.8

20+yr <20y 106 95 1 2 1 2 2 3 0 1 1 2 0 1 3.5

25-<30 30-<35 114 90 2 2 1 2 3 2 0 1 1 2 0 1 2 3.25

20+yr 20+yr 90 98 1 2 1 2 2 1 0 1 1 1 0 1 2 1 3

30-<35 =35+ 114 100 2 2 2 2 1 3 0 1 1 0 3 1 3.38

<20y <20y 102 98 2 1 2 2 2 2 0 1 1 2 0 1 2 0 3.52

<20y 25-<30 92 94 1 2 1 2 2 2 0 1 1 2 1 1 3 1 3.63

25-<30 25-<30 115 2 2 2 3 3 2 0 1 1 2 0 1 2 1 3.28

=35+ =35+ 115 2 1 2 2 1 2 0 1 1 2 0 1 0 4.01

25-<30 20+yr 103 97 2 2 1 2 2 1 1 2 1 2 0 1 3 0 3.92

25-<30 25-<30 106 94 1 2 1 3 3 2 0 1 1 2 0 2 3 0 3.96

<20y =35+ 108 1 2 1 2 2 3 0 1 1 0 0 2 2 1 3.8

<20y <20y 94 90 2 1 1 2 1 2 1 1 1 0 0 1 3 1 2.93

30-<35 <20y 103 93 2 1 2 2 1 0 1 1 1 0 1 0 4.26

<20y <20y 112 108 1 1 2 2 2 1 0 1 1 3.69

=35+ =35+ 122 2 2 2 3 3 1 0 1 1 2 0 1 4.78

30-<35 100 102 1 2 2 1 1 1 2 1 1 1 4.05

30-<35 =35+ 117 98 1 1 2 2 1 3 1 2 1 0 3.13

<20y 25-<30 106 98 2 2 2 2 3 2 0 1 1 0 0 1 3 0 3.64

30-<35 =35+ 109 83 2 2 2 3 1 2 0 1 1 2 0 2 1 1 2.87

20+yr 20+yr 91 97 1 1 1 1 2 1 0 2 2 1 0 1 2 1 3.62

25-<30 25-<30 99 96 1 2 2 2 2 3 2 2 1 1 1 1 1 0 3.24

30-<35 =35+ 117 94 2 2 1 3 2 1 0 1 1 0 0 1 1 1 3.71

25-<30 30-<35 108 96 2 2 2 3 3 2 0 1 1 2 1 1 3 1 3.51

20+yr <20y 114 1 1 1 2 2 2 0 1 1 2 0 1 2 0 3.36

25-<30 25-<30 110 1 1 2 3 3 2 1 1 1 2 1 3.36

<20y 20+yr 95 91 2 2 1 2 2 3 0 1 1 1 0 1 0 2.7

30-<35 30-<35 117 2 1 1 2 2 3 0 1 1 1 0 1 3 1 3.92

25-<30 25-<30 99 2 1 2 2 3 2 0 1 1 2 0 2 3 0 2.73

30-<35 30-<35 108 87 1 1 2 2 2 2 0 1 1 0 0 2 2 0 3.94

30-<35 30-<35 112 100 2 1 1 2 2 3 1 1 1 1 0 1 1 3.67

<20y <20y 97 87 2 1 1 1 1 1 0 1 1 1 0 2 2 1 2.76

20+yr <20y 103 94 1 2 1 2 2 0 1 1 1 0 1 2 0 3.88

<20y 25-<30 122 91 2 1 1 3 3 3 0 1 1 2 0 1 2 1 3.28

30-<35 <20y 120 102 2 2 2 1 2 2 1 2 1 2 0 1 0 3.22

25-<30 25-<30 104 2 2 2 3 2 2 0 1 1 2 0 1 3 1 3.85

30-<35 =35+ 107 83 1 1 3 1 3 0 1 1 2 1 2 0 2.67

20+yr <20y 96 88 2 2 1 2 2 1 1 1 1 2 0 1 1 3.6

20+yr <20y 100 106 2 1 1 2 2 3 0 1 1 2 0 1 2 0 3

=35+ =35+ 118 78 1 2 3 3 3 0 1 1 1 1 1 3 1 3.64

<20y <20y 107 1 2 2 2 2 3 0 1 1 0 1 1 3 0 3.48

25-<30 30-<35 112 92 2 1 1 2 2 3 0 1 1 2 1 1 3 0 3.31

25-<30 25-<30 114 119 1 1 2 1 1 2 0 2 1 2 0 1 3 0 3.14

25-<30 30-<35 101 91 2 2 2 2 2 3 1 2 1 2 0 1 2 0 3.65

20+yr 103 98 1 2 2 3 1 0 1 1 2 1 1 1 0 4

<20y 25-<30 118 108 2 2 1 3 2 2 0 1 1 2 0 2 3 1 3.3

<20y 25-<30 95 98 2 1 2 3 2 1 1 1 1 2 1 1 3 1 3.74

20+yr 20+yr 95 81 1 1 2 2 0 1 2 2 0 1 2 1 3.66

<20y <20y 112 108 2 1 1 2 1 2 1 1 1 2 0 1 0 4.27

<20y 25-<30 97 92 2 2 2 2 2 3 0 1 1 2 0 1 2 0 2.835

25-<30 25-<30 75 2 1 2 2 2 2 0 2 1 1 0 1 2 1 3.22

30-<35 25-<30 122 102 2 1 2 2 3 2 0 1 1 2 0 2 2 0 3.775

30-<35 =35+ 108 105 1 2 2 3 3 2 0 1 1 2 0 1 2 0 3.34

<20y 25-<30 112 103 1 2 1 2 3 2 0 1 1 2 0 2 3.92

20+yr 102 96 1 2 2 2 1 0 1 2 0 1 1 2 1 3.43

25-<30 25-<30 132 122 1 1 1 3 3 2 2 2 1 2 1 1 3 1 3.09

20+yr <20y 103 2 1 2 2 2 0 1 1 0 1 2 1 0 3.14

<20y <20y 93 98 2 1 2 2 2 2 0 1 1 1 1 1 2 0 3.4

<20y 25-<30 106 77 2 1 1 2 3 1 0 1 1 2 2 4.54

<20y 25-<30 93 97 2 2 2 2 2 2 1 1 1 2 1 2 3 0 2.74

30-<35 30-<35 105 97 2 1 2 2 2 2 0 1 1 2 1 1 3 1 3.17

<20y 30-<35 92 1 1 1 2 1 0 1 1 2 0 1 1 0 5.07

<20y =35+ 95 101 2 1 1 2 3 3 1 1 1 1 0 1 2 1 3.26

25-<30 <20y 114 100 2 2 2 2 2 3 0 1 1 2 1 1 3 1 3.32

<20y <20y 129 110 2 1 1 3 3 2 0 1 1 2 0 1 2 1 3.46

20+yr 98 79 1 2 2 1 2 1 0 1 1 0 1 1 0 3.73

<20y 118 112 1 2 1 2 1 1 1 1 1 1 1 2 1 3.58

<20y 25-<30 101 97 2 1 2 2 2 3 2 2 1 1 1 1 1 0 3.56

25-<30 25-<30 127 139 2 1 1 3 3 3 0 1 1 1 0 1 2 0 3.98

30-<35 30-<35 100 120 1 2 2 2 2 3 0 1 1 1 1 3

25-<30 25-<30 100 101 2 2 2 3 2 2 0 1 1 2 1 1 3 0 3.5

25-<30 25-<30 100 2 1 2 2 2 3 0 2 1 2 0 1 2 0 3.44

<20y <20y 90 104 2 1 1 3 2 3 0 1 1 1 1 1 2 1 3.895

20+yr <20y 91 100 2 2 1 1 2 2 1 1 1 0 0 2 2 1 3.22

30-<35 30-<35 112 2 1 2 2 1 2 1 1 1 1 0 1 3 1 2.99

25-<30 30-<35 115 101 2 2 1 2 2 2 0 1 1 2 1 1 2 0 3.9

<20y <20y 112 95 2 1 2 1 2 2 1 1 1 1 3 1 3.42

<20y 25-<30 129 110 2 2 1 3 2 2 2 1 1 2 0 1 2 4.05

=35+ =35+ 112 102 2 1 1 3 2 0 1 1 1 0 1 2 1 5.28

<20y 25-<30 98 82 2 2 2 2 2 2 0 1 1 0 1 1 2 1 3.03

20+yr 97 92 1 2 2 2 2 0 1 1 1 1 1 2 0 3.58

25-<30 30-<35 114 98 1 1 2 2 3 2 2 1 1 1 0 1 3 1 2.84

25-<30 30-<35 105 100 2 1 1 2 2 2 0 1 1 1 1 3.67

<20y <20y 108 110 2 1 1 2 2 2 2 1 1 2 1 1 2 0 3.91

25-<30 25-<30 97 101 2 1 2 1 2 3 0 1 1 1 0 1 2 0 3.75

25-<30 30-<35 82 94 1 1 1 1 2 1 2 2 1 3.48

25-<30 =35+ 98 1 1 2 2 2 1 0 1 1 0 3 1 3.29

=35+ =35+ 103 95 2 2 1 2 2 3 0 1 1 2 1 1 3.355

<20y <20y 104 96 1 1 1 1 2 1 2 1 1 1 1 1 2 0 3.1

<20y 25-<30 123 114 1 2 2 3 3 3 0 2 1 2 0 1 2 0 3.25

20+yr 25-<30 109 92 2 2 1 1 2 1 2 2 1 0 0 1 2 1 2.58

<20y <20y 101 1 2 2 2 2 1 0 2 1 1 1 1 2 1 3.05

20+yr 25-<30 109 1 2 2 2 2 1 0 1 1 1 0 1 2 1 3.47

<20y 25-<30 129 106 1 2 2 2 3 3 0 1 1 1 0 1 2 1 3.81

30-<35 =35+ 91 97 2 1 2 2 1 2 2 2 1 1 1 1 1 0 3.37

25-<30 25-<30 92 1 2 2 1 2 1 0 2 1 1 1 1 3 0 3.76

25-<30 =35+ 94 89 2 1 2 1 2 3 0 2 1 1 0 1 2 0 4.25

30-<35 30-<35 89 82 1 2 1 2 2 2 0 2 1 0 0 2 3.54

20+yr <20y 82 1 2 1 2 2 3 2 2 1 1 0 1 3 1 3.54

<20y <20y 81 90 1 1 2 2 2 2 1 1 1 0 1 2 0 3.33

<20y <20y 97 2 1 2 2 2 1 1 1 1 2 1 3.11

30-<35 =35+ 124 2 2 2 1 1 2 0 1 1 2 0 1 3 1 4.24

25-<30 30-<35 109 108 2 1 1 2 3 2 0 2 1 2 1 1 2 1 3.6

<20y 25-<30 114 103 1 1 1 2 2 2 0 1 1 1 0 1 2 1 3.54

25-<30 25-<30 118 106 2 2 1 3 3 3 1 1 1 2 1 1 3 0 2.55

20+yr <20y 97 2 2 2 1 1 2 2 2 1 1 1 1 2 1 3.56

<20y 30-<35 91 108 2 2 1 2 2 1 0 1 1 2 1 1 2 0 3.22

20+yr <20y 108 2 1 2 1 3 1 1 1 1 1 2.91

25-<30 30-<35 109 106 2 1 2 2 2 2 1 1 0 1 2.81

<20y 95 101 1 2 1 1 1 2 0 1 2 1 4.59

<20y 25-<30 120 94 2 1 2 2 2 2 0 1 1 1 1 1 2 0 3.96

20+yr <20y 85 1 2 2 3 2 1 0 2 2 1 0 1 2 0 3.09

25-<30 25-<30 101 106 1 1 2 2 2 3 0 1 1 1 0 1 1 1 3.02

<20y <20y 82 87 2 1 1 1 2 1 1 2 1 1 0 1 2 0 4.21

<20y <20y 107 2 2 2 1 2 2 0 1 1 1 0 1 2 0 4.18

<20y 25-<30 117 2 1 2 3 3 2 0 1 1 2 0 1 1 2.99

<20y <20y 117 104 2 2 2 2 3 2 0 1 1 2 1 1 2 0 3.8

20+yr 20+yr 107 98 2 2 1 3 2 1 0 1 1 1 0 1 2 0 3.11

25-<30 <20y 127 103 2 1 1 2 2 2 1 1 1 2 1 2 2 1 3.18

20+yr <20y 108 88 1 2 2 2 2 1 1 1 1 1 1 1 2 1 3.32

<20y <20y 106 90 2 2 1 1 1 1 2 1 1 1 1 1 2 1 3.24

<20y <20y 97 1 2 2 2 1 3 1 2 1 2 1 3

30-<35 30-<35 97 90 2 2 2 1 2 2 0 1 1 2 0 1 2 1 3.94

25-<30 <20y 111 2 2 2 3 2 3 0 1 1 2 1 1 2 1 3.7

25-<30 25-<30 112 112 2 1 1 2 2 3 0 1 1 2 0 1 2 0 2.99

=35+ =35+ 95 85 2 1 1 1 1 0 1 1 1 1 1 2 1 4.42

30-<35 30-<35 112 79 2 2 2 3 3 1 1 1 2 0 1 1 3 1 3.48

=35+ =35+ 112 71 2 2 1 3 2 2 0 1 1 1 0 1 3 1 4.045

30-<35 25-<30 100 105 2 2 1 3 2 3 0 1 1 2 0 1 2 0 3.59

25-<30 30-<35 107 87 2 1 1 2 1 2 0 1 1 1 2 4.355

<20y 25-<30 105 100 1 1 2 2 2 2 0 1 1 1 1 1 2 1 3.39

<20y <20y 92 98 1 2 1 2 2 3 2 1 1 1 0 1 2 1 3.68

25-<30 30-<35 110 96 2 2 2 2 3 2 1 2 1 2 1 1 3 0 2.99

20+yr <20y 114 2 2 2 2 2 1 1 1 2 2 3.35

30-<35 30-<35 105 2 2 1 3 3 2 0 1 1 2 1 2 2 1 2.96

<20y <20y 108 88 2 2 1 2 3 3 1 1 1 0 1 1 2 0 4.32

<20y <20y 109 87 2 1 1 2 1 3 0 1 1 1 1 1 3 0 3.37

<20y 25-<30 107 97 2 1 2 2 2 1 0 2 1 2 1 1 3 0 3.81

<20y 25-<30 104 88 2 1 1 2 2 1 2 2 1 1 0 1 2 1 3.95

25-<30 30-<35 102 110 1 1 2 2 1 2 1 2 1 1 1 1 2 0 3.31

<20y 25-<30 115 103 1 2 2 2 3 3 1 1 1 2 0 1 3 1 1.72

=35+ =35+ 105 114 2 2 1 1 1 3 0 2 1 2 1 1 2 1 3.26

<20y <20y 103 81 2 2 1 2 2 2 0 2 1 0 1 1 2 1 3.23

30-<35 30-<35 106 102 2 1 2 2 1 1 0 1 1 2 1 2 0 2.79

20+yr <20y 79 87 2 2 2 2 2 3 2 1 1 1 1 2 0 3.66

<20y 25-<30 83 87 1 2 1 2 2 1 0 2 1 0 2 3.76

30-<35 =35+ 123 112 2 2 2 3 2 0 1 1 2 1 2 3 0 2.91

30-<35 30-<35 114 109 1 2 1 2 2 3 2 2 1 1 1 2.69

20+yr 30-<35 94 90 1 1 1 1 2 1 0 1 1 0 0 1 3.035

<20y 25-<30 115 112 2 2 2 3 3 1 0 1 1 2 0 1 3 0 4.12

20+yr 99 1 2 2 3 2 1 0 1 1 2 1 1 2 0 4.4

25-<30 30-<35 114 110 2 1 2 1 1 1 0 1 1 2 1 1 2 0 3.97

20+yr 96 90 1 2 2 2 1 0 1 1 1 1 1 2 1 3.63

30-<35 30-<35 83 93 2 2 1 3 1 2 1 1 1 2 1 2 2 0 2.94

20+yr 98 88 1 2 2 2 1 2 1 1 1 1 2 2 1 3.38

25-<30 25-<30 115 2 1 2 3 2 2 2 1 1 2 1 1 2 1 3.08

20+yr 105 100 1 2 1 2 2 1 2 0 0 1 3 0 3.07

<20y <20y 103 92 1 1 2 2 2 1 0 1 1 1 0 1 1 1 3.585

20+yr <20y 110 109 1 1 2 2 2 2 0 1 1 1 1 3.11

25-<30 =35+ 102 2 1 2 2 2 3 1 1 1 0 1 2 3 0 3.76

25-<30 30-<35 82 104 2 1 1 2 2 1 0 1 1 2 1 1 2 0 2.95

<20y <20y 103 1 2 2 1 2 1 1 1 1 1 1 1 3 0 2.79

30-<35 25-<30 105 82 2 2 1 3 1 1 0 1 1 0 0 2 2 1 3.35

<20y 25-<30 111 110 2 1 1 3 3 3 0 1 1 2 0 1 3 1 3.34

20+yr 20+yr 104 1 2 2 2 2 1 1 1 1 0 0 1 2 1 2.47

25-<30 25-<30 101 98 1 1 1 2 1 2 0 1 1 2 0 1 2 1 3.48

30-<35 30-<35 98 102 2 1 2 3 2 1 0 1 1 2 0 1 2 0 3.74

25-<30 =35+ 117 2 1 1 3 3 3 1 1 1 1 1 1 2 1 3.47

20+yr <20y 108 96 2 2 1 2 2 2 0 1 1 2 1 1 2 0 3.39

25-<30 25-<30 105 119 2 2 2 2 3 3 0 1 1 2 1 1 3 0 2.95

25-<30 30-<35 109 108 2 2 2 1 1 3 1 1 1 1 0 1 3 0 3.12

25-<30 25-<30 105 101 1 2 1 2 1 1 2 1 1 1 1 1 2 0 3.9

20+yr 20+yr 101 98 2 2 1 2 2 2 0 1 1 0 1 3.34

20+yr <20y 105 95 2 1 2 2 2 1 0 1 1 1 1 2.52

<20y 25-<30 95 1 2 2 2 2 2 1 1 1 2 0 1 3 0 2.36

<20y 25-<30 100 87 2 2 1 2 2 2 2 1 1 0 0 2 2 0 2.155

<20y 25-<30 92 98 2 1 1 2 2 3 0 2 1 1 1 1 2 1 4.42

25-<30 25-<30 97 1 2 1 2 3 2 2 1 1 1 0 1 2 0 2.8

30-<35 30-<35 108 95 2 1 2 2 2 1 2 1 1 1 1 1 2 1 3.09

<20y <20y 110 94 1 1 1 3 2 1 1 1 1 2 1 1 2 1 3.64

<20y <20y 106 84 2 1 1 1 3 0 2 1 0 0 1 1 1 2.99

25-<30 25-<30 106 110 2 1 2 2 3 1 0 1 1 2 1 2 2 1 3.81

<20y 30-<35 91 108 2 1 2 2 2 3 0 2 1 0 1 1 2 1 3.76

30-<35 30-<35 102 95 2 1 1 2 2 0 1 1 1 1 3 0 4.05

<20y 25-<30 97 106 1 2 2 3 2 3 0 1 1 2 0 2 2 0 4.03

25-<30 25-<30 104 98 2 2 1 3 2 3 1 2 1 1 1 1 2 0 2.28

25-<30 30-<35 102 87 2 2 2 2 1 0 1 1 2 0 1 4.48

<20y <20y 118 112 1 2 1 2 2 3 0 1 1 2 1 2.95

<20y <20y 107 125 2 2 1 2 2 2 1 1 1 1 0 1 2 0 3.54

<20y <20y 98 88 2 2 2 2 2 1 1 2 1 1 1 1 0 3.29

<20y <20y 101 108 2 1 1 2 2 3 0 1 1 2 0 1 2 0 4.02

20+yr 25-<30 84 98 1 1 1 1 1 1 0 1 1 1 1 1 3 0 3.09

30-<35 25-<30 40 44 1 1 1 1 3 2 2 1 0 1 1.98

<20y =35+ 109 2 2 2 2 1 0 1 1 2 1 2 3 0 3.21

<20y 20+yr 115 92 1 2 1 2 2 1 1 2 1 2 1 2 2 0 3.24

25-<30 =35+ 101 106 2 1 1 2 2 2 0 2 1 1 1 1 2 1 3.09

25-<30 25-<30 105 112 2 2 2 2 1 1 0 2 1 0 3 0 3.195

30-<35 25-<30 100 1 1 2 2 3 2 2 1 2 0 1 2 1 3.53

<20y <20y 118 2 1 2 2 3 2 0 1 1 0 1 1 3 0 3.11

<20y <20y 103 110 2 1 2 2 2 2 0 1 1 2 1 1 3 0 4.05

<20y 25-<30 102 95 2 1 2 2 2 2 0 1 1 1 0 1 3 0 3.2

30-<35 30-<35 110 109 2 2 2 3 3 3 2 1 1 1 1 1 2 1 3.66

25-<30 30-<35 84 108 1 2 2 2 1 1 0 1 1 2 1 2 1 3.03

<20y 30-<35 99 96 2 1 2 2 1 2 0 1 1 2 1 3.67

<20y =35+ 105 84 1 1 2 2 3 2 1 1 0 1 2 1 3.82

25-<30 25-<30 120 1 2 1 3 3 3 2 2 1 1 1 1 2 1 2.99

30-<35 =35+ 120 89 2 2 1 2 1 2 0 2 1 2 1 1 3 1 3.545

=35+ 30-<35 97 97 2 1 2 2 1 1 1 1 0 1 1 1 3.05

25-<30 30-<35 115 84 2 1 1 1 2 1 1 1 1 1 1 1 3 1 3.77

<20y 25-<30 112 114 2 2 2 3 2 3 0 1 1 2 1 1 2 1 3.5

25-<30 25-<30 102 100 2 2 1 2 3 2 0 1 1 2 1 1 2 1 4.01

<20y 25-<30 107 96 2 1 2 2 2 3 1 1 1 1 1 1 2 0 3.52

<20y <20y 114 100 2 1 1 2 3 2 0 1 1 2 1 1 2 0 3.83

<20y <20y 100 94 1 1 2 2 2 1 0 1 1 2 1 1 3 1 3.57

20+yr <20y 88 102 1 2 2 2 2 1 1 1 1 1 2 1 2.1

25-<30 25-<30 103 2 1 2 2 2 2 0 1 1 2 1 1 3 0 3.95

25-<30 25-<30 99 88 2 1 1 1 0 2 1 0 1 1 3.9

<20y 25-<30 110 90 1 2 1 2 2 1 2 1 1 2 1 1 1 2.73

<20y <20y 103 80 2 1 1 1 2 1 2 2 1 1 1 2.7

20+yr <20y 102 1 2 1 1 2 1 1 2 1 1 1 1 0 3.645

<20y 25-<30 110 108 2 1 2 2 3 2 0 1 1 2 1 1 2 1 3.7

<20y <20y 120 1 2 2 2 1 3 0 1 1 2 0 1 2 1 3.16

<20y 25-<30 110 114 2 1 2 3 3 1 0 1 1 2 0 1 2 1 3.36

<20y <20y 91 1 2 2 2 2 3 0 2 1 2 1 1 2 1 3.48

20+yr <20y 106 88 2 2 1 2 3 3 1 1 1 1 1 1 2 1 3.54

25-<30 30-<35 92 2 2 1 3 3 1 2 2 1 0 0 1 3 0 3.06

25-<30 25-<30 109 108 2 1 2 1 3 0 1 1 2 1 1 2 0 3.8

<20y 25-<30 100 103 2 2 2 2 2 2 1 1 1 2 0 1 2 0 1.582

<20y 25-<30 98 91 2 1 1 2 2 2 2 2 1 2 1 1 2 1 3.16

20+yr 20+yr 100 90 1 2 2 2 2 2 0 1 1 2 2 2 1 3.85

<20y 25-<30 99 1 2 2 2 3 0 1 2 1 0 1 2 1 3.51

<20y 92 85 2 2 2 2 0 2 1 2 1 1 2 1 3.06

<20y 25-<30 118 97 2 1 1 2 2 2 0 1 1 1 1 1 2 1 3.55

25-<30 25-<30 111 96 2 1 1 3 2 2 2 1 1 0 1 1 3 1 3.68

20+yr 20+yr 95 81 2 2 1 2 2 3 2 1 1 1 1 2.6

25-<30 25-<30 115 98 2 1 1 3 3 3 0 1 1 2 1 1 2 1 3.655

25-<30 30-<35 92 92 1 1 2 3 2 3 0 1 1 1 2 3.67

<20y <20y 81 85 1 1 1 2 2 2 1 1 1 1 1 1 2 1 3.12

20+yr 20+yr 103 97 2 2 2 2 3 1 0 1 1 1 0 1 2 1 3.36

25-<30 25-<30 103 96 2 1 2 2 2 1 0 2 1 1 1 1 2 1 2.92

25-<30 30-<35 103 110 1 1 1 2 2 1 0 1 1 1 1 1 3 0 3.61

<20y 25-<30 110 104 2 1 2 2 2 3 1 1 1 1 1 2 2 0 2.74

=35+ 30-<35 100 1 2 2 1 2 1 2 1 2 2 0 1 2 1 3.35

<20y <20y 107 98 2 2 1 2 2 1 0 1 1 2 0 1 2 0 3.42

<20y <20y 105 92 1 2 2 1 2 2 1 1 1 1 3 0 2.81

30-<35 30-<35 90 87 2 1 1 2 2 1 0 2 1 1 1 2 2 1 3.89

<20y 25-<30 101 86 2 1 2 2 2 1 0 1 1 1 1 1 3.26

25-<30 <20y 96 98 2 1 1 1 1 1 2 1 1 1 0 2 2 1 3.13

<20y 25-<30 120 117 2 1 1 2 2 3 0 1 1 1 1 3.63

30-<35 25-<30 101 1 1 1 3 1 2 1 1 1 2 1 1 3.29

<20y 30-<35 96 1 2 2 2 2 0 1 1 1 1 3.8

=35+ =35+ 95 100 2 2 1 2 1 1 1 1 2 0 1 3 1 3.04

25-<30 30-<35 120 103 2 2 2 3 2 2 0 1 1 2 1 1 3 0 3.12

20+yr 25-<30 98 2 2 2 2 2 3 1 2 1 1 0 1 3 1 3.33

<20y 25-<30 97 91 2 2 2 2 2 2 0 1 1 1 0 1 2 0 3.04

20+yr <20y 90 2 2 2 2 2 1 0 1 1 1 1 1 2 1 3.12

20+yr 20+yr 85 112 1 2 1 3 1 1 1 1 1 2 1 1 2 1 3.9

30-<35 =35+ 122 97 2 2 1 1 1 0 1 1 1 0 1 0 2.65

<20y 25-<30 92 90 2 1 1 2 2 3 0 1 1 1 1 1 2 0 2.5

<20y <20y 89 91 1 2 1 2 3 1 0 1 1 2 1 1 2 0 2.98

30-<35 30-<35 112 97 2 2 1 2 3 3 2 2 1 0 1 2 2 1 2.44

25-<30 30-<35 95 91 2 1 2 2 2 3 0 1 1 1 1 3 0 3.69

25-<30 25-<30 103 1 1 2 3 1 2 0 1 1 1 1 2 3.78

<20y 25-<30 93 87 1 2 2 2 1 2 1 1 1 1 1 2 0 2.91

<20y <20y 117 100 2 1 1 2 3 1 0 2 1 2 1 2 2 0 3.13

<20y <20y 123 100 2 1 2 2 2 2 0 1 1 2 1 1 1 4.2

20+yr <20y 99 2 1 1 1 2 1 2 1 1 2 1 2 2 1 2.95

25-<30 25-<30 93 100 1 1 1 2 1 2 0 1 1 0 1 2 2 1 1.88

25-<30 25-<30 97 93 2 1 2 2 2 3 0 1 1 0 1 1 2 0 2.36

25-<30 =35+ 102 2 1 2 1 1 3 2 1 1 1 1 1 2 0 3.05

25-<30 30-<35 95 80 1 1 1 2 2 1 1 2 1 2 1 1 2 1 3.6

20+yr <20y 98 84 2 1 2 3 3 1 2 2 0 1 1 2 1 3.85

<20y <20y 115 85 2 1 1 2 3 3 1 2 1 2 1 1 0 3.9

<20y 101 89 1 2 1 2 1 0 1 1 1 1 1 2 1 4.46

20+yr <20y 110 96 2 1 1 2 2 2 2 1 1 2 1 1 3 1 3.59

<20y <20y 95 1 1 2 2 2 1 1 1 1 2 0 2.89

25-<30 <20y 110 103 1 2 1 1 2 2 0 1 3.38

<20y 25-<30 104 97 1 2 2 2 2 3 0 1 1 1 1 3.25

<20y 30-<35 106 106 2 1 2 3 3 3 0 1 1 2 1 1 3.6

<20y 25-<30 109 91 2 1 2 3 3 2 0 1 1 1 1 1 2 0 3.35

30-<35 25-<30 99 103 2 2 2 2 3 3 1 2 1 0 1 1 2 0 2.86

30-<35 25-<30 110 2 1 1 3 3 3 0 1 1 2 0 1 2 0 3.47

30-<35 30-<35 103 77 1 1 1 1 1 0 2 1 0 3 1 3.15

<20y 92 86 1 2 2 2 2 1 2 1 1 1 1 1 2 1 2.86

30-<35 25-<30 119 87 2 2 1 2 2 1 0 2 2 1 1 1 3 1 3.6

20+yr <20y 106 101 2 2 1 2 2 1 1 1 1 0 1 1 2 0 3

20+yr <20y 109 94 2 2 2 2 3 2 0 2 1 1 0 1 2 1 2.97

25-<30 25-<30 102 106 2 1 2 3 2 3 0 1 1 2 1 1 2 0 3.805

30-<35 30-<35 107 84 2 1 1 1 2 2 0 1 1 2 0 1 3.04

20+yr <20y 107 101 2 2 2 2 2 2 2 1 1 2 1 1 3.85

20+yr <20y 106 92 2 2 1 2 2 1 1 1 1 2 1 1 3 0 3.11

<20y 25-<30 112 95 2 1 2 2 2 2 0 1 1 2 1 1 2 0 3.26

25-<30 25-<30 112 108 2 1 1 3 3 2 0 1 1 2 1 2 2 1 3.13

20+yr <20y 97 2 1 1 2 2 1 1 2 1 1 2 1 2.98

25-<30 25-<30 111 103 2 1 2 2 3 2 0 1 1 2 1 1 2 1 3.45

<20y 30-<35 102 103 2 2 1 2 2 3 0 1 1 2 1 1 2 1 3.45

<20y 30-<35 103 92 2 1 2 2 1 3 0 1 1 2 1 1 2 0 2.755

25-<30 25-<30 98 108 2 1 2 2 2 1 2 2 1 2 0 1 3.17

30-<35 30-<35 98 100 1 1 2 2 2 3 1 1 1 2 1 2 3 0 3.6

25-<30 25-<30 100 104 2 1 1 2 2 2 0 1 1 0 1 3 0 3.23

25-<30 30-<35 89 86 2 2 2 2 2 2 0 1 1 2 0 1 0 2.97

25-<30 25-<30 127 103 2 1 1 2 2 1 0 1 1 2 1 1 3 0 3.08

<20y <20y 110 2 2 1 2 2 1 0 1 2 1 0 1 2 1 3.3

25-<30 30-<35 111 97 1 1 2 3 2 2 0 1 1 0 1 1 2 0 2.384

30-<35 =35+ 94 98 2 2 2 1 2 2 0 1 1 2 1 1 0 2.96

30-<35 =35+ 103 94 2 2 1 2 1 1 2 1 1 2 1 1 2 0 3.17

30-<35 30-<35 91 100 2 2 2 2 2 3 0 1 1 2 0 2 3.49

<20y <20y 106 92 2 2 1 3 3 2 0 1 1 2 1 2 3 1 3.65

25-<30 30-<35 114 91 2 1 2 2 2 2 1 1 2 1 1 2 1 3.4

30-<35 =35+ 108 117 2 2 2 3 2 3 0 1 1 2 1 1 2 1 3.82

=35+ =35+ 103 96 2 2 1 1 1 3 0 1 1 1 1 1 0 3.44

=35+ =35+ 117 98 2 1 2 2 2 0 1 0 1 1 2 0 3.95

<20y 25-<30 118 114 1 1 3 3 2 0 1 1 2 1 1 3 0 3.48

25-<30 25-<30 97 104 1 1 2 2 1 2 1 1 1 1 4

25-<30 30-<35 103 100 1 1 1 3 3 3 0 1 1 2 1 1 1 0 3.78

25-<30 30-<35 100 82 2 1 3 2 0 1 0 1 3.22

30-<35 =35+ 80 87 2 1 1 2 1 2 2 1 1 1 0 1 2 1 3.68

<20y <20y 110 117 2 1 2 2 3 2 0 1 1 2 1 1 2 0 3.65

30-<35 25-<30 122 99 1 2 2 3 3 3 0 1 1 2 1 1 1 3.83

<20y 25-<30 106 2 2 1 3 2 3 0 1 1 1 0 1 2 0 4.11

<20y 114 101 1 2 1 2 3 2 2 1 0 1 2 0 2.56

25-<30 30-<35 104 95 2 1 1 3 2 3 0 1 1 2 0 1 1 1 3.58

25-<30 30-<35 85 97 1 1 1 2 2 3 0 1 1 0 1 1 2 1 4.51

<20y 30-<35 103 97 2 2 2 2 3 2 2 1 2 1 1 3 0 3.82

<20y <20y 104 104 2 2 2 2 2 3 0 1 1 1 1 1 2 0 3.53

<20y <20y 117 110 2 1 2 3 3 3 0 1 1 1 1 1 3 0 3.24

<20y 25-<30 92 84 2 2 1 2 2 2 2 2 1 0 1 1 2 1 2.49

<20y 25-<30 118 88 1 2 1 2 2 3 0 1 1 1 1 1 3.52

<20y <20y 97 87 2 2 2 1 2 3 0 2 1 1 0 1 3.41

20+yr 103 94 1 1 2 2 1 1 1 1 0 1 1 1 1 3.86

<20y 25-<30 110 97 1 2 1 2 2 2 0 1 1 1 0 1 2 0 3.4

25-<30 30-<35 112 93 2 1 3 3 3 0 1 1 2 1 1 2 0 2.55

20+yr <20y 104 2 2 2 2 2 3 1 1 1 0 0 1 2 1 3.97

25-<30 25-<30 104 89 2 1 3 0 2 2 1 2 3.32

<20y 120 93 2 1 2 2 2 2 2 1 0 0 1 0 2.53

30-<35 =35+ 98 98 2 2 2 2 2 2 0 1 1 0 1 3.75

25-<30 89 103 1 2 1 2 2 1 2 1 1 0 1 2 1 2.235

25-<30 25-<30 95 98 2 1 2 2 2 3 0 1 1 2 0 1 1 3.25

20+yr 99 92 1 2 2 2 1 0 1 1 0 1 1 2 1 2.74

20+yr 25-<30 103 2 1 2 2 3 3 1 1 1 1 1 1 2 1 2.64

20+yr 20+yr 94 2 2 2 2 1 2 1 1 0 1 1 1 2.81

<20y <20y 98 112 2 1 1 2 2 2 2 2 1 0 1 2 2 1 3.81

<20y 25-<30 92 92 1 2 2 1 1 2 2 1 1 2 1 2.79

20+yr <20y 110 2 2 2 3 3 1 0 1 1 2 1 1 2 0 3.22

20+yr 114 1 2 1 2 3 2 2 1 0 1 1 2 1 3.55

30-<35 25-<30 109 88 2 1 2 1 1 1 0 1 1 0 1 1 3 1 2.455

25-<30 25-<30 103 101 2 1 2 2 2 3 0 1 1 2 1 2 2 1 3.6

<20y 25-<30 100 93 2 1 1 2 2 3 1 2 1 1 1 1 2 0 3.5

25-<30 100 100 1 2 1 3 1 0 1 1 2 1 1 2 0 3.91

<20y 25-<30 105 97 2 1 1 2 3 3 0 1 1 1 1 1 2 1 3.17

<20y <20y 101 86 2 2 2 2 2 3 0 1 1 2 1 1 2 0 4.12

25-<30 102 96 1 1 1 3 3 2 0 2 1 2 1 1 2 0 3.95

25-<30 30-<35 102 93 2 1 2 1 2 3 0 2 1 0 1 3.45

<20y <20y 109 96 2 2 2 2 1 3 0 1 1 2 0 1 2 1 3.05

<20y 30-<35 120 110 2 1 1 2 2 2 2 1 1 2 1 1 2 0 3.11

<20y <20y 107 104 2 2 1 2 2 2 0 1 1 0 1 1 3 0 3.39

<20y 25-<30 84 96 2 2 1 3 3 3 1 1 1 1 1 1 2 0 3.32

<20y 25-<30 103 87 1 2 1 3 3 1 0 1 1 1 1 1 2 0 4.03

<20y <20y 80 91 1 1 2 1 1 2 2 2 1 0 1 1 2 1 2.43

30-<35 =35+ 97 107 2 1 2 1 1 2 2 1 1 2 1 1 2 0 3.5

30-<35 =35+ 117 107 2 2 1 2 2 3 2 2 1 1 1 2 0 3.45

25-<30 25-<30 93 87 1 2 1 2 2 1 0 2 1 0 1 1 2 1 3.65

=35+ =35+ 94 88 1 2 1 1 2 1 0 1 1 2 1 3.31

25-<30 25-<30 120 108 2 1 1 3 3 3 2 1 1 1 0 1 3 0 2.95

25-<30 25-<30 103 92 2 1 2 2 2 3 0 2 2 1 2 0 3.24

25-<30 =35+ 91 1 2 2 2 1 1 2 1 1 1 1 2 0 3.57

<20y <20y 106 95 2 2 2 2 2 2 0 1 1 0 1 1 2 0 1.54

20+yr <20y 106 104 2 2 2 2 3 3 0 1 1 1 1 1 2 0 4.04

25-<30 25-<30 99 104 2 1 2 2 2 2 0 1 1 2 1 1 2 1 3.52

<20y <20y 94 1 1 1 3 2 3 2 2 1 1 1 1 2 0 2.75

<20y 25-<30 117 104 2 2 1 2 2 2 2 2 1 2 1 1 2 0 3.25

25-<30 25-<30 100 93 2 1 1 2 2 1 0 1 1 0 1 1 3 1 3.8

30-<35 25-<30 101 99 1 1 2 2 2 3 0 1 1 1 1 1 2 0 3.05

25-<30 25-<30 98 92 2 1 2 2 2 2 1 1 1 1 1 3.44

30-<35 30-<35 110 2 2 2 3 3 3 0 1 1 2 1 1 3.375

25-<30 25-<30 102 104 2 1 2 3 2 3 0 1 1 2 1 1 2 1 3.64

25-<30 25-<30 107 90 2 1 1 2 2 0 1 1 2 0 1 3.995

<20y <20y 95 85 2 2 1 2 2 2 1 1 1 1 1 2 3.58

<20y 25-<30 110 92 2 1 2 3 3 3 2 2 1 1 1 1 2 0 2.65

<20y <20y 107 114 1 2 2 2 2 3 1 1 1 1 1 2 0 3.72

<20y <20y 118 1 2 2 1 2 2 0 1 1 1 1 2.985

30-<35 =35+ 115 98 2 2 2 3 3 2 0 1 1 1 0 1 2 0 3.02

<20y 30-<35 97 95 1 2 2 3 2 3 2 1 1 2 0 1 0 3.17

25-<30 30-<35 109 2 1 2 2 2 2 0 2 1 2 1 1 3 0 3.51

<20y <20y 99 104 2 1 2 2 2 3 2 1 1 1 0 1 2 1 3

30-<35 =35+ 90 80 2 2 1 1 2 2 0 2 1 1 1 3.98

<20y <20y 92 57 2 1 2 2 2 1 0 1 1 0 1 3.04

20+yr 30-<35 108 100 2 1 1 2 1 2 0 1 1 2 1 1 2 0 2.65

20+yr 101 99 1 2 2 2 1 0 1 1 1 1 2.27

25-<30 30-<35 92 89 2 1 2 3 2 3 0 1 1 1 0 2 1 0 3.12

25-<30 30-<35 109 110 2 1 1 3 1 3 0 1 1 2 1 2 2 1 3.27

=35+ =35+ 85 2 2 2 2 2 3 0 1 1 1 1 4.01

<20y <20y 99 85 1 2 2 2 2 1 0 2 1 0 1 1 2 1 3.9

30-<35 25-<30 97 87 2 2 1 2 2 0 2 1 1 0 1 2 1 1.495

<20y 25-<30 101 90 2 2 1 2 2 2 0 1 1 2 1 1 2 0 3.7

20+yr <20y 100 101 2 2 2 2 2 2 0 1 1 2 0 3.32

<20y 25-<30 104 89 1 1 2 3 3 1 0 1 1 2 1 1 2 0 3.83

25-<30 30-<35 103 91 2 2 1 2 2 2 0 1 1 2 1 1 3 1 3.56

<20y 25-<30 89 94 2 2 1 1 1 3 0 1 1 0 0 1 2 1 2.74

<20y 25-<30 115 85 1 1 1 2 2 2 0 1 1 2 1 1 2 1 3.91

<20y <20y 110 1 2 2 2 2 2 2 1 1 0 1 1 2 0 3.61

<20y <20y 96 87 2 2 1 1 3 3 0 1 1 2 1 1 2 1 3.78

25-<30 25-<30 107 86 2 1 1 2 3 3 0 1 1 1 1 1 2 0 3.65

25-<30 25-<30 106 101 1 1 1 1 2 1 2 2 1 1 2 3.41

<20y 25-<30 102 94 1 2 2 2 1 2 0 1 1 2 1 1 2 0 2.83

30-<35 =35+ 111 2 1 2 2 2 1 2 1 1 1 1 1 2 0 3.43

20+yr <20y 85 88 1 2 2 2 2 1 1 1 1 1 3.37

<20y <20y 107 101 2 2 2 3 1 2 0 2 1 2 1 3.88

<20y =35+ 105 1 2 3 3 3 0 1 1 2 1 1 2 0 3.7

20+yr <20y 109 95 2 1 1 2 1 2 0 1 1 2 1 1 2 1 3.87

30-<35 25-<30 106 82 2 2 1 3 2 1 2 1 1 0 1 1 2 1 4.155

25-<30 25-<30 120 95 1 1 2 2 2 0 1 1 1 1 1 0 1.905

20+yr 20+yr 118 101 1 2 1 2 2 3 0 1 1 0 1 1 2 0 3.61

<20y 25-<30 117 110 2 1 2 1 2 3 2 1 1 2 1 1 2 0 3.38

25-<30 =35+ 98 93 2 2 2 2 1 1 0 1 1 0 1 1 1 1 4.04

25-<30 30-<35 92 100 2 1 2 1 3 2 1 1 2 0 2 2 0 3.9

25-<30 30-<35 104 114 2 1 2 2 2 3 0 1 1 2 1 1 2 1 3.93

<20y 102 96 2 1 1 2 2 2 0 2 1 2 1 1 2 0 3.65

25-<30 25-<30 69 79 1 1 1 1 2 2 2 1 1 0 1 1 2 0 2.93

25-<30 25-<30 105 88 1 1 1 2 1 2 0 2 1 2 1 1 2 1 3.09

25-<30 25-<30 105 97 2 1 1 2 2 2 0 1 1 2 1 1 2.665

<20y 25-<30 98 91 2 2 2 2 2 3 2 2 1 1 1 1 2 1 3.15

25-<30 =35+ 101 101 2 1 2 2 2 1 0 1 1 2 1 1 2 1 3.59

25-<30 25-<30 109 85 1 1 1 1 3 2 0 1 1 1 1 1 3 0 3.95

25-<30 =35+ 94 101 1 1 2 3 2 2 0 1 1 2 1 2 2 0 3.7

20+yr <20y 118 102 2 2 2 3 2 3 0 2 1 2 1 1 2.955

25-<30 30-<35 98 2 1 2 1 1 3 0 1 1 0 0 1 2 1 3.13

<20y <20y 95 1 1 2 2 2 3 0 1 1 1 1 3.31

25-<30 25-<30 112 112 2 2 2 2 3 3 0 1 1 2 1 1 3 1 3.33

=35+ <20y 90 91 2 2 1 1 1 1 0 1 1 1 1 2 3 0 3.38

30-<35 =35+ 97 98 1 1 2 2 2 1 2 1 1 0 1 2 2 1 3.62

25-<30 30-<35 105 93 1 1 1 2 2 2 0 1 1 1 1 1 2 0 3.45

=35+ =35+ 102 2 1 1 1 3 1 0 1 1 1 1 1 3.36

<20y 25-<30 108 100 1 1 2 2 2 1 1 1 1 1 1 2 0 3.22

25-<30 30-<35 106 85 2 2 2 2 2 3 0 1 1 2 0 1 3 1 3.27

30-<35 25-<30 97 2 1 1 1 2 2 2 1 1 1 1 3.11

<20y <20y 87 90 2 1 2 2 2 2 0 1 1 2 1 1 2 1 3.92

25-<30 30-<35 95 76 2 2 1 2 1 1 0 2 1 2 2 3.5

30-<35 105 109 2 1 1 3 3 3 0 1 1 2 1 3.18

30-<35 =35+ 110 89 2 1 2 2 3 3 0 1 1 1 1 1 1 0 2.91

=35+ =35+ 91 70 2 2 1 3 2 2 0 1 1 1 1 1 1 1 2

<20y <20y 118 114 2 1 2 2 3 2 1 1 1 2 1 1 2 1 2.38

<20y 114 103 1 1 2 2 2 3 0 1 1 2 0 1 3.89

25-<30 112 90 1 2 1 2 2 2 2 1 1 1 1 2 0 3.72

<20y <20y 103 103 1 2 2 3 2 1 0 1 1 2 1 1 2 0 4.375

<20y 25-<30 105 112 1 1 1 1 2 2 2 1 2 2 0 1 2 0 2.9

20+yr <20y 92 85 2 2 2 2 3 1 1 2 1 2 1 1 3.46

20+yr <20y 100 96 1 2 2 2 2 3 2 1 1 0 1 0 2.72

<20y 104 95 2 1 2 1 0 2 1 1 1 2 2 0 1.57

20+yr <20y 107 109 1 1 2 3 2 2 2 1 2 1 1 2 0 4.09

25-<30 30-<35 112 92 2 1 2 2 3 3 0 1 1 2 1 1 2 1 3.23

30-<35 25-<30 94 84 2 1 1 1 1 1 0 1 1 1 3.45

<20y 30-<35 92 81 1 2 2 2 3 0 1 1 3.55

20+yr <20y 79 2 1 1 3 2 2 2 2 1 1 1 2 0 3.49

<20y <20y 99 102 2 1 2 1 3 0 1 1 2 1 2.7

<20y <20y 122 93 2 1 2 2 2 1 2 1 1 1 1 1 2 1 3.32

30-<35 =35+ 115 92 2 2 2 2 2 0 1 1 0 1 3.605

<20y 30-<35 104 90 1 1 1 3 3 3 0 1 1 2 1 2 0 3.56

25-<30 30-<35 120 88 2 1 1 2 1 2 0 1 1 0 1 2 2 1 3.19

25-<30 25-<30 88 2 1 2 1 1 1 0 1 1 0 0 1 2 0 2.51

20+yr 20+yr 95 97 2 2 1 1 2 2 2 1 1 0 1 1 2 3.15

25-<30 30-<35 89 88 1 1 1 1 2 2 2 1 3.855

<20y 25-<30 115 103 2 1 1 2 2 3 0 1 1 2 1 3 0 3.38

<20y 25-<30 86 2 2 2 2 2 2 1 2 1 3.66

20+yr <20y 107 98 2 1 2 2 2 2 2 1 1 1 1 2 2 0 3.21

<20y <20y 99 101 2 2 2 2 2 2 0 1 1 1 2 0 1.83

<20y <20y 97 94 1 1 2 3 2 2 0 1 1 2 1 1 1 0 3.76

<20y <20y 110 108 2 1 1 2 0 1 1 0 0 1 3 0 4.28

25-<30 25-<30 88 1 2 2 2 2 2 0 1 1 1 1 3.21

<20y <20y 107 89 2 2 1 3 2 3 1 1 1 2 0 1 2 0 3.65

<20y 25-<30 102 88 2 1 1 1 1 1 0 1 1 1 2 2 1 3.97

<20y <20y 93 86 2 2 2 1 2 1 1 1 1 0 1 1 2 1 1.71

25-<30 25-<30 105 1 2 1 3 3 2 1 1 1 2 1 1 2 0 3.575

25-<30 30-<35 94 96 2 1 1 1 2 0 2 1 1 1 1 2 1 3.12

<20y <20y 86 84 1 1 1 2 3 1 0 1 3.26

30-<35 30-<35 108 2 1 1 1 1 2 0 1 1 1 0 1 2 1 3.94

25-<30 25-<30 111 88 2 1 2 1 2 1 1 2 1 1 0 1 2 0 3.58

=35+ 30-<35 105 95 2 2 2 1 1 2 2 1 0 1 1 0 1.93

<20y <20y 109 95 1 2 2 2 2 2 1 1 1 1 1 1 2 1 3.88

30-<35 25-<30 111 117 2 2 2 3 3 2 0 1 1 2 1 1 2 0 4.31

<20y <20y 101 101 2 1 1 2 2 2 0 1 1 1 1 1 2 0 3.9

25-<30 30-<35 105 103 2 2 1 2 2 3 1 1 1 2 1 1 2 1 4.33

<20y 25-<30 106 87 2 1 2 1 1 2 0 1 1 1 1 1 0 3.18

25-<30 99 1 2 2 2 3 1 2 2 1 1 1 1 1 0 3.39

<20y 25-<30 90 113 2 1 2 2 2 2 2 1 1 1 1 1 2 1 4.065

<20y <20y 102 96 1 2 1 3 3 3 0 1 1 1 1 2 0 3.06

<20y 25-<30 109 1 2 1 3 3 1 0 1 1 2 0 1 2 0 3.66

25-<30 25-<30 118 108 1 1 2 2 3 2 2 2 1 2 1 1 3 1 3.04

<20y 25-<30 101 91 2 1 3 3 3 1 1 1 2 0 1 3 0 2.62

30-<35 30-<35 94 91 1 2 1 1 2 3 0 1 1 1 1 1 3.06

<20y <20y 103 101 1 2 2 1 1 1 2 2 1 1 0 1 2 0 3.03

<20y <20y 79 86 2 1 2 2 2 2 0 1 1 0 1 1 2 1 3.71

25-<30 30-<35 103 106 2 1 2 2 1 3 0 1 1 2 0 1 3 1 3.78

30-<35 30-<35 97 106 1 2 2 3 3 3 0 1 1 0 1 3 0 3.9

<20y 25-<30 120 100 2 2 2 1 2 2 0 1 1 1 1 2 2 1 3.4

25-<30 =35+ 105 104 2 1 2 3 3 3 0 1 1 1 1 1 2.27

25-<30 25-<30 104 81 2 1 2 2 2 1 1 1 1 1 0 1 0 3.53

30-<35 =35+ 95 99 2 1 2 2 1 2 0 1 1 1 0 2 3 0 3.57

30-<35 30-<35 125 124 2 2 1 2 3 3 0 1 1 2 1 1 3.73

25-<30 25-<30 105 100 1 2 2 3 2 3 0 1 1 2 1 1 2 0 3.12

30-<35 30-<35 96 100 2 2 1 2 2 3 0 1 1 2 1 1 3 0 3.27

25-<30 25-<30 110 2 2 1 2 2 1 0 1 1 1 1 4.11

25-<30 25-<30 123 88 2 1 1 2 3 2 0 1 1 1 2 2.66

<20y <20y 92 89 2 1 2 2 1 2 0 1 1 1 1 3.72

25-<30 25-<30 99 94 1 1 2 2 1 0 1 1 1 0 1 3 1 4.26

25-<30 30-<35 101 90 1 1 2 2 2 1 0 1 1 1 1 1 3.46

25-<30 25-<30 99 89 2 1 2 2 2 2 0 1 1 2 1 1 2 1 2.94

<20y 25-<30 108 103 1 2 2 2 2 1 0 1 1 2 1 1 2 0 3.14

<20y 25-<30 98 98 1 1 2 3 3 2 0 1 1 1 1 1 2 0 3.56

30-<35 30-<35 123 88 2 1 2 1 3 2 0 1 1 1 1 1 2 0 3.86

30-<35 30-<35 114 87 2 1 2 1 2 3 0 1 1 1 0 2 1 1 3.7

25-<30 =35+ 85 83 2 1 2 2 2 3 0 1 1 0 1 2 0 3.755

30-<35 =35+ 104 107 2 1 2 3 2 2 0 1 1 2 0 2 3 1 3.21

<20y <20y 89 93 2 2 1 2 1 2 0 1 1 0 1 1 2 1 3.5

25-<30 25-<30 123 95 2 2 1 2 2 1 2 1 2 0 0 2 2 1 3.78

25-<30 30-<35 107 98 2 1 3 2 2 0 2 1 1 0 1 2 1 3.74

25-<30 25-<30 110 97 2 1 2 2 2 3 1 1 1 2 0 1 3 1 3.145

<20y 30-<35 112 101 2 1 1 2 3 3 0 1 1 1 1 1 0 3.61

25-<30 25-<30 111 2 2 2 3 3 3 0 1 1 2 0 1 2 0 3.06

25-<30 30-<35 82 96 2 1 3 3 3 0 1 1 1 1 3.61

20+yr <20y 90 1 1 1 1 1 1 2 1 1 0 1 3.13

25-<30 25-<30 123 112 2 1 1 2 2 2 0 1 1 2 0 1 2 0 4

=35+ 25-<30 88 97 1 1 2 2 0 1 1 0 1 2.62

25-<30 30-<35 103 94 2 2 2 1 2 3 2 1 1 1 0 1 3 0 3.265

25-<30 25-<30 108 108 2 1 1 3 3 1 0 2 1 2 1 1 2 0 3.45

=35+ 30-<35 112 93 2 1 1 1 2 0 1 1 1 0 1 2 1 4.08

<20y 25-<30 104 98 1 1 2 2 2 3 0 1 1 1 0 1 2 0 3.91

30-<35 25-<30 114 112 2 1 1 3 3 3 0 1 1 2 0 1 2 1 4.2

<20y 25-<30 111 97 2 1 1 2 3 3 0 1 1 1 0 1 2 0 4

30-<35 30-<35 108 99 2 2 2 2 1 2 0 1 1 0 1 2 3 0 2.88

25-<30 25-<30 111 90 1 1 2 2 1 2 0 1 1 2 0 2 3 0 3.93

<20y <20y 114 92 2 2 2 2 2 0 2 1 1 1 2.425

<20y 20+yr 107 1 2 2 2 2 0 2 1 0 1 1 2 0 3.11

<20y <20y 102 94 1 2 1 2 2 1 2 2 1 0 1 3.18

<20y =35+ 110 97 2 1 2 3 3 3 0 1 1 2 0 1 3 0 4.02

25-<30 25-<30 114 98 2 2 1 2 2 3 0 1 1 1 1 1 2 0 4.68

<20y <20y 99 88 2 1 2 2 3 0 1 1 1 1 1 2 0 3.91

<20y 25-<30 109 100 2 1 1 2 2 3 0 1 1 1 0 1 3 0 2.44

<20y 25-<30 99 98 2 2 2 2 1 2 0 1 1 1 0 2 2 1 3.33

<20y 25-<30 95 103 1 1 2 2 2 3 0 2 1 1 0 1 2 1 3.13

<20y <20y 109 93 2 1 2 2 2 2 1 1 1 1 0 2 2 0 3.65

<20y <20y 92 90 1 1 1 2 2 1 0 1 1 1 1 1 1 3.42

25-<30 30-<35 95 89 2 2 1 2 2 3 0 1 1 2 1 1 2 1 2.78

<20y <20y 100 91 2 1 2 2 2 1 2 1 1 0 1 2 2 1 2.73

<20y 30-<35 88 85 2 1 1 1 2 3 0 2 1 2 1 2 0 3.28

30-<35 25-<30 104 1 2 2 2 2 0 1 1 1 1 1 3 0 4.03

25-<30 25-<30 115 119 2 1 2 2 3 3 0 1 1 2 1 1 3 0 3.45

25-<30 30-<35 97 84 2 2 3 2 1 1 1 1 1 1 1 1 0 2.99

20+yr 79 89 2 2 1 2 1 3 0 1 1 1 0 1 2 1 4.02

25-<30 30-<35 89 108 1 2 1 3 2 3 1 1 1 2 1 1 2 1 3.76

25-<30 25-<30 109 2 1 1 3 2 2 0 1 1 2 0 1 2 0 3.88

20+yr <20y 86 97 2 2 2 1 1 1 2 1 1 1 0 2 2 0 3.9

=35+ 30-<35 107 93 2 1 2 1 3 3 2 1 1 2 1 2 0 2.865

<20y <20y 83 86 2 2 2 2 3 0 1 1 2 0 2 1 0 3.36

<20y 25-<30 97 95 2 1 2 2 2 2 2 2 1 0 0 1 0 2.88

20+yr <20y 99 2 2 2 2 3 0 1 1 0 1 3.64

25-<30 30-<35 89 79 2 2 2 2 1 1 1 1 2 0 2 2 1 3.235

<20y 30-<35 118 103 2 2 1 2 2 3 0 1 1 1 1 2 2 1 2.87

<20y <20y 94 88 2 1 1 2 2 1 2 1 1 1 0 1 2 0 2.9

=35+ 115 79 2 2 2 1 2 0 1 0 2.92

25-<30 30-<35 115 2 1 2 2 2 3 0 1 1 1 1 1 0 2.98

30-<35 =35+ 110 91 2 2 2 3 3 1 0 1 1 1 0 2 2 1 2.72

<20y <20y 111 97 2 2 2 2 2 3 0 1 1 2 0 1 2 1 3.94

<20y 25-<30 103 109 2 2 1 1 2 3 0 1 1 0 0 1 2 0 4.02

25-<30 30-<35 103 112 1 2 2 2 3 3 0 1 1 2 1 1 2 1 3.67

=35+ 30-<35 96 107 2 2 2 2 2 3 1 2 1 2 1 1 2 1 3.42

<20y <20y 103 91 1 1 2 2 1 0 1 1 0 1 3.8

<20y 25-<30 85 82 2 1 2 3 1 3 0 1 1 1 0 1 2 0 3.8

25-<30 30-<35 115 100 2 2 2 3 3 3 0 1 1 2 1 1 3 0 3.6

20+yr <20y 95 92 2 2 2 1 1 1 1 1 1 0 0 1 2 1 2.63

25-<30 25-<30 107 104 2 1 1 2 2 2 1 2 1 2 0 2 2 0 3.59

25-<30 30-<35 105 112 2 1 2 2 2 3 2 2 1 0 0 1 2 0 3.085

<20y <20y 103 107 2 2 2 2 2 2 0 1 1 1 0 1 2.58

25-<30 25-<30 100 90 2 1 1 2 1 3 0 1 1 0 0 1 2 1 3.98

<20y 25-<30 108 85 2 1 1 2 2 3 1 1 1 0 0 1 2 0 3.225

25-<30 30-<35 107 112 2 2 2 3 1 3 1 1 1 2 0 1 2 0 3.48

30-<35 30-<35 106 1 2 2 2 0 1 1 2 1 3.18

<20y <20y 112 103 2 2 2 2 2 3 0 1 1 2 1 3.515

<20y <20y 92 93 2 1 1 2 2 2 2 1 1 0 0 2 2 1 3.745

25-<30 114 110 2 2 2 2 2 2 0 1 1 0 0 1 3.3

20+yr <20y 90 2 2 1 2 2 0 1 1 1 1 2 2 1 2.96

<20y <20y 127 119 2 2 1 3 3 3 0 1 1 1 0 1 2 1 3.92

30-<35 30-<35 108 89 2 1 2 2 2 2 1 2 1 1 1 2 3 1 3.39

20+yr 20+yr 92 85 1 2 1 2 2 1 0 1 1 1 1 3.61

<20y <20y 73 93 2 1 1 1 1 2 0 1 1 0 0 1 2 1 3.585

<20y 25-<30 102 94 2 2 1 2 2 2 0 1 1 2 0 1 2 0 3.82

25-<30 30-<35 102 96 1 1 2 2 1 2 1 1 1 0 2 0 3.21

25-<30 30-<35 123 2 1 1 2 2 3 0 1 1 0 0 1 2 0 3.74

<20y <20y 103 104 2 1 2 2 1 2 0 1 1 2 0 1 2 0 3.43

25-<30 25-<30 99 95 2 1 2 3 2 3 0 1 1 1 0 1 3 0 3.64

<20y 30-<35 103 1 2 1 2 2 3 2 1 1 1 0 1 3 0 3.29

<20y 25-<30 93 80 2 2 1 2 2 2 1 2 1 1 1 1 2 1 3.48

<20y <20y 115 95 2 1 2 2 2 0 1 1 2 1 1 2 0 3.795

25-<30 25-<30 115 97 2 1 1 3 3 2 0 1 1 2 1 1 2 0 3.09

<20y =35+ 103 88 1 1 2 3 1 3 1 2 1 2 0 1 1 3.5

<20y 30-<35 94 87 1 1 1 2 3 2 0 1 1 1 0 1 2 1 3.92

<20y 30-<35 108 94 2 2 1 2 1 3 0 1 1 1 0 1 2 0 4.42

20+yr 90 105 1 2 1 2 1 1 1 1 2 0 1 0 3.77

25-<30 25-<30 96 93 2 1 1 2 2 2 0 1 1 2 0 1 2 1 4.42

25-<30 25-<30 103 86 2 1 1 2 2 2 0 2 1 1 0 2 2 0 3.56

25-<30 25-<30 103 110 2 1 1 2 3 3 1 1 1 1 1 1 2 1 3.47

30-<35 30-<35 111 93 2 1 2 1 3 2 0 1 1 2 0 1 2 0 3.29

25-<30 25-<30 97 97 2 1 1 2 3 1 1 1 1 0 1 3 0 3.5

25-<30 30-<35 105 95 2 1 1 3 3 2 1 1 1 2 0 1 3 0 2.93

20+yr 20+yr 100 101 1 1 1 1 2 1 2 1 1 1 1 1 3 0 3.7

25-<30 25-<30 101 1 1 2 2 2 1 1 1 1 2 0 1 3 0 3.15

20+yr 20+yr 94 87 2 2 2 1 2 1 1 1 1 1 0 2 2 0 3.41

<20y 25-<30 92 90 2 1 2 2 2 2 0 2 1 1 1 3.37

<20y 25-<30 94 96 2 1 1 2 1 3 2 1 1 1 0 1 3 1 3.53

<20y <20y 94 103 2 1 1 2 3 1 1 1 1 2 0 1 2 1 4

=35+ =35+ 118 117 1 1 1 3 3 1 0 1 1 2 1 1 3.49

25-<30 25-<30 90 110 2 2 1 2 2 2 0 2 1 1 0 2 2 1 3.59

20+yr <20y 103 128 2 2 1 3 2 2 0 1 1 0 0 1 2 1 2.42

25-<30 25-<30 112 94 2 1 1 2 2 3 0 1 1 2 0 2 1 0 3.71

<20y 25-<30 108 91 2 1 1 2 2 3 1 0 0 2 2 1 4.19

<20y <20y 105 1 2 2 2 2 3 0 1 1 2 0 1 2 0 3.76

25-<30 25-<30 106 100 2 1 2 2 3 1 2 1 1 0 1 2 0 3.13

<20y <20y 102 85 2 1 2 2 2 3 0 1 1 1 1 3.34

25-<30 30-<35 82 84 2 1 1 1 1 2 0 1 1 0 0 1 3.22

25-<30 25-<30 115 106 2 1 1 3 3 2 0 1 1 1 0 1 1 3.61

<20y <20y 89 100 1 1 2 2 2 2 1 1 1 0 1 3 0 3

20+yr <20y 94 105 1 2 1 2 2 2 1 1 1 2 0 1 2 0 3.71

25-<30 30-<35 88 104 2 2 2 3 2 2 1 1 2 0 2 2 1 2.67

25-<30 25-<30 106 100 2 1 1 2 2 2 1 2 0 2 2 1 4.1

25-<30 30-<35 86 87 2 1 2 1 2 2 0 2 1 0 1 3.52

<20y 25-<30 90 93 2 1 2 1 2 3 0 2 1 2 0 1 2 1 3.62

25-<30 92 96 1 2 1 1 1 0 1 1 2 1 3.4

25-<30 30-<35 123 112 1 1 1 3 2 3 0 1 1 2 1 1 2 0 2.89

25-<30 25-<30 92 98 2 2 1 2 2 3 1 1 1 2 0 1 2 1 3.76

25-<30 <20y 92 94 2 2 2 2 2 0 2 1 1 0 1 2 0 3.3

<20y 103 86 1 2 1 2 2 2 1 1 0 1 2 0 3.26

25-<30 30-<35 122 98 2 1 2 3 3 2 0 1 1 2 0 1 2 1 3.31

25-<30 30-<35 93 87 2 1 2 2 2 2 0 1 1 2 1 3.045

30-<35 <20y 86 84 2 1 1 1 1 2 2 2 1 2 1 1 0 3.21

20+yr 86 92 1 2 2 2 3 0 1 1 0 0 1 1 2.98

25-<30 30-<35 114 89 2 2 1 1 2 2 1 1 0 1 2 0 1.81

<20y 25-<30 111 112 1 1 1 3 1 1 0 1 1 2 0 1 2 1 3.24

25-<30 30-<35 107 96 2 1 1 2 2 2 2 2 1 1 0 2 2 1 3.58

30-<35 =35+ 106 101 2 2 1 2 1 3 2 1 1 2 1 3.35

25-<30 25-<30 106 103 2 1 2 2 2 3 2 1 1 2 0 1 0 3.11

30-<35 =35+ 97 1 1 2 2 2 1 2 1 0 0 1 2 1 3

25-<30 30-<35 102 90 2 1 1 2 1 2 0 1 1 2 0 1 3 1 2.645

<20y <20y 115 98 2 1 2 3 3 2 0 1 1 2 1 1 3.38

25-<30 30-<35 109 104 2 1 1 2 2 2 2 1 1 1 0 1 2 0 2.16

25-<30 25-<30 110 114 2 1 1 3 3 3 0 1 1 2 0 1 3 1 3.09

25-<30 25-<30 115 95 2 1 1 1 2 3 0 1 1 2 0 1 3.76

<20y <20y 120 119 2 1 3 2 2 0 1 1 2 1 2.88

20+yr 25-<30 80 102 2 2 2 2 2 1 1 1 1 0 1 3 1 3.045

<20y <20y 86 101 2 1 2 1 1 2 2 1 1 0 0 1 2 0 2.89

25-<30 =35+ 105 1 2 3 1 3 0 1 1 2 2 3.76

<20y 100 93 2 1 2 2 2 2 1 1 1 0 1 3 0 3.72

<20y 25-<30 92 86 2 2 2 2 3 3 0 1 1 2 0 1 2 0 3.25

25-<30 25-<30 100 106 2 1 2 2 3 3 0 1 1 1 1 2 1 3.51

=35+ 30-<35 97 102 1 1 2 2 2 3 0 1 1 2 0 1 3 1 3.29

<20y <20y 84 84 1 1 2 2 1 2 2 1 1 1 0 1 2 1 2.84

25-<30 30-<35 114 1 2 2 3 2 3 0 1 1 0 0 2 1 0 3.48

25-<30 30-<35 110 83 2 1 1 1 3 3 0 1 1 0 1 3.39

30-<35 =35+ 110 114 2 2 1 3 3 3 1 2 1 2 1 1 2 0 3.11

25-<30 25-<30 107 122 1 1 1 2 2 2 2 2 1 1 1 3.45

25-<30 25-<30 105 1 2 1 1 2 2 0 1 1 2 0 1 1 3.16

25-<30 25-<30 93 1 2 2 2 2 1 2 1 1 0 0 1 2 0 3.25

<20y =35+ 110 117 1 2 2 3 2 3 0 1 1 2 1 4.21

30-<35 =35+ 88 1 2 2 2 1 2 1 1 1 1 0 1 1 2.68

25-<30 25-<30 132 117 1 2 2 3 3 2 0 1 2 2 0 2 2 0 3.66

<20y <20y 83 95 2 1 1 2 2 2 1 1 1 2 0 1 2 0 3.66

=35+ =35+ 110 61 2 1 2 1 2 1 0 1 1 0 1 1 3 1 3.53

=35+ =35+ 109 89 2 2 1 2 2 3 0 1 1 2 0 1 2 1 3.17

20+yr <20y 109 105 1 1 3 2 2 2 1 1 1 0 1 2 0 2.61

=35+ =35+ 91 1 1 2 2 3 0 1 1 0 0 1 2 0 4.03

<20y <20y 118 102 1 1 1 2 2 3 0 1 1 2 0 1 2 0 3.925

25-<30 =35+ 109 106 2 1 2 3 2 1 2 1 1 1 0 1 2 0 2.8

<20y 25-<30 90 92 2 1 1 2 2 2 0 1 1 0 0 1 2 0 3.72

=35+ =35+ 103 112 2 2 2 2 3 2 2 2 1 1 0 1 2 0 2.98

<20y 25-<30 82 82 2 1 2 2 2 3 0 2 1 2 0 1 2.86

<20y <20y 86 90 2 2 1 2 2 2 2 2 1 0 0 1 2 1 3.49

30-<35 =35+ 109 1 2 3 1 1 0 2 1 1 0 1 2 1 3.04

25-<30 25-<30 111 112 2 2 1 3 3 3 0 1 1 2 0 1 3 0 3.05

<20y 25-<30 105 1 1 1 2 3 1 0 1 1 0 1 3.25

<20y 25-<30 117 101 2 1 2 3 2 2 0 1 1 2 0 1 0 4.41

=35+ =35+ 94 1 2 1 3 3 3 0 1 1 0 0 1 2 1 3.47

=35+ 30-<35 111 117 2 1 1 2 2 3 0 1 1 2 0 1 3 1 3.033

<20y 25-<30 101 98 1 1 1 2 2 3 0 1 1 2 0 1 2 0 3.425

25-<30 25-<30 98 94 2 1 2 2 2 1 0 2 1 2 0 2 2 1 2.42

=35+ =35+ 94 102 2 1 1 2 2 3 0 1 1 1 0 1 3 0 3.595

25-<30 25-<30 92 96 2 2 1 2 2 1 0 1 1 0 0 2 2 1 2.42

=35+ =35+ 108 120 2 1 1 3 3 2 1 2 1 2 0 1 3 1 3.67

25-<30 25-<30 81 77 2 2 1 1 2 1 0 1 1 1 0 1 2 1 4.25

<20y 25-<30 103 104 1 2 1 2 2 1 0 1 1 0 0 2 3 0 4.21

25-<30 25-<30 104 117 2 2 1 2 2 2 0 1 1 1 0 1 2 0 3.74

<20y 25-<30 115 103 2 2 1 3 2 2 2 1 2 2 0 1 2 0 4.485

30-<35 30-<35 104 103 2 1 2 2 3 3 1 2 0 1 3 0 3.61

25-<30 30-<35 97 93 2 1 1 2 1 2 2 1 1 1 1 3.89

25-<30 25-<30 110 1 1 1 3 3 0 1 1 0 1 3.06

30-<35 30-<35 89 90 2 1 2 2 2 2 1 1 1 2 0 1 3 1 3.21

<20y <20y 111 85 2 2 1 2 2 1 2 1 1 1 0 1 2 1 3.75

25-<30 30-<35 103 108 1 2 2 2 2 1 0 1 1 1 1 3.62

<20y <20y 110 87 2 1 1 2 2 2 0 1 1 1 0 1 3.28

20+yr 100 99 1 2 1 2 2 2 2 1 1 1 2 3.5

30-<35 30-<35 109 97 2 1 2 2 2 3 1 1 1 2 0 1 2 1 3.495

<20y 30-<35 88 84 2 1 1 2 2 3 0 1 1 2 0 1 2 1 4.65

<20y 114 101 1 2 2 3 2 1 1 1 1 2 1 1 2 0 3.245

25-<30 25-<30 120 2 1 1 3 2 2 0 1 1 2 0 1 2 0 3.2

25-<30 25-<30 123 112 2 2 1 2 2 3 0 1 1 2 0 2 2 0 3.37

25-<30 30-<35 102 93 2 1 2 2 2 3 1 1 1 1 0 1 2 1 3.105

30-<35 25-<30 109 93 1 1 1 2 1 2 0 1 1 2 0 1 2 0 3.52

25-<30 30-<35 114 97 2 2 2 2 3 3 0 1 1 2 0 2 2 0 3

=35+ =35+ 123 1 1 1 1 1 2 0 1 1 2 0 1 2 1 3.905

25-<30 30-<35 101 96 2 2 1 2 2 2 0 1 1 2 0 2 2 0 4.23

30-<35 25-<30 94 86 1 2 2 1 3 0 2 1 0 0 1 0 3.35

<20y 25-<30 105 99 1 2 2 3 3 3 0 1 1 2 0 1 2 0 3.29

20+yr 88 95 2 2 1 3 0 1 2 0 0 1 2 0 2.09

=35+ =35+ 112 78 2 1 2 2 1 0 1 1 2 0 2 2 1 3.545

20+yr <20y 108 90 2 2 1 2 2 1 2 1 1 1 0 1 2 0 3.67

<20y 25-<30 111 98 1 2 1 2 2 2 0 1 1 1 0 2 2.825

<20y 25-<30 96 91 2 2 1 2 1 2 1 1 1 1 1 3.84

25-<30 25-<30 95 82 2 1 3 3 0 1 1 1 0 1 2 1 2.99

<20y 25-<30 93 96 1 1 1 2 1 2 0 1 1 0 3.4

25-<30 25-<30 81 87 2 1 2 2 2 2 2 2 1 0 0 1 2 1 3.18

<20y 25-<30 108 94 2 1 1 2 3 2 0 1 1 2 0 1 2 1 4.23

<20y 25-<30 102 92 2 1 2 2 3 3 0 1 1 2 1 3.92

<20y 25-<30 101 101 1 1 1 2 1 3 0 1 1 2 0 1 2 1 3.63

25-<30 30-<35 110 125 2 1 2 2 3 0 1 1 2 0 1 2 0 3.47

25-<30 25-<30 118 2 1 2 2 2 0 1 1 0 0 1 2 1 3.26

<20y <20y 117 98 1 1 1 2 2 3 0 2 1 2 0 1 2 0 3.66

30-<35 30-<35 118 96 1 1 2 2 2 1 0 1 1 2 1 1 3 0 3.81

30-<35 30-<35 115 98 2 2 1 3 3 2 0 1 1 2 0 2 2 0 3.28

<20y 89 76 2 2 3 1 1 1 2 1 0 1 1 3.18

20+yr <20y 102 102 1 2 1 2 1 1 0 2 1 1 0 1 2 0 3.21

25-<30 110 91 2 2 2 3 3 3 0 2 1 2 0 1 3 0 3.46

<20y 25-<30 85 1 2 2 2 1 1 1 1 2 1 3.565

30-<35 30-<35 120 125 2 1 2 3 3 3 0 1 1 2 0 1 2 1 4.325

<20y <20y 97 100 2 2 1 1 2 2 1 1 1 0 1 3.19

<20y 25-<30 97 95 1 1 2 2 1 1 0 1 1 2 0 1 3 0 3.06

<20y 25-<30 95 1 2 2 2 2 3 0 1 1 2 0 1 2 1 2.82

25-<30 30-<35 110 91 2 1 2 2 3 3 0 1 1 2 0 1 2 1 3.495

20+yr 20+yr 95 87 1 2 1 2 2 2 2 1 0 0 1 2 1 3.53

<20y <20y 90 90 2 1 2 2 2 3 0 2 1 2 0 1 2 1 3.21

<20y 25-<30 114 100 2 1 2 2 2 1 0 1 1 2 0 2 2 0 3.56

25-<30 25-<30 101 94 2 2 1 2 2 2 0 1 1 2 0 1 0 2.81

25-<30 30-<35 112 92 2 2 1 2 2 2 0 1 1 1 0 1 3 1 3.39

20+yr <20y 103 117 2 1 2 2 1 0 1 1 1 0 1 2 0 4.4

25-<30 25-<30 94 90 2 1 2 2 2 2 0 1 1 2 0 1 2 0 3.57

<20y 25-<30 93 94 2 2 1 2 3 2 0 1 1 2 0 1 2 0 3.34

30-<35 30-<35 118 1 1 2 3 3 3 0 1 1 2 0 1 3 0 3.64

30-<35 =35+ 102 79 2 1 2 2 3 2 1 1 0 0 2 1 0 2.69

30-<35 30-<35 108 103 2 1 1 2 2 3 0 2 1 1 0 1 2 0 4.17

<20y 30-<35 90 80 2 2 1 2 3 2 1 2 0 2 3.44

<20y <20y 115 100 2 1 2 2 1 3 2 1 1 2 0 1 2 1 4.01

20+yr <20y 98 1 2 1 2 1 1 2 1 2 0 1 2 0 3.46

<20y 109 97 1 2 1 2 2 1 1 1 0 0 1 2 1 3.27

25-<30 30-<35 100 96 2 2 1 2 2 3 2 1 1 1 0 1 3 0 3.31

30-<35 30-<35 106 100 1 1 2 2 3 1 0 1 1 2 0 1 3 0 3.85

25-<30 25-<30 105 97 1 1 2 2 2 2 0 2 1 1 0 1 2 0 3.79

30-<35 30-<35 111 107 2 1 3 2 3 0 1 1 1 0 1 3 0 3.61

20+yr 20+yr 83 98 1 2 1 2 2 1 1 1 1 1 0 1 0 3.15

<20y <20y 107 81 2 2 1 2 1 3 0 1 1 1 0 1 2 1 3.58

25-<30 30-<35 112 100 2 1 1 2 2 3 0 2 1 1 0 1 1 0 3.44

<20y <20y 106 101 2 1 1 2 2 2 2 1 1 0 2 0 1.886

25-<30 25-<30 106 91 2 1 2 2 1 3 2 1 1 1 1 1 2 0 3.75

=35+ 25-<30 90 87 1 1 2 1 2 3 0 1 1 0 0 1 2 0 4.23

30-<35 30-<35 100 84 2 1 2 3 3 2 1 1 0 1 1 2 1 3.49

<20y <20y 104 88 2 2 2 2 3 3 1 1 1 1 0 1 3 1 3.97

25-<30 25-<30 118 110 2 1 2 3 3 2 0 2 1 2 0 2 2 1 3.08

25-<30 25-<30 88 77 2 2 2 2 2 2 1 2 1 2 0 1 1 3.935

25-<30 25-<30 109 81 1 2 1 2 2 2 2 1 1 2 0 1 2 1 3.298

30-<35 30-<35 102 112 2 1 2 2 2 3 2 2 1 2 0 1 0 2.48

25-<30 30-<35 97 88 2 1 1 2 2 2 0 1 1 2 0 2 4.01

25-<30 30-<35 96 96 2 1 3 3 2 2 1 2 0 1 1 2 1 2.93

20+yr <20y 102 87 1 1 2 2 1 1 0 2 1 2 1 3.775

30-<35 30-<35 89 93 2 2 2 3 2 1 0 1 1 2 0 1 2 0 2.415

30-<35 =35+ 109 88 2 1 1 1 1 3 0 1 1 1 0 1 1 0 3.8

<20y 25-<30 99 98 2 1 1 2 2 3 0 2 1 2 0 2 2 0 3.8

25-<30 30-<35 111 94 2 2 1 2 2 3 0 1 1 1 0 1 3.33

30-<35 30-<35 120 105 2 2 1 3 3 3 0 1 1 2 1 1 2 1 4.415

<20y <20y 97 101 2 1 2 3 2 3 0 1 1 1 1 2.53

25-<30 =35+ 104 101 2 1 1 2 3 1 2 1 1 1 0 1 2 1 3.33

<20y <20y 114 110 2 2 2 2 2 1 2 1 1 1 1 1 2 0 3.37

20+yr <20y 97 92 1 2 2 2 2 2 0 1 1 2 1 1 2 3.26

<20y <20y 99 97 1 2 2 2 2 3 1 1 1 1 0 1 1 0 3.25

<20y 25-<30 103 98 2 1 1 2 2 3 0 1 1 1 1 4.1

30-<35 =35+ 114 97 2 1 2 1 2 2 2 1 2 0 1 2 0 2.79

25-<30 30-<35 102 98 2 1 1 2 1 3 0 1 1 2 0 1 2 1 3.3

30-<35 25-<30 97 107 2 2 2 2 2 3 2 2 1 0 0 1 2 1 3.85

30-<35 30-<35 105 93 2 2 2 2 2 2 0 1 2 1 0 2 2 0 3.54

<20y <20y 89 88 2 2 1 2 1 2 1 1 0 1 3.15

<20y 25-<30 99 103 2 1 1 2 2 3 0 1 1 2 0 2 2 1 4.59

25-<30 30-<35 106 93 2 2 2 3 2 2 0 2 1 2 0 2 2 1 3.13

25-<30 30-<35 94 97 2 2 2 2 2 3 2 2 1 1 0 1 2 1 3.01

30-<35 25-<30 118 117 2 2 1 3 3 2 1 1 1 2 0 1 3 0 2.69

30-<35 30-<35 90 1 1 1 2 2 2 0 2 1 2 0 1 2 1 3.72

<20y <20y 92 87 2 1 2 2 2 2 0 1 1 0 2 2.96

30-<35 30-<35 111 117 2 1 1 2 2 0 1 1 2 0 1 2 1 3.74

<20y =35+ 109 1 2 1 2 3 2 0 1 1 0 1 1 2 0 3.53

<20y 25-<30 99 89 2 2 2 2 2 3 0 1 1 1 1 1 2 0 4

25-<30 =35+ 111 95 1 2 1 1 2 1 0 1 1 1 0 1 2 1 3.9

25-<30 25-<30 79 80 1 2 1 2 3 1 0 1 1 1 1 1 1 1 4.36

<20y <20y 114 97 1 1 2 2 2 2 0 1 1 2 0 2 2 0 3.7

<20y 25-<30 118 2 1 2 2 2 1 2 1 1 2 0 1 2 0 3.27

<20y <20y 101 113 2 2 2 2 1 3 1 1 1 1 0 1 2 0 4.06

20+yr <20y 99 94 2 2 2 2 1 0 1 1 0 0 2 2 0 3.3

<20y 25-<30 112 93 2 1 1 2 2 2 0 1 1 2 0 1 2 1 3.45

<20y <20y 108 108 2 1 1 1 2 3 0 1 1 1 0 1 2 0 3.9

<20y <20y 125 90 2 2 1 2 2 1 2 1 1 0 2 1 2.88

<20y 25-<30 98 111 2 1 2 2 2 2 1 1 2 0 2 0 3.01

<20y 25-<30 110 106 2 2 2 2 2 2 2 2 1 0 0 1 3 1 3.77

25-<30 25-<30 100 108 1 1 1 3 3 3 0 1 1 1 1 1 2 0 3.96

25-<30 25-<30 112 100 2 1 2 2 2 2 0 1 1 2 0 1 2 1 3.47

30-<35 30-<35 101 95 1 1 2 1 2 2 2 2 1 0 1 1 0 4.57

30-<35 30-<35 108 88 1 1 2 2 2 1 0 1 1 2 0 1 1 3.06

30-<35 30-<35 102 90 1 1 1 2 2 2 0 1 1 2 0 1 3 0 5.39

<20y <20y 88 94 2 2 1 3 3 1 0 1 1 1 0 1 2 0 3.56

<20y <20y 101 2 2 2 2 2 3 0 1 1 1 0 1 2 3.49

30-<35 30-<35 90 109 2 2 2 2 2 1 2 2 1 2 1 1 2 1 2.7

<20y <20y 101 2 2 1 2 2 3 0 2 1 1 2 3.16

<20y <20y 108 92 1 1 2 3 2 3 1 1 1 1 1 3.69

25-<30 30-<35 117 91 1 2 1 2 2 1 2 1 1 1 1 1 2 1 3.615

25-<30 =35+ 115 100 2 1 1 3 3 3 0 1 1 2 0 1 2 0 4.29

25-<30 25-<30 108 114 2 2 2 2 2 3 2 1 1 2 0 1 2 0 2.66

30-<35 25-<30 105 104 2 1 2 2 2 2 1 1 1 2 1 1 2 0 3.19

20+yr <20y 101 92 1 2 1 2 2 3 0 1 1 2 0 1 2 0 3.63

25-<30 30-<35 112 103 2 2 1 2 3 3 0 1 1 1 0 1 2 0 1.495

25-<30 97 1 1 2 1 0 1 2 0 3.9

25-<30 30-<35 104 68 2 1 2 3 2 2 0 1 1 1 1 1 3 1 3.245

20+yr 100 102 1 2 1 2 1 0 1 1 1 0 1 2 0 3.27

25-<30 =35+ 112 110 2 1 1 1 1 2 0 1 1 2 1 1 3 1 3.78

<20y <20y 110 98 1 1 1 2 3 2 0 1 1 2 1 1 2 0 4.13

20+yr <20y 112 95 1 2 2 2 3 1 2 1 1 3.61

<20y =35+ 101 89 2 1 1 1 1 2 2 1 1 1 0 1 1 3.1

20+yr <20y 102 93 1 1 1 3 2 2 2 2 1 1 0 1 2 0 3.37

<20y <20y 99 1 2 2 2 1 0 1 1 1 1 1 2 1 3.03

25-<30 25-<30 102 104 1 2 2 2 3 0 1 1 1 0 1 2 0 3.51

25-<30 30-<35 109 108 2 2 2 1 1 0 1 1 1 0 1 1 0 2.45

20+yr <20y 120 98 2 2 1 2 3 2 0 1 1 2 1 1 0 3.74

25-<30 25-<30 101 92 2 1 2 2 3 3 1 1 1 1 1 1 3 1 2.63

<20y <20y 88 88 2 1 1 2 1 1 0 1 1 2 0 1 2 0 3.46

<20y 25-<30 101 96 1 1 2 2 1 3 0 1 1 1 0 1 2 0 2.99

<20y 25-<30 125 2 1 2 3 2 3 0 1 1 2 0 1 3 0 3.715

<20y 25-<30 105 82 2 1 2 3 2 2 0 1 1 2 0 1 1 3.9

<20y 25-<30 98 93 1 2 1 1 2 1 2 1 2 1 0 1 2 0 2.85

25-<30 30-<35 94 2 1 1 2 2 2 0 1 1 1 1 1 4.19

25-<30 30-<35 102 49 2 2 1 3 2 1 0 1 1 0 0 2 3 1 2.98

=35+ =35+ 104 93 1 1 2 1 2 3 0 1 1 0 0 1 3 0 3.5

=35+ =35+ 120 120 2 2 2 3 3 3 1 1 1 2 1 1 2 0 3.98

20+yr <20y 120 105 1 2 2 1 2 3 1 1 0 0 1 3 0 3.715

20+yr 25-<30 97 1 1 1 2 2 2 2 1 1 1 1 3.92

30-<35 25-<30 117 117 2 1 1 2 2 3 2 1 1 1 0 1 2 0 2.99

25-<30 30-<35 97 86 2 1 1 2 2 3 0 2 1 2 0 1 2 0 3.6

20+yr <20y 112 99 2 2 2 2 2 0 1 1 1 1 1 2 0 3.21

30-<35 25-<30 110 1 2 1 3 2 2 0 1 1 2 0 1 3 0 3.15

30-<35 25-<30 137 98 1 1 2 3 2 3 0 1 1 2 1 1 3 0 4.4

20+yr 25-<30 96 82 2 2 2 2 1 3 0 1 1 1 1 3.53

<20y <20y 107 119 2 1 2 3 1 1 1 1 1 1 1 3.29

20+yr <20y 115 98 2 2 2 1 2 1 1 1 1 3.47

=35+ =35+ 115 97 2 1 1 2 1 2 0 1 1 0 3 1 2.97

<20y 25-<30 115 2 1 3 2 2 2 1 1 1 0 1 2 0 3.26

<20y 25-<30 103 106 2 2 1 2 2 3 0 2 1 1 0 1 1 0 3.67

25-<30 30-<35 102 87 2 1 2 2 2 2 2 2 1 0 0 1 0 2.93

20+yr 25-<30 105 93 2 1 1 2 2 2 1 2 1 1 2 0 3.73

25-<30 <20y 93 97 2 2 2 3 3 1 2 1 1 2 0 1 2 1 3.04

<20y <20y 109 104 2 1 2 2 3 2 0 1 1 1 0 1 2.37

<20y 30-<35 92 88 2 2 2 2 2 3 2 1 1 2 0 2 0 2.98

30-<35 25-<30 97 107 1 2 2 3 2 2 0 1 2 2 1 1 2 0 3.32

25-<30 30-<35 100 86 2 1 1 1 2 2 1 2 1 1 0 1 0 3.78

25-<30 <20y 98 103 2 1 2 3 3 2 0 2 1 2 0 1 2 1 3.58

30-<35 <20y 123 107 2 2 2 3 2 2 0 1 1 2 1 3.18

<20y 25-<30 112 101 2 1 3 2 2 2 1 1 2 0 1 2 0 3.52

25-<30 20+yr 97 80 2 1 2 2 1 1 2 2 1 0 1 3.69

<20y <20y 95 87 1 1 2 2 2 2 0 1 1 1 1 1 0 3.78

20+yr 20+yr 110 117 1 1 1 2 2 1 1 1 1 0 1 2.96

25-<30 25-<30 111 112 1 1 2 3 3 3 0 1 1 2 1 2.81

<20y 25-<30 95 101 1 2 1 2 2 2 1 1 1 1 0 1 2 1 4.12

25-<30 25-<30 112 100 2 1 1 2 2 3 0 2 1 0 0 1 2 1 3.41

25-<30 106 108 2 2 1 2 2 2 0 1 1 2 0 1 1 0 3.69

<20y <20y 108 97 2 2 1 2 2 3 2 1 1 2 1 3.26

=35+ =35+ 107 2 1 2 1 2 0 1 0 0 1 1 0 2.16

30-<35 30-<35 108 110 2 1 2 2 3 3 0 1 1 2 0 1 2 1 3.06

30-<35 30-<35 96 95 2 2 2 2 2 3 0 1 1 1 0 1 2 1 3.465

<20y <20y 107 102 1 2 1 3 2 1 1 1 1 2 1 1 1 0 3.29

30-<35 30-<35 94 104 2 1 1 2 2 2 0 1 1 2 0 2 3 1 4.51

20+yr <20y 106 101 2 2 1 2 2 2 0 1 1 1 1 3.65

25-<30 =35+ 112 1 1 1 2 3 1 0 1 2 0 0 1 2 0 3.39

20+yr =35+ 106 93 2 2 1 1 2 1 0 1 1 1 0 1 2 0 2.71

25-<30 30-<35 132 1 1 1 2 3 3 0 2 1 2 1 3.21

<20y 25-<30 105 93 2 2 2 3 1 3 1 1 1 0 0 1 2 0 3.25

20+yr <20y 114 95 2 2 2 2 3 0 1 1 1 0 1 3 0 3.8

<20y 25-<30 110 100 2 1 1 2 2 0 1 1 2 0 1 2 0 4.22

<20y <20y 105 100 1 2 2 2 2 2 2 1 1 2 1 1 3 1 3.41

25-<30 25-<30 90 89 2 1 2 2 2 3 0 1 1 2 1 1 2 0 3.57

30-<35 30-<35 96 93 2 1 1 2 2 3 2 1 1 0 0 1 2 1 3.32

20+yr <20y 109 1 2 1 1 2 1 2 1 2 2 0 1 2 0 4.14

<20y <20y 100 99 1 1 1 3 2 3 0 1 1 1 0 1 1 1 3.58

25-<30 25-<30 104 100 2 1 1 2 2 2 0 1 1 2 0 1 2 0 3.5

30-<35 30-<35 107 2 2 2 2 0 1 1 2 2 3.47

25-<30 30-<35 100 103 1 2 1 2 2 2 0 1 1 2 0 1 0 2.98

25-<30 30-<35 111 2 1 1 3 2 2 0 2 1 2 0 2 2 0 3.38

<20y <20y 100 102 2 1 1 1 2 2 1 1 1 1 0 1 2 0 2.58

<20y <20y 95 82 1 2 1 2 2 1 1 1 0 0 2 2 1 3.76

<20y 25-<30 92 97 1 2 1 2 2 2 0 1 1 1 0 3.96

<20y <20y 114 100 2 1 2 2 2 3 2 1 1 1 1 1 3 1 3.43

20+yr <20y 101 85 2 1 1 2 2 2 1 2 0 0 1 2 0 3.355

25-<30 30-<35 95 80 2 2 2 2 1 2 3.725

<20y 25-<30 109 101 2 2 1 2 2 2 1 1 1 2 1 1 3 0 3.33

=35+ =35+ 122 128 2 1 1 3 3 3 0 1 1 2 0 1 2 0 3.6

<20y <20y 91 99 1 1 2 2 1 1 2 1 1 1 0 1 2 0 2.91

25-<30 30-<35 110 112 2 1 2 3 3 3 0 1 1 2 0 1 2 1 3.22

25-<30 30-<35 104 103 1 1 1 3 2 1 1 1 1 1 0 1 2 0 3.61

<20y <20y 115 122 2 2 2 3 3 3 2 1 1 2 0 2 2 0 3.83

25-<30 30-<35 109 103 1 1 1 2 2 3 0 2 1 2 1 3.49

=35+ =35+ 118 83 2 2 2 2 2 2 1 1 1 1 1 3.27

25-<30 25-<30 114 95 2 1 2 2 2 1 1 1 1 0 1 2 2 0 1.81

20+yr <20y 102 102 2 2 1 2 2 2 0 2 1 0 0 2 3 0 3.36

30-<35 =35+ 98 128 2 1 1 2 3 3 0 1 1 1 0 1 2 1 3.78

<20y 25-<30 112 115 2 2 1 2 3 2 0 1 1 2 1 1 2 0 3.31

<20y <20y 106 2 1 2 2 2 2 0 1 1 2 0 1 2 1 3.13

25-<30 <20y 102 114 2 1 1 2 2 1 0 1 1 2 0 1 3 1 3.48

<20y <20y 110 93 1 2 1 3 2 3 2 1 1 1 0 1 2 0 3.31

30-<35 30-<35 102 93 1 1 2 2 2 2 0 1 1 0 0 2 0 3.43

30-<35 =35+ 98 104 2 1 2 2 2 2 0 1 1 2 0 1 2 0 2.225

25-<30 25-<30 99 97 2 1 2 2 2 3 1 1 1 1 2 3.63

<20y 100 119 1 2 1 2 1 0 1 1 1 0 1 2 0 4.6

20+yr 120 87 2 2 1 1 3 2 2 1 1 0 1 1 3.13

30-<35 =35+ 115 77 2 1 1 2 3 3 0 1 1 1 0 1 1 0 3.62

25-<30 =35+ 108 100 2 1 2 2 2 2 2 1 1 1 0 2 2 0 2.9

25-<30 30-<35 100 87 1 1 1 1 2 2 2 2 2 1 0 1 2 1 3.9

25-<30 25-<30 103 101 2 1 1 3 2 1 1 2 1 0 1 1 0 3.01

25-<30 30-<35 98 93 2 1 1 2 2 2 0 1 1 2 0 1 2 0 3.68

25-<30 <20y 98 87 1 2 2 2 1 3 0 1 1 0 0 1 0 2.74

<20y 25-<30 114 101 2 1 1 2 2 3 2 2 1 1 1 3.95

25-<30 30-<35 120 112 2 2 1 2 2 3 2 2 1 0 0 1 3 0 3.34

25-<30 25-<30 105 96 2 2 2 3 2 2 0 1 1 1 0 1 2 0 2.92

25-<30 25-<30 100 1 1 2 2 2 2 1 1 1 2 0 1 2 0 4.06

<20y <20y 95 1 1 2 2 2 0 2 1 1 0 1 2 1 3.32

<20y <20y 110 95 2 1 1 2 2 3 0 1 1 2 0 1 2 0 3.46

<20y 25-<30 120 100 1 1 1 2 1 1 0 1 1 2 1 2.186

25-<30 30-<35 96 87 2 1 2 2 3 3 0 1 1 1 0 1 0 3.135

30-<35 =35+ 99 77 2 2 2 2 3 2 2 1 2 0 1 2 2 1 3.13

<20y <20y 93 101 2 1 2 2 2 3 1 1 1 2 0 1 1 3.63

25-<30 25-<30 100 93 2 1 1 2 2 0 1 1 2 0 1 1 0 3.68

<20y <20y 108 1 2 2 1 2 1 2 2 1 0 2 1 3.35

<20y <20y 120 100 1 2 1 2 1 1 1 1 1 1 0 1 2 0 3.71

20+yr 96 1 2 2 2 1 1 2 1 1 0 1 1 2 0 2.95

<20y 25-<30 103 101 2 1 2 2 3 1 1 1 2 2 3.57

<20y 25-<30 106 103 2 2 1 2 2 3 0 1 1 2 0 1 2 1 4.15

25-<30 25-<30 102 85 2 1 1 1 1 2 1 1 2 0 1 3 0 3.46

20+yr <20y 91 104 2 1 2 2 2 1 0 1 1 1 0 1 2 0 3.425

<20y 25-<30 102 91 2 1 2 1 2 3 0 1 1 1 0 1 2 0 2.92

=35+ =35+ 94 94 1 1 2 1 1 3 2 1 1 1 1 1 2 0 3.13

25-<30 25-<30 111 98 1 2 1 2 2 1 0 1 1 1 1 3.78

<20y 30-<35 99 96 2 1 2 2 1 3 0 1 1 1 0 1 3 0 3.42

<20y 25-<30 93 88 2 1 2 1 2 1 0 2 1 1 0 2 1 1 3.39

30-<35 =35+ 102 91 1 2 2 3 1 2 1 2 0 1 2 1 3.16

20+yr 20+yr 100 2 1 1 2 2 1 0 1 1 2 1 3.83

<20y <20y 107 105 1 2 2 3 2 3 2 1 1 2 1 1 0 3.49

<20y 25-<30 103 106 2 1 1 2 2 3 0 1 1 2 0 2 2 1 3.125

<20y <20y 75 2 1 2 2 2 2 1 2 1 1 0 2 2 1 2.75

30-<35 30-<35 110 98 2 1 1 2 2 1 0 1 1 0 0 1 3.33

20+yr 25-<30 120 100 2 1 1 2 3 2 0 1 1 2 1 1 2 0 4.29

30-<35 30-<35 107 2 1 2 2 2 3 0 1 1 1 0 1 2 1 3.08

=35+ =35+ 109 92 1 2 1 2 2 1 1 1 1 1 1 2 1 3.42

30-<35 =35+ 83 95 2 1 1 1 1 0 1 1 2 0 1 2 1 3.5

<20y 25-<30 101 84 1 1 2 1 2 3 0 1 1 1 0 1 2 1 2.9

25-<30 30-<35 110 106 2 2 2 3 3 3 0 1 1 2 0 1 2 1 3.7

<20y <20y 97 89 2 2 2 2 2 3 0 1 1 0 2 1 2.09

<20y <20y 114 106 1 1 2 3 3 2 0 1 1 2 0 1 2 0 3.38

20+yr <20y 110 90 1 2 2 2 2 3 0 1 1 1 1 3.14

25-<30 30-<35 107 114 2 1 2 3 2 3 2 1 1 2 0 1 3 1 2.3

25-<30 30-<35 110 110 2 2 1 2 2 3 0 1 1 2 0 1 2 1 3.99

20+yr <20y 106 104 2 1 1 2 2 2 0 2 1 2 0 1 3.96

<20y 25-<30 104 96 2 1 1 1 2 2 0 1 1 2 0 2 2 0 3.495

30-<35 =35+ 92 85 1 1 1 1 2 1 1 2 0 1 2 1 3.77

20+yr <20y 106 97 2 2 1 2 2 2 2 1 1 1 0 1 2 0 2.1

25-<30 30-<35 97 90 2 2 1 2 2 1 1 1 1 1.014

25-<30 30-<35 110 2 1 1 2 3 1 0 1 1 2 0 1 1 0 3.79

25-<30 25-<30 94 114 2 1 2 2 2 1 0 1 1 1 0 1 2 1 2.86

<20y 25-<30 118 95 2 1 1 1 3 2 2 1 1 2 0 1 2 0 3.275

25-<30 25-<30 104 92 2 1 1 3 3 3 0 1 1 2 0 1 2 1 3.795

25-<30 =35+ 129 95 2 1 1 2 2 2 0 1 1 2 0 1 1 3.42

30-<35 30-<35 109 102 2 2 1 2 1 3 0 1 1 2 0 1 3 0 3.25

<20y 25-<30 90 92 1 1 2 3 2 1 0 1 1 1 1 2 1 3.03

25-<30 25-<30 92 2 1 2 1 1 2 2 1 1 2 1 1 2 1 4.08

25-<30 <20y 93 93 2 1 1 2 2 2 0 1 1 2 0 2 2 0 3.57

=35+ =35+ 114 87 2 1 1 2 3 2 1 1 2 0 0 1 3 0 4.03

20+yr 108 1 2 1 1 1 1 1 1 0 2 2 1 3.61

20+yr <20y 115 96 2 2 1 2 2 0 1 1 0 0 1 2 0 2.81

25-<30 25-<30 99 80 2 2 1 2 2 3 1 2 1 1 1 1 2 1 3.18

25-<30 =35+ 123 96 2 1 1 2 2 2 0 2 1 1 0 1 2 1 3.57

=35+ =35+ 100 113 2 2 2 1 1 2 0 1 1 1 1 1 3 0 3.04

20+yr <20y 118 102 1 2 1 2 1 0 1 1 1 2 4.28

25-<30 30-<35 95 108 1 2 1 2 2 2 2 1 1 2 0 1 3 0 3.025

<20y 25-<30 109 86 2 2 1 2 2 2 0 1 1 2 0 2 1 4.26

25-<30 25-<30 106 90 2 1 2 2 2 2 0 1 1 2 0 1 2 1 3.08

20+yr 106 102 1 2 1 1 1 2 2 1 1 0 2 2 3.07

<20y 25-<30 102 114 2 2 1 2 2 3 0 1 1 2 0 1 2 1 4.23

<20y =35+ 99 97 2 2 2 2 2 3 0 2 1 2 1 3.59

=35+ 92 79 1 2 2 1 2 3 2 2 1 0 0 1 2 1 2.78

=35+ =35+ 122 88 1 1 1 3 1 1 0 1 1 2 1 1 3.43

25-<30 <20y 115 93 2 1 1 1 2 2 0 1 1 2 0 1 0 3.4

25-<30 30-<35 107 117 1 1 2 3 3 3 0 1 1 2 0 1 3 0 2.77

<20y 25-<30 101 91 2 1 2 2 2 1 0 1 1 1 0 1 2 1 4.005

20+yr <20y 112 97 2 2 2 2 2 1 2 1 1 1 0 1 1 2.84

<20y <20y 109 101 1 1 2 2 2 3 0 1 1 1 0 1 1 0 4.55

30-<35 30-<35 92 2 1 1 1 2 0 1 1 0 1 1 3.03

<20y <20y 110 81 2 2 2 2 2 2 2 1 1 0 1 2.77

25-<30 =35+ 84 96 1 1 1 2 1 2 0 1 1 2 0 1 2 0 4.01

25-<30 30-<35 97 103 1 2 1 2 2 2 0 1 1 1 0 1 2 0 3.925

20+yr <20y 104 1 2 2 2 2 2 1 1 1 1 0 1 2 1 3.73

30-<35 =35+ 125 128 2 1 1 2 3 3 0 1 1 2 0 1 2 0 3.72

20+yr <20y 95 88 2 2 1 3 2 3 0 1 1 2 0 1 2 0 3.28

<20y 25-<30 109 108 2 1 2 3 2 3 0 1 1 1 0 1 2 0 3.07

=35+ =35+ 99 103 2 2 2 2 1 2 0 1 1 2 0 1 2 0 3.92

20+yr 86 90 1 2 2 2 3 0 1 1 0 0 1 2 1 3.74

<20y 25-<30 91 98 2 2 2 2 2 2 0 1 1 1 0 1 2 0 3.5

25-<30 30-<35 109 112 2 2 1 3 2 2 0 1 1 1 0 1 2 1 3.31

25-<30 =35+ 100 2 2 1 2 2 2 0 1 1 2 0 2 2 1 3.41

30-<35 30-<35 118 103 2 1 2 3 2 2 0 1 1 2 0 2 3 0 3.17

25-<30 25-<30 99 2 1 2 2 3 3 2 2 1 0 1 1 2 0 3.19

<20y 25-<30 112 101 2 1 1 3 3 3 0 1 1 2 1 1 2 0 3.16

<20y <20y 98 93 2 1 1 3 0 1 1 0 1 1 1 0 3.83

25-<30 30-<35 104 94 2 1 2 3 3 2 0 1 1 2 0 1 3 0 3.36

<20y 99 1 2 2 2 3 2 1 1 1 0 1 2 0 3.55

=35+ 25-<30 95 90 1 2 2 2 2 1 1 2 1 0 0 1 1 1 3.76

30-<35 30-<35 100 100 1 2 2 2 2 2 2 1 0 0 1 2 0 3.91

25-<30 25-<30 110 2 1 2 2 2 3 0 2 1 2 0 1 3 0 3.83

<20y 25-<30 104 1 1 2 2 3 2 1 1 1 2 1 1 1 1 3.7

30-<35 115 97 2 2 1 2 3 1 2 2 1 2 0 1 3 0 3.555

<20y 25-<30 96 92 2 2 2 2 3 2 1 2 1 2 0 1 2 0 3.05

20+yr 107 1 1 1 3 2 0 1 1 1 1 1 2 1 2.94

<20y 25-<30 111 104 1 2 2 2 1 1 0 1 1 2 1 2.66

20+yr 20+yr 114 93 2 2 2 2 2 1 1 1 1 1 1 3.47

<20y 25-<30 104 106 2 2 2 2 2 2 0 1 1 2 0 1 2 0 3.415

25-<30 25-<30 111 108 2 1 1 1 3 3 2 1 1 1 1 3.25

<20y <20y 94 85 2 2 1 2 1 3 1 2 1 2 1 2 1 3.08

25-<30 30-<35 101 112 2 1 2 2 2 2 1 1 0 0 1 2 0 3.67

<20y 25-<30 100 110 2 2 1 2 2 3 0 1 1 1 1 1 2 1 3.5

<20y <20y 96 2 2 2 3 3 2 0 1 1 1 1 1 2 1 3.29

<20y 25-<30 111 98 2 2 2 2 2 3 0 1 1 2 0 1 2 0 3.8

20+yr 30-<35 109 1 2 2 1 3 2 1 2 1 2 1 2.89

25-<30 30-<35 110 89 2 1 2 3 3 2 1 2 1 2 0 2 2 0 3.7

=35+ =35+ 94 98 1 2 2 2 2 2 0 1 1 2 0 2 2 1 3.84

<20y <20y 99 97 2 2 2 1 2 1 2 1 1 1 0 1 2 3.74

<20y 25-<30 95 2 1 2 2 2 3 0 1 1 2 0 1 2 0 3.45

<20y <20y 108 103 1 2 2 2 2 2 0 1 1 2 0 1 3 0 3.24

25-<30 30-<35 111 2 2 1 2 1 3 2 1 1 1 0 1 2 0 2.99

<20y <20y 118 2 1 2 2 2 2 1 1 1 1 0 1 2 0 2.29

<20y 91 79 2 2 2 1 1 1 1 1 0 1 1 1 1 2.64

<20y <20y 92 110 1 2 1 2 2 1 0 1 1 2 0 1 2 0 3.29

=35+ =35+ 96 81 1 2 2 2 2 2 0 1 1 0 2 1 1 3.23

=35+ 30-<35 122 100 1 2 1 2 3 3 0 1 1 2 0 1 2 1 4.125

25-<30 30-<35 100 1 2 2 3 3 1 0 1 1 2 1 3.475

25-<30 25-<30 101 106 1 2 2 3 2 3 1 1 1 2 0 1 2 1 3.01

=35+ 30-<35 111 84 2 2 1 1 2 1 2 1 1 2 0 1 2 1 3.7

<20y 30-<35 97 2 2 2 3 2 1 1 1 2 0 1 2 0 2.87

20+yr <20y 115 92 1 2 2 2 2 1 1 1 1 1 2 0 2.795

20+yr 25-<30 90 1 1 1 2 2 2 2 2 1 0 0 1 2 0 3.42

25-<30 30-<35 107 101 2 1 2 2 2 3 0 1 1 1 0 1 3 0 3.36

25-<30 <20y 85 88 1 1 1 1 2 2 1 1 1 2 1 2 2 1 2.355

25-<30 25-<30 111 101 2 2 2 2 3 3 0 1 1 2 0 1 1 0 3.67

25-<30 25-<30 103 98 2 1 1 2 2 1 1 1 1 1 2 0 3.48

25-<30 =35+ 122 81 1 2 2 2 2 1 0 1 1 2 1 1 0 3.67

20+yr <20y 99 93 2 2 2 3 2 1 0 2 1 2 0 1 2 0 3.87

25-<30 25-<30 103 90 2 1 1 1 2 3 0 1 1 2 0 1 2 1 3.42

25-<30 30-<35 115 104 2 1 2 2 3 2 2 1 1 1 0 1 2 0 3.085

<20y 25-<30 111 97 2 2 2 2 2 1 2 1 2 0 1 2 0 3.71

30-<35 =35+ 99 103 2 2 2 2 2 3 0 1 1 2 0 1 2 0 2.78

30-<35 30-<35 118 2 1 2 3 2 3 0 1 1 2 0 1 3 0 2.55

<20y 25-<30 97 2 1 1 1 1 1 0 1 1 1 0 1 1 0 3.1

=35+ =35+ 97 97 1 2 1 3 2 1 2 1 1 1 0 1 2 0 2.63

<20y 25-<30 84 96 1 1 2 2 2 0 1 1 2 0 1 2 0 3.75

<20y 25-<30 110 93 1 2 1 2 3 1 0 2 1 2 0 2 2 0 3.98

25-<30 25-<30 112 86 1 1 1 2 2 0 2 1 1 0 1 2 0 3.885

25-<30 25-<30 100 97 2 1 2 2 3 0 1 1 0 1 2 2 0 3.13

25-<30 25-<30 122 104 2 1 2 2 3 3 2 1 1 1 0 1 2 0 3.53

20+yr <20y 97 90 1 2 2 2 2 1 2 1 1 1 1 2.93

25-<30 25-<30 90 95 2 2 1 1 2 2 2 1 1 1 1 2.552

30-<35 25-<30 104 103 2 1 2 3 3 2 0 1 1 2 1 1 1 3.64

<20y 25-<30 111 104 2 2 1 2 1 3 0 2 2 1 0 3.98

<20y <20y 111 88 1 1 2 2 2 0 1 1 2 1 3.84

<20y =35+ 100 92 2 2 2 1 2 2 2 2 1 2 0 1 1 1 2.87

<20y 30-<35 104 2 1 2 3 1 1 1 2 1 0 1 1 2 0 4.03

<20y <20y 99 88 1 1 1 2 2 2 0 1 1 2 0 1 2 0 4.23

25-<30 30-<35 109 101 1 1 2 2 2 2 0 1 1 1 0 1 0 3.76

=35+ =35+ 115 81 1 2 2 1 2 2 0 1 1 2 1 1 2 1 2.965

25-<30 25-<30 97 101 1 2 1 2 2 2 0 1 1 2 0 1 2 0 3.51

20+yr <20y 99 86 1 2 2 1 2 2 1 1 1 2 1 1 3 1 3.79

=35+ =35+ 109 105 2 2 1 2 3 2 0 1 1 2 1 1 3 0 3.6

25-<30 30-<35 112 122 1 2 1 3 2 1 0 1 1 2 0 1 2 0 3.56

25-<30 25-<30 107 100 1 2 3 3 2 0 1 1 2 1 1 2 1 3.91

25-<30 25-<30 116 98 2 2 1 2 3 3 0 1 1 1 0 1 2 1 3.665

25-<30 25-<30 106 85 2 1 2 2 2 2 1 1 1 1 0 1 3 0 3.52

<20y 25-<30 108 1 1 2 2 2 2 2 2 1 0 2 1 3.53

<20y 25-<30 83 86 2 1 2 2 2 3 0 1 1 2 0 1 2 1 3.6

25-<30 110 125 2 1 1 2 3 2 0 1 1 2 1 1 2 0 3.89

20+yr <20y 93 102 1 2 2 2 1 1 0 2 1 0 0 1 2 3.15

25-<30 <20y 95 97 2 1 1 2 2 1 1 1 1 1 0 1 2 1 3

25-<30 25-<30 88 82 2 1 1 1 3 2 0 1 1 0 1 2 1 3.26

30-<35 30-<35 104 114 2 2 1 3 2 3 2 2 2 2 1 3.26

30-<35 30-<35 95 101 2 1 2 1 2 3 2 2 1 0 1 1 3 0 4.34

<20y <20y 90 87 2 2 1 1 2 0 2 1 1 0 1 2 1 3.59

30-<35 30-<35 103 93 2 1 2 2 1 1 0 2 1 2 0 1 2

20+yr <20y 86 91 2 1 2 2 2 2 0 1 1 0 1 3.57

<20y <20y 102 91 2 1 1 2 2 3 0 1 1 0 1 1 1 0 3.54

<20y 30-<35 110 83 2 1 1 2 1 3 0 1 1 0 2 4.35

25-<30 30-<35 97 98 2 1 2 2 2 2 2 2 1 2 0 1 2 0 3.41

<20y 25-<30 112 112 2 1 2 2 2 3 2 1 1 2 0 1 2 1 3.18

20+yr 112 98 1 2 1 2 1 2 1 1 2 1 2.54

20+yr <20y 83 89 2 2 1 1 2 1 2 1 1 0 0 1 2 1 4.02

25-<30 =35+ 97 1 1 1 3 2 0 1 1 2 1 1 2 1 3.73

<20y <20y 114 100 2 2 2 2 2 2 0 1 1 2 0 1 2 0 3.31

<20y 30-<35 108 101 1 2 1 2 3 1 0 1 1 2 1 1 3 1 2.91

25-<30 25-<30 107 95 2 1 2 2 2 3 0 1 1 2 1 1 3 0 3.565

30-<35 =35+ 111 104 2 1 2 3 2 3 0 1 1 2 0 1 3 1 2.64

30-<35 30-<35 115 99 1 2 2 2 2 3 0 1 1 2 0 1 3 0 3.49

<20y <20y 107 110 2 2 2 2 3 2 0 2 1 2 0 1 2 0 3.68

<20y =35+ 110 1 1 1 3 1 2 0 1 1 2 0 1 2 1 3.56

<20y 25-<30 92 80 2 1 1 2 2 2 0 1 1 2 0 1 2 1 3.43

=35+ =35+ 104 1 1 2 1 1 2 2 2 1 1 2 2.96

25-<30 =35+ 99 2 2 1 1 3 0 1 1 1 0 1 3 1 3.69

25-<30 25-<30 94 100 2 2 2 2 2 2 0 1 1 2 0 1 2 0 3.67

25-<30 30-<35 114 96 1 1 2 3 3 1 0 1 1 2 0 1 2 0 3.65

25-<30 30-<35 98 94 2 2 2 2 2 3 0 1 1 2 0 1 2 0 3.45

25-<30 25-<30 115 95 1 1 2 1 2 0 1 1 2 0 1 2 1 3.64

25-<30 30-<35 99 2 1 2 2 1 2 1 1 1 0 1 1 3 0 3.95

20+yr 20+yr 86 92 1 2 1 3 1 1 0 1 1 1 1 1 2 1 4.31

<20y <20y 113 1 2 2 2 3 2 0 1 1 2 0 1 2 1 3.54

25-<30 30-<35 94 103 2 2 2 3 3 0 2 1 2 0 1 1 3.3

20+yr 99 95 1 2 1 2 2 2 0 1 2 0 0 1 2 0 3.28

<20y 25-<30 101 108 1 1 2 2 2 2 0 1 1 1 1 2.96

<20y <20y 117 111 2 1 1 3 3 1 0 1 1 2 1 1 2 0 3.135

20+yr <20y 106 105 1 1 2 1 1 1 0 1 1 0 1 1 2 1 3.85

<20y 25-<30 87 80 1 1 2 2 1 2 2 1 0 2 2.52

<20y 25-<30 93 103 2 2 2 2 2 2 1 2 1 2 1 1 2 1 2.82

25-<30 30-<35 111 81 2 2 2 3 3 3 0 1 1 1 0 2 2 1 3.65

<20y 25-<30 88 90 2 1 1 2 2 2 1 2 1 1 0 1 2 0 2.216

<20y 25-<30 111 112 2 1 1 2 2 2 0 1 1 2 0 1 2 0 3.84

20+yr <20y 88 86 2 1 2 2 2 1 0 1 1 0 0 2 2 1 3.02

<20y 30-<35 95 103 2 2 2 3 3 2 0 1 1 1 0 1 2 0 3.145

<20y 25-<30 106 85 2 2 2 2 3 2 0 1 1 0 1 3 1 1.695

<20y 25-<30 107 1 2 2 2 2 2 0 1 1 0 1 1 1 1 2.62

<20y <20y 108 1 2 1 2 2 0 1 1 1 1 1 2 0 3.54

<20y 25-<30 97 103 1 1 1 3 2 2 0 1 1 1 1 1 2 1 4.27

<20y 25-<30 97 89 1 1 2 2 2 2 0 1 1 2 0 1 2 0 3.08

<20y 25-<30 88 86 1 2 1 2 2 0 1 1 1 0 1 2 1 4.09

<20y 25-<30 101 94 2 2 1 2 2 1 2 1 1 1 0 1 2 0 3.66

<20y 30-<35 104 92 1 1 2 2 2 2 0 1 1 1 1 2 3 0 2.61

<20y 25-<30 105 99 1 2 1 2 2 2 2 1 1 1 1 1 3 1 3.73

<20y <20y 99 88 2 2 1 2 2 2 2 1 2 2 1 1 2 0 3.65

25-<30 25-<30 98 104 2 1 1 2 1 3 0 1 1 0 0 1 2 1 3.17

25-<30 25-<30 110 88 2 1 2 2 2 3 0 2 1 0 0 2 2 1 2.94

<20y 25-<30 127 108 1 1 1 2 2 2 1 1 2 1 3.35

30-<35 =35+ 112 112 2 2 1 3 3 3 0 1 1 2 0 2 2 0 2.58

30-<35 <20y 105 2 2 1 3 2 3 0 1 1 2 0 1 3 1 4.32

<20y 25-<30 104 98 2 1 2 2 3 0 1 1 2 0 1 2 1 3.23

<20y <20y 92 88 2 2 1 2 2 1 0 1 1 1 1 1 2 0 4.08

<20y 25-<30 108 108 1 2 1 2 2 1 2 1 1 1 0 1 2 1 3.08

<20y <20y 107 2 1 1 3 2 2 0 1 1 1 0 1 2 0 3.49

25-<30 30-<35 107 107 2 2 1 2 2 2 0 1 1 1 0 1 2 1 3.69

=35+ =35+ 88 95 2 1 1 2 2 2 0 1 1 2 1 1 1 3.7

25-<30 30-<35 98 112 2 1 1 2 2 3 0 1 1 1 0 2 1 3.8

25-<30 30-<35 108 87 2 1 2 2 2 2 0 1 1 2 1 2 2 1 3.27

<20y 102 87 2 2 2 2 2 2 2 1 1 0 1 3.6

<20y <20y 92 1 2 3 1 2 1 1 1 0 0 1 2 0 2.68

<20y <20y 106 88 2 1 2 2 3 1 1 1 1 1 0 1 1 0 4.08

25-<30 30-<35 120 133 2 2 1 2 3 2 0 1 1 1 1 2 2 1 3.45

30-<35 =35+ 104 107 2 1 1 2 2 0 1 1 2 1 2 3 1 4.03

25-<30 =35+ 100 117 1 2 1 3 3 1 0 1 1 2 0 2 3 0 3.41

25-<30 25-<30 107 1 2 2 2 2 2 0 1 1 1 0 1 2 0 3.53

25-<30 25-<30 107 1 2 1 3 3 2 0 1 1 2 0 1 3 1 4

30-<35 25-<30 100 91 2 2 2 2 2 1 2 2 2 1 1 1 3 1 3.25

=35+ =35+ 82 89 1 1 1 1 0 1 1 1 1 1 2 1 3.97

<20y <20y 122 111 2 1 1 2 1 3 1 1 1 1 1 1 2 0 3.62

<20y <20y 94 2 1 2 1 2 2 1 1 2 1 1 2 1 3.46

25-<30 25-<30 92 85 1 2 2 2 3 2 1 1 0 0 2 2 1 3.3

30-<35 30-<35 118 108 2 2 1 3 3 3 0 1 1 2 1 3.215

25-<30 30-<35 99 92 2 1 1 2 3 3 0 1 1 2 0 1 2 0 4

=35+ =35+ 115 128 2 2 2 3 2 0 1 1 2 0 1 3 1 2.85

<20y <20y 96 88 2 2 1 2 2 1 2 1 1 1 0 1 2 0 3.07

<20y 101 88 1 1 2 3 2 1 2 1 1 2 1 3.78

<20y 25-<30 103 110 1 1 1 2 3 2 0 1 1 2 0 1 2 0 3.255

<20y <20y 85 76 2 2 2 2 2 1 2 1 1 1 1 1 2 1 2.35

<20y <20y 90 2 2 2 2 2 0 1 1 1 0 1 3 1 2.42

<20y <20y 110 101 1 2 2 2 2 3 0 1 1 2 1 1 2 0 3.46

<20y 25-<30 102 102 2 1 1 2 2 1 2 1 1 2 0 1 2 0 3.76

25-<30 30-<35 88 80 1 1 1 1 2 2 0 1 1 1 0 1 3.9

25-<30 =35+ 114 106 2 1 2 1 1 2 2 1 1 1 1 2 0 4.3

30-<35 =35+ 115 110 2 1 2 1 3 2 0 1 1 2 1 1 2 0 4.1

<20y 30-<35 102 88 2 2 2 2 3 3 0 1 1 1 0 1 2 0 2.995

25-<30 <20y 99 95 2 1 1 2 2 3 1 2 1 2 1 1 3 0 3.57

20+yr <20y 83 98 2 1 2 2 3 1 1 1 1 0 1 2 1 3.51

<20y <20y 78 79 2 1 1 2 2 2 1 1 2 0 1 2 1 3.4

<20y <20y 102 104 2 1 1 2 2 3 0 1 1 2 1 1 2 1 4.36

25-<30 30-<35 103 96 2 2 2 2 3 1 0 1 1 2 1 1 2 0 3.01

<20y <20y 101 111 2 2 2 3 3 3 0 1 1 1 0 2 2 0 3.11

25-<30 30-<35 111 97 2 1 2 2 2 3 0 1 1 1 0 1 2 0 3

<20y <20y 102 96 2 2 1 2 2 1 0 1 1 1 0 2 2 1 3.71

<20y 20+yr 89 1 2 1 2 1 3 0 1 1 0 1 1 2 1 3.004

25-<30 25-<30 86 88 2 2 1 2 2 2 0 1 1 2 0 2 1 0 4.41

25-<30 25-<30 99 89 2 1 2 2 2 2 0 1 1 0 1 3.51

20+yr <20y 101 86 2 2 2 1 2 0 1 1 1 2 3.37

25-<30 =35+ 103 98 2 2 1 2 2 3 0 1 1 2 0 1 2 0 2.835

25-<30 30-<35 105 1 1 1 2 3 3 0 1 1 1 1 1 0 4.47

25-<30 96 86 2 1 2 2 2 2 1 2 1 2 1 2 1 3.59

<20y <20y 110 87 2 2 1 1 1 2 1 1 0 1 3.49

<20y <20y 85 1 1 1 1 2 2 1 1 1 1 0 1 2 0 3.4

25-<30 25-<30 115 110 2 2 2 2 3 1 0 1 1 1 0 1 2 1 4.27

<20y 25-<30 107 90 1 1 1 2 2 3 1 1 1 1 0 1 2 0 2.98

30-<35 =35+ 109 97 2 1 1 2 1 1 0 1 1 2 0 1 2 1 3.195

25-<30 30-<35 110 95 2 2 1 2 2 2 0 1 1 2 1 1 1 0 3.12

<20y 25-<30 88 80 2 1 2 1 2 3 1 2 1 2 1 1 4.415

25-<30 =35+ 96 90 1 2 2 3 2 2 2 1 1 2 1 2 2 0 2.68

<20y <20y 96 85 1 2 2 2 2 1 1 1 2 1 2.66

25-<30 25-<30 105 96 2 1 1 2 2 3 0 1 1 2 1 1 2 0 3.48

<20y <20y 98 93 2 2 2 2 1 2 0 1 1 0 0 1 2 1 3.79

25-<30 30-<35 106 92 2 2 2 2 3 3 0 1 1 2 1 1 3 0 4.09

20+yr =35+ 101 96 1 2 2 2 3 3 0 1 1 0 0 1 2 1 2.95

<20y <20y 96 91 2 1 2 1 1 1 2 1 1 2 0 1 2 1 3.21

30-<35 =35+ 125 114 2 1 2 3 3 2 0 2 1 2 1 2 2 0 4.02

<20y 25-<30 113 109 2 1 2 3 1 1 0 1 1 1 0 1 0 3.57

30-<35 =35+ 102 98 2 1 1 2 2 3 0 1 1 0 0 1 1 0 3.68

20+yr 90 88 1 2 2 1 1 2 2 1 1 1 2 2 1 3.54

25-<30 30-<35 96 92 2 1 1 2 2 2 0 1 1 2 1 1 2 1 3.77

30-<35 30-<35 117 128 2 2 1 2 2 3 0 2 1 2 0 1 2 1 3.51

20+yr <20y 103 85 2 2 2 1 2 1 0 1 1 0 1 1 2 0 4.03

30-<35 =35+ 104 105 2 1 1 2 2 3 0 1 1 2 0 2 2 1 4.1

<20y <20y 90 100 1 1 2 2 1 1 0 2 1 1 1 2 3 0 3.15

20+yr <20y 94 84 1 2 2 2 1 1 2 2 2 2 3.465

=35+ =35+ 79 1 2 1 1 2 2 2 1 0 0 1 2 0 2.76

<20y <20y 111 110 2 1 2 1 3 0 1 1 1 0 1 2 0 4.11

25-<30 30-<35 103 98 2 1 2 1 2 2 0 1 1 2 0 2 2 0 3.29

<20y 109 88 2 1 2 3 1 2 2 1 1 1 0 1 2 0 2.73

25-<30 25-<30 92 86 1 1 2 3 3 0 1 1 0 0 1 1 3.35

<20y <20y 110 122 2 2 2 3 2 3 0 1 1 2 1 2 1 2.64

<20y 30-<35 107 103 2 2 1 2 2 3 0 1 1 2 0 1 1 1 3.3

<20y 25-<30 94 83 2 1 2 2 2 2 0 1 1 2 0 1 2 0 3.36

25-<30 =35+ 97 101 2 2 2 1 1 3 2 1 1 0 1 3.18

<20y 25-<30 122 91 2 2 1 3 3 3 0 1 1 2 0 1 2 1 3.06

30-<35 30-<35 107 93 2 2 2 2 2 3 0 1 1 2 0 1 3 0 4.11

<20y 30-<35 94 1 2 1 2 2 0 2 2 1 0 1 2 1 3.63

25-<30 25-<30 78 85 2 1 2 1 2 3 2 1 1 0 0 1 2 1 3.39

20+yr 25-<30 104 104 1 2 1 1 2 3 2 2 2 0 0 1 3 0 2.93

<20y =35+ 92 97 2 2 2 3 2 3 0 1 1 1 1 3.21

<20y 30-<35 85 2 2 2 2 2 3 2 1 1 1 1 1 2 1 2.91

25-<30 =35+ 120 92 2 2 1 3 2 3 0 1 1 2 0 1 2 0 3.275

<20y 25-<30 92 94 2 2 2 2 3 0 1 1 0 0 1 2 1 4.14

<20y 25-<30 88 89 2 1 1 2 2 3 0 2 1 2 0 1 2 0 3.83

25-<30 30-<35 104 101 2 1 1 2 2 2 2 1 1 0 0 1 2 1 3.47

30-<35 =35+ 106 90 2 2 2 3 0 2 2 0 1 1 0 2.67

<20y <20y 91 92 2 2 1 3 3 3 0 2 1 1 1 1 2 1 3.4

25-<30 =35+ 114 91 1 2 1 1 3 1 0 1 1 2 0 1 2 0 2.74

25-<30 25-<30 84 98 2 1 1 3 3 3 0 1 1 2 0 1 2 1 3.02

30-<35 30-<35 117 99 1 2 1 3 3 3 0 1 1 1 1 4.075

<20y 25-<30 114 104 2 2 2 2 3 1 2 1 1 1 0 1 2 1 4.03

25-<30 25-<30 129 101 2 1 1 2 2 3 0 1 1 1 0 1 2 0 3.28

<20y 20+yr 102 91 1 2 1 2 2 1 2 2 1 1 0 1 2 1 3.4

<20y <20y 114 109 2 1 1 2 3 3 0 1 1 1 0 1 2 0 2.95

<20y =35+ 127 2 2 2 1 3 0 1 1 2 1 1 2 1 3.01

<20y 30-<35 97 89 2 1 2 2 2 2 0 1 1 1 1 1 2 0 2.84

25-<30 30-<35 102 80 2 1 2 3 1 1 1 0 0 1 1 3.62

25-<30 30-<35 92 110 1 1 1 2 2 2 2 2 1 1 0 1 2 1 2.64

<20y 25-<30 124 101 2 2 2 3 2 3 0 2 1 2 0 1 2 1 4.15

25-<30 25-<30 91 119 2 2 2 2 3 3 0 1 1 2 0 1 3 0 3.38

20+yr 25-<30 103 101 2 2 2 2 2 2 0 1 1 1 0 1 2 0 3.1

25-<30 95 95 2 1 2 2 2 2 2 1 1 1 1 2 0 3.54

=35+ =35+ 97 86 2 2 1 1 1 3 2 2 1 0 0 1 2.3

<20y 96 100 1 2 2 2 1 1 2 1 2 1 2 1 3.99

<20y 25-<30 81 103 2 1 1 2 2 3 1 2 1 0 1 1 2 1 3.8

<20y 25-<30 88 2 1 2 2 3 1 0 1 1 1 0 1 2 1 3.16

20+yr <20y 89 99 2 1 2 1 1 1 1 1 1 0 1 2 0 3.725

<20y 25-<30 104 104 2 1 2 2 2 3 0 1 1 2 0 2 2 0 3.71

<20y <20y 118 95 1 2 2 2 3 3 0 1 1 2 0 1 2 0 3.26

20+yr <20y 103 91 2 2 1 2 2 1 1 1 1 2 0 1 1 1 2.95

30-<35 25-<30 98 102 2 1 1 2 1 0 1 1 2 0 2 2 1 3.18

25-<30 <20y 109 104 2 1 1 2 2 3 0 1 1 1 1 1 2 1 3.22

<20y 25-<30 117 101 2 1 1 2 2 3 0 1 1 2 0 1 2 0 3.965

25-<30 25-<30 111 108 2 1 1 2 2 2 0 1 1 2 1 1 2 1 2.685

25-<30 25-<30 97 80 2 1 1 2 2 2 0 2 1 2 3.05

=35+ =35+ 111 89 2 2 1 2 1 2 0 1 1 1 1 1 2 0 2.32

25-<30 30-<35 112 117 1 2 1 3 3 3 2 1 2 1 0 1 2 1 3.61

<20y <20y 100 115 2 2 1 2 2 3 0 1 1 1 1 2 2 0 2.747

30-<35 30-<35 98 90 2 2 2 2 3 3 0 1 1 2 0 1 0 3.11

<20y <20y 117 89 1 2 1 3 2 1 0 2 2 2 0 1 2 1 3.2

25-<30 30-<35 108 97 2 1 1 3 2 1 1 1 2 0 1 0 3.39

25-<30 =35+ 103 87 2 2 2 1 2 3 0 1 1 2 0 1 2 0 3.23

=35+ =35+ 94 90 2 2 1 2 2 2 0 1 1 2 1 3.955

<20y 110 94 2 2 2 1 2 2 2 2 1 1 0 1 1 0 3.575

25-<30 30-<35 102 2 2 2 3 3 3 1 1 1 2 0 1 3 0 3.36

=35+ 25-<30 112 89 2 2 1 3 3 1 0 1 1 0 2.86

25-<30 25-<30 103 106 1 1 2 2 2 2 0 2 1 1 1 2 2 0 2.81

<20y <20y 106 92 1 1 2 2 2 2 0 2 1 0 0 2 1 1 3.64

<20y 30-<35 84 1 2 1 2 2 3 0 1 1 0 0 2 2 1 2.94

25-<30 30-<35 91 2 2 1 2 2 0 2 1 2 0 2 2 0 3.95

<20y 30-<35 108 98 2 2 2 2 3 2 0 1 1 2 0 2 2 1 3.66

30-<35 30-<35 90 104 2 1 2 3 3 2 0 1 1 2 1 1 1 0 2.585

<20y 25-<30 125 125 2 2 1 2 2 3 1 1 1 2 0 1 2 1 4.015

<20y <20y 108 83 1 2 2 2 2 2 0 1 1 2 0 1 3 0 3.48

<20y 25-<30 115 104 2 2 2 2 2 3 1 1 1 2 0 2 2 0 2.96

30-<35 30-<35 100 1 1 1 2 1 1 1 1 2 1 1 1 2 0 3.54

25-<30 30-<35 79 97 1 1 3 2 1 0 1 1 2 0 1 3 0 3.47

<20y 25-<30 107 2 2 1 2 2 2 2 2 1 2 2 4.04

25-<30 25-<30 120 139 2 1 1 3 3 2 0 1 1 2 0 2 2 1 3.75

25-<30 30-<35 107 98 2 2 1 3 1 2 0 1 1 2 0 1 0 4.035

<20y 25-<30 106 90 1 1 2 3 2 1 0 2 1 1 0 1 2 0 3.51

<20y 30-<35 94 91 1 2 2 2 2 3 0 1 1 1 0 1 2 1 3.72

30-<35 96 85 2 2 1 0 1 1 2 0 1 3 1 4.05

25-<30 25-<30 104 100 1 1 2 2 1 3 1 1 1 0 0 1 2 0 3.35

25-<30 25-<30 134 103 2 2 1 2 2 1 0 1 1 2 0 2 2 0 2.795

30-<35 =35+ 111 96 1 2 1 3 3 3 0 1 1 2 0 1 2 0 4.12

25-<30 25-<30 99 100 2 1 1 2 2 3 0 1 1 2 0 2 2 0 3.18

20+yr <20y 101 81 2 1 2 2 2 0 1 1 1 0 1 2 2.89

<20y 25-<30 114 96 2 1 1 2 2 3 0 1 1 1 0 1 3 0 3.76

<20y 25-<30 105 89 2 1 1 3 3 2 0 1 1 1 0 2 2 1 4.2

30-<35 =35+ 108 105 1 1 2 3 3 3 1 1 1 2 0 1 0 4

=35+ =35+ 88 2 2 2 2 2 1 0 1 1 1 1 3.46

25-<30 30-<35 117 94 2 1 2 3 2 2 1 1 1 0 1 1 1 0 2.77

25-<30 30-<35 114 108 1 2 2 3 3 3 0 1 1 2 1 1 3 0 3.18

20+yr <20y 107 102 1 1 2 2 2 2 1 1 1 0 2 2.63

<20y <20y 97 86 2 1 1 2 2 3 2 1 1 0 0 1 1 0 4.595

<20y 25-<30 93 76 2 1 1 1 1 3 0 1 1 1 1 1 2 1 2.495

25-<30 30-<35 106 97 2 1 2 3 3 3 0 1 1 2 0 1 2 0 3.8

<20y 25-<30 99 1 1 2 2 2 1 1 1 2 0 1 2 0 3.26

25-<30 30-<35 110 117 1 1 2 2 2 3 1 1 1 1 1 1 2 0 3.96

30-<35 30-<35 99 84 2 2 2 1 1 2 0 1 1 1 0 1 2 0 3.35

30-<35 25-<30 118 117 2 2 1 3 3 1 0 1 1 2 0 1 3 1 3.99

<20y 25-<30 106 109 1 2 1 2 2 1 1 2 2 2 0 1 2 0 3.7

20+yr <20y 107 87 1 2 2 2 1 1 2 1 1 0 0 1 2 1 3.14

20+yr <20y 90 1 1 2 2 2 1 0 1 1 0 2 0 2.9

<20y 25-<30 119 129 2 1 1 3 1 3 0 1 1 2 0 1 2 0 3.35

20+yr <20y 82 1 2 1 2 2 0 2 1 1 0 1 2 1 3.3

25-<30 25-<30 106 112 2 2 1 2 3 3 0 1 1 2 0 1 3 0 3.375

20+yr <20y 86 1 2 2 2 2 1 2 1 1 1 0 1 2 1 3.08

25-<30 25-<30 109 1 2 1 2 3 1 0 1 1 1 0 1 3 0 3.715

<20y <20y 113 103 2 1 2 3 2 2 0 1 1 2 0 1 3 0 4.585

20+yr <20y 82 88 1 1 2 2 2 2 1 1 1 1 0 1 2 1 2.57

<20y 25-<30 108 99 2 2 2 1 3 3 0 1 1 2 0 1 2 0 3.155

<20y 25-<30 95 100 2 1 2 2 1 1 3.84

<20y <20y 96 101 2 1 2 2 2 3 0 1 1 1 0 1 2 0 2.98

<20y 25-<30 122 1 2 1 3 3 3 0 1 1 2 1 3.21

25-<30 25-<30 97 2 2 1 2 2 2 0 1 1 2 0 1 2 1 3.3

30-<35 30-<35 97 81 1 1 2 1 3 2 0 1 1 2 0 1 2 0 3.4

30-<35 <20y 83 98 1 2 2 2 1 1 1 1 0 1 1 2.9

30-<35 =35+ 114 109 2 1 2 3 3 2 2 1 1 2 0 1 3 0 3.8

<20y 25-<30 105 90 2 1 2 2 2 2 1 1 1 0 2 0 3.83

<20y <20y 97 2 2 2 2 2 0 1 1 1 1 2 2 1 4.12

=35+ =35+ 112 103 2 2 2 2 2 3 1 1 1 1 0 1 3 0 4.09

25-<30 <20y 111 108 2 2 1 2 1 2 0 1 1 2 0 1 2 0 3.245

<20y <20y 97 84 1 2 2 3 3 0 1 1 2 1 3.68

25-<30 25-<30 93 101 1 1 2 2 3 3 0 1 1 0 0 1 2 1 3.43

<20y 25-<30 114 109 2 2 1 2 2 2 1 1 1 2 1 3.71

<20y <20y 103 89 1 2 2 2 2 2 2 1 1 0 1 2 0 3.965

30-<35 30-<35 118 117 2 1 1 2 3 3 0 1 1 2 1 1 2 1 4.87

<20y 25-<30 106 94 2 2 2 3 3 1 0 1 1 2 0 1 3 1 3.655

20+yr <20y 118 2 2 2 3 3 1 0 1 2 1 1 3.75

<20y <20y 104 100 1 2 1 2 3 3 0 1 1 0 1 1 0 4.62

30-<35 97 88 2 1 1 2 1 2 0 1 1 2 0 1 2 3.83

25-<30 30-<35 89 98 2 1 1 3 3 3 0 2 1 2 0 1 1 0 3.88

30-<35 30-<35 111 99 1 2 2 1 2 1 2 2 1 0 0 1 1 2.146

20+yr <20y 107 87 1 2 1 2 2 2 2 1 1 1 3 1 1.928

<20y 30-<35 102 2 2 2 2 1 1 1 0 1 3.28

<20y 25-<30 114 100 2 2 2 3 3 1 1 1 1 0 1 1 1 3.18

20+yr 20+yr 83 1 2 2 1 2 1 0 1 1 1 0 1 0 3.06

<20y <20y 83 97 1 1 2 3 2 2 2 1 1 0 1 3.4

<20y 25-<30 122 111 2 2 1 3 2 2 0 1 1 2 0 1 2 0 3.02

<20y <20y 101 1 2 2 2 2 2 2 2 1 0 0 2 2 1 4

<20y 25-<30 105 114 2 1 2 2 3 1 0 1 1 2 0 1 2 0 3.26

<20y <20y 101 114 2 1 2 2 3 2 0 2 1 2 1 1 2 0 1.165

<20y 93 1 2 1 1 0 0 1 2 0 4

30-<35 =35+ 109 109 2 2 1 2 2 2 2 1 1 0 1 2 1 3.57

<20y 25-<30 115 108 2 2 1 2 2 2 0 1 1 2 0 2 2 0 4.245

<20y =35+ 99 85 2 1 2 2 2 2 0 1 1 0 1 1 2 1 3.405

30-<35 =35+ 125 103 2 1 2 1 2 0 1 1 2 0 1 2 1 3.68

30-<35 =35+ 104 1 2 1 2 1 3 0 1 2 0 0 1 2 3.41

30-<35 =35+ 117 75 2 1 1 1 1 2 0 1 1 0 0 1 3 1 3.38

25-<30 25-<30 96 96 2 1 2 3 3 3 0 1 1 2 0 1 3 0 3.4

<20y <20y 94 95 1 1 2 2 2 0 1 1 0 0 1 2 0 2.83

25-<30 30-<35 118 110 1 2 2 3 3 2 0 1 1 2 0 1 3 1 3.11

=35+ =35+ 99 95 2 2 1 2 2 2 2 1 1 1 0 2 2 1 2.77

<20y 25-<30 113 96 2 1 2 2 2 3 1 1 1 2 0 1 2 0 3.98

20+yr <20y 117 97 2 2 1 2 2 1 1 1 1 1 0 1 2 1 2.89

25-<30 25-<30 103 98 2 1 2 2 2 2 2 2 1 1 0 1 2 0 2.62

25-<30 30-<35 87 92 2 1 2 2 2 3 0 1 1 1 0 1 2 0 4.32

<20y 25-<30 94 105 1 1 1 2 1 3 0 1 1 0 0 1 2 0 3.66

<20y <20y 93 105 2 1 1 1 2 2 0 1 1 1 1 1 2 0 3.84

<20y =35+ 117 2 2 2 2 3 1 0 1 1 2 0 2 3 1 3.36

=35+ =35+ 104 109 2 1 2 3 3 3 0 1 1 1 0 2 3 0 3.85

30-<35 30-<35 111 95 2 1 1 2 1 3 0 1 1 0 0 1 2 0 3.39

20+yr <20y 86 105 2 2 2 2 1 2 1 1 0 0 1 3 1 3.51

20+yr <20y 106 1 2 2 2 2 2 0 2 1 1 0 1 2 1 3.74

<20y <20y 94 82 1 1 1 2 2 2 2 2 1 0 0 1 1 3.47

25-<30 25-<30 115 97 2 2 1 3 3 2 0 1 1 2 0 1 2 1 2.53

<20y <20y 108 1 1 1 1 2 1 0 1 1 1 0 1 2 0 3.1

25-<30 30-<35 106 90 2 2 2 2 2 3 0 1 1 0 0 1 2 0 3.86

<20y 98 1 2 2 2 1 2 2 1 0 0 1 3.52

25-<30 <20y 104 93 2 1 2 3 2 1 0 1 1 2 1 3.74

<20y <20y 104 1 2 1 2 2 1 1 1 1 0 0 1 0 4.33

30-<35 =35+ 110 117 2 1 2 3 3 3 0 1 1 2 0 1 2 0 3.19

<20y <20y 108 2 2 1 1 1 2 2 2 1 0 0 1 2 1 3.045

25-<30 25-<30 94 85 1 2 1 1 1 2 2 2 1 1 0 1 2 1 2.38

25-<30 25-<30 132 114 2 2 1 3 3 3 0 1 1 2 0 1 3 1 3.38

<20y <20y 87 87 2 2 1 1 2 0 1 1 0 0 2 1 0 3.94

=35+ =35+ 107 82 2 2 2 3 1 3 0 1 1 0 0 1 2 0 3.56
