# Supplementary material for: Do children born to teenage parents have lower adult intelligence? A prospective birth cohort study
Source: PLoS One. 2017 Mar 9;12(3):e0167395. doi: 10.1371/journal.pone.0167395 (PMC5344312; doi:10.1371/journal.pone.0167395)
Supplement: S1 Data — (PDF) [file pone.0167395.s005.pdf]

Supplementary file: input data

Data Dictionary

Variable name	Label	Value label
mage_FCV_5CAT	Maternal age at first clinic visit	13-19=0<br>20-24=1<br>25-29=3<br>30-34=4<br>35+=5
fage_FCV_5CAT	Paternal age (reported by mother at her first clinic visit)	13-19=0<br>20-24=1<br>25-29=3<br>30-34=4<br>35+=5
offiq21	peabody-standard score	
mumiq21	peabody-standard score	
samepart	same partner as birth of child (reported at 14y FU)	1 No<br>2 Yes
planned	Planned to get pregnant	1 No<br>2 Yes
sex	Child gender	1 Male<br>2 Female
mumedu	Maternal education at first clinic visit	1 Incomplete high<br>2 Complete high<br>3 Post-high
dadedu	Paternal education	1 Incomplete high<br>2 Complete high<br>3 Post-high
familyincome	Family income at first clinic visit	1 (<AU$10400)<br>2 (AU$10400-15599)<br>3 >(AU$15599)
mumsmoking	Smoking during pregnancy	0 Never smoked<br>1 1 to 9 cigarettes/day<br>2 10+ cigarettes/day
bingepreg	Binge drinking in pregnancy	1 No<br>2 yes

mumdep	Depression at first clinic visit	1 No<br>2 Yes
breastfeeding6	Breastfeeding (reported at 6-m FU)	1 Never<br>2 <4 months<br>3 4+ months
childattend	Child attended at preschool	0 Yes<br>1 No
Mother_child	**Mother-child interaction**	1 No<br>2 Yes
csmack	Physical punishment	1 Always<br>2 Sometime<br>3 Never
childbehab	**Explaining for child bad behaviour**	0 Always<br>1 Not always
**birthwet**	Birth weight in (kg)	

mage_FCV_5CAT	fage_FCV_5CAT	offiq21	mumiq21	samepart	planned	sex	mumedu	dadedu	familyincome	mumsmoking	bingepreg	mumdep	breastfeeding6	childattend	mother_child	csmack	childbehab	birthwet
=35+	=35+	111	103	2	1	1	2	2	2	0	1	1	2	0	1	2	0	3.38
<20y	25-<30	109	101	1	2	2	1	3	2	2	2	1	2	0	1		0	2.87
<20y	<20y	105	117	2	2	2	2	2	2	0	1	1	0	0	1	2		4.22
25-<30	30-<35	105		2	2	2	3	3	3	0	2	1	2	0	1	3	0	3.96
25-<30	30-<35	123	107	1	2	2	2	2	3	1	2	1	1	1	1	3	1	3.02
<20y	25-<30	117	87	2	2	1	3	3	3	0	1	1	2		1			3.64
<20y	25-<30	94	88	2	1	1	2	2	2	1	1		1	1	1	2	1	3.5
20+yr		105	97	1	2	1	2		2	0	1	1	2	0	1	2	0	3.74
<20y	<20y	100	86	1	1	2	2		1	1	2	1	1	0	1	2	1	2.96
20+yr	20+yr	104	95	2	2	2	2	1	1	0	2	1	1	0	1	2	1	3.755
20+yr	<20y	105	100	1	2	1	1			0	1	1	0	0	1	2	1	2.66
20+yr	<20y	113	92		2	1	1	2	1	0	1	1		0		2	1	4.28
<20y	<20y	87	101		2	1	2	3	1	0	1	1	2	0	2	2		3.3
<20y	<20y	94	97	2	1	1	1	3	3	0	2	1	1	0	1	2	0	3.14
<20y	25-<30	108		2	2	2	2	2	1	0	1	1	2	0	2	2	0	3.14
25-<30	30-<35	104	108		2	2	2	2	1	0	1	1	2		1			4.38
25-<30	25-<30	101		1	1	1	2	2	2	0	1	1	2	0	1	2	1	3.81
<20y	<20y	102	95	2	1	2	2	2	3	0	1	1	1	0	1	2	0	2.75
<20y	30-<35	103	98	2	1	2	1	3	1	0	1	1	2	0	1	2	1	3.5
25-<30	30-<35	93	101	2	2	1	1	2	1	0	1	1	2	0	1	3	1	3.71
<20y	<20y	105		1	2	1	2	2	1	1	1	1		0		3	1	4.39
25-<30	=35+	91	104	2	2	2	2	2	3	2	1	1	1	0	2	3	1	4.27
<20y	<20y	102		2	1	1	1	1	1	0	1	1	1	0	1	2	0	3.82
25-<30	25-<30	102	91	2	1	2	2	2	1	1	1	1	1	0	1	2	1	2.34
25-<30	30-<35	96		2	2	1	2	1	2	0	1	1	2	0	2	2	1	3.06
30-<35	=35+	91	83	2	1	1	1	3	2	0	1	1	2	0	2	3	0	3.18
<20y	25-<30	109	89		2	1	2	1	2	0	1	1	1	0	1	3	0	4.11

20+yr	30-<35	102		1	2	1	1		3	2	2	1	1	0	1		0	2.73
<20y	25-<30	101	104	2	1	2	3	3	3	0	1	1	2	0	1	3	0	3.08
<20y	25-<30	94	94	2	1	1	2	2	3	2	2	1	1	0	1	2	1	3.56
20+yr	25-<30	100	90	2	2	2	2	2	3	0	1	1	1	0	1	2	0	3.86
30-<35	30-<35	124		2		1	2	3	3	1	1	1	2	0	1	3	1	3.62
<20y	25-<30	123	110	2	2	1	3	2	3	1	1	1	2	0	1	2	1	2.69
20+yr	20+yr	118	115	1	2	1	2	2	3	0	1	1	1	0	1	2	1	3.47
<20y	25-<30	100	101	2	1	2	3	2	2	2	1	1	2	0	1	1	1	3.75
<20y	<20y	99	81	2	2	1	2	2	3	1	1	1	1	0	1	1		2.98
25-<30	=35+	100	87	1	2	1	3	2	2	1	1	1	1	0	1	2	1	3.63
25-<30	30-<35	84	87	2	2	2	2	2	2	0	1	1	1	0	1	2	1	5.05
25-<30	30-<35	105	100	2	2	2	2	2	2	0	1	1	2	0	1	2	0	3.64
<20y	25-<30	100	97	2	2	1	3	3	1	1	2	1	1	0	1	2	0	3.76
<20y	<20y	103	92	1	2	2	2	2	1	1	2	1	1	0	1	3	0	2.88
<20y	<20y	90	86	2	2	2	2	2	1	0	2	1		0	1	2	1	3.42
25-<30	25-<30	97	117	2	2	1	3	2	2	0	1	1	1	0	1	2	0	2.8
<20y	25-<30	114	94	2	1	1	3	3	1	0	1	1	2	0	1	2	0	4.29
<20y	<20y	111	94	2	2	1	2	2	2	0	1	1	1	0	1	2	0	3.425
25-<30	30-<35	107	103	2	1	1	3	3	3	0	1	1	2	0	1	2	0	3.96
20+yr	25-<30	101	96	2	2	2	1	2	1	0	1	1	0	0	1	2	1	3.07
20+yr	25-<30	102		2	1	1	2	2	2	1	1	1						2.62
20+yr	<20y	104	92	1	2	2	2	2	2	2	1	1	1		2	2	0	3.38
25-<30	=35+	102	117	2	2	2	3	3	2	0	1	1	1	0	1	2	1	3.65
25-<30	30-<35	88	86	2	1	1	2	1	2	1	1	1	0	0	1		0	3
<20y	25-<30	96	92	2	2	2	2	1	2	0	1	1	1	0	1		1	3.83
<20y	25-<30	80	96	2	1	1	2	1		1	1	1	2	0	1	2	1	2.375
20+yr	<20y	91	76	2	2	2	1	1	2	2	1	1	1	0	1	2	1	3.17
30-<35	=35+	104	105	2	2	2	2	2	2	0	1	1	2	0	1	2	1	2.93

30-<35	=35+	85		1	1	2	2	2	1	0	1	1		0		1	1	3.41
<20y	30-<35	103	98	1		2	1	1	2	0	1		2	0	1	2	1	2.57
25-<30	30-<35	118	98	2	1	2	2	2	2	0	2	1	2	0	1	2	1	3.09
30-<35	=35+	111	102	2	1	2	1	3	2	0	1	1	1	0	1		0	3.09
30-<35	<20y	118		2	1	2	3	3	1	0	1	1	2		1	2	0	4.2
<20y	25-<30	107	106	2	1	2	2	3	3	0	1	1	1	0	1	2	1	3.75
=35+	=35+	99		1	2	2	2	1	3	2	2	1	0		1			2.54
=35+	=35+	101	98	1	2	2	1	1	3	2	1	1	0	0	2	2	0	3.13
<20y	<20y	90	78	1	2	1	2	2		1	2	1	1	0	1	2	1	2.75
=35+	=35+	110		2	2	1	3	3	2	0	1	1	2	0	1	3	0	3.3
20+yr	<20y	107	91	1	2	1	3	3	1	0	1	1	0	0	1	2	0	
20+yr	<20y	94	88	1	2	2	2	1	2	1	1	2		0		2	1	2.38
20+yr	20+yr	103	87	2	1	1	1	1	1	0	1	1	1	0	1	2	1	3.99
20+yr	<20y	108	101	2	2	2	3	2	2	0	1	1	0	0	2	2	1	2.74
30-<35	=35+	114	81	1	2	1	1	2	1	0	1	1	2	1	1		0	3.5
20+yr	<20y	100	94	1	2	2	2	1	1	0	1	1	1	0	1	3	1	2.94
<20y	<20y	103	92	2	2	1	2	3	2	1	1	1	2	0	1	2	1	3.48
<20y	25-<30	104	88	1	2	1	1	2		2	1	1	1	0	1	2	1	3.12
20+yr	20+yr	114	95	1	2	1	2	2		1	1	1	1	0	1	1	1	3.73
25-<30	30-<35	105	76	2	1	1	1	3	2	0	1	1	2	0	1	2	1	2.85
25-<30	=35+	120		2	2	1	2		1	0	1	1	1	0	1	2	1	3.53
<20y	25-<30	111	110	1	1	1	3	3	1	0	1	1	2	1	1	1	0	3.47
<20y	25-<30	99	93	2	2	1	3	2	3	0	1	1	0	0	1	2	1	4.53
30-<35	30-<35	102	85	2	1	1	2	2	3	0	1	1	1	0	1	3	0	3.61
<20y	25-<30	85	97	2	1	2	2	2	2	1	1	1	1	0	1	2	1	3.06
20+yr	<20y	88	87	1	2	2	1	3	1	2	2	1	0		1			2.98
25-<30	25-<30	118	114	1	2	2	2	2	2	1	1	1	2	0	1	3	0	2.95
20+yr	<20y	110	86	1	2	1	1	1	2	2	1	2	1	0	2	2	1	3.29

<20y	25-<30	94	92	1	2	2	1	1	1	0	1	1	2	0	1	2	1	3.47
25-<30	25-<30	92	78	2	1	1	2	2	2	0	2	1	0	0	1	3	1	2.39
<20y	<20y	97	93	2	2	2	1	2	1	0	1	1	1	0	1	2	1	3.503
20+yr	<20y	110	109	2	2	2	2	2	1	0	1	1	2		1			3.35
25-<30	30-<35	120	106	2	1	1	2	3	2	0	1	1	2	0	1	2	0	3.02
25-<30	30-<35	108		2	1	1	3	3	1	0	1	2	1	0	2	3	1	3.885
<20y	25-<30	118	94	2	1	1	2	3	3	0	1	1	2	0	1	2	0	3.36
<20y	<20y	122	108	1	2	2	2	2	3	1	2	1	1	0	1	2	1	3.7
20+yr	20+yr	98		1	2	1	2	2	3	1	1	1			1			3.56
25-<30	30-<35	94	90	2	1	2	2	3	2	0	1	1	2	0	1		0	3.46
20+yr	<20y	97	101	2	2	1	2	2	1	2	1	1	1	0	1	2	0	3.12
25-<30	=35+	90	87	2	2	1	1	1	1	2	1	1	1		1			2.71
20+yr	<20y	114	103	2	2	1	3	3	1	0	1	1						3.39
25-<30	25-<30	117		2	1	1	2	2	1	2	2	1	0	0	1	2	1	3.06
=35+	=35+	94	91	2	1	2	1	1	2	0	1	1	1	0	1		0	2.46
25-<30	30-<35	103	88	2	2	2	1	1	2	0	1	1	2	0	2	2	0	3.1
<20y	<20y	110			2	2	1	2	2	2	1	1	1	0	1	2	0	3
25-<30	25-<30	85	103	2	1	2	2	2	3	1	1	1	1	0	1		1	3.25
<20y	25-<30	100	104	1	2	1	2	2	2	0	1	1	2	0	1	2	1	4.01
<20y	30-<35	107		2	1	2	2	1	3	0	1	1	2	0	1	2	1	3.8
=35+		111	96			2	3		1	2	1	1	0		1			4.075
<20y		85			2	2	1		1	2	2	1	0	0	1	3	0	3.42
<20y	25-<30	102		2	2	1	1	1	2	1	1	2	0	0	1	2	1	2.83
25-<30	=35+	122	91	2	1	2	2	2	2	0	1	1	2	0	1		1	3.9
<20y	30-<35	111		1	1	1	1	2	1	0	1	1	2	0	1	2	1	4.32
30-<35	25-<30	117	97	2	1	1	1	2	2	1	2	1	2	0	1	2	0	3.84
30-<35	25-<30	101	120	2	2	2	3	3	2	0	1	1	2	0	1	3	0	3.26
25-<30	=35+	115		2	1	2	3	1	1	0	1	1	2		2			3.6

20+yr	20+yr	91		1	2	1	2	1	1	0	2	1	1	0	1	2	1	3.255
25-<30	25-<30	108	98	2	1	1	2	2	2	0	1	1	1	0	1	2	1	3.82
25-<30	25-<30	100	91	2	1	1	2	2	3	0	2	1	2	0	1	2	1	3.435
<20y	25-<30	100	91	1	2	1	1	1	1	1	1	1		0	1	2	1	3.79
25-<30	30-<35	103	101	2	1	1	3	3	2	1	1	1	1	0	1	2	0	3.43
25-<30	30-<35	99	98	2	1	2	2	2	3	1	2	1	2	0	1	2	1	3.4
<20y	25-<30	99		2	1	2	3	2	2	2	1	1	2	0	1	2	1	3.155
25-<30	25-<30	102	88	1		2	1	1	2	0	1	1	1	0	1	2	0	2.26
25-<30	<20y	110	93	2	2	2	2	3	2	1	2	1	2	0	1	3	0	3.21
30-<35	=35+	110		1	2	2	3	1	2	1	2	1	2	0	1	2	0	3.5
<20y		110	90	2		1	2	2		0	2		1	0	1	3	1	4.35
25-<30	25-<30	110	106	2	1	2	2	3	2	0	1	1	2	0	2	3	0	3.25
<20y	25-<30	107	85	2	1	1	2	3	2	0	1	1	1	0	1	2	1	3.5
25-<30	<20y	86	86	1	2	1	2	2		0	1	1	1	0	1	2	1	3.86
25-<30	25-<30	100		1	2	1	2	2	1	0	1	1	1	0	1	2	0	3.24
<20y	25-<30	103	88	1	1	2	2	2	2	0	2	1	0	0	2	2	1	3.6
<20y	<20y	97		2	1	1	1	1	1	2	1	2	1	0	2	2	1	2.68
25-<30		104	93		1	2	3	2	1	0	1	1	2	1	1	2	1	3.34
25-<30	30-<35	111	110	2	1	2	2	1	1	0	1	1	2	0	2	2	1	3.46
30-<35	30-<35	102	109	2	2	2	2	2	2	0	1	1	0	0	1			3.77
<20y	25-<30	101	88		2	1	1	2	2	2	1	2	1	0	1	2	0	3.27
<20y	30-<35	113	100	2	1	2	2	2	3	2	1	1	1	0	1	1	0	3.58
25-<30		103	98	1	1	2	3		1	0	1	2	2	0	1	3	1	3.48
20+yr	<20y	95	77	2	1	1	1	2	1	2	2	1	1		1			3.51
30-<35	30-<35	109		1	1	2	2	2	2	0	1	1	2	0	1	1		3.34
20+yr	<20y	122	109	2	2	2	2	2	3	0	1	1	1	0	1	2	0	3.26
<20y	<20y	101		1	2	1	2	2	1	1	2	1	0	1	1	2	0	2.95
20+yr	=35+	101	100	1	1	1	2	2	1	0	1	1	0	0	2		0	2.96

25-<30	<20y	111	96	1	1	2	2	1	3	0	1	2	2	0	2	2	0	3.285
<20y	<20y	80		1	2	1	1	1		0	1	1	0		1			3.53
25-<30	25-<30	102		1	1	2	2	2	1	0	1	1	1	0	2	1	0	3.5
<20y	<20y	99	92	2	2	1	2	2	3	0	1	1	0	0	1	3	1	3.65
25-<30	25-<30	97		2	2	1	2	2	3	2	1	2	0	0	2	2	0	3.9
25-<30	25-<30	103	96	2	1	1	2	2	1	0	1	2	1	1	2	1	0	2.75
30-<35	=35+	110	98	2	1	2	2	2	2	0	2		1	0	2		0	2.8
30-<35	30-<35	110	117	2	1	1	3	3	1	0	1	1	2	0	1	3	0	3
25-<30	25-<30	111		2	1	2	2	2	1	0	1	1	2	0	1	2	1	3.93
25-<30	25-<30	100	108	1	1	2	2	2	2	1	1	1	2	0	1	3	1	3.71
30-<35	30-<35	117	94	2	1	1	2	2	2	2	1	1	2	0	1	3	0	3.62
<20y	25-<30	100	86		2	2	2	2	1	0	1	1	2	0	1	2	1	3.56
=35+	=35+	84			1	2	2	1	1	0	1	1	0	0	1	3	1	3.04
<20y	25-<30	109		2	1	2	3	2	2	0	1	1	2	0	2	2	1	2.97
25-<30	25-<30	110	92	2	1	2	2	2	3	0	2	1	1	0	1	2	1	4.11
<20y	25-<30	89	95	1	1	1	2	2	2	0	2	1	1	0	1	2	1	3.18
30-<35	30-<35	115	117		2	1	2	3	3	2	2	1	1	1	1	2	0	3
30-<35	30-<35	97	100	1	2	2	2	2	2	0	1	1	1	0	1	3	0	3.71
30-<35	=35+	103	97	2	2	2	1	2	3	0	2	1	1	1	1	2	0	3.19
25-<30	25-<30	111		1	2	1	1	2	2	0	1	1	1	0	1	2	1	4.08
<20y		100	91	2	2	2	2	2	1	0	1	1	2	0	1	2	0	2.56
25-<30	30-<35	100	84	2	2	2	2	1	2	2	1	1	2	0	1	2	1	3.25
20+yr		107	112	1	2	2	3		1	2	1	2	1	0	1	3	0	2.79
25-<30	25-<30	115	101	2	1	1	2	2	2	0	1	1	2	0	1	2	0	3.36
20+yr	25-<30	96	82	2	2	2	1	3	2	2	1	1	0	1	1	2	1	2.83
20+yr	25-<30	110		2	1	2	2	2	1	0	1	1	1	1	1	1	0	3.37
<20y	25-<30	115	98	2	1	2	2	2	3	0	2	1	1	1	1	2	1	3.68
=35+	=35+	117	109	1	2	2	2	3	1	0	1	1	2	0	1	3	0	3.8

25-<30	30-<35	110	108	2	1	1	3	3	1	2	1	1	2	0	1	2	0	3.98
<20y	25-<30	108		2	1	2	3	2	3	0	2	1	1	0	1		1	3.65
20+yr	25-<30	94		2	2	2	1			2	1	1		0		2	0	3.02
<20y	<20y	112	97	2	2	1	3	3	1	0	1	1	1		1			3.73
20+yr	<20y	117		2	2	1	2	2	1	1	1	1	1	1	1	2	1	4.43
25-<30	25-<30	114	106	2	1	1	3	3	3	0	1	1	0	0	1	2	0	3.8
20+yr	<20y	89		2	2	2	2	2	1	1	2	1	2	0	1		0	2.99
20+yr	25-<30	120	107	2	2	1	2	2	3	0	1	1	2	0	1	3	0	2.92
25-<30	<20y	112	101	2		2	1	2	1	2	1	1	2	0	1	1		2.5
25-<30	25-<30	100		2	1	1	2	3	3	2	1	1	2	0	1	2	0	3.48
<20y	25-<30	101	93		2	1	2	2	1	0	1	1	2	0	2	3	1	3.83
<20y	25-<30	107	106	2	2	1	2	2	2	2	2	1	2	0	1	2	0	4.32
<20y	<20y	104	96	2	2	2	2	2	2	0	1	1	2	0	1	2	0	3.925
25-<30	25-<30	100	94	2	2	1	2	2	2	0	1	1	1	0	1	2	0	3.9
20+yr	20+yr	105	101	1	2	2	2	2	1	2	1	1	1		2			2.67
<20y	<20y	108	95	1	1	1	2	2	1	1	1	1	2	0	1	3	1	3.43
<20y	25-<30	103	90	1	2	1	1	1	1	0	1	1	2	1	1	2	0	4.34
<20y	25-<30	91	101		1	1	1	2	1	0	1	1	1	0	1	2	1	3.085
<20y	25-<30	80	112	2		1	2	1	1	1	1	1	1	1	1	2	0	2.805
20+yr	20+yr	78	82	2	2	2	1	1	2	2	1	1	0	0	1	2	1	2.86
25-<30	<20y	99	93	2	2	1	1	1	1	0	1	1	0	0	1	2	0	2.99
<20y	=35+	100		2	2	2	1	1	2	1	1	2	2	0	1	3	0	4.08
<20y	30-<35	97	96	2	2	2	2	2	1	0	1	1	0	0	1	3	0	3.17
<20y	30-<35	90	98		2	2	3	1	2	0	2	1	0		2			2.97
25-<30	25-<30	98	83	2	1	2	2	2	3	1	1	1	1	1	1	2	0	2.86
25-<30	25-<30	105	96	2	2	2	2	3	1	0	1	1	0	0	1	2	0	3.31
20+yr	20+yr	99		1	2	2	2	2	1	2	1	1	1	0	1	2	0	3.34
20+yr	<20y	107			2	2	3	2	1	2	1	1	1	0	1	2	0	2.7

25-<30	30-<35	94	93	2	2	2	2	2	3	1	2	1	1	0	1	2	0	3.28
<20y	<20y	94	95	1	2	1	2	2	1	0	2	1	1	0	1	2	1	3.09
30-<35	=35+	110	120	1		2	2	1	1	0	1	1	1	0	2		0	4.13
25-<30	25-<30	122	114	2	1	1	3	2	1	0	1	1	2	0	1	2	0	3.65
30-<35	25-<30	99		2	2	1	2	2	3	1	1	2	2	0	1	1	0	3.07
<20y	25-<30	100		1	1	2	2	2	3	0	2	1	2	0	1	2	0	3.63
=35+	=35+	108	88	2	2	2	2	2	1	0	1	1	2	1	1	2	0	3.25
25-<30	30-<35	102	95	2	1	1	3	3	3	2	1	1	1	0	1	2	1	3.16
<20y	25-<30	88	86	2	1	1	2	2	1	0	2	1	1	0	2	2	0	3.4
30-<35	=35+	99	99	2	1	2	3	2	2			1	2	0	1	2	0	3.62
25-<30	25-<30	109	108	2	2	2	2	1	3	0	1	1	2	0	2	2	1	3.83
25-<30	25-<30	122	119	2	2	1	2	3	2	0	1	1	2	1	1	1	1	2.82
<20y	25-<30	110	90	2	2	1	1	2	2	0	1	1	1	1	1	1	1	3.37
20+yr	<20y	106	99	2	2	2	3	3	2	1	1	1	2	0	1	2	0	3.76
25-<30	30-<35	96	103	2	2	1	2	2	1	2	1	1	0	1	1	3	0	3.38
30-<35	=35+	100	93	2	2	1	3	2	3	0	1	1	2	0	1			2.94
20+yr	<20y	90		1	2	2	1	1	1	2	1	2	2	0	1	2	1	3.43
=35+	30-<35	103		1	2	1	1	1	1	2	1	1	0	0	1	2	0	2.94
<20y	<20y	104	104	2	1	2	2	1		0	2	1	1	0	1	2	1	3.57
25-<30	=35+	82	100			2	1	1	3	2	1	1	2	0	1	2	0	2.52
20+yr	<20y	84	111	1	1	2	3	1	1	1	2	1	1	0	1	3	0	2.93
25-<30	<20y	110	94	1	2	2	3	3	2	0	1	1	0	0	1	2	1	3.14
<20y	25-<30	108	100	2	1	2	2	2	3	2	1	1	2	0	2	2	0	3.04
20+yr		99	96	1	2	2	2	2	1	1	1	1	0	0	1	2	1	3.33
<20y	<20y	107	97	2	2	2	2	2	1	2	1	1	1	0	1	2		3.34
<20y	25-<30	111		1	1	2	2	2	3	0	1	1	0	0	1	2	1	3.84
<20y	25-<30	93	86	2	1	1	1	2	2	0	2	1	2	0	1	2	1	3.17
<20y	30-<35	106	114	1		1	3	1	2	0	1	1	2	0	1	2	0	4.6

30-<35	30-<35	102	90	1	2	2	2	2	2	0	1	1	1		1	3	1	3.87
30-<35	30-<35	101	81	2	1	1	2	3	2	0	1	1	1	1	2	3	1	3.6
25-<30	25-<30	102		2	1	1	3	3	2	0	1	1	2	0	1	3	0	2.43
<20y	<20y	114	103	2	1	2	2	2	3	0	1	1	2	0	1		1	3.125
25-<30	30-<35	94	100	2	2	2	2	2		0	1	1	1	1	1	2	1	3.4
<20y	25-<30	103	108	2	1	1	2	2	2	0	2	1	2	0	1	2	0	3.83
25-<30	=35+	107	89	2		1	1	2	2	0	1	1	2	0	1	2	0	3.87
25-<30	25-<30	101		2	2	2	3	2	2	0	1	1	2	0	1	2	0	3.66
20+yr	<20y	89		2	2	1	2	2	2	0	1	1	0	0	2	2	1	3
=35+	30-<35	104		2	2	2	3	3	2	0	1	1	1	0	1	3	0	3.63
25-<30	30-<35	103	105	2	2	1	2	2	2	0	1	1	0		1			4.43
<20y	<20y	111	96	2	2	2	2	1	2	0	1	1	2		1	1	1	3.17
25-<30	25-<30	103	81	2	1	2	1	1	1	2	2	1	1	0	2	2	1	3.62
<20y	25-<30	104	93	2	1	2	2	2	1	0	1	1	0	0	1	2	1	3.24
25-<30	30-<35	105	98	2	1	1	2	2	2	0	1	1	1	1	1	2	0	4.49
<20y		89		2	1	1	2	1	1	0	1	1	0	0	1	2	0	2.835
25-<30	30-<35	117		2	2	2	3	3	3	0	1	1	2	0	1	2	0	3.5
<20y	<20y	94	84	2	2	2	2	2	3	1	2	1	1	0	1	2	0	2.65
25-<30	30-<35	109	98	2	2	1	2	2	1	0	2	1	0	0	1	2	1	3.18
<20y	30-<35	103	87	2	1	2	2	2	2	2	2	2		1		3	0	3.38
25-<30	30-<35	93	96	2	1	2	2	1	2	0	1	1	0	1	1	2		3.44
25-<30	25-<30	110	106	2	2	2	2	2	2	0	1	1	2	1	1	2	1	3.37
25-<30	30-<35	94	119	2	2	1	3	3	2	0	1	1	2	0	1	2	0	3.95
25-<30		106	93	2	2	1	2			0	1	1	0	1	2	2	0	4.25
20+yr	<20y	107	115	2	2	1	2	2	2	0	1	1	2	0	1	2	0	3.08
25-<30	30-<35	98	101	2	1	1	1	2	3	2	2	1	1	0	1	2	0	4.71
20+yr		115		1	2	2	2			0	1	1	1	0	1	3	0	3.03
<20y	25-<30	112	106	2	1	2	2	2	2	0	1	1	1		1	2	0	3.46

20+yr	<20y	102	83	2	2	1	2	2	1	1	2	1	0	0	1	1	0	2.4
30-<35	30-<35	109	88	2	2	1	2	1	2	0	1	1	2	0	1	2	0	3.26
<20y	<20y	100		2	2	1	2	1	2	1	1	1	1	0	1	2	0	4.03
30-<35	=35+	115	107	2	2	2	2	2	2	0	1	1	0	0	1	2	0	3.71
25-<30	30-<35	114	98	2	1	1	1	2	3	0	1	1		1		2	0	3.34
25-<30	=35+	99	92	1	1	1	2	1	2	0	1	1	0	0	1	2	0	3.25
20+yr	<20y	108	99	2	2	1	2	2	2	2	1	1	0		1			3.71
<20y	<20y	104	89	2	1	1	2	2	2	0	1	1	1	0	1	3	0	3.87
25-<30	30-<35	108	96		1	1	2	2		0	1	1	2	1	1	3	0	3.44
<20y	<20y	100	92	2	2	1	2	2	1	0	1	1	2	0	1	2	0	3.38
<20y	<20y	105	106	1	1	2	3	1	2	0	1	1	1	0	1	2	1	3.27
<20y	25-<30	98	101	2	2	2	3	2	2	0	1	1	2	0	1	2	1	3.49
25-<30	25-<30	110	104	2	2	2	3	2	2	0	1	1	1	0	1	2	1	3.24
25-<30	25-<30	101	100	2	1	2	2	2	1	1	2	1	2	0	2	2	1	2.54
30-<35	=35+	102	97	2	2	2	2	2	2	2	2	1				2	0	3.96
25-<30	25-<30	122	90	2	1	1	2	2	2	0	2	2	1	0	2	2	1	3.97
30-<35	30-<35	123	103	2	1	2	3	2	2	0	2	1	2	0	1	2	1	4.32
25-<30	=35+	85	86	2	1	1	1	1	2	0	1	1	2	0	1	2	0	3.88
30-<35	30-<35	115	109	2	2	2	3		2	0	1	1	1	1	1	1	0	4.32
30-<35	=35+	102	99	2	2	1	3	1	2	0	1	1	2	0	1	2	1	1.98
30-<35	=35+	109	114	2	2	2	2	3	2	0	1	1	1	0	1	3	0	3.67
25-<30	30-<35	81			2	2	2	2	2	2	2	1	2	0	1	2	1	3.77
30-<35	<20y	115	107	2	1	2	2	2	3	2	1	1	1	1	1	2	1	3.305
25-<30	30-<35	105	100	2	2	2	3	3	3	0	1	1	0	0	2	2	0	3.03
30-<35	=35+	111	80	2	2	1	3	3	1	0	1	1		1	2	3	0	3.02
25-<30	30-<35	109	98	2	1	2	3	3	3	0	1	1	2	0	1		0	3.29
25-<30	30-<35	110	98	1	2	2	1	2	1	0	1	1	2		1		1	3.48
25-<30	25-<30	104	101	2	2	2	2	2	3	1	1	1	2	0	1	3	0	2.96

25-<30	25-<30	96	91	1	2	1	2	1	2	0	1	1	2		2	1	0	3.46
<20y	25-<30	103	92	2	1	1	2	1	2	0	1	2	1	1	1	2	1	3.82
25-<30	25-<30	114	81	2	2	2	2	1	1	2	2	1	1	0	1		0	2.74
25-<30	25-<30	100	94	1	1	1	2	2	2	0	1	1		0		2	0	3.51
30-<35	=35+	118	102	2	1	2	3	2	2	0	1	1	2	0	1	3	1	3.08
30-<35	25-<30	120	147	2	1	2	3	3	1	0	1	1	2	0	1	2	0	3.51
25-<30	30-<35	100	108	2	1	1	2	1	2	0	1	1	2	0	2	2	0	3
<20y	<20y	102	91	2	1	2	2	2	1	0	1	1	1	0	1	2	0	3.42
<20y	25-<30	83	80	2	1	1	2	2	3	1	2	1	1		1			2.98
<20y	30-<35	105	117	1	2	2	3	2	1	0	1	1	2	1	2	2	1	3.36
30-<35	=35+	125		2		1	2	2	2	0	1	1	2	0	1	3	0	3.28
20+yr	<20y	108		1	1	2	2	2	1	0	1	1	2	0	1	2	0	3.17
25-<30	25-<30	100	92	2	2	2	2	2	2	0	1	1	2	0	1	3	1	3.1
30-<35	30-<35	101	98	2	2	2	2	2	2	0	2	1	1	0	1	1	1	3.19
<20y	<20y	109	88	1	2	1	3	2	1	0	1	1	1	0	1			3.32
=35+	=35+	92	95	2	2	2	2	1	2	0	1	1	0	1	1	2	0	4.55
30-<35	=35+	114	114	1	2	2	2	3	3	1	1	1	2	0	1	2	0	2.9
25-<30	30-<35	122	117	2	1	1	3	3	3	0	1	1	2	0	1	3	0	4
25-<30	25-<30	109	103	2	2	1	3	3	2	0	1	1	1	0	1	2	0	3.72
<20y	30-<35	92		2	1	1	2	2	2	1	2	1		0		2	1	3.16
<20y	30-<35	98	96	2	2	1	2	2	2	0	1	1	2	0	1	3		3.35
<20y	<20y	93	85	2	2	2	1	2	1	1	1	1	0	1	1	2	0	3.97
<20y	<20y	100	112	2	2	2	3	3	3	0	1	1	2	0	1	2	0	3.02
30-<35	=35+	91		2	2	2	1	2	2	2	1	1	0		1			3.41
30-<35	30-<35	108	103	1		2	3	2	1	0	1	1	1		1			2.49
20+yr	<20y	96	88	2	2	1	2		1	0	2	1	2	0	1	2	1	3.49
<20y	<20y	111	92	2	1	1	3	2	3	0	1	1	2	0	1	2	1	3.39
25-<30	25-<30	120	95	2	1	1	1	2	2	0	1	1	2	0	1	2	1	3.51

25-<30	25-<30	105		2	1	2	2	2	2	0	1	1	2	1	1	2	1	3.53
<20y	<20y	83		1	2	1	2	2	2	1	1	1	0		2			3.71
25-<30	30-<35	117	112	1	2	1	2	2	3	0	1	1	1	0	1	2	0	3.75
20+yr	20+yr	97	100	2	1	2	2	3	1	0	1	1	2	1	1	2	1	4.38
30-<35	=35+	100	103	2	1	1	3	1	2	0	1	1	2	1	2	2	1	4.51
<20y	<20y	108	100	2	1	1	2	2	3	0	1	1	2	0	1	2	0	3.565
25-<30	25-<30	105		2	1	2	2	3	3	0	1	1	2	0	1	1	1	3.48
25-<30	25-<30	115	94	2	2	1	2	2	2	0	1	1	0	0	1	2	1	3.07
<20y	25-<30	89	98	2	2	2	2	2	2	0	1	1	0	0	1	2	0	3.195
30-<35	30-<35	98		2	2	2	1	2	2	0	1	1	1	1	1	3	1	3.1
25-<30	25-<30	83	92		2	2	2	1	2	2	2	1	2	0	1	2	1	4.11
25-<30	30-<35	107	97	2	2	1	3	1	2	0	1	1	2	0	2	2	1	4.12
30-<35	=35+	106	95	2	1	1	3	1	3	0	1	1	2	1	2	2	0	3.78
30-<35	30-<35	134	99	2	2	1	2	2	2	2	2	1	2	0	1	2	0	2.45
30-<35	25-<30	84	84		1	2	1	2	1	0	1	1	1	0	1	2	0	3.18
30-<35	30-<35	110	107	2	2	1	2	2	2	0	1	1	2	0	1	2	0	4.3
<20y		97	95	2	1	2						1	1		2	2	0	3.04
<20y	30-<35	103		2	2	2	2	2	3	0	1	1	1		1			3.45
<20y	<20y	104	93	1	2	2	2	3	3	0	1	1	2	0	1	2	0	4.25
30-<35	=35+	90	105	2	2	2	2	2	1	0	1	1	2	0	1	2	0	3.503
<20y	25-<30	118	110	2	2	1	2	3	2	0	1	1	2	0	1	3	0	3.36
20+yr		110	104	1	2	1	2		2	1	1	1	1	0	1	2	0	3.31
30-<35	=35+	115	107	2	1	2	2		1	0	1	1	0	0	1	2	0	3.3
<20y	25-<30	92	85	2	2	2	1	2	3	1	1	1	0	0	1	2	1	3.27
<20y	25-<30	115		1	2	1	3	2	2		1	1	1	1	1	2	0	3.68
20+yr	20+yr	107		1	2	1	2	2	2	0	1	1	2	0	1	2	0	3.42
<20y	<20y	106		2	1	2	2	2	2	0	1	1	2		1			3.17
<20y	25-<30	94	92	2	1	1	2	2	1	0	1		2	0	1	2	0	3.36

<20y		105	88	1	2	1	2	2	1	2	1	1	2	1	1		1	3.105
30-<35	=35+	117	87		2	1	1	2	2	0	1	1	2	0	2	3	1	3.92
<20y	<20y	97	81	2	1	2	1	1	2	0	1	1	0	1	1	2	1	3.01
20+yr	<20y	111			2	2	2	2	1	0	1	1	0	1	1			2.9
<20y	25-<30	104		2	2	1	2	2	2	1	2	1	2		1			3.245
<20y	<20y	110	98	2	2	2	2	2	3	0	2	1	2	0	1	2	0	2.62
25-<30	30-<35	103	81		2	1	3	3	3	0	1	1	1	0	1	2		4.16
<20y	<20y	105	89	2	1	1	2	2	2	0	1	1	1	0	1	2	0	2.54
<20y	<20y	100		2	1	2	2	2	2	0	1	1		0		2	1	3.3
30-<35	30-<35	90	89	2	2	2	2	2	1	2	1	1	1	1	1	2	0	3.44
=35+	=35+	101	88	1	2	1	1	1	1	2	1	2	0	0	2		0	3.37
<20y	<20y	87	83	1	1	1	1	2	1	1	2	1	1	1	2	2	1	3.81
20+yr	<20y	102	88	2	1	2	2	2	2	0	1	1	0	0	1	2	1	3.64
<20y	<20y	111		1	2	2	2	2		2	1	2	0	0	1	2	1	2.68
25-<30	25-<30	127	122		2	1	2	2	3	0	1	1	2	0	1	2	0	3.65
<20y	25-<30	87	84	1	1	1	1	1	1	2	1	1	1	1	1	3	0	3.39
25-<30	30-<35	94	81	1	1	2	1	3	2	2	2	1	1		1			3.31
25-<30	30-<35	114	103	2	1	1	2	2	2	0	1	1	2	0	1	2	1	3
<20y	<20y	101	90		1	2	3	3	1	2	2	1						3.39
25-<30	=35+	88	94	2	1	2	2	2	2	0	1	1	2		2			3.49
<20y	25-<30	80	52	1	2	2	3	3	1	0	1	1	0	0	1	3	0	4.24
<20y	<20y	117	87	1	2	2	2	2	1	1	2	1	0	1	1		0	3.27
<20y	25-<30	123	101	2	1	2	2	3	1	0	1	1	0	1	1			3.475
<20y	30-<35	91	86	2	2	1	2	1	1	0	2	1	1	0	1	2	1	3.48
25-<30	25-<30	103		2	2	2	2	3	3	0	1	1	2	0	1	2	1	4.17
20+yr	<20y	96	101		1	2	2	2	2	1	1	1		0		2	1	3.28
25-<30	25-<30	122	104	2	1	1	2	2	2	2	2	1	2	0	1	2	1	3.3
20+yr	20+yr	115		1	2	1	2	2	1	0	1	1	2	1	1	2	0	3.5

20+yr	25-<30	113		2	2	2	2	2	1	0	1	1	2	0	2	2	0	3.14
20+yr	<20y	115		2	1	1	2	3	1	0	1	1		1				3.43
<20y	25-<30	88		2	1	1	2	2	2		1	1	1	0	1		0	2.8
25-<30	25-<30	114	92	2	1	1	1	1	1	0	1	1	1		2			3.58
<20y	<20y	99	87	2	2	2	2	1	1	2	2	1	0	1	1	2	1	3.69
20+yr		102	96	1	2	2	1		1	0	1	1	2		1			3.13
=35+	=35+	82	90	2	2	1	2	2	3	0	1	1		0		2	1	3.24
<20y	25-<30	92	88	2	2	1	2	2	2	1	2	1	1	1	1	2	1	2.78
=35+	=35+	105		2	2	1	2	1	3	2	2	1	2	1	1		0	2.82
=35+	=35+	103	99	2	2	2	2	2	2	0	1	1	1	0	1	3	0	4.37
<20y	25-<30	98	93	2	2	2	3	3	3	0	1	1	2	0	1	3	1	3.3
<20y	25-<30	111	101	1	2	2	1	2	1	1	1	1	1		1			3.61
<20y	<20y	94	83	2	1	1	2	2	1	0	1	1	2	0	1	1		3.49
25-<30	25-<30	102	108	2	2	2	2	3	2	2	1	1	1	0	1	2	1	2.79
25-<30	25-<30	105		2	2	2	2	2	2	0	1	1	2	0	1	2	0	3.08
25-<30	25-<30	114		2	2	2	3	3	2	2	1	1	2	0	1	2	0	2.94
25-<30	25-<30	113		1	1	1	3	3	2	0	1	1	1	1	1	2	0	4.15
30-<35	30-<35	104	102	1	2	2	1	1	2	1	1	1	0	0	1	1	0	2.95
20+yr	20+yr	92	111	2	1	1	3	2	2	0	1	2	2		1			3.47
25-<30	25-<30	100	89	2	2	2	2	2	2	0	1	1	2	0	2	1	1	2.96
25-<30	30-<35	106		2	1	1	3	2	3	0	1	1	2	1	1	3	0	3.86
25-<30	30-<35	112	95	2	2	1	2	2	1	0	1	1	1	0	1	2	0	3.66
<20y	25-<30	108	100	2	2	2	3	3	2	0	1	1	2	0	1	2	0	3.94
25-<30	30-<35	103	103	1	2	2	2	1	3	2	2	1	0	1	2		0	2.92
<20y	25-<30	81		1	1	2	1	2	1	0	1	1	0	0	1	3	1	3.14
25-<30	25-<30	107	89	2	2	2	3	2	2	0	2	1	2	1	2	2		2.98
25-<30	30-<35	111			1	2	3	3	2	0	2	1	2		2			3.31
20+yr	<20y	98	115	1	2	2	3	2	3	1	1	1	1	1	1	3	1	2.83

25-<30	25-<30	108	96	2	2	2	1	1	1	0	1	1	1	0	1		0	3.38
25-<30		102		2	1	1	1	3	2	0	1	1	2	0	1	2	1	3.35
<20y	25-<30	106		2	2	1	2	1	1	0	2	1	2	1	2	3	1	3.44
=35+	=35+	87	93	2	1	2	1	1	2	0	1	1	1	0	1			3.64
<20y	<20y	104	88	2	2	1	1	1	1	1	2	1	1	1	1	2	1	3.23
20+yr	<20y	91	96	1	2	2	1	1	1	1	2	1	0	1	1	2	0	3.33
25-<30	30-<35	81	88	2	2	1	2	1	1	1	1	1	1	1	1	2	1	3.38
25-<30	30-<35	111	98	2	2	1	3	1	1	0	2	1	2	1	1	2	1	3.355
30-<35	<20y	108	106	1	2	2	2	2	2	0	1	1	0	0	1	2	1	2.86
25-<30	=35+	103		1	2	2	3	3	3	0	1	2	2	1	2	2	1	3.2
<20y	25-<30	99	98	2	1	2	2	3	2	0	1	1	2	1	1	3	1	3.76
20+yr	<20y	76	81	2	2	1	1	2	1	0	1	1	1	0	1	2	1	3.96
=35+	30-<35	124	103	2	1	1	2	3	1	0	1	1	1	0	2	2	0	3.5
20+yr	<20y	100		1	2	1	3	3	2	1	2	1	2	1	1	2	0	2.93
<20y	<20y	102	84	2	2	1	1	2	1	1	2	1	0	1	1	2	1	3.73
<20y	<20y	88		2	1	2	1	1	1	0	2	1	0	0	1	2	1	2.78
<20y	<20y	114			2	1	3	2	3	0	1	1	2	0	1	3	1	3.6
<20y	25-<30	105	108	2	2	2	3	3	2	0	1	1	2	0	2	2	0	3.38
25-<30	30-<35	105			1	1	1	3	2	0	1	1	2	0	1	3	1	3.405
25-<30	30-<35	101		2	2	1	2	2	2	0	1	1	1	1	1	2	1	3.33
25-<30	30-<35	115	86	2	2	1	2	2	1	2	2	1	1		2			3.29
<20y	<20y	110		2	2	1	3	2	1	0		1	0	0	1	2	1	3.78
20+yr	20+yr	101		1	2	1	2	3		0	1	1	1	1	1	2	0	4.56
25-<30	25-<30	80	96	2	2	2	2	1	1	0	2	1		1		2	0	3.44
=35+	=35+	114	91	2	2	1	1	2	1	0	1	1	2	0	2	2	1	3.62
<20y	25-<30	112	100	2	2	2	2	3	3	2	1	1	2	1	1	2	0	3.29
<20y	25-<30	85	93	2	2	1	1	1	1	2	1	1	1	0	1	2	1	3.57
<20y	25-<30	104	94	2	2	2	3	2	3	0	2	1	2	1	1	2	1	3.11

<20y	<20y	105		1	1	1	2	2	3	0	1	1	0	0	1	3	0	3.09
20+yr		84	92	2	2	1	2		1	0	1	2	0	0	1	1	1	3.39
20+yr	<20y	103	98	2	2	1	2	2	1	2	2	1	1	0	1	2	1	3.8
25-<30	25-<30	114	104	2	1	2	1	2		0	1	1	0	1	1	3	0	3.45
25-<30	25-<30	101	90	2	1	2	2	2	2	0	1	1	2	0	1	2	0	3.84
<20y	25-<30	114		2	2	1	3	3	2	0	2	1	2	0	2	2	0	3.37
<20y	<20y	96	93	1	1	2	2	2	1	1	2	1	0		1			3.74
<20y		86	104	1	2	2	2		1	1	1	1	1	0	2	2	0	3.91
25-<30	25-<30	86	88	2	1	2	3	3	2	0	1	1	2	0	1	2	1	2.73
20+yr	<20y	104		2	2	2	1	2	2	0	2	1	1	1	1	2	1	3.63
<20y	25-<30	85	82	2	2	1	2	2	2	1	1	1	1	0	2	2	1	3.34
<20y	<20y	79	85	2	2	1	2	2	2	0	1	1	0	0	1	3	0	3.14
30-<35	=35+	103	100	1	2	1	1	1	1	2	2	1	1	0	1	2	1	2.81
30-<35	=35+	110	98	2	1	1	2	1	1	2	2	1	0	0	1		0	2.685
<20y	25-<30	99		2	1	1	2	2	2	0	1	1	2	0	1		0	3.485
<20y	25-<30	99		2	2	1	3	2	1	0	1	1	1	0	1	2	0	3.73
25-<30	25-<30	107	93	2	1	2	2	3	1	2	1	1	2	0	1	2	0	2.66
30-<35	30-<35	114		2	1	2	2	2	2	0	1	1	2	1	1		1	3.63
30-<35	25-<30	117	95	1	1	1	1	1	3	1	2	1	0		1			3.53
30-<35	25-<30	104	94	1	2	1	3	2	2	1	1	1	1	0	1	2	1	2.48
20+yr	25-<30	97			2	2	2	2	2	1	1	1	0	1	1	2	1	3.31
30-<35	30-<35	102	91	2	1	2	2	1	1	0	1	1	2	0	1		0	3.33
25-<30	25-<30	107		2	1	2	2	2		0	1	1	0	1	1	2	0	3.52
25-<30	30-<35	112		2	2	2	3	3	2	0	1	1	2	1	1	2	0	3.08
30-<35	=35+	122	102	1	2	1	3	1	3	2	1	1	1	0	1	3	1	3.1
25-<30	25-<30	82	89	2	2	1	2	2	2	2	2	1	2	1	2	2	1	3.27
<20y	<20y	99	97	2	1	2	2	2	2	0	1	1	0	0	1		0	3.78
<20y	25-<30	97		2	2	2	2	2	1	0	2	1	1	1	1	2	0	3.19

30-<35	=35+	94	98	1	2	1	2	2	2	0	1	1	2	0	1	2	1	3.74
<20y	30-<35	112	92	1		1	2	3	3	0	1	1	1	1	1	3	0	4.07
<20y	<20y	98	89	2	2	2	2	3	3	0	1	1	2	1	1	1	0	4.12
30-<35	25-<30	114		2	2	2	3	3	3	2	1	1	1	0	1	2	1	3.49
25-<30	=35+	111	94	1	1	2	1	1	2	0	1	1	1	1	1	2	0	3.36
<20y	25-<30	101	103	2	2	2	2	2	3	2	2	1	2	0	1		1	2.2
20+yr	20+yr	114	94	1	2	1	1	1	1	1	2	1	0	1	1	2	1	4.05
<20y	25-<30	102		2	2	2	2	2	3	2	1	1	2	0	1	2	1	3.09
=35+	=35+	107	100	2	2	2	1	1	2	0	1	1	0	0	1	2	0	3.34
<20y	25-<30	107	104	2	2	1	2	2	1	0	1	1	2	0	1	2	0	2.98
<20y	25-<30	104		2	1	1	2	3	2	0	1	1	1	1	1	2	1	2.95
20+yr	20+yr	94	117	1	2	2	2	2	1	1	2	2	2	1	1	2	0	3.795
<20y	25-<30	109	90	2	2	1	2	2	3	0	1	1	2	0	1	2	1	2.93
<20y		111	94	2	1	1	2	2	2	0	1	1		0		2	1	3.35
20+yr	25-<30	85			2	2	2	1	1	2	2	1	1	0	2	2	1	3.2
30-<35	30-<35	92		1	2	2	1	2	2	2	1	1	1	1	1	2	0	3.26
20+yr	20+yr	106		2	2	1	2	2	3	1	1	1	1	0	1	2	1	4.38
<20y	<20y	113	110	2	2	1	2	2	2	0	1	1	2	0	1	2	0	3.66
25-<30	30-<35	111		2	2	1	3	2	1	0	1	1	2	0	2	2	1	3.39
20+yr	<20y	91	90	2	2	1	2	3	1	0	1	1	2	0	1	1	0	3
20+yr	<20y	98	90	2	2	2	3	2	2	0	2	1	1	0	1	2	1	4.04
20+yr	30-<35	114	90	2	2	2	2	3	2	1	1	1	1		1			3.48
20+yr	20+yr	101	92	1	1	2	1	2	1	0	1	1	1	1	1	3	0	3.48
25-<30	25-<30	110	88	2	1	2	2	3	2	0	1	1	2	1	2	3	0	3.08
25-<30	30-<35	97	97	2	1	2	3	2	2	0	1	1	1	0	1	2	0	3.01
<20y	25-<30	98		2	1	2	2	2	2	2	2	1		1		2	1	3.2
<20y	25-<30	126	88	2	1	1	2	2	2	0	2	1	1	1	1	2	1	2.66
25-<30	30-<35	95	83	2	2	2	1	1	1	0	2	1	2	0	1	3	0	3.41

<20y	25-<30	107	101	1	1	2	2	2	1	0	1	1	2	1	2	2	1	3.16
<20y	<20y	103	96	2	1	1	2	2	2	0	1	1	1		1			3.5
<20y	25-<30	104	88	2	2	1	2	3	2	0	1	2	0	0	1	2	1	3.72
25-<30	30-<35	85	90	2	1	1	2	3	2	0	1	1	0	0	1	3	1	2.89
25-<30	25-<30	91	93	2	2	1	2	1	1	0	1	1	2	0	1	2	1	3.74
20+yr	25-<30	78	76		1	1	1	1	1	2	1	1	1	0	1	2	1	2.29
<20y	<20y	95	101	2	2	2	3	2	3	0	1	1	1	0	1	1	0	3.31
<20y	25-<30	100	88	2	1	1	3	3	2		2	1	1	1	1	2	0	3.45
20+yr	<20y	98	97	1	2	2	2	2	1	0	1	1	1	0	1	2	0	3.11
<20y	<20y	114	108	2	2	1	3	3	3	0	1	1	2	1	1	2	1	3.52
<20y	<20y	100	50		2	2	1	1	1	2	1	1	1	0		2	1	3.09
30-<35	25-<30	114		2	2	1	2	2	1	0	1	1	2	0	1	2	0	3.75
<20y	25-<30	91	97	2	1	2	2	2	2	2	1	1	2	1	1		1	3.91
20+yr	<20y	98	102	1	1	2	2	2	3	0	1	1	1	0	1		0	3.51
25-<30	30-<35	118	101	1		1	2	2	1	2	2		1	1	1	2	1	2.5
25-<30	<20y	96	81	2	2	2	2	2	1	2	2	1	1	0	1	2	1	2.27
20+yr		102	99	1	2	2	3		1	2	1	1	2	0	1	2	0	3.48
<20y	<20y	123	117	1	1	1	2	2	3	2	2	1	0	0	1	2	1	3.18
<20y	<20y	94		2	1	2	1	1	3	1	1	1	0	1	1	2	1	3.17
25-<30	30-<35	100	77	1		1	2	2	1	0	2	1	0	0	1	2	0	3.69
30-<35	=35+	110		2	1	2	3	3	2	0	1	1	1	1	1	1	0	3.38
30-<35	30-<35	94	87	1	1	2	1	2	1	0	1	1	1	1	1	3	1	3.13
<20y	<20y	111	110	2	2	2	2			2	1	1	2	1	1	2	0	3.51
<20y	25-<30	59	90	1	1	2	2	2	2	1	1	1	0		1	2	0	3.44
<20y	25-<30	82	88	2	2	1	2	2	1	0	1	1	2	1	1	2	1	3.94
<20y	<20y	108	97	2	2	1	3	2	1	2	2	1	1	1	1	1	0	3.44
=35+	=35+	117	147	2	1	1	3	2	3	0	1	1	2	1	1	2	0	4.07
25-<30	25-<30	104	100	2	1	2	2	2	1	0	1	1	0	1	2	2	0	3.25

<20y	=35+	101	103	1	2	2	2	2	1	2	1	1	2	1	1	3	0	2.82
<20y	<20y	86		2		2	1		1	2	2		0	1	2	1	1	2.61
<20y	<20y	113	95	1	1	2	2	2	3	2	1	1	1	0	1	2	1	3.3
25-<30	25-<30	115	119	2	1	2	2	2	2	0	1	1	2	0	1	3	0	3.83
30-<35	30-<35	114	98	2	2	1	2	1	3	0	1	1	2	1	1	3	1	3.54
<20y	25-<30	108	106	1	2	1	2	2	3	2	2	1	0		1			2.93
25-<30	25-<30	105	106	2	1	2	2	2	1	1	2	1	1	1	1	2	0	2.91
<20y	<20y	118		1	2	1	2	3	1	0	1	1	2	1	1			3.73
<20y		97	103	1	2	2	2		1	2	2	1	0	0	1	2	0	3.01
25-<30	=35+	105	105	2	2	2	3	1	1	0	1	1	1	1	2	3	0	2.88
<20y	<20y	114	117	2	2	1	2	2	1	1	1	1	1	0	1	3	0	3.67
30-<35	30-<35	102	103	2	1	1	2	2	2	0	1	1	2	1	1		0	3.31
<20y	25-<30	122	114	1	1	1	2	3	3	0	2	1	1	0	1	2	0	4.12
25-<30	30-<35	108	95	1	2	2	2	3	2	0	1	1	2	1	1	2	0	3.56
<20y	25-<30	100	98		2	2	2	2	1	2	1	1	0	0	2	1	1	3.45
<20y	<20y	107	101	1	1	2	3	2	2	0	1	1	2	1	1	1	0	3.46
20+yr	<20y	110	87	2	2	2	1	2	1	0	2	1	1	1	1	2	1	2.96
<20y	25-<30	110		2	2	1	2	3	2	1	1	1	2	1	1	2	1	2.97
<20y	<20y	92		1	2	1	2	2	2	0	1	1	0	1	2	2	1	3.39
<20y	<20y	111	101	2	1	2	2	2		0	2	1	2	1	1	2	1	3.82
<20y	<20y	97	79	2	2	1	2	2	2	2	1	1	2	1	1	2	1	2.96
<20y	<20y	94	84	2	1	1	2	3	1	2	2	1	1	0	1	1	1	3.46
25-<30	25-<30	106	92	2	2	1	2	1	2	0	1	1	2	0	1	2	0	3.17
<20y	<20y	92	81	1	1	2	1	2	2	2	1	1	2		2			2.86
<20y	25-<30	107	103	2	2	2	2	1	2	2	1	1	1	0	1	2	1	3.61
25-<30	25-<30	103	100	2	1	1	2	2	2	0	1	1	2	1	1	2	0	4.83
20+yr	20+yr	115		1	2	2	2	2	1	1	1	2	2	0	1	2	0	3.84
<20y	30-<35	94	87	2	1	2	2	1	1	0	1	1	0	1	1	2	0	3.01

<20y	<20y	112	110	1	1	1	2	3	3	0	1	1	2	0	1	2	1	3.71
30-<35	30-<35	117	78	2		2	1	3		0	2	1	2	1	1	2	0	4.04
20+yr	<20y	101	100	2	2	2	2	2	2	0	1	1	1	1	1	2	0	3.16
30-<35	30-<35	120	102	2	2	1	2	3	3	0	1	1	2	1	1	2	1	3.92
25-<30	25-<30	91	88	2	2	1	1	1	1	0	1	2	0	0	2	2		3.355
25-<30	30-<35	125	112	2	1	1	2	2	2	0	1	1	1	0	1	2	0	3.29
20+yr		84		1	2	2	2	2		1	1	2		0		2	1	3.02
30-<35	=35+	107	93	2	2	2	1	1	1	0	1	1	2	0	1	2	1	3.02
20+yr	<20y	94		1	1	2	3	1	2	1	1	1	1	1	1	2	1	3.56
<20y	<20y	102	88	2	2	2	3	2	1	0	2	1	2	1	1	3	1	3.75
<20y	25-<30	112	101	1	2	2	2	1	1	0	1	1	2	0	1	2	0	1.585
25-<30	30-<35	108	106	2	2	2	3	2	1	0	1	1	2	1	1	2	1	3.74
<20y	<20y	105	97		1	2	3	1	1	2	1	1	0		1			3.08
<20y	<20y	118	117	2	1	1	2	2	3	2	1	1	1	0	1	2	1	4.08
<20y	25-<30	96			2	2	2		1	0	1	1	2		1			3.9
30-<35	30-<35	105	80	2		1	1	3	1	0	1	1	0	1	2	2	0	2.95
<20y	25-<30	100	80	1	1	1	2	1	1	0	1	1	0	1	1	2	1	4.25
20+yr	<20y	71	90	2	1	2	2	2		2	1	1		1			1	2.178
<20y		85	89	1	2	2	2		1	2	2	1	0		1			2.3
25-<30	25-<30	96	88	2	1	2	1	2	2	2	1	1	0	1	1	2	0	2.61
25-<30	25-<30	98	98	2	2	2	2	2	2	0	1	1	0	1	1	2	0	4.28
25-<30	25-<30	99	94	2	1	1	2	3	3	0	1	1	2	0	1	2	1	3.75
25-<30	30-<35	91	85	2	1	2	2	1	1	2	2	1	1	1	1	2	0	3.36
25-<30	30-<35	115	100	2	2	2	2	2	2	0	1	1	2	0	1	2	0	3.48
30-<35	30-<35	72	40	2	1	2	1	1	2	0	1	1	2		1			3.59
20+yr	25-<30	118	109	2	2	2	3	1	2	0	1	1	1	1	1	2	1	3.27
<20y	<20y	92	87	2	2	2	1	2	1	2	2	1	0	0	1	2		2.99
<20y	30-<35	90	85	1	2	2	1	1	3	0	1	1	1	0	1	2	0	2.94

<20y	25-<30	113			1	1	3	3	2	1	1	1	1	1	1	2	0	3.05
30-<35	25-<30	118		2	1	1	2	2	2	0	1	1	2	0	1		0	3.79
=35+	=35+	104	102	2	2	1	2	1	2	0	1	1	2	1	2		0	3.19
20+yr	<20y	100		2	1	2	2	2	2	0	2	1	0	0	1	2	1	2.78
25-<30	25-<30	101		2	1	2	2	3	3	0	1	1	2	1	1	2	0	3
=35+	=35+	103	95	2	1	2	1	1	2	0	1	1	2	1	1	3	1	3.01
<20y	25-<30	105		2	2	1	3	1	1	0	1	1	1	0	1	2	1	2.8
25-<30	30-<35	113	87	2	1	1	2	2	1	0	1	1	2	1	1	2	0	3.6
25-<30	25-<30	122	125	2	1	2	3	2	2	1	1	1	2	1	1	2	1	3.7
<20y	30-<35	104	90	2	2	2	1	1	1	0	1	1	1	0	1	2	1	3.92
25-<30	25-<30	115	108		1	2	2	2	3	0	2	1	2	1	1	3	1	2.98
25-<30	<20y	106	91	2	1	2	2	3	2	0	1	1	1		1			3.44
30-<35	=35+	96		2	1	2	2	3		0	2	1	0	0	2			3.64
30-<35	=35+	120	128	2	1	1	2	3	3	0	1	1	2	0	1	2	0	2.92
25-<30	=35+	111	106	2	2	1	3	3	3	0	1	1	2	1	1	2	1	3.35
<20y	25-<30	101	95	2	2	1	2	2	1	0	1	1	1	0	1	2	0	3.72
25-<30	25-<30	100	97	2	1	2	2	2	3	2	2	1	1	0	1	2		3.88
25-<30	25-<30	100	78	2	2	2	1	2	2	0	1	1	0	1	2	2	1	2.435
25-<30	30-<35	94	114	1	2	2	3	3	2	0	1	1	2	1	1	2	0	3.67
<20y	<20y	103	95	1	2	2	2	1	3	0	1	1	1	1	1	3	1	3.81
30-<35	30-<35	102	105	2	2	1	2	2	2	0	1	1	0	0	1		0	4.93
20+yr		86		2		2	1		1	0	1	1	0	1	1	2	1	2.95
<20y	<20y	100	84		2	1	1	2	1	0	1	1	0	0	1	2	1	3.62
<20y	<20y	103	101	2	1	1	2	3	2	0	1	1	2	0	1	2	0	3.49
25-<30	25-<30	117	108	2	1	2	2	2	3	0	1	1	1	1	1	2	1	3.94
25-<30	25-<30	102	90	2	2	2	1	2	1	2	1	1	1	1	1		0	3.28
30-<35	=35+	111	99	1	2	1	2	2	1	0	1	1	2	1	1	2	1	3.187
25-<30	25-<30	98	96	2	2	2	2	3	1	0	2	1	1	0	1	2	0	3.69

<20y	25-<30	104	103	1	2	1	3	3	3	1	1	1		1		3	0	3.36
30-<35	30-<35	102	93		2	1	3	3	3	0	1	1	1	0	1	2	0	3.5
25-<30	25-<30	100	87	2	1	1	2	3	1	2	1	1	1	1	1	2	1	3.22
25-<30	25-<30	122	101	2	2	1	3	3	2	0	1	1	2	0	1	2	0	3.53
30-<35	30-<35	100		1	1	2	2	3	3	0	1	1	2	1	2	3	1	3.63
25-<30	=35+	94		2	2	2	2	3	3	0	1	1	2	1	1	2	1	3.06
30-<35	=35+	84	81	2		2	1	1	1	2	1	1	1	1	1		1	3.58
30-<35	30-<35	95		2	2	2	2	2	2	0	1	1	2	1	2	2	1	3.09
30-<35	30-<35	107		2	2	2	2	2	2	0	1	2	2	0	2	2	1	3.69
<20y	25-<30	122	110	1	1	1	2	2	2	0	1	1	2	0	1	2	0	3.41
25-<30	30-<35	108	76	2	1	2	2	2	2	1	1	1	2	1	1	2	1	2.535
=35+	=35+	97		2	2	2			3	0	1	1	0	0	1	3	1	3.23
<20y	25-<30	90	101	1	1	2	2	3	2	0	1	1	0	1	1	2	1	2.74
=35+	=35+	108	112	2	2	1	3	1	2	0	1	1	2	0	1	2	1	3.12
25-<30	30-<35	96	95	2	2	2	2	2	1	0	1	1	2		1	3	1	2.96
<20y	25-<30	102	86	2	2	2	2	3	3	1	1	1	1	0	1	3	1	2.89
25-<30	25-<30	92	88		1	2	2	2	2	0	2	1	0	0	1	2	1	3.15
20+yr	<20y	110	99	1	2	2	3	2	1	1	2	1	2		1			3.675
<20y	30-<35	81	100	1	1	1	2	3	2	2	2	1	2		2			2.84
20+yr	20+yr	89	83	2	2	1	1	1	1	1	1	1	1	1	1	2		4.06
25-<30	25-<30	83		2	2	2	1		1	0	1	1	0	0	2	3	1	2.62
30-<35	30-<35	97	102	2	1	1	2	2	3	0	1	1	2	1	1	3	0	3.79
20+yr	20+yr	86		1	2	1	2	2	2	1	2	1	1	1	1	2	1	3.4
<20y	25-<30	84	79		1	2	1	2	2	1	1	1						3
<20y	25-<30	89	86	2	1	1	2	2	1	1	1	1	1	1	1	2	1	3.82
20+yr	<20y	94		1	2	2	2	2	1	0		1	2	1	1	2	1	3.08
20+yr	<20y	85	105	2	2	2	2	3	2	0	1	2	2	1	1	2	1	3.02
<20y	<20y	114	100	2	1	1	1	2	1	0	1	1	1	1	1	2	1	3.45

25-<30	25-<30	115	94	2	2	2	2	2	3	2	1	1	2	0	2	3	0	3.37
25-<30	25-<30	89	94	2	1	2			1	0	1	1	1	0	1	2	1	3.94
<20y	25-<30	94	88	2	1	1	2	2	3	0	1	1	2	1	1	3	1	3.45
<20y		103	88	2	1	2				0	1	1	2	0	1	3	0	3.13
25-<30	30-<35	111	117	2	1	1	2	2	2	0	1	1	2	0	1	2	1	3.7
<20y		98	88	1	2	2	3		3	0	1	1	0	0	1	2	0	3.38
<20y	25-<30	120		1	2	1	1	3	2	1	1	1	2	1	1	2	0	3.49
=35+	=35+	100	109	2	2	1	3	3	2	0	1	1	0	0	1	2	1	3.98
<20y	<20y	103	94	2	1	2	2	1	1	2	2	1	1	1	1	2	1	2.54
30-<35	=35+	110	117	1	2	2	3	1	2	0	2	1	2	1	1	2	1	3.88
<20y	25-<30	102	95	2	1	1	2	3	1	0	1	1	1	0	1	2	1	3.54
25-<30	25-<30	94	98	2	1	2	2	3	2	0	1	1	1	1	2	2	1	3.27
<20y	<20y	102		1	1	1	2	2	1	2	2	1	1	1	1	1	1	3.39
<20y	<20y	78	100	1	2	1	2	2	1	0	1	1		0		2	0	4.06
<20y		100		1	2	1	2		2	0	1	1	1	1	1	2	1	3.39
=35+	=35+	114		2	2	1	1	1	2	0	1	1	2	0	1	3	0	3.85
<20y	<20y	88	81	2	2	2	2	2	1	0	1	1	1	0	2	1	1	3.48
25-<30	30-<35	104	104	2	1	1	3	3	2	2	1	1	1	1	1	3	0	3.73
<20y	=35+	103	112	2	2	2	2	2	2	0	2	1	1	0	1	2		4.15
30-<35	30-<35	104	107	2	2	1	2	2	1	2	1	1	1	0	1		0	3.25
<20y	25-<30	114	96	2	2	2	2	2	2	0	1	1	1	1	1	2	0	3.25
25-<30	25-<30	122	117	2	1	1	2	2	3	0	1	1	2	1	1	3	0	3.71
25-<30	30-<35	95	91	2	1	1	1		2	0	1	1	2	1	1	2	0	3.47
<20y	<20y	104	98	2	2	1	2	3	2	0	1	1	2	0	1	2	1	2.8
<20y	25-<30	105	92	2	2	2	2	3	3	0	2	1	2	1	1	2	0	4.43
<20y	25-<30	115	97	2	1	2	3	3	3	0	1	1	2	1	1		0	4.08
30-<35	30-<35	104	105	2	1	1	2	2	2	0	1	1	1	0	2	2	0	3.54
=35+	=35+	105	89	1	2	1	3	3	2	0	1	1	2	1	1	3	0	4.82

20+yr	20+yr	105	108	1	2	2	2	2	2	0	1	2	2	1	1	2	1	3.5
<20y	25-<30	102		2	2	2	2	3	2	0	1	1	1	1	1	2	0	3.51
<20y	25-<30	97	98	1	1	1	2	1	1	2	1	1	0	1	2	2	0	2.87
20+yr	20+yr	122	91	1	1	1	1	2	3	1	1	1	1	1	1	2	1	3.24
<20y	<20y	84	84	1	2	1	1	1	2	2	2	1	0		1			2.98
25-<30	30-<35	97	91	1	1	2	2	1	3	2	2	2	1	1	1	2	1	2.81
25-<30	25-<30	84	79	2	1	1	1	2	2	1	2	1	0	1	1	2	1	3.87
20+yr	<20y	111	99	2	2	2	2		1	1	1	1	1		1			3.24
<20y	25-<30	108		1	1	1	2	2	2	1	1	1	1	1	1	2	0	3.35
<20y	<20y	85	80	2	2	2	1	1	1	0	1	1	0	1	1	2	0	3.4
25-<30	25-<30	110	100	2	2	2	3	3	2	0	1	1	0	0	1	2	0	3.72
30-<35	30-<35	99		2	2	1	2	2	1	0	1	1		1	1	3	1	3.24
25-<30	25-<30	117	94	2	1	1	2	2	1	2	1	1	0	1	2	2	0	2.34
25-<30	25-<30	94	100	1	2	2	2	2	1	2		2	2	1	1		1	2.89
<20y	25-<30	88	93	2	2	1	2	2	1	0	1	1	0	1	2	2	0	4.32
25-<30	30-<35	107	95	2	2	1	2	2	3	0	1	1	1	1	1	2	0	3.64
<20y	25-<30	114	98	2	1	2	2	2	1	2	1	1	2	1	1	2	0	3.5
<20y	<20y	99	91	1	2	2	3	3	3	2	2	1	2	1	1	2	0	3.32
<20y	30-<35	94	82	2	2	2	1	2	1	2	2	1	0	0	1		0	3.5
30-<35	30-<35	104	117	2	1	1	3	3	2	0	1	1	2	1	1	2	0	3.58
25-<30	30-<35	113	110	2	2	2	3	3	1	1	1	1	2	1	1	2	1	3.68
20+yr	20+yr	106	101	1	2	2	2	2	2	0	1	1	2	0	1	3	0	2.96
<20y	25-<30	106	95	2	2	2	3	3	2	0	2	1	0	1	1	2	0	3.64
25-<30	30-<35	94	80	2		1	2	3	3	0	1	1	2	1	1	3	1	3.65
<20y		111	98	1	2	1	2			0	1	1	2	1	1	2	0	2.75
<20y	25-<30	115	104	2	1	1	3	3	2	0	1	1	2	0	1	2	1	2.46
25-<30	25-<30	108	97	1	2	1	3	2	1	2	1	1	2	1	1	2	1	3.46
<20y		103	101	1	2	1	2	1	3	0	1	1	2	1	1	2	1	2.93

20+yr	<20y	108	98	2	2	2	3	2		0	1	1	2	0	1	1	1	3.18
<20y	25-<30	100	89	2	2	2	3	3	1	0	1	1	1	1	1	2	1	3.96
25-<30	25-<30	96	96	2	2	1	2	2	2	0	2	1	2	1	1	2	0	3.12
<20y	<20y	80	81	1	1	2	2	2	1	0	1	1	2		1			2.92
20+yr	<20y	85	94	2	2	1	1	3	2	2	2	1	0	0	1		1	2.81
<20y	<20y	108		1	2	2	2	2	1	1	1	1	1	1	1	2	1	2.89
20+yr	<20y	107		1	2	2	1	2	1	1	1	1	1	1	1	2	1	2.63
30-<35	30-<35	101	94	2	1	2	2	2	1	0	1	1	2	0	1	2	0	3.38
<20y	25-<30	115	106	2	1	2	2	2	1	2	1	1	0	0	1	2	0	2.89
30-<35	30-<35	97	112	1	2	2	2	3	1	1	1	1	2	1	1	3	1	3.38
30-<35	=35+	115	93	2	1	1	1	2	3	0	1	1	1	0	2	2	0	3.99
20+yr	30-<35	110	101	1	2	1	2	2	2	0	1	1	2	1	2	2	0	3.78
<20y	25-<30	97	100	2	2	2	2	2	2	0	1	1	2	1	1	1	1	3.38
20+yr	<20y	103	95	1	2	2	2	2		1	1	1	2	1	1		1	2.72
<20y	<20y	100	95	1	2	2	2	2	3	1	1	1	2	1	1	1	1	2.61
25-<30	25-<30	108	104	2	1	1	2	3	3	0	1	1	2	1	1	2	0	3.7
25-<30	25-<30	106	104	2	1	2	2	3	3	2	1	1	1	1	1	2	0	3.53
<20y	30-<35	102	95	2	2	1	2	3	1	2	1	1		1		1	1	4.17
20+yr		113	105	1	2	2	2		3	0	2	1	2	0	1	2	0	3.735
20+yr		98	111	1	1	2	2		1	2	2	1	2	0	2	2	1	2.29
30-<35	30-<35	114	112	2	1	1	2	2	3	0	2	1	1	1	1	2	0	2.84
25-<30	=35+	104	104	2	1	1	3	3	3	1	2	1	1	1	1	2	0	3.3
<20y	<20y	111		2	1	2	2	2	1	2	1	1						2.62
<20y	20+yr	99		2	2	1	2	2	3	0	1	1	1		2			3.71
25-<30	25-<30	102	98	2	2	1	2	2	3	2	1	1	1	1	1		0	4.49
<20y	<20y	101	87	1	2	1	2	2		2	2	1	2	1	1	2	1	4.04
30-<35	=35+	85	90	1	1	1	1	2	1	2	2	1	1	1	2		0	3.43
<20y	25-<30	102	88	2	1	1	2	2	1	0	2	1	2	0	1	2	1	3.39

<20y	<20y	108	101	1	1	1	2	1	3	2	1	1	1	1	1	2	1	4.03
25-<30	25-<30	106	95	1	1	1	2	2	2	0	1	1	2		1			4.16
30-<35	25-<30	108	128	2	2	1	2	2	1	1	2	1	2	1	1	2	1	3.08
<20y	<20y	123	112	2	1	2	3	2	3	0	1	1	1	1	1	2	1	3.16
25-<30	25-<30	104		1	1	2	2	2	1	0	1	1	0	0	1	3	0	3.9
25-<30	30-<35	102	104	2	2	2	2	3	2	0	1	1	0		1	1	0	3.625
30-<35	30-<35	115	86	1	2	2	2	3	2	0	1	1	2	0	1		1	3.12
25-<30	<20y	109	95	1	2	1	2	3	2	0	1	1	1		1	2	0	3.67
30-<35	=35+	115	91	2	2	1	1	1	1	2	1	1	1	1	1		1	3.76
25-<30	30-<35	105	94	2	1	1	2	2	2	2	1	1	2	1	1	2	1	4.12
25-<30	30-<35	120	117	2	1	1	2	2	2	1	1	1	2	1	1	2	0	3.94
20+yr		99	86	2	2	1	1	2	1	0	1	1	1	0	2	2	1	3.36
25-<30	=35+	102	105	2	1	1	1	1	1	0	1	1	0	1	1	2	1	3.07
25-<30	25-<30	117	110	1	1	1	2	2	1	0	2	1	1	1	1	2	0	3.9
<20y	<20y	105	88	2	1	2	2	2	2	0	2	1	1	1	1	2	0	3.07
25-<30	25-<30	108	89	2	2	2	2	2	2	1	2	1	0	1	1	2	0	2.52
30-<35	30-<35	96	89	2	2	1	2	1	3	0	2	1	1	0	1	2	1	3.76
<20y	<20y	99	104	2	1	1	2	2	2	1	1	1	1	0	2	2	1	4.04
30-<35	<20y	105	91	2	2	2	2	2	1	2	2	2	1	1	1	2	1	3.51
<20y	<20y	95	88	2	2	1	2	2	1	0	1	1	2	0	1		0	3.55
30-<35	=35+	99	99	2	2	2	2	1	1	0	1	1	1	0	2	2	0	3.41
25-<30	30-<35	108	100	2	1	1	2	3	2			1	2	1	1	2	0	3.64
<20y	<20y	120	94	2	1	1	2	2	2	0	1	1	1	1	1		0	3.4
<20y	<20y	107	95	2	2	1	2	2	1	0	1	1	2	1	1	2	0	3.09
25-<30	25-<30	99		1	1	1	3	3	2	0	1	1	0	1	1	2	0	3.71
25-<30	25-<30	89	87	2	1	2	1	2		0	2	1	0	0	1		0	3.55
25-<30	25-<30	99	108	1	1	1	3	3	3	0	1	1	2	1	1	2	0	4.64
20+yr	20+yr	104	100		2	2	3	2	1	0	1	1	1		1			3.65

20+yr	<20y	81	86	2	2	1	1	2	1	0	1	1	1	0	2	2	1	3.82
30-<35	30-<35	103		1	2	1	1	1	3	0	1	1	1	0	1	3	1	3.06
20+yr	20+yr	111		1	2	1	2	2	1	1	2	1	1		1			3.74
25-<30	25-<30	97	110	2	1	2	2	2	1	2	1	1	1	1	1	2	0	2.77
<20y	25-<30	99		2	1	2	2	2	3	0	1	1	2	1	1	2	1	3.08
<20y	25-<30	110	101	2	1	1	3	3	3	0	1	1	2	1	1	3	0	3.06
<20y	25-<30	77	83	1	1	2	2	2	1	0	1	1	0	1	1	2	1	2.34
20+yr	20+yr	115	101	2	2	1	2	2	3	1	1	1						4.02
<20y	25-<30	97	84	1	1	1	2	2	1	2	1		1	1	1		0	2.85
20+yr		117				1	1		1	0	1	1	0		1			3
25-<30	30-<35	107	66	2	2	1	2	3	2	1	1	1	1	0	1	2	1	4.31
<20y	25-<30	86	91	2	2	1	2	2	2	2	1		1	1	1	2	0	3.22
<20y	25-<30	101	94	1	1	1	3	3	2	0	2	1	1	1	1	2	1	3.71
30-<35	30-<35	103		1	1	1	1	1	2	2	1	1		0		3	1	2.79
20+yr	25-<30	102	87	2	2	2	2	2		1	2	1	1	0	2	2	1	3.88
<20y	<20y	100	81	2	2	2	2	2	3	1	1	1	1	0	1	1		3.26
25-<30	25-<30	92	88	2	2	2	1	2	2	0	1	1	0	0	1	2	0	2.76
<20y	25-<30	99	100	2	1	1	2	3	2	0	2	1	2	0	1	1	0	3.39
=35+	=35+	109	112	1	1	1	3	3	3	0	1	1	2	1		3	1	4.32
<20y	<20y	78		1	1	2	2	1	1	2	1	1	1	1	1	1	1	3.64
<20y	25-<30	90	85	1	2	1	2	2	1	2	1	1	0	0	2	2	1	4.42
25-<30	25-<30	100	101	2	1	1	3	2	2	1	1	1	2	0	1	2	0	3.1
<20y	<20y	118		1	2	2	2	3		0	1	1	1	1	1	2	1	2.48
25-<30	25-<30	112	110	2	1	2	2	2	2	0	2	1	2	1	2	2	1	3.35
<20y	25-<30	110	110	2	2	1	2	2	3	0	1	1	1	1	1	2	1	3.41
25-<30	25-<30	122	106	2	1	2	1	3	3	0	1	1	2	0	1	3	0	1.96
<20y	25-<30	104	93	1	1	2	2	2	2	0	1	1	1		1	2	1	3.295
<20y	<20y	105	88	2	2	2	2	3	1	1	1	1	1	1	1		0	3.68

20+yr	20+yr	102	88	1	1	1	2	2	2	1	1	2	1	0	1	2	1	3.4
20+yr	<20y	100	94	2	1	1	2	1	1	1	1	1		1		2	1	3.16
20+yr	<20y	107			2	2	2	2	1	0	1	1	2		1			3.43
20+yr	<20y	108	109	1	2	1	2	2	1	0	1	2	1		1			1.48
<20y	<20y	118	101	2	2	2	2	3	3	0	1	1	2	0	1	2	0	3.36
<20y	25-<30	89	91	1	2	2	2	1	1	2	1	1	0		1			3.04
20+yr	<20y	109	107	1	2	1	2	2	1	1	1	1	1	1	1			3.09
30-<35		100		1	1	1	1	1	1	0	1	2	1		1			3.5
20+yr	20+yr	123			2	1	2	2	1	0	2	1	1	0	1	2	0	2.89
<20y	25-<30	105	103	2	2	2	2	2	3	2	1	1	1	0	1	3	0	3.11
<20y	<20y	112	96	1	2	1	3	2	1	0	1	1	1	1	1	2	0	2.23
<20y	<20y	113	88	2	1	1	3	3	1	0	1	1	2	0	1	2	1	3.83
<20y	25-<30	89	82	1	1	1	2	2	2	0	1		2	1	1		0	3.42
<20y	30-<35	118		2	2	1	2	2	1	0	2	1	0	1	1			4.01
30-<35	25-<30	103		2	1	2	2	2	1	0	1	1	2	1	2	1	0	3.51
30-<35	=35+	120	89	2	2	1	2	2	2	2	1	1	2	1	1	2	1	3.63
25-<30	25-<30	89	106	2	1	2	1	1	2	1	1	1	1	0	1	1	1	2.72
30-<35	30-<35	123	103	2	2	1	2	2	2	0	1	1	2	0	1	2	1	3.95
<20y	25-<30	102		2	1	1	2	2	2	1	1	1	0	1	1	1	1	3.3
25-<30	25-<30	81		2	2	2	2	2	1	2	2	1		1		2	0	3.91
25-<30	25-<30	112	114	2	2	2	2	1	3	0	1	1	2	0	1	2	1	3.57
30-<35	30-<35	108	100	2	2	2	1	3		0	1	1	2	1	1			3.19
25-<30	<20y	100	89	1	1	2	2	2	2	0	1	1	1	1	2		0	3.51
20+yr	25-<30	109	92	2	2	1	2	1	1	0	1	1	2		1			3
<20y	<20y	110		2	2	1	1	2	1	1	1	1	1	1	1	2	0	3.11
<20y	<20y	89		1	1	2	2	1	1	0	1	1	1	1	1	2	1	3.38
<20y	=35+	94	101	1	1	2	2	2	2	0	1	1	0	0	1	3	0	3.43
25-<30	30-<35	118	77	2	1	2	3	2	2	0	1	1	2	0	1	2	1	2.68

25-<30	25-<30	83	93	2		1	3	2	2	0	1		2	1	1	2	1	3.92
<20y	25-<30	104		2	2	2	2	2	2	0	1	1	1	1	1	2	0	4.33
30-<35	=35+	108		2	2	1	2	1	1	0	1	1	2		1			3.93
25-<30	30-<35	118		2	1	1	3	3	2	0	2	1	2	0	1		0	3.935
30-<35	30-<35	97	81	2	2	1	2	2	1	0	1	1	0	1	1	2	1	2.93
25-<30	25-<30	101	88	1	1	2	3	2	2	0	1	1	2		1			3.45
<20y	25-<30	105		2	2	2	2	2	3	0	1	1	1	1	1	3	1	3.32
<20y	25-<30	114	98	2	2	2	2	2	3	0	2	1	1	1	1	1	0	3.86
<20y	25-<30	105	117	2	2	1	2	2	2	0	2	1	1	0	1	2	1	3.42
30-<35	25-<30	107	102	2	1	1	2	2	3	0	2	1	2	0	1		0	2.41
<20y	<20y	127	112	2	2	1	3	3	3	0	1	1	2	1	2	3	1	3.03
25-<30	25-<30	95	91	2	2	2	2	2	1	0	1	1	2	1	2	2	1	3.68
<20y	=35+	92		1	2	2	2	1	1	2	1	1	0	0	1	2	1	4.68
<20y		109	100	2	2	1	2	2	2	0	1	1	2	1	1	2	0	3.29
<20y	<20y	96		1	1	1	2	2	1	2	1	1	0		2			3.08
25-<30	<20y	97	76	2	1	2	2	2		0	1	1	0	0	1	3	0	3.85
30-<35	30-<35	97	93	1	2	1	3	3	1	0	1	1	1	1	1	3	1	2.38
<20y	25-<30	108		1	1	2	3	2	1	0	1	1	1	0	1	2	0	4.56
25-<30	30-<35	99	103	1	1	2	2	1	2	2	2	1	2	1	1	2	1	3.7
<20y	25-<30	100	100	2	1	1	3	3	2	0	1	1	2	0	1	2	1	3.21
<20y	<20y	89	101	2	1	2	1	1	1	2	1	1	0	1	1	2	0	3.6
25-<30	=35+	101	105	2		2	3	3	3	0	1	1	2	0	1	2	1	3.89
25-<30	25-<30	97	94	2	2	2	2	1	2	0	2	1	1		1			3.25
25-<30	25-<30	90	106	2	1	1	2	2	1	0	1	1	2	1	1	3	0	3.14
<20y	25-<30	99	81	2	1	2	1	2	1	0	2	1	1	0	1	3	1	3.19
<20y	25-<30	108	97	1	1	2	2	3	3	2	2	1	0	1	1	3	0	3.48
<20y	30-<35	91	106	1	2	2	1	2	3	0	2	1	2	1	2	2	1	3.2
<20y	<20y	98	94	2	2	1	1	1	2	2	1	1	0	0	1	2	0	2.66

30-<35	25-<30	117	95	1	2	2	2	2	2	2	2	1	1	1	1	2	1	2.79
<20y	25-<30	123		2	1	2	2	3	3	0	1	1	2	1	1			3.85
<20y	<20y	103	97	2	2	2	3	2	1	0	1	1	2	1	1	3	1	3.22
25-<30	<20y	104	94		1	1	2	1	3	0	2	1	1	0	1	3	0	2.73
<20y	25-<30	98	100	2	1	1	2	1	1	0	1	1	1	1	1	2	0	3.1
25-<30	=35+	111	103	2	1	2	2	1	2	0	1	1	1	1	1	2	1	3.11
20+yr	<20y	92	99	1	1	2	2	1	1	2	1	1	0	1	1	2	1	2.76
30-<35	=35+	93	93	2	2	2	2	2	2	0	1	1	1	0	1	3	0	3.49
25-<30	=35+	100	112	1	1	2	2	2	1	0	2	1	2	1	1		0	3.05
<20y	25-<30	105	103	2	1	2	2	2	3	0	1	1	1	1	1	3	1	2.91
25-<30	25-<30	101	112	2	1	1	3	3	2	1	1	1	1	0	1	3	0	3.45
=35+	=35+	111	128	2	1	2	3	3	3	0	1	1	2	0	1	2	1	3.59
20+yr	<20y	103		1	2	1	1	3	1	0	1	1	1	1	1	2	1	4.64
20+yr	<20y	97	90	2	1	1	2	2	2	0	1	1	1	0	1	2	1	3.51
25-<30	30-<35	126	98	2	1	1	2	2	3	0	1	1	2	1	2	2	1	3.1
20+yr	20+yr	89	105	1	2	1	3	2	2	0	1	1	1	0	1	2	0	3.55
25-<30	25-<30	93	94	2	1	1	2	2	2	0	1	1	2	0	1	2	1	2.28
<20y	<20y	98	92	1	1	1	1	2	1	1	1	1	1		1			3.97
30-<35	=35+	75	93	1	2	2	3	3	3	0	1	1	1	0	1	2	0	2.76
<20y	30-<35	105	101	2	1	1	2	2	3	0	1	1	0	0	1	2	0	3.09
=35+	=35+	106	98	1	2	1	2	3	3	0	1	1	2	0	1	1		3.34
<20y	<20y	127	88	1	1	1	2	2	3	0	1	1	2	0	1	2	0	3.99
30-<35	30-<35	109	100	2	2	2	2	2	2	0	1	1	2	0	1	3	1	3.51
<20y	<20y	102	97	2	2	1	1	1	1	2	1	1	1	1	1	2	1	3.66
<20y	25-<30	114	89	1	1	2	2	2	1	0	1	1	1		1			3.45
20+yr	<20y	102		2	1	2	3	2	1	1	1	1	1	0	1	2	1	3.19
20+yr	<20y	96	97	1	1	2	2	2	2	0	1	1	1	0	1	2	0	3.4
<20y		103	96	1	2	2	1		1	2	1	1	1	0	1	2	1	3.48

<20y	<20y	104		2	1	2	1	1	1	0	1	1	2	0	1	2	0	3.42
20+yr	<20y	91		1	1	1	2	1	1	1	2	1	2	1	1	1	0	3.29
30-<35	30-<35	97	97	2	2	2	2	1	2	0	1	1	2	1	1	3	1	3.57
<20y	30-<35	108		1	1	2	2	1	2	2	1	1	0	1	1	2	1	2.41
<20y	<20y	105	98	2	1	2	2	2	1	0	1	1	2	0	1	3	1	2.99
20+yr	<20y	97	97	2	1	2	2	2	1	0	1	1	1	0	1	2	1	3.26
<20y	25-<30	102	88	2	2	2	2	2	1	0	1	1	0	0	1	3	0	2.1
30-<35	30-<35	103	94	1	1	2	2	2	1	0	2	1	0	1	1	2	0	3.26
20+yr		84	85	1	2	2	2	1	1	0	1	1	0	0	2	2	1	3.44
<20y	25-<30	84	85	1	1	1	2	2	1	0	2	1	1	0	1	1		2.85
=35+	=35+	95	93	2		1	2	2	1	2	1	1	0		1			3.67
20+yr	20+yr	87		1	2	2	2	1	1	2	2	1		1		3	1	2.74
30-<35	30-<35	110	83	2	2	1	2	1	2	0	1	1	0	0	1		0	3.9
25-<30	30-<35	114	119	2	1	1	2	3	3	0	1	1	2	0	1	3	0	3.37
<20y	25-<30	115	88	2	2	1	1	2	3	0	1	1	2	1	1	2	0	3.25
<20y	<20y	109	90	1	1	1	2	2	2	0	2	1	2	0	1	2	0	4.48
<20y	25-<30	102	95	2	2	2	2	3	3	0	2	1	2	0	1		0	3.84
<20y	20+yr	107	122	1	2	1	2	3	2	0	1	1	2	0	1	2	0	3.915
<20y	25-<30	117			2	1	2	1	3	0	1	1	2	0	1	2	1	3.81
<20y	<20y	92	97	2	2	1	2	2	3	0	2	1	1	1	1	2	0	3.98
<20y	25-<30	100	81	2	1	2	1	1	1	2	1	1	0	0	1	3	1	3.6
<20y	<20y	106	108	2	2	2	2	2	3	0	1	1	1	0	1	3	0	3.02
25-<30	25-<30	107	106		1	1	2	2	2	2	2	1	1	1	2	2	0	4.46
25-<30	30-<35	115	80	2	2	1	2	3	3	0	1	1	1	0	1	2	1	4.74
<20y	<20y	115		1	2	1	2	2	3	1	1	1	0	0	1	2	1	3.7
30-<35	=35+	109	94	2	1	2	2	2	2	0	1	1	2	1	1		0	4.12
<20y	<20y	107	96	1	1	1	2	2	1	0	2	1	2		1			4.07
25-<30	25-<30	97	101	2	1	1	2	3	2	0	1	1	2	0	2	2	0	3.71

25-<30	30-<35	107	104	2	1	2	2	1	3	0	1	1	2	0	1	2	0	3.58
30-<35	=35+	105	95	1	2	2	1	3	3	0	2	1		1	1	2		3.78
30-<35	=35+	97	109	2	1	1	2	1	2	0	1	1	1	0	1	2	1	4.33
30-<35	30-<35	103	98	2	1	2	1	1	1	1	1	1	2	1	1	2	0	2.97
<20y	<20y	101		1	1	2	2	2	1	2	2	1	0	0	2	2	1	2.91
20+yr	<20y	100		1	2	2	1	2	2	1	1	2	1	1	1	1		3.61
20+yr	20+yr	100		1		1	2	2	2	0	1	1	0	1	1	2	1	3.37
30-<35	=35+	105	105	2	1	1	2	2	1	0	2	1	2	0	1	2	1	3.69
<20y	25-<30	108	97	2	1	1	2	2	2	0	1	1	0	0	2	2	1	3.61
<20y	<20y	89	96		1	1	2	2	2	0	1	1	1	1	1	2	1	3.55
20+yr		101		1	2	1	2	2	1	0	1	1	0	1	1	2	0	3.28
20+yr	<20y	110	114	1	2	1	1	2	3	1	1	1		0	2	2	0	4.1
<20y	25-<30	118	101	2	2	2	2	2	2	0	2	1	1	1	1	2	0	3.53
<20y	25-<30	114	129	2	1	2	3	2	2	0	1	1	2	0	1	2	0	2.82
<20y	25-<30	111	88	2	2	1	2	3	3	0	2	1	2	0	1	2		3.11
25-<30	25-<30	92	119	2	1	2	2	2	2	0	2	1	2	0	1	1	0	2.47
<20y	<20y	88			1	2	2	3	3	0	1	1	1	0	1	2	1	2.99
30-<35	25-<30	98	97	2	1	1	2	3	2	2	2	1	1	0	1			4.34
<20y	25-<30	101	108	2	1	2	3	1	2	0	1	1	2	1	1	2	0	3.34
<20y	25-<30	107	94	2	1	2	3	3	2	0	1	1	2	0	1	3	1	3.19
=35+	30-<35	122	117	2	2	2	3	3	3	0	1	1	2	0	2	3	0	3.22
20+yr	<20y	112		2	2	1	2	3	3	0	1	1	1	0	1	2	1	4.19
20+yr	<20y	112		2	2	1	2	3		0	1	1	2	0	1	2	0	3.36
30-<35	30-<35	113	93	2	1	2	1	2	2	0	1	1	2	0	1	1		3.505
20+yr	<20y	112		1	1	1	2	1	1	1	1	1		1		2	1	3.16
<20y	30-<35	113	96	2	1	1	2	2	2	0	1	1	1	1	2	2	1	3.99
25-<30	30-<35	114	98	2	1	2	3	3	3	0	2	1	2	0	1	2	0	3.34
20+yr	<20y	93	98	2	2	1	1	2	2	1	1	1	2	1	1	3	0	3.4

<20y	25-<30	100		2	2	2	1	2	1	2	2	1		0		2	1	2.89
<20y	<20y	110	98	2	2	1	2	1	2	2	1	1	2	1	1	2	1	3.27
<20y	<20y	102	106	2	1	1	2	2	3	0	1	1	2	0	1	2	1	3.24
20+yr	<20y	83	89	2	2	1	2	2	1	1	1	1	0	0	1	1	1	2.56
<20y	<20y	96	97	2	2	2	2	2	1	0	1	1	1	0	1	2	0	4.075
25-<30	25-<30	97	89			1	3	2	2	0	2	1	1		2			3.25
<20y	25-<30	114	92	2	1	1	2	3	1	1	1	1	2		1			3.46
25-<30	<20y	91	90	2	1	2	2	2	2	0	1	1		0		2	1	3.57
30-<35	30-<35	117	97	2		2	2			0	1	1	2	0	1		0	3.19
20+yr	<20y	105	94	2	2	2	2	2	1	0	1	1	1		1			2.99
25-<30	30-<35	102		1	1	1	1	3	2	0	1	1	2	0	1	3	1	3.68
<20y	30-<35	104	91	2	1	1	1	2	3	0	1	1	1	0	1	2	1	3.5
<20y	25-<30	113	101	2	1	2	1	2	1	0	1	1	1	1	1	2	0	3.45
25-<30	30-<35	101		1		1	1	1	1	0	2	1	2	0	1	3	0	3.79
=35+	=35+	89	92	2	2	1	2	3	2	0	1	1	2	1	2	2	0	3.27
=35+	25-<30	101	95	2	1	2		2	2	1	1	1	2	0	1	2	0	3.08
25-<30	=35+	107	82		2	1	3	3	1	0	1	1	2		2	1	1	3.23
30-<35	30-<35	115	109	2	1	1	2	2	3	2	1	1	1	0	1	2	0	3.76
30-<35	=35+	91	88	1	2	2	2	2	2	0	2	1		1			0	3.26
<20y	=35+	98		2	1	2	2	1	2	2	2	1	1	0	1		0	3.34
<20y	25-<30	117	97	2	1	2	3	1	2	0	1	1	2	0	1	2	0	3.69
20+yr	20+yr	103	93	1	2	1	2	2	3	1	1	1	1		1	2	1	1.98
20+yr		122	92	2	2	1	2		2	0	1	1	2	1	1	2	0	3.18
=35+	=35+	104	98	2		1	1	1		0	1	1	2	0	1		0	3.53
=35+	=35+	106	107	2		1	3	1	2	2	2	1	0	1	1		0	3.91
25-<30	=35+	114		2	2	2	2	2	1	1	1	1	2	0	1		0	2.48
<20y	25-<30	83	93	2	2	1	2	2	2	2	2	1	0	0	2			3.5
25-<30	30-<35	106	97		2	1	2	2	3	0	2	1	2		1			3.57

20+yr	<20y	91	85	2	2	2	2	2		1	2	2	2	1	1	3	1	3.2
=35+	30-<35	97	77	2	1	2	1	2	1	0	1	1	1	0	1	2	1	3.57
25-<30	25-<30	98	90	1	1	2	2	3	2	2	1	1	0	1	2	1	1	3.43
20+yr	<20y	103	105	1	1	1	2	1	1	0	1	1	1	0	1	3	1	3.52
30-<35	=35+	112	93	2	2	1	2	2	1	0	1	1	0	0	1	2	0	3.95
<20y	<20y	95		2	1	2	2	2	1	1	1	1	1	0	1	2	0	3.15
25-<30	30-<35	91	88	2	2	2	2	3	1	0	1	2	0	0	1	1	0	3.7
25-<30		107		1	2	1	3	3	1	0	1	2	2		1			2.8
30-<35	25-<30	97	93	2	1	2	2	2	2	0	1	1	2	0	1	2	0	4.15
<20y		106	97	1	2	1	2		2	0	2	1	1	0	1	3	0	3.78
<20y	<20y	114		1	1	2	2	3	1	0	2	1	1	0	2	1	0	2.938
<20y	<20y	103	106		1	1	3	3	3	0	1	1	1	0	1	1	0	3.81
20+yr	<20y	108	109	2	2	1	2	3	3	0	1	1	2	0	1	2	0	2.93
<20y	25-<30	105	103	2	2	2	2	2	1	2	1	2	2	0	1	2	0	3.47
20+yr	20+yr	95	96	2	2	2	2	2	1	1	1	1	0	0	1	2	1	2.85
25-<30	30-<35	115		2	1	2	3	3	3	2	1	1	1	0	1	3	1	2.95
25-<30	30-<35	110	96	2	1	2	3	3	2	0	1	1	1	0	2	2	0	3.08
=35+	=35+	117	93	2		1	2	2	2	0	1	1	2	1	1	2	0	3.59
<20y	25-<30	106	104	1	1	1	2	2	1	0	1	1	1	0	1	2	0	4.21
<20y	<20y	98	88	2	1	1	2	2	2	1	1	1	0	0	1	2	0	2.53
25-<30	25-<30	115	98	2	1	1	2	2	3	0	1	1	2	1	1		1	2.6
<20y	25-<30	85	87	1	1	2	1	1	1	2	1		0	0	1		0	3.13
30-<35	=35+	105	102	2	1	1	1	2	2	0	1	1	1	0	1	2	1	3.49
<20y	25-<30	117		2	1	2	2	2	2	0	1	1	1	0	2	2	0	3.115
<20y	<20y	108	89	2	1	2	2	2	1	2	1	1	2	0	1	2	0	4.06
25-<30	25-<30	88	98	2		1	2	2	1	0	1	1	1	1	1	3	1	3.52
20+yr	<20y	125		1	2	2	2	2	1	0	1	1	1		1			3.14
=35+	=35+	103		2	1	2	3	3	2	2	1	1	1	0	2	3	0	3.83

25-<30	25-<30	110	95	2	1	2	1	2	1	0	1	1	2	0	1	2	0	3.53
25-<30	30-<35	135	117	2	2	1	2	2	3	0	1	1	2	0	2	2	0	3.02
20+yr	<20y	100	97	2	2	1	2	3	1	1	1	1	1	0	2	2	0	3.59
25-<30	25-<30	103	95	2	1	2	2	2	3	0	1	1	2	1	1		0	2.63
20+yr	<20y	92	101	2	2	1	1	1	1	0	1	1	0	1	1	2	0	2.65
25-<30		107	108		1	1	2	2	1	0	1	1	0		1			3.15
25-<30	25-<30	123	97	2	2	1	2	2	3	1	1	1	1	0	1	3	1	3.78
20+yr	<20y	86		1	2	2	2	3	1	0	1	1	1	0	1	2	1	3.625
25-<30	=35+	115	89	2	1	2	2	1	2	0	1	1	2	0	1	2	1	3.37
<20y	25-<30	104			1	1	2	2	3	0	1	1	1	0	1	3	1	2.97
<20y	<20y	92	80	1	1	2	2	2	1	0	1	1	0	0	2	2	1	3.19
<20y	25-<30	115	106	2	1	1	2	3	2	0	1	1	1	0	1	3	0	3.8
25-<30	30-<35	102		2	2	2	1	2	1	1	1	1	2	0	1	2	0	2.99
<20y	25-<30	91	88	2	1	1	2	2	2	0	1	1	1	0	1	2	1	3.23
25-<30	25-<30	96	93	2	1	2	2	1	2	1	1	1		1		2	1	3.38
25-<30	=35+	114	103	1	1	2	2	3	2	0	2	1	2	0	1	2	0	3.09
<20y	25-<30	117	110	2	2	2	3	2	3	0	1	1	1	0	1	3	1	3.4
<20y	25-<30	102	110	2	1	2	3	2	2	0	1	1	1	0	1	2	0	3.91
20+yr	<20y	103	105	2	2	1	3	3	1	1	1	1	1	0	1		1	3.41
<20y	=35+	110			2	1	2	2	1	2	1	1						3.49
=35+	=35+	103	109	2	2	2	3	1	2	0	1	1	2	0	2	3	1	3.74
25-<30	30-<35	106	86	2	1	2	1	3	2	2	2	1	1		1	2	1	3.15
20+yr		99		2	2	2	1			2	2	1	0	0	1	2	1	3.64
<20y	<20y	94	97	2	1	2	2	2	2	0	1	1	2	0	1		0	3.59
=35+	=35+	115	75	2	1	1	1	1	1	0	1	1	2	0	2	1	1	3.17
25-<30	<20y	110	95		2	1	2	1	2	0	1	1	2	0	1	2	1	3.06
<20y	<20y	92	91	2	2	2	2	2	1	0	2	1	1	0	1	1	0	3.46
<20y	<20y	104		1	2	2	2	1	2	1	1	1	2	0	1	2	0	3.73

<20y	25-<30	114		1	2	1	3	3	3	0	1	1	0		2			3.93
20+yr	30-<35	113		2	2	1	2	3		2	1	1	1	0	1		1	2.83
=35+	=35+	102	103	2		2	2	1	1	0	1	1	2	0	1	2	0	3.92
25-<30	30-<35	103	95	2	1	2	2	2	2	0	1	1	1	0	1	2	0	3.64
25-<30	30-<35	101	92	1	2	1	2	1	2	0	1	1	0	0	2	1	1	3.88
<20y	<20y	100		1	2	2	2	2	1	2	2	1	0		1			3.59
=35+	=35+	101	87	2	2	1	2	2	1	1	1	1	2	1	1			3.25
30-<35	30-<35	89	95	2	1	2	2	2	3	0	1	1	1	0	1	2	1	3.91
25-<30	25-<30	117	106	2	1	2	2	2	2	0	1	1	2	0	1	2	1	3.02
20+yr		108	122	1	2	1	2		1	1	1	1	1	0	1	2	0	2.78
<20y	<20y	107		2	1	1	2	1	1	2	1	1	1	0	1	2	1	2.97
<20y	<20y	83	90	1	1	2	2	2	1	1	1	1	0	0	1	2	1	3.29
<20y	<20y	112	101	2	2	1	2	2	1	0	1	1						2.77
20+yr	<20y	101			1	2	2	2	1	0	1	1	2		1			3.6
30-<35	30-<35	107	93	2	1	1	2	2	2	1	2	1	0	0	1	3	1	3.61
<20y	<20y	87	94	1	2	1	2	2	2	0	1	1	1	0	1	2	1	3.36
<20y	25-<30	108	97	2	1	2	3	2	2	0	2	1	2	0	1			3.39
25-<30	=35+	74	79	2		2	2	2	2	2	2	1	0	0	1	2	1	2.6
<20y	<20y	118	93	2	2	2	1	2	3	0	1	1	2	0	1	2	1	3.05
25-<30	25-<30	85	82	2	2	2	1	2	1	2	1	1	1	1	1	2	1	3.02
<20y	<20y	106		1	2	2	3	2	2	1	1	1	0		1			2.9
20+yr	25-<30	91	82	2	2	2	2	2	2	0	1	1	2	1	1	3	0	3.9
<20y	30-<35	115		2	2	2	2	2	1	2	1	1	0	0	1	3	0	3.14
<20y	=35+	125		1	2	2	3	3	1	0	1	1	2	0	1	3	0	3.19
<20y	<20y	92	88	2	1	2	1	2	2	1	1	1	1		2	2	1	3.82
20+yr	<20y	88		1	2	2	2	2	1	1	1	1	1		1			3.26
25-<30	30-<35	105	103	2	1	1	2	2	2	0	2	1	2	0	1	2	1	3.17
20+yr	30-<35	118		1	1	1	2	2	1	0	1	1	0	1	1	2	0	2.94

25-<30	25-<30	97	110	2	2	2	2	3	1	1	1	1	1	0	1	2	1	3.73
=35+	=35+	110	97	2	2	2	2	3	2	0	1	1	2	0	1	3	1	3.2
20+yr	20+yr	90		2	2	1	2	2	1	1	1	1						3.6
25-<30	25-<30	88		2	2	1	3	3	3	0	1	1	2	0	1	2	1	3.1
<20y	25-<30	104	110	2	2	2	2	2	2	2	1	1	1	0	1	1	1	3.4
<20y	25-<30	103	87	1	2	2	1	2	1	1	1	1	0		2		1	3.205
25-<30	30-<35	115	115	2	1	1	3	2	3	0	1	1	1	0	2	2	0	4.18
30-<35	30-<35	96	114	1	1	2	2	2	1	0	1	1	0	1	1	2	1	4.6
<20y	30-<35	95	89	2	2	1	2	2	1	1	1	1	1	0	1			3.92
20+yr	<20y	98	94	1	2	1	2	1	2	0	1	1	0	0	2	2	1	3.35
20+yr	25-<30	84	96	1	1	2	2	2	2	0	1	1	1	0	1	2	0	4.4
<20y	<20y	89		1	2	2	2	3	1	0	1	1	1		1			2.335
25-<30	<20y	114		1	2	2	2	1	1	2	2	1	0	0	1	2	0	2.76
<20y	<20y	99	93	2	1	1	1	3		1	1	1	2	0	1	2	1	3.12
30-<35	30-<35	98	85	2	1	1	1	2	2	2	2	1	0	0	1	1	0	2.99
<20y	25-<30	118		2	2	2	2	2	2	2	2		1	0	1		0	3.08
<20y	<20y	120	100	1	2	2	3	3	1	1	1	2	1	1	2	1	0	3.81
<20y	<20y	117		2	1	2	2	2	2	0	1	1	2	0	1	2	1	3.41
<20y	<20y	103		1	1	1	2	2	3	0	1	1	2		1			3.625
25-<30	25-<30	109	96	2	2	2	2	2	2	0	2	1	2	0	1	2	1	3.92
30-<35	30-<35	99		2	1	2	2	3	3	0	1	1	2	0	1	3	0	3.95
25-<30	25-<30	78		2	2	1	2	2	3	2	1	1		0		2	1	4.81
<20y		98	89	1		2	2	2	1	2	2		1	0	1	2	0	3.55
20+yr	20+yr	100		1	2	1	2	2	1	0	2	1	1	0	1	2	1	3.82
30-<35	=35+	105	117	1	1	1	2	1	2	0	1	1	2	1	1	2	0	3.34
25-<30	25-<30	108		2	1	2	3	3	3	0	1	1	2	0	1	2	0	3.46
=35+	=35+	108	83	2	2	1	2	3	3	1		1	1	0	2	2	0	3.54
25-<30	25-<30	93	93	2	2	1	2	2	2	0	2	1	2	0	1	3	0	4.11

<20y	<20y	100		2	1	1	1	2	2	2	2	1	1	0	1	3	1	1.904
<20y	20+yr	95	100	2	2	2	3	2	2	0	1	1	2	0	1	2	0	4
25-<30	30-<35	102	84	1	1	2	1	1	2	2	1	1	1	0	1	3	1	2.51
20+yr		101		2	2	2	2		2	0	1	1	0	1	1	2	0	3.6
20+yr	<20y	91	92	1	2	1	2	1	1	1	2	1	1	0	1	2	0	3.33
25-<30	25-<30	105	112	2	2	1	2	2		2	1	1	1	0	1	2	0	2.75
20+yr	<20y	85		2	1	2	2	1	2	0	2	1	1	0	1	2	1	3.36
<20y	<20y	132	103	2	1	1	3	3	3	0	1	1	2	0	1	2	0	4.2
30-<35	30-<35	96	87	1	1	1	1	1	2	0	1	1	1	1	1	2	1	4.22
<20y	25-<30	120		2	2	2	2	2	3	0	1	1	2	0	2	2	0	3.17
30-<35	25-<30	129	97	2	1	1	2	3	3	0	1	1	0		1			3.02
<20y	25-<30	111	100	2	1	1	2	2	1	0	1	1	2	0	1	2	1	2.8
20+yr	<20y	114	104	2	1	1	3	2	2	0	1	1	2	0	1	2	0	4.33
<20y	30-<35	103		1	1	2	3	2	3	0	1	1	2	1	1	2	1	3.49
=35+	=35+	110	100	2	2	2	2	3	2	0	1	1	2	0	2	3	1	3.47
30-<35	=35+	112		2		2	2	2	2	0	1		0	0	1	2	1	3.76
<20y	<20y	105	100	1	2	2	3		1	0	1	2	1	1	1	2	1	3.14
<20y	<20y	120	108		2	2	2	3	2	1	1	1	1		1			3.92
30-<35	25-<30	115		2	1	1	2	2	2	0	1	1	2	0	1	2	0	3.58
<20y	25-<30	112	85	2	2	2	2	2	2	0	1	1	2		1			3.16
20+yr	<20y	95			2	2	2	1	1	2	1	1	1	0	1	2	0	2.58
25-<30	25-<30	95	117	2	2	1	2	1	2	0	1	1	1	1	1	2	0	3.02
=35+	=35+	96	61	2	1	1	3	1	2	0	1	1	2		1			3.24
<20y	25-<30	122	100		1	1	2	1	1	0	1	1	2	0	1		0	4.265
30-<35	30-<35	107		2	1	2	2	2	1	1	1	1	0	1	1	2	1	2.67
<20y		120		1	2	2	2	2	1	2	1	1	1	0	1	3	1	3.65
30-<35	=35+	111		2	2	1	3	3	2	0	1	1	2	0	1	3	1	3.9
<20y	25-<30	103		1	1	2	2	2	1	0	1	1	1	0	1		1	3.72

<20y	<20y	100	88	1	1	1	2	2	2	0	2	1	0		1			3.63
<20y	<20y	110	95	1	2	2	2	2	2	0	1	1	1	1	1	2	0	2.89
25-<30	30-<35	109	92	2	1	1	1	1	2	0	2	1	1		1			4.25
30-<35	30-<35	110	107	2	1	2	3	3	3	0	2	1	2	0	1	3	0	3.67
<20y	25-<30	114	86	2	1	1	1	2	3	1	2	1	1	0	1		0	3.65
25-<30	30-<35	118	117	2	1	1	3	3	3	1	2	1	2	0	1	2	0	3.08
25-<30	30-<35	104	106	2	2	2	2	3	2	1	2	1	2	1	1	2	1	3.09
25-<30	30-<35	114	119	2	2	2	2	2	1	0	1	1	0		1			2.85
25-<30	25-<30	114		2	1	1	2	3	1	0	1	1	2	0	1	2	1	3.81
30-<35	=35+	109	87	2		1	2	1	1	0	1	1	2	0	1			2.31
30-<35	30-<35	114	100	2	1	2	2	2	1	0	1	1	1	0	2	3	1	3.68
25-<30	25-<30	115		2	1	1	2	1	2	2	1	1	1	0	1	2	0	3.39
30-<35	=35+	91	86		1	1	1	2	3	2	1	1	0	0	1	1	0	3.12
25-<30	=35+	96	91	2	2	2	2	2	2	2	2	1	0	0	1	3	0	3.64
<20y	25-<30	120	101	2	2	2	3	2	3	0	1	1	2	1	1	1	0	3.4
<20y	<20y	100		2	2	1	2	2	2	0	2	1	2	0	1	2	1	3.58
25-<30	25-<30	108	88	2	2	1	3	3	3	0	2	1	1	0	1	2	0	3.17
25-<30	30-<35	109	92	2	2	2	3	2	2	0	1	1		1		2	1	2.88
<20y	<20y	82	88	2	2	1	2	1	1	2	1	2	1	0	2	2	0	3.08
25-<30	25-<30	97	95	2	1	1	2	1	2	0	1	1	1	0	1	2	1	3.645
=35+	=35+	102	102	2	1	2	2	2	2	0	1	1	2	0	1	2	0	4.33
25-<30	25-<30	111	112	2	2	2	3	3	2	0	1	1	1	0	1	2	0	3.14
25-<30	30-<35	97		1	2	1	2	3	3	0	1	1	2	0	1	2	0	3
30-<35	30-<35	107	103	2	2	2	3	3		0	1	1	2	0	1	2	1	3.69
20+yr	<20y	92	92	2	2	1	1	1	1	1	1	1	0	1	1	2	1	2.86
20+yr		93	105	1	2	1						1	1	0	1	2	0	3.89
25-<30	30-<35	107	108	2	2	1	3	3	2	0	1	1	1	0	2	2	1	3.92
<20y	<20y	102	94	1		1	3	3	1	1	2	1	2	0	1	3	0	3.61

25-<30	30-<35	112	104	2	2	1	2	2	3	0	1	1	2	1	1	2	1	3.88
<20y	25-<30	120	108	2	2	1	2	2	3	0	1	1	1	1	1	2	1	3.52
<20y	25-<30	100	100	2	1	2	3	2	2	0	2	1	1	0	1	2	1	3.43
<20y	<20y	100		2	1	2	3	2	2	0	1	1	1	0	1	2	0	4.17
30-<35	=35+	94	88	2	2	2	2	1	3	0	1	1	2	0	1		0	3.78
<20y	25-<30	112	92	2	1	2	2	2	1	2	1	1	1	0	1	2	0	2.69
20+yr	<20y	105	102	1	2	2	2	2	3	0	1	1	1	0	1	2	1	3.24
25-<30	25-<30	114	93	2	1	2	3	3	3	0	1	1	2	0	1	2	0	3.3
20+yr	<20y	95	100	2	2	2	2	2	2	2	2	1	1	0	1			3.1
30-<35	=35+	113	100	2	1	2	3	3	3	0	1	1	2	0	1	2	1	3.25
<20y	25-<30	103	95	2	1	1	2	2	2	0	1	1	0	0	1	3	0	3.62
25-<30	30-<35	114	93	1	1	1	2	1	3	1	1	1	0		1			3.96
<20y	=35+	115	114	1	2	1	2	1	2	2	1	1	2	0	1	2	0	3.23
<20y	<20y	107	110	2	1	1	2	1	2	1	2	1	0	0	1	2	1	1.24
25-<30	30-<35	111	101	2	2	2	2	2	2	0	1	1	1	0	1		1	3.14
<20y	25-<30	118		1	1	1	1	2	2	2	2	1		0		2	1	2.05
<20y	25-<30	101		2	1	2	3	3	3	0	1	1	2	0	1	2	1	3.01
<20y	25-<30	115	112	1	2	1	2	1	2	0	1	1	2	0	1	2	0	3.39
<20y	20+yr	100	93	1	2	1	2	2	1	1	1	1	2	0	1	3	0	3.49
25-<30	25-<30	91	90	1	1	1	3	3	2	0	1	1	0	1	1	2	1	3.85
30-<35	25-<30	123	102	2	1	1	2	3	3	0	1	1	2	0	1		0	3.55
=35+	=35+	91		2	1	2	1	2	2	0	1	1	2	1	1	2	0	3.63
20+yr	25-<30	94	98	2	1	1	2	2	1	2	2	1	1	0	1	2	1	2.5
30-<35	30-<35	105	107	2	2	2	2	2	1	0	1	1	2	0	1	2	1	3.54
<20y	30-<35	106	114	1	2	2	2	2	3	1	1	1	1		1			3.16
25-<30	25-<30	108	104	1	2	2	3	3	1	2	1	1	2	0	1	3	0	3.36
25-<30	<20y	92	83	2	1	2	1	2	2	0	1	1	0	0	1	3	1	2.72
25-<30	=35+	114	112	2	2	1	2	3	3	0	2	1	1		1	2	0	3.16

<20y	<20y	100	97	2	1	2	2	2	3	2	2	1	2	0	1	2	1	3.09
25-<30	25-<30	114	104	2	1	1	2	3	2	1	1	1	2	1	1	2	0	2.87
<20y	<20y	95	86	2	2	2	2	2	1	2	1	1	1	0	1	1		4.055
25-<30	25-<30	101	106	2	1	2	3	2	3	0	1	1	2	0	1	3	1	3.23
30-<35	=35+	100	95	2	2	2	2	3	2	0	1	1	1		1	1	0	3.01
<20y	<20y	92	90	2	2	1	2	2	2	1	1	1	1	0	1	2	0	2.27
25-<30	25-<30	99	80	1	2	2	2	2	1	2	1	2	0	0	2	2	1	3.04
25-<30	30-<35	109	95	2	1	1	2	2	3	0	1	1	1	0	1	3	0	2.82
25-<30	=35+	92	97	2	2	1	2	3	3	1	1	1	2		1			3.42
25-<30	30-<35	105	69		1	2	3	3	1	0	1	1	2		1			2.55
<20y	30-<35	117	110	1	2	2	3	2	2	0	1	1	2	0	1	2	1	4.08
25-<30	=35+	83	92	1	2	2	3	2		1	1	1	2	1	1		0	3.97
20+yr		110	84	1		1	2		1	0	1		1	0	2	2	1	3.84
25-<30	25-<30	93	98	2	1	1	2	3	2	0	1	1	1	0	1	2	1	3.07
<20y	<20y	110	103	2	1	2	2	2	1	0	1	1	2	0	1	1	0	3.08
30-<35	=35+	105	107	2	2	1	2	2	3	0	2	1	1	0	1	2	0	3.79
<20y	25-<30	105	88	2	1	2	2	2	2	0	1	1	0	0	1		0	3.51
25-<30	30-<35	104	88	2	1	2	2	2	2	0	1	1	0	0	2	2	1	3.75
=35+	=35+	106	90	1	2	2	2	1	1	0	1	1	0	0	1	2	1	3.19
25-<30	25-<30	132	104	2	2	1	3	3	2	0	1	1	2	0	2	2	1	4.09
<20y	25-<30	99	101	1	1	1	2	2	2	2	2	1	1	0	1	2	0	3.5
30-<35	=35+	105		2	2	2	1	2	2	0	1	1	2	0	2	2	0	3.37
20+yr	<20y	101	103	1	2	2	2	1	1	1	1	1	1	0	1	2	0	3.71
25-<30	=35+	101	110	2	2	2	2	1	2	0	1	1	2	0	1	3	1	3.97
<20y	<20y	97	101	1	1	2	2	2	2	2	1	1	1		1			3.85
=35+	=35+	100	103	2	2	1	2	3	2	0	1	1	2	0	1		1	2.025
25-<30	25-<30	99	106	2	2	1	3	2	1	0	1	1		0			1	3.62
25-<30	25-<30	103	98	2	1	1	3	3	2	0	1	1	2	0	2	2	0	3.98

<20y	<20y	101	101	2	1	1	2	2	2	1	2	1	1	1	1	3	1	3.57
<20y	<20y	103		2	1	1	2	2	3	0	1	1	0		1			2.47
<20y	25-<30	94	90	2	2	2	2	2	2	0	1	1	0	0	1	3	1	3.53
25-<30	25-<30	123	91	2	1	1	2	2	3	1		1	1	0	1	2	0	2.86
<20y	25-<30	94	82	2	1	2	2	2	1	0	1	2	0	0	1	2	1	2.56
25-<30	=35+	94	94	2	1	1	2	2	3	0	1	1	2	0	2	2	1	3.82
20+yr	<20y	95		1	2	2	2	2	3	0	1	1	1		1			2.78
30-<35	25-<30	103	85		2	2	1	2	1	0	1	1	0	0	1	2	1	3.15
25-<30	30-<35	114	108	2	1	1	2	3	1	2	2	1	2	0	1	2	0	3.28
20+yr	<20y	91	101	2	1	2	2	2	3	0	1	1	2	1	1	2	0	3.42
<20y	25-<30	101	103	2	2	2	2	1	2	2	1	1	0	0	1	2	0	3.03
25-<30	25-<30	94	91	2	1	2	2	2	1	0	1	1	1	0	1		1	4.13
20+yr	20+yr	100		1	2	2	2	2	1	0	1	1	1	0	2	3	0	3.76
=35+	30-<35	94	90	1	2	1	1	3	2	0	1	1	1	1	1	2	0	4.62
<20y	30-<35	99	93	2	1	1	2	2	3	0	1	1	2		1			3.09
<20y	25-<30	106	85	2	2	2	3	1	3	0	1	1	2	1	1	2	0	3.16
20+yr	<20y	103			2	1	1	2	2	2	1	1						3.05
=35+	=35+	112	90	2	2	2	2	2	2	0	1	1	0	0	1	3	0	4.16
20+yr		102		1	2	2	2		2	0	1	1	0	1	1	3	1	3.98
<20y		86			2	1	1		1			1	0		1			3.04
20+yr	<20y	96	102	2	1	1	2	2	1	1	1	1	2	0	1	2	0	3.5
25-<30	30-<35	114	101			2	2	2		0	1		1	0	1	2	1	3.98
<20y	25-<30	114	100	2	1	1	2	2	2	2	1	1		0			1	3.13
<20y	<20y	96	104	1	2	2	3	3	3	0	1	1	1		1			3.09
25-<30	30-<35	100	96	2	1	1	2	2	3	0	2	1	2	0	1	2	0	2.92
25-<30	25-<30	102	110	2	1	2	2	2	2	0	1	1	2	1	1	2	0	3.16
30-<35	=35+	120		1	2	2	1	1	1	0	1	1	2	0	2		0	3.2
<20y	<20y	90	86	1	1	2	1	1	2	2	1	1	1	0	1	2	0	3.07

<20y	25-<30	98	82	2	1	1	1	3	2	0	2	1	1	0	1	1	1	3.68
30-<35	25-<30	90	92	2	1	1	2	2	2	0	2	1	2	0	1	2	0	3.72
25-<30	25-<30	104	101	2	2	2	2	3	2	1	2	1	1	1	1	2	0	2.715
<20y	30-<35	103		2	2	2	2	2	3	2	1	1	1		1			3.51
20+yr	25-<30	111	100	1	2	1	2	3	3	0	1	1		0		3	0	3.35
<20y	25-<30	118	88	1	1	2	2	1	3	0	1	1	1	1	1	3	0	4.04
<20y	<20y	87	81	1	2	2	1		1	1	2	1	1	1	2	2	1	2.68
<20y	<20y	111		2	1	2	3	3	2	0	1	1	2	0	1	2	0	4.06
20+yr	25-<30	92		1	1	1	2	2	1	0	1	1	1	0	1	2	1	2.78
<20y	<20y	85		2	2	1	2	2	1	0	1	1	1	0	1	3	1	2.69
=35+	=35+	104	124	1	2	1	2	2	3	0	1	1	2	0	1	2	0	3
<20y	30-<35	108	88	2	2	1	2	2	2	2	1	1	1	0	1	2	0	3.58
20+yr	<20y	108		1	2	2	2	2	3	0	1	1	1	1	1		1	2.65
25-<30	30-<35	90	87	2	1	1	2	2	1	2	1	1	0	0	1	2	0	3.46
<20y	25-<30	100	95		2	2	1	2	2		1	1	2	0	2	2	1	3.03
<20y	<20y	102		2	1	2	2	2	2	0	2	1	1	0	1	2	0	3.07
30-<35	30-<35	105		2	1	2	2	2	3	0	1	1	0	0	1	2	1	2.95
20+yr	<20y	109	99	1	2	2	2	2	2	0	2	1	2	1	1	1	0	3.86
25-<30	30-<35	90	88	2	2	2	2	2	3	0	1	1	1	1	2	2	1	4.15
<20y	<20y	120		1	2	1	2	1	3	1	2	1	1		1			3.37
30-<35	=35+	112		1	2	1	2	2	2	0	2	1	0	1	1	2	0	4.2
<20y	25-<30	110	110	2	1	1	2	1	2	0	1	1	1	0	1	2	0	2.92
20+yr	<20y	86		2	2	2	2	1	2	0	1	1	1	1	1	2	1	3.9
25-<30	25-<30	112	110	1	2	1	2	2	3	2	2	1	1		2			2.28
<20y	<20y	100	103	2	2	1	2	2	2	1	1	1	1	0	1	2	1	3.82
<20y	25-<30	94		1	2	1	2	2	1	2	1	2	1		1			3.82
20+yr	<20y	95	104	1	2	2	3	2	2	0	1	1	1	0	1	2	1	3.1
30-<35	30-<35	117	79	2		1	1	1	1	0	1	1	2	0	1	2	1	3.26

<20y	25-<30	117		2	1	1	3	2	3	1	1	1	2	0	1	2	1	2.79
<20y	30-<35	114	85	1	2	1	3	1	1	0	1	1	1	0	1	3	0	4.19
20+yr	20+yr	109	102	1	2	2	2	1	1	1	1	1	1	0	1	1	1	3.45
25-<30	30-<35	113	98	2	2	1	3	3	1	0	1	1	2	1	1	2	1	4.15
30-<35	30-<35	111	95	2		1	2	2	1	0	1		2	0	1	3	1	3.38
25-<30	=35+	68			2	1	3	1	1	0	1	2		1		2	1	4.13
<20y	30-<35	97	89	1		1	1	1	1	2	1		0	0	2		0	2.92
20+yr		99	100	2	2	2	1			0	1	1	2		1			3.34
<20y	25-<30	97	97	2	2	2	1	2	2	0	1	1	1	1	1	3	0	3.19
<20y	25-<30	115	108	2	2	2	3	3	3	0	1	1	2	0	1	2	1	4.2
25-<30	=35+	106	77	1		1	2		3	2	2	1	0		1			3.47
30-<35	=35+	103	91		2	2	3	2	2	1	1	1	1	1	2		0	3.13
30-<35	=35+	99	91	2	1	1	2	2	2	0	2	1	2	0	1	2	0	3.55
<20y	25-<30	107	91	2	2	1	1	1	1	0	1	1	2	0	1	2	1	2.486
30-<35	<20y	100	103	2	2	2	2	2	1	2	1	2	2	0	1	2	0	3.5
25-<30	25-<30	98	98	2	2	2	3	3	3	0	1	1	1	0	1		0	3.6
<20y	=35+	100		2	1	1	2	2	3	0	1	1	1		1			2.08
<20y	<20y	101	92	2	2	1	2	2	1	1	1	1	1	1	1	2	1	3.26
20+yr	<20y	80	92	1	2	1	2	2	1	1	2	1	1	0	1	2	1	2.34
<20y	<20y	97	110	2	2	2	2	3	3	1	1	1	1	1	1	2	1	3.06
25-<30	25-<30	109	96	2	2	2	2	2	2	0	1	1		1		2	1	4
<20y	25-<30	115	101	2	1	2	2	2	2	0	1	1	1	0	1	2	0	3.46
30-<35	30-<35	105	95	2	2	2	2	1	1	0	1	1	2	0	1	1	1	3.89
25-<30	=35+	82	108	2	2	1	2	3	3	0	1	1	1	0	1	2	1	3.72
25-<30	=35+	114		2	1	2	1	3	3	0	1	1	1	1	2	2	1	3.07
30-<35	30-<35	94	85	2	1	2	2	2	3	0	1	1	1	0	1	2	0	3.11
<20y	<20y	99		1	1	1	2	2	1	0	2	1	0	1	1	2	1	4.11
30-<35	30-<35	104	91	1		1	2	2	3	2	1	1	1	0	1	2	1	3.22

<20y	<20y	115	112	2	2	2	3	3	1	0	1	1	1	0	1	2	0	2.89
25-<30	30-<35	117	100	2	1	2	2	2	3	0	2	1	2	1	1	2	0	2.855
30-<35	30-<35	117		2	2	2	1	1	3	0	1	1	1	0	1	2	1	3.49
20+yr	20+yr	91	101	1	2	1	2	2	2	0	1	1	1	0	1	1	1	3.12
30-<35	30-<35	104	81	2	2	1	1	3	2	1	1	1	2	0	1	3	0	4.27
20+yr	<20y	106		2	2	2	1	2	1	2	2	1						2.97
25-<30	30-<35	94	80	2	1	2	2	2	1	0	1	1	0	0	1	3	1	3.69
25-<30	30-<35	107	106		2	1	1	2	2	0	1	1	1		1			3.27
<20y	<20y	117	108	1	1	1	3	3	2	2	1	1	2	0	1	2	0	3.78
=35+	=35+	96		2	2	2	1	1	2	0	1	1	1	0	1			3.55
25-<30	25-<30	85			2	2	2	2	2	0	1	1	2	0	1	2	0	3.45
25-<30	25-<30	110		2	2	2	2	3	3	0	1	1	2	0	1	2	0	2.75
<20y	<20y	90	82	1	1	1	2	2	2	0	2	1	0		1			2.45
<20y	25-<30	111		1	2	2	2	1	1	0	1	1	2		1			3.8
20+yr	<20y	106	95	1	2	1	2	2	3	0	1	1	2	0	1			3.5
25-<30	30-<35	114	90	2	2	1	2	3	2	0	1	1	2	0	1	2		3.25
20+yr	20+yr	90	98	1	2	1	2	2	1	0	1	1	1	0	1	2	1	3
30-<35	=35+	114	100	2	2	2	2	1	3	0	1	1		0		3	1	3.38
<20y	<20y	102	98	2	1	2	2	2	2	0	1	1	2	0	1	2	0	3.52
<20y	25-<30	92	94	1	2	1	2	2	2	0	1	1	2	1	1	3	1	3.63
25-<30	25-<30	115		2	2	2	3	3	2	0	1	1	2	0	1	2	1	3.28
=35+	=35+	115		2	1	2	2	1	2	0	1	1	2	0	1		0	4.01
25-<30	20+yr	103	97	2	2	1	2	2	1	1	2	1	2	0	1	3	0	3.92
25-<30	25-<30	106	94	1	2	1	3	3	2	0	1	1	2	0	2	3	0	3.96
<20y	=35+	108		1	2	1	2	2	3	0	1	1	0	0	2	2	1	3.8
<20y	<20y	94	90	2	1	1	2	1	2	1	1	1	0	0	1	3	1	2.93
30-<35	<20y	103	93	2	1	2	2		1	0	1	1	1	0	1		0	4.26
<20y	<20y	112	108	1	1	2	2	2	1	0	1	1						3.69

=35+	=35+	122		2	2	2	3	3	1	0	1	1	2	0	1			4.78
30-<35		100	102	1	2	2	1		1	1	2	1	1		1			4.05
30-<35	=35+	117	98	1	1	2	2	1	3	1	2	1		0				3.13
<20y	25-<30	106	98	2	2	2	2	3	2	0	1	1	0	0	1	3	0	3.64
30-<35	=35+	109	83	2	2	2	3	1	2	0	1	1	2	0	2	1	1	2.87
20+yr	20+yr	91	97	1	1	1	1	2	1	0	2	2	1	0	1	2	1	3.62
25-<30	25-<30	99	96	1	2	2	2	2	3	2	2	1	1	1	1	1	0	3.24
30-<35	=35+	117	94	2	2	1	3	2	1	0	1	1	0	0	1	1	1	3.71
25-<30	30-<35	108	96	2	2	2	3	3	2	0	1	1	2	1	1	3	1	3.51
20+yr	<20y	114		1	1	1	2	2	2	0	1	1	2	0	1	2	0	3.36
25-<30	25-<30	110		1	1	2	3	3	2	1	1	1	2		1			3.36
<20y	20+yr	95	91	2	2	1	2	2	3	0	1	1	1	0	1		0	2.7
30-<35	30-<35	117		2	1	1	2	2	3	0	1	1	1	0	1	3	1	3.92
25-<30	25-<30	99		2	1	2	2	3	2	0	1	1	2	0	2	3	0	2.73
30-<35	30-<35	108	87	1	1	2	2	2	2	0	1	1	0	0	2	2	0	3.94
30-<35	30-<35	112	100	2	1	1	2	2	3	1	1	1	1	0	1		1	3.67
<20y	<20y	97	87	2	1	1	1	1	1	0	1	1	1	0	2	2	1	2.76
20+yr	<20y	103	94	1	2	1	2	2		0	1	1	1	0	1	2	0	3.88
<20y	25-<30	122	91	2	1	1	3	3	3	0	1	1	2	0	1	2	1	3.28
30-<35	<20y	120	102	2	2	2	1	2	2	1	2	1	2	0	1		0	3.22
25-<30	25-<30	104		2	2	2	3	2	2	0	1	1	2	0	1	3	1	3.85
30-<35	=35+	107	83		1	1	3	1	3	0	1	1	2		1	2	0	2.67
20+yr	<20y	96	88	2	2	1	2	2	1	1	1	1	2	0	1		1	3.6
20+yr	<20y	100	106	2	1	1	2	2	3	0	1	1	2	0	1	2	0	3
=35+	=35+	118	78	1		2	3	3	3	0	1	1	1	1	1	3	1	3.64
<20y	<20y	107		1	2	2	2	2	3	0	1	1	0	1	1	3	0	3.48
25-<30	30-<35	112	92	2	1	1	2	2	3	0	1	1	2	1	1	3	0	3.31
25-<30	25-<30	114	119	1	1	2	1	1	2	0	2	1	2	0	1	3	0	3.14

25-<30	30-<35	101	91	2	2	2	2	2	3	1	2	1	2	0	1	2	0	3.65
20+yr		103	98	1	2	2	3		1	0	1	1	2	1	1	1	0	4
<20y	25-<30	118	108	2	2	1	3	2	2	0	1	1	2	0	2	3	1	3.3
<20y	25-<30	95	98	2	1	2	3	2	1	1	1	1	2	1	1	3	1	3.74
20+yr	20+yr	95	81		1	1	2	2		0	1	2	2	0	1	2	1	3.66
<20y	<20y	112	108	2	1	1	2	1	2	1	1	1	2	0	1		0	4.27
<20y	25-<30	97	92	2	2	2	2	2	3	0	1	1	2	0	1	2	0	2.835
25-<30	25-<30	75		2	1	2	2	2	2	0	2	1	1	0	1	2	1	3.22
30-<35	25-<30	122	102	2	1	2	2	3	2	0	1	1	2	0	2	2	0	3.775
30-<35	=35+	108	105	1	2	2	3	3	2	0	1	1	2	0	1	2	0	3.34
<20y	25-<30	112	103	1	2	1	2	3	2	0	1	1	2	0	2			3.92
20+yr		102	96	1	2	2	2		1	0	1	2	0	1	1	2	1	3.43
25-<30	25-<30	132	122	1	1	1	3	3	2	2	2	1	2	1	1	3	1	3.09
20+yr	<20y	103		2	1	2	2	2		0	1	1	0	1	2	1	0	3.14
<20y	<20y	93	98	2	1	2	2	2	2	0	1	1	1	1	1	2	0	3.4
<20y	25-<30	106	77	2	1	1	2	3	1	0	1	1	2		2			4.54
<20y	25-<30	93	97	2	2	2	2	2	2	1	1	1	2	1	2	3	0	2.74
30-<35	30-<35	105	97	2	1	2	2	2	2	0	1	1	2	1	1	3	1	3.17
<20y	30-<35	92			1	1	1	2	1	0	1	1	2	0	1	1	0	5.07
<20y	=35+	95	101	2	1	1	2	3	3	1	1	1	1	0	1	2	1	3.26
25-<30	<20y	114	100	2	2	2	2	2	3	0	1	1	2	1	1	3	1	3.32
<20y	<20y	129	110	2	1	1	3	3	2	0	1	1	2	0	1	2	1	3.46
20+yr		98	79	1	2	2	1	2	1	0	1	1	0	1	1		0	3.73
<20y		118	112	1	2	1	2		1	1	1	1	1	1	1	2	1	3.58
<20y	25-<30	101	97	2	1	2	2	2	3	2	2	1	1	1	1	1	0	3.56
25-<30	25-<30	127	139	2	1	1	3	3	3	0	1	1	1	0	1	2	0	3.98
30-<35	30-<35	100	120	1	2	2	2	2	3	0	1	1	1		1			3
25-<30	25-<30	100	101	2	2	2	3	2	2	0	1	1	2	1	1	3	0	3.5

25-<30	25-<30	100		2	1	2	2	2	3	0	2	1	2	0	1	2	0	3.44
<20y	<20y	90	104	2	1	1	3	2	3	0	1	1	1	1	1	2	1	3.895
20+yr	<20y	91	100	2	2	1	1	2	2	1	1	1	0	0	2	2	1	3.22
30-<35	30-<35	112		2	1	2	2	1	2	1	1	1	1	0	1	3	1	2.99
25-<30	30-<35	115	101	2	2	1	2	2	2	0	1	1	2	1	1	2	0	3.9
<20y	<20y	112	95	2	1	2	1	2	2			1	1	1	1	3	1	3.42
<20y	25-<30	129	110	2	2	1	3	2	2	2	1	1	2	0	1	2		4.05
=35+	=35+	112	102	2	1	1	3	2		0	1	1	1	0	1	2	1	5.28
<20y	25-<30	98	82	2	2	2	2	2	2	0	1	1	0	1	1	2	1	3.03
20+yr		97	92	1	2	2	2		2	0	1	1	1	1	1	2	0	3.58
25-<30	30-<35	114	98	1	1	2	2	3	2	2	1	1	1	0	1	3	1	2.84
25-<30	30-<35	105	100	2	1	1	2	2	2	0	1	1	1		1			3.67
<20y	<20y	108	110	2	1	1	2	2	2	2	1	1	2	1	1	2	0	3.91
25-<30	25-<30	97	101	2	1	2	1	2	3	0	1	1	1	0	1	2	0	3.75
25-<30	30-<35	82	94	1	1	1	1	2	1	2	2	1						3.48
25-<30	=35+	98		1	1	2	2	2	1	0	1	1		0		3	1	3.29
=35+	=35+	103	95	2	2	1	2	2	3	0	1	1	2	1	1			3.355
<20y	<20y	104	96	1	1	1	1	2	1	2	1	1	1	1	1	2	0	3.1
<20y	25-<30	123	114	1	2	2	3	3	3	0	2	1	2	0	1	2	0	3.25
20+yr	25-<30	109	92	2	2	1	1	2	1	2	2	1	0	0	1	2	1	2.58
<20y	<20y	101		1	2	2	2	2	1	0	2	1	1	1	1	2	1	3.05
20+yr	25-<30	109		1	2	2	2	2	1	0	1	1	1	0	1	2	1	3.47
<20y	25-<30	129	106	1	2	2	2	3	3	0	1	1	1	0	1	2	1	3.81
30-<35	=35+	91	97	2	1	2	2	1	2	2	2	1	1	1	1	1	0	3.37
25-<30	25-<30	92		1	2	2	1	2	1	0	2	1	1	1	1	3	0	3.76
25-<30	=35+	94	89	2	1	2	1	2	3	0	2	1	1	0	1	2	0	4.25
30-<35	30-<35	89	82	1	2	1	2	2	2	0	2	1	0	0	2			3.54
20+yr	<20y	82		1	2	1	2	2	3	2	2	1	1	0	1	3	1	3.54

<20y	<20y	81	90		1	1	2	2	2	2	1	1	1	0	1	2	0	3.33
<20y	<20y	97		2	1	2	2	2	1	1	1	1	2		1			3.11
30-<35	=35+	124		2	2	2	1	1	2	0	1	1	2	0	1	3	1	4.24
25-<30	30-<35	109	108	2	1	1	2	3	2	0	2	1	2	1	1	2	1	3.6
<20y	25-<30	114	103	1	1	1	2	2	2	0	1	1	1	0	1	2	1	3.54
25-<30	25-<30	118	106	2	2	1	3	3	3	1	1	1	2	1	1	3	0	2.55
20+yr	<20y	97		2	2	2	1	1	2	2	2	1	1	1	1	2	1	3.56
<20y	30-<35	91	108	2	2	1	2	2	1	0	1	1	2	1	1	2	0	3.22
20+yr	<20y	108			2	1	2	1	3	1	1	1	1		1			2.91
25-<30	30-<35	109	106		2	1	2	2	2	2	1	1	0		1			2.81
<20y		95	101	1		2	1			1	1		2	0	1	2	1	4.59
<20y	25-<30	120	94	2	1	2	2	2	2	0	1	1	1	1	1	2	0	3.96
20+yr	<20y	85		1	2	2	3	2	1	0	2	2	1	0	1	2	0	3.09
25-<30	25-<30	101	106	1	1	2	2	2	3	0	1	1	1	0	1	1	1	3.02
<20y	<20y	82	87	2	1	1	1	2	1	1	2	1	1	0	1	2	0	4.21
<20y	<20y	107		2	2	2	1	2	2	0	1	1	1	0	1	2	0	4.18
<20y	25-<30	117		2	1	2	3	3	2	0	1	1	2	0	1	1		2.99
<20y	<20y	117	104	2	2	2	2	3	2	0	1	1	2	1	1	2	0	3.8
20+yr	20+yr	107	98	2	2	1	3	2	1	0	1	1	1	0	1	2	0	3.11
25-<30	<20y	127	103	2	1	1	2	2	2	1	1	1	2	1	2	2	1	3.18
20+yr	<20y	108	88	1	2	2	2	2	1	1	1	1	1	1	1	2	1	3.32
<20y	<20y	106	90	2	2	1	1	1	1	2	1	1	1	1	1	2	1	3.24
<20y	<20y	97		1	2	2	2	1	3	1	2	1	2		1			3
30-<35	30-<35	97	90	2	2	2	1	2	2	0	1	1	2	0	1	2	1	3.94
25-<30	<20y	111		2	2	2	3	2	3	0	1	1	2	1	1	2	1	3.7
25-<30	25-<30	112	112	2	1	1	2	2	3	0	1	1	2	0	1	2	0	2.99
=35+	=35+	95	85	2		1	1	1	1	0	1	1	1	1	1	2	1	4.42
30-<35	30-<35	112	79	2	2	2	3	3	1	1	1	2	0	1	1	3	1	3.48

=35+	=35+	112	71	2	2	1	3	2	2	0	1	1	1	0	1	3	1	4.045
30-<35	25-<30	100	105	2	2	1	3	2	3	0	1	1	2	0	1	2	0	3.59
25-<30	30-<35	107	87	2	1	1	2	1	2	0	1		1		1	2		4.355
<20y	25-<30	105	100	1	1	2	2	2	2	0	1	1	1	1	1	2	1	3.39
<20y	<20y	92	98	1	2	1	2	2	3	2	1	1	1	0	1	2	1	3.68
25-<30	30-<35	110	96	2	2	2	2	3	2	1	2	1	2	1	1	3	0	2.99
20+yr	<20y	114			2	2	2	2	2	1	1	1	2		2			3.35
30-<35	30-<35	105		2	2	1	3	3	2	0	1	1	2	1	2	2	1	2.96
<20y	<20y	108	88	2	2	1	2	3	3	1	1	1	0	1	1	2	0	4.32
<20y	<20y	109	87	2	1	1	2	1	3	0	1	1	1	1	1	3	0	3.37
<20y	25-<30	107	97	2	1	2	2	2	1	0	2	1	2	1	1	3	0	3.81
<20y	25-<30	104	88	2	1	1	2	2	1	2	2	1	1	0	1	2	1	3.95
25-<30	30-<35	102	110	1	1	2	2	1	2	1	2	1	1	1	1	2	0	3.31
<20y	25-<30	115	103	1	2	2	2	3	3	1	1	1	2	0	1	3	1	1.72
=35+	=35+	105	114	2	2	1	1	1	3	0	2	1	2	1	1	2	1	3.26
<20y	<20y	103	81	2	2	1	2	2	2	0	2	1	0	1	1	2	1	3.23
30-<35	30-<35	106	102	2	1	2	2	1	1	0	1	1	2	1	2		0	2.79
20+yr	<20y	79	87	2	2	2	2	2	3	2	1	1	1		1	2	0	3.66
<20y	25-<30	83	87	1	2	1	2	2	1	0	2	1	0		2			3.76
30-<35	=35+	123	112	2	2	2	3		2	0	1	1	2	1	2	3	0	2.91
30-<35	30-<35	114	109	1	2	1	2	2	3	2	2	1	1		1			2.69
20+yr	30-<35	94	90	1	1	1	1	2	1	0	1	1	0	0	1			3.035
<20y	25-<30	115	112	2	2	2	3	3	1	0	1	1	2	0	1	3	0	4.12
20+yr		99		1	2	2	3	2	1	0	1	1	2	1	1	2	0	4.4
25-<30	30-<35	114	110	2	1	2	1	1	1	0	1	1	2	1	1	2	0	3.97
20+yr		96	90	1	2	2	2		1	0	1	1	1	1	1	2	1	3.63
30-<35	30-<35	83	93	2	2	1	3	1	2	1	1	1	2	1	2	2	0	2.94
20+yr		98	88	1	2	2	2		1	2	1	1	1	1	2	2	1	3.38

25-<30	25-<30	115		2	1	2	3	2	2	2	1	1	2	1	1	2	1	3.08
20+yr		105	100	1	2	1	2			2	1	2	0	0	1	3	0	3.07
<20y	<20y	103	92	1	1	2	2	2	1	0	1	1	1	0	1	1	1	3.585
20+yr	<20y	110	109	1	1	2	2	2	2	0	1	1	1		1			3.11
25-<30	=35+	102		2	1	2	2	2	3	1	1	1	0	1	2	3	0	3.76
25-<30	30-<35	82	104	2	1	1	2	2	1	0	1	1	2	1	1	2	0	2.95
<20y	<20y	103		1	2	2	1	2	1	1	1	1	1	1	1	3	0	2.79
30-<35	25-<30	105	82	2	2	1	3	1	1	0	1	1	0	0	2	2	1	3.35
<20y	25-<30	111	110	2	1	1	3	3	3	0	1	1	2	0	1	3	1	3.34
20+yr	20+yr	104		1	2	2	2	2	1	1	1	1	0	0	1	2	1	2.47
25-<30	25-<30	101	98	1	1	1	2	1	2	0	1	1	2	0	1	2	1	3.48
30-<35	30-<35	98	102	2	1	2	3	2	1	0	1	1	2	0	1	2	0	3.74
25-<30	=35+	117		2	1	1	3	3	3	1	1	1	1	1	1	2	1	3.47
20+yr	<20y	108	96	2	2	1	2	2	2	0	1	1	2	1	1	2	0	3.39
25-<30	25-<30	105	119	2	2	2	2	3	3	0	1	1	2	1	1	3	0	2.95
25-<30	30-<35	109	108	2	2	2	1	1	3	1	1	1	1	0	1	3	0	3.12
25-<30	25-<30	105	101	1	2	1	2	1	1	2	1	1	1	1	1	2	0	3.9
20+yr	20+yr	101	98	2	2	1	2	2	2	0	1	1	0		1			3.34
20+yr	<20y	105	95	2	1	2	2	2	1	0	1	1	1		1			2.52
<20y	25-<30	95		1	2	2	2	2	2	1	1	1	2	0	1	3	0	2.36
<20y	25-<30	100	87	2	2	1	2	2	2	2	1	1	0	0	2	2	0	2.155
<20y	25-<30	92	98	2	1	1	2	2	3	0	2	1	1	1	1	2	1	4.42
25-<30	25-<30	97		1	2	1	2	3	2	2	1	1	1	0	1	2	0	2.8
30-<35	30-<35	108	95	2	1	2	2	2	1	2	1	1	1	1	1	2	1	3.09
<20y	<20y	110	94	1	1	1	3	2	1	1	1	1	2	1	1	2	1	3.64
<20y	<20y	106	84	2		1	1	1	3	0	2	1	0	0	1	1	1	2.99
25-<30	25-<30	106	110	2	1	2	2	3	1	0	1	1	2	1	2	2	1	3.81
<20y	30-<35	91	108	2	1	2	2	2	3	0	2	1	0	1	1	2	1	3.76

30-<35	30-<35	102	95	2	1	1	2	2		0		1	1	1	1	3	0	4.05
<20y	25-<30	97	106	1	2	2	3	2	3	0	1	1	2	0	2	2	0	4.03
25-<30	25-<30	104	98	2	2	1	3	2	3	1	2	1	1	1	1	2	0	2.28
25-<30	30-<35	102	87	2	2	2	2	1		0	1	1	2	0	1			4.48
<20y	<20y	118	112	1	2	1	2	2	3	0	1	1	2		1			2.95
<20y	<20y	107	125	2	2	1	2	2	2	1	1	1	1	0	1	2	0	3.54
<20y	<20y	98	88	2	2	2	2	2	1	1	2	1	1	1	1		0	3.29
<20y	<20y	101	108	2	1	1	2	2	3	0	1	1	2	0	1	2	0	4.02
20+yr	25-<30	84	98	1	1	1	1	1	1	0	1	1	1	1	1	3	0	3.09
30-<35	25-<30	40	44		1	1	1	1	3	2	2	1	0		1			1.98
<20y	=35+	109		2	2	2	2		1	0	1	1	2	1	2	3	0	3.21
<20y	20+yr	115	92	1	2	1	2	2	1	1	2	1	2	1	2	2	0	3.24
25-<30	=35+	101	106	2	1	1	2	2	2	0	2	1	1	1	1	2	1	3.09
25-<30	25-<30	105	112	2	2	2	2	1	1	0	2	1		0		3	0	3.195
30-<35	25-<30	100		1	1	2	2	3	2	2	1	2	0		1	2	1	3.53
<20y	<20y	118		2	1	2	2	3	2	0	1	1	0	1	1	3	0	3.11
<20y	<20y	103	110	2	1	2	2	2	2	0	1	1	2	1	1	3	0	4.05
<20y	25-<30	102	95	2	1	2	2	2	2	0	1	1	1	0	1	3	0	3.2
30-<35	30-<35	110	109	2	2	2	3	3	3	2	1	1	1	1	1	2	1	3.66
25-<30	30-<35	84	108	1	2	2	2	1	1	0	1	1	2	1	2	1		3.03
<20y	30-<35	99	96	2	1	2	2	1	2	0	1	1	2		1			3.67
<20y	=35+	105	84	1	1	2	2	3	2		1	1	0		1	2	1	3.82
25-<30	25-<30	120		1	2	1	3	3	3	2	2	1	1	1	1	2	1	2.99
30-<35	=35+	120	89	2	2	1	2	1	2	0	2	1	2	1	1	3	1	3.545
=35+	30-<35	97	97	2	1	2			2	1	1	1	1	0	1	1	1	3.05
25-<30	30-<35	115	84	2	1	1	1	2	1	1	1	1	1	1	1	3	1	3.77
<20y	25-<30	112	114	2	2	2	3	2	3	0	1	1	2	1	1	2	1	3.5
25-<30	25-<30	102	100	2	2	1	2	3	2	0	1	1	2	1	1	2	1	4.01

<20y	25-<30	107	96	2	1	2	2	2	3	1	1	1	1	1	1	2	0	3.52
<20y	<20y	114	100	2	1	1	2	3	2	0	1	1	2	1	1	2	0	3.83
<20y	<20y	100	94	1	1	2	2	2	1	0	1	1	2	1	1	3	1	3.57
20+yr	<20y	88	102	1	2	2	2	2	1	1	1	1		1		2	1	2.1
25-<30	25-<30	103		2	1	2	2	2	2	0	1	1	2	1	1	3	0	3.95
25-<30	25-<30	99	88			2	1	1	1	0	2	1	0	1	1			3.9
<20y	25-<30	110	90	1	2	1	2	2	1	2	1	1	2	1	1		1	2.73
<20y	<20y	103	80	2	1	1	1	2	1	2	2	1	1		1			2.7
20+yr	<20y	102		1	2	1	1	2	1	1	2	1	1		1	1	0	3.645
<20y	25-<30	110	108	2	1	2	2	3	2	0	1	1	2	1	1	2	1	3.7
<20y	<20y	120		1	2	2	2	1	3	0	1	1	2	0	1	2	1	3.16
<20y	25-<30	110	114	2	1	2	3	3	1	0	1	1	2	0	1	2	1	3.36
<20y	<20y	91		1	2	2	2	2	3	0	2	1	2	1	1	2	1	3.48
20+yr	<20y	106	88	2	2	1	2	3	3	1	1	1	1	1	1	2	1	3.54
25-<30	30-<35	92		2	2	1	3	3	1	2	2	1	0	0	1	3	0	3.06
25-<30	25-<30	109	108	2		1	2	1	3	0	1	1	2	1	1	2	0	3.8
<20y	25-<30	100	103	2	2	2	2	2	2	1	1	1	2	0	1	2	0	1.582
<20y	25-<30	98	91	2	1	1	2	2	2	2	2	1	2	1	1	2	1	3.16
20+yr	20+yr	100	90	1	2	2	2	2	2	0	1	1	2		2	2	1	3.85
<20y	25-<30	99		1	2	2	2	3		0	1	2	1	0	1	2	1	3.51
<20y		92	85	2	2	2	2			0	2	1	2	1	1	2	1	3.06
<20y	25-<30	118	97	2	1	1	2	2	2	0	1	1	1	1	1	2	1	3.55
25-<30	25-<30	111	96	2	1	1	3	2	2	2	1	1	0	1	1	3	1	3.68
20+yr	20+yr	95	81	2	2	1	2	2	3	2	1	1	1		1			2.6
25-<30	25-<30	115	98	2	1	1	3	3	3	0	1	1	2	1	1	2	1	3.655
25-<30	30-<35	92	92	1	1	2	3	2	3	0	1	1	1		2			3.67
<20y	<20y	81	85	1	1	1	2	2	2	1	1	1	1	1	1	2	1	3.12
20+yr	20+yr	103	97	2	2	2	2	3	1	0	1	1	1	0	1	2	1	3.36

25-<30	25-<30	103	96	2	1	2	2	2	1	0	2	1	1	1	1	2	1	2.92
25-<30	30-<35	103	110	1	1	1	2	2	1	0	1	1	1	1	1	3	0	3.61
<20y	25-<30	110	104	2	1	2	2	2	3	1	1	1	1	1	2	2	0	2.74
=35+	30-<35	100		1	2	2	1	2	1	2	1	2	2	0	1	2	1	3.35
<20y	<20y	107	98	2	2	1	2	2	1	0	1	1	2	0	1	2	0	3.42
<20y	<20y	105	92	1	2	2	1	2	2	1	1	1		1		3	0	2.81
30-<35	30-<35	90	87	2	1	1	2	2	1	0	2	1	1	1	2	2	1	3.89
<20y	25-<30	101	86	2	1	2	2	2	1	0	1	1	1	1	1			3.26
25-<30	<20y	96	98	2	1	1	1	1	1	2	1	1	1	0	2	2	1	3.13
<20y	25-<30	120	117	2	1	1	2	2	3	0	1	1	1		1			3.63
30-<35	25-<30	101		1	1	1	3	1	2	1	1	1	2		1		1	3.29
<20y	30-<35	96			1	2	2	2	2	0	1	1	1		1			3.8
=35+	=35+	95	100	2	2	1	2	1	1		1	1	2	0	1	3	1	3.04
25-<30	30-<35	120	103	2	2	2	3	2	2	0	1	1	2	1	1	3	0	3.12
20+yr	25-<30	98		2	2	2	2	2	3	1	2	1	1	0	1	3	1	3.33
<20y	25-<30	97	91	2	2	2	2	2	2	0	1	1	1	0	1	2	0	3.04
20+yr	<20y	90		2	2	2	2	2	1	0	1	1	1	1	1	2	1	3.12
20+yr	20+yr	85	112	1	2	1	3	1	1	1	1	1	2	1	1	2	1	3.9
30-<35	=35+	122	97	2	2	1	1		1	0	1	1	1	0	1		0	2.65
<20y	25-<30	92	90	2	1	1	2	2	3	0	1	1	1	1	1	2	0	2.5
<20y	<20y	89	91	1	2	1	2	3	1	0	1	1	2	1	1	2	0	2.98
30-<35	30-<35	112	97	2	2	1	2	3	3	2	2	1	0	1	2	2	1	2.44
25-<30	30-<35	95	91	2	1	2	2	2	3	0	1	1		1	1	3	0	3.69
25-<30	25-<30	103		1	1	2	3	1	2	0	1	1	1	1	2			3.78
<20y	25-<30	93	87		1	2	2	2	1	2	1	1	1	1	1	2	0	2.91
<20y	<20y	117	100	2	1	1	2	3	1	0	2	1	2	1	2	2	0	3.13
<20y	<20y	123	100	2	1	2	2	2	2	0	1	1	2	1	1		1	4.2
20+yr	<20y	99		2	1	1	1	2	1	2	1	1	2	1	2	2	1	2.95

25-<30	25-<30	93	100	1	1	1	2	1	2	0	1	1	0	1	2	2	1	1.88
25-<30	25-<30	97	93	2	1	2	2	2	3	0	1	1	0	1	1	2	0	2.36
25-<30	=35+	102		2	1	2	1	1	3	2	1	1	1	1	1	2	0	3.05
25-<30	30-<35	95	80	1	1	1	2	2	1	1	2	1	2	1	1	2	1	3.6
20+yr	<20y	98	84		2	1	2	3	3	1	2	2	0	1	1	2	1	3.85
<20y	<20y	115	85	2	1	1	2	3	3	1	2	1	2	1	1		0	3.9
<20y		101	89	1	2	1	2		1	0	1	1	1	1	1	2	1	4.46
20+yr	<20y	110	96	2	1	1	2	2	2	2	1	1	2	1	1	3	1	3.59
<20y	<20y	95		1	1	2	2	2				1	1	1	1	2	0	2.89
25-<30	<20y	110	103	1	2	1	1	2				2	0		1			3.38
<20y	25-<30	104	97	1	2	2	2	2	3	0	1	1	1		1			3.25
<20y	30-<35	106	106	2	1	2	3	3	3	0	1	1	2	1	1			3.6
<20y	25-<30	109	91	2	1	2	3	3	2	0	1	1	1	1	1	2	0	3.35
30-<35	25-<30	99	103	2	2	2	2	3	3	1	2	1	0	1	1	2	0	2.86
30-<35	25-<30	110		2	1	1	3	3	3	0	1	1	2	0	1	2	0	3.47
30-<35	30-<35	103	77		1	1	1	1	1	0	2	1	0			3	1	3.15
<20y		92	86	1	2	2	2	2	1	2	1	1	1	1	1	2	1	2.86
30-<35	25-<30	119	87	2	2	1	2	2	1	0	2	2	1	1	1	3	1	3.6
20+yr	<20y	106	101	2	2	1	2	2	1	1	1	1	0	1	1	2	0	3
20+yr	<20y	109	94	2	2	2	2	3	2	0	2	1	1	0	1	2	1	2.97
25-<30	25-<30	102	106	2	1	2	3	2	3	0	1	1	2	1	1	2	0	3.805
30-<35	30-<35	107	84	2	1	1	1	2	2	0	1	1	2	0	1			3.04
20+yr	<20y	107	101	2	2	2	2	2	2	2	1	1	2	1	1			3.85
20+yr	<20y	106	92	2	2	1	2	2	1	1	1	1	2	1	1	3	0	3.11
<20y	25-<30	112	95	2	1	2	2	2	2	0	1	1	2	1	1	2	0	3.26
25-<30	25-<30	112	108	2	1	1	3	3	2	0	1	1	2	1	2	2	1	3.13
20+yr	<20y	97			2	1	1	2		2	1	1	2	1	1	2	1	2.98
25-<30	25-<30	111	103	2	1	2	2	3	2	0	1	1	2	1	1	2	1	3.45

<20y	30-<35	102	103	2	2	1	2	2	3	0	1	1	2	1	1	2	1	3.45
<20y	30-<35	103	92	2	1	2	2	1	3	0	1	1	2	1	1	2	0	2.755
25-<30	25-<30	98	108	2	1	2	2	2	1	2	2	1	2	0	1			3.17
30-<35	30-<35	98	100	1	1	2	2	2	3	1	1	1	2	1	2	3	0	3.6
25-<30	25-<30	100	104	2	1	1	2	2	2	0	1	1	0	1		3	0	3.23
25-<30	30-<35	89	86	2	2	2	2	2	2	0	1	1	2	0	1		0	2.97
25-<30	25-<30	127	103	2	1	1	2	2	1	0	1	1	2	1	1	3	0	3.08
<20y	<20y	110		2	2	1	2	2	1	0	1	2	1	0	1	2	1	3.3
25-<30	30-<35	111	97	1	1	2	3	2	2	0	1	1	0	1	1	2	0	2.384
30-<35	=35+	94	98	2	2	2	1	2	2	0	1	1	2	1	1		0	2.96
30-<35	=35+	103	94	2	2	1	2	1	1	2	1	1	2	1	1	2	0	3.17
30-<35	30-<35	91	100	2	2	2	2	2	3	0	1	1	2	0	2			3.49
<20y	<20y	106	92	2	2	1	3	3	2	0	1	1	2	1	2	3	1	3.65
25-<30	30-<35	114	91	2	1	2	2	2	2	1	1		2	1	1	2	1	3.4
30-<35	=35+	108	117	2	2	2	3	2	3	0	1	1	2	1	1	2	1	3.82
=35+	=35+	103	96	2	2	1	1	1	3	0	1	1	1	1	1		0	3.44
=35+	=35+	117	98	2		1	2	2	2	0	1		0	1	1	2	0	3.95
<20y	25-<30	118	114		1	1	3	3	2	0	1	1	2	1	1	3	0	3.48
25-<30	25-<30	97	104	1	1	2	2	1	2	1	1	1			1			4
25-<30	30-<35	103	100	1	1	1	3	3	3	0	1	1	2	1	1	1	0	3.78
25-<30	30-<35	100	82			2	1	3	2	0	1		0		1			3.22
30-<35	=35+	80	87	2	1	1	2	1	2	2	1	1	1	0	1	2	1	3.68
<20y	<20y	110	117	2	1	2	2	3	2	0	1	1	2	1	1	2	0	3.65
30-<35	25-<30	122	99	1	2	2	3	3	3	0	1	1	2	1	1		1	3.83
<20y	25-<30	106		2	2	1	3	2	3	0	1	1	1	0	1	2	0	4.11
<20y		114	101	1	2	1	2		3	2	2	1	0		1	2	0	2.56
25-<30	30-<35	104	95	2	1	1	3	2	3	0	1	1	2	0	1	1	1	3.58
25-<30	30-<35	85	97	1	1	1	2	2	3	0	1	1	0	1	1	2	1	4.51

<20y	30-<35	103	97		2	2	2	2	3	2	2	1	2	1	1	3	0	3.82
<20y	<20y	104	104	2	2	2	2	2	3	0	1	1	1	1	1	2	0	3.53
<20y	<20y	117	110	2	1	2	3	3	3	0	1	1	1	1	1	3	0	3.24
<20y	25-<30	92	84	2	2	1	2	2	2	2	2	1	0	1	1	2	1	2.49
<20y	25-<30	118	88	1	2	1	2	2	3	0	1	1	1	1	1			3.52
<20y	<20y	97	87	2	2	2	1	2	3	0	2	1	1	0	1			3.41
20+yr		103	94	1	1	2	2		1	1	1	1	0	1	1	1	1	3.86
<20y	25-<30	110	97	1	2	1	2	2	2	0	1	1	1	0	1	2	0	3.4
25-<30	30-<35	112	93	2		1	3	3	3	0	1	1	2	1	1	2	0	2.55
20+yr	<20y	104		2	2	2	2	2	3	1	1	1	0	0	1	2	1	3.97
25-<30	25-<30	104	89	2		1			3	0	2		2	1	2			3.32
<20y		120	93	2	1	2	2	2		2	2	1	0	0	1		0	2.53
30-<35	=35+	98	98	2	2	2	2	2	2	0	1	1	0		1			3.75
25-<30		89	103	1	2	1	2	2	1	2	1		1	0	1	2	1	2.235
25-<30	25-<30	95	98	2	1	2	2	2	3	0	1	1	2	0	1		1	3.25
20+yr		99	92	1	2	2	2		1	0	1	1	0	1	1	2	1	2.74
20+yr	25-<30	103		2	1	2	2	3	3	1	1	1	1	1	1	2	1	2.64
20+yr	20+yr	94			2	2	2	2	1	2	1	1	0	1	1		1	2.81
<20y	<20y	98	112	2	1	1	2	2	2	2	2	1	0	1	2	2	1	3.81
<20y	25-<30	92	92	1	2	2	1	1	2	2	1	1	2		1			2.79
20+yr	<20y	110		2	2	2	3	3	1	0	1	1	2	1	1	2	0	3.22
20+yr		114		1	2	1	2		3	2	2	1	0	1	1	2	1	3.55
30-<35	25-<30	109	88	2	1	2	1	1	1	0	1	1	0	1	1	3	1	2.455
25-<30	25-<30	103	101	2	1	2	2	2	3	0	1	1	2	1	2	2	1	3.6
<20y	25-<30	100	93	2	1	1	2	2	3	1	2	1	1	1	1	2	0	3.5
25-<30		100	100	1	2	1	3		1	0	1	1	2	1	1	2	0	3.91
<20y	25-<30	105	97	2	1	1	2	3	3	0	1	1	1	1	1	2	1	3.17
<20y	<20y	101	86	2	2	2	2	2	3	0	1	1	2	1	1	2	0	4.12

25-<30		102	96	1	1	1	3	3	2	0	2	1	2	1	1	2	0	3.95
25-<30	30-<35	102	93	2	1	2	1	2	3	0	2	1	0		1			3.45
<20y	<20y	109	96	2	2	2	2	1	3	0	1	1	2	0	1	2	1	3.05
<20y	30-<35	120	110	2	1	1	2	2	2	2	1	1	2	1	1	2	0	3.11
<20y	<20y	107	104	2	2	1	2	2	2	0	1	1	0	1	1	3	0	3.39
<20y	25-<30	84	96	2	2	1	3	3	3	1	1	1	1	1	1	2	0	3.32
<20y	25-<30	103	87	1	2	1	3	3	1	0	1	1	1	1	1	2	0	4.03
<20y	<20y	80	91	1	1	2	1	1	2	2	2	1	0	1	1	2	1	2.43
30-<35	=35+	97	107	2	1	2	1	1	2	2	1	1	2	1	1	2	0	3.5
30-<35	=35+	117	107	2	2	1	2	2	3	2	2	1	1	1	2		0	3.45
25-<30	25-<30	93	87	1	2	1	2	2	1	0	2	1	0	1	1	2	1	3.65
=35+	=35+	94	88	1	2	1	1	2	1	0	1	1	2		1			3.31
25-<30	25-<30	120	108	2	1	1	3	3	3	2	1	1	1	0	1	3	0	2.95
25-<30	25-<30	103	92	2	1	2	2	2	3	0	2		2		1	2	0	3.24
25-<30	=35+	91		1		2	2	2	1	1	2	1	1	1	1	2	0	3.57
<20y	<20y	106	95	2	2	2	2	2	2	0	1	1	0	1	1	2	0	1.54
20+yr	<20y	106	104	2	2	2	2	3	3	0	1	1	1	1	1	2	0	4.04
25-<30	25-<30	99	104	2	1	2	2	2	2	0	1	1	2	1	1	2	1	3.52
<20y	<20y	94		1	1	1	3	2	3	2	2	1	1	1	1	2	0	2.75
<20y	25-<30	117	104	2	2	1	2	2	2	2	2	1	2	1	1	2	0	3.25
25-<30	25-<30	100	93	2	1	1	2	2	1	0	1	1	0	1	1	3	1	3.8
30-<35	25-<30	101	99	1	1	2	2	2	3	0	1	1	1	1	1	2	0	3.05
25-<30	25-<30	98	92	2	1	2	2	2		2	1	1	1	1	1			3.44
30-<35	30-<35	110		2	2	2	3	3	3	0	1	1	2	1	1			3.375
25-<30	25-<30	102	104	2	1	2	3	2	3	0	1	1	2	1	1	2	1	3.64
25-<30	25-<30	107	90	2	1	1	2	2		0	1	1	2	0	1			3.995
<20y	<20y	95	85	2	2	1	2	2		2	1	1	1	1	1	2		3.58
<20y	25-<30	110	92	2	1	2	3	3	3	2	2	1	1	1	1	2	0	2.65

<20y	<20y	107	114	1	2	2	2	2	3	1	1	1	1	1		2	0	3.72
<20y	<20y	118		1	2	2	1	2	2	0	1	1	1		1			2.985
30-<35	=35+	115	98	2	2	2	3	3	2	0	1	1	1	0	1	2	0	3.02
<20y	30-<35	97	95	1	2	2	3	2	3	2	1	1	2	0	1		0	3.17
25-<30	30-<35	109		2	1	2	2	2	2	0	2	1	2	1	1	3	0	3.51
<20y	<20y	99	104	2	1	2	2	2	3	2	1	1	1	0	1	2	1	3
30-<35	=35+	90	80	2	2	1	1	2	2	0	2	1	1		1			3.98
<20y	<20y	92	57	2	1	2	2	2	1	0	1	1	0		1			3.04
20+yr	30-<35	108	100	2	1	1	2	1	2	0	1	1	2	1	1	2	0	2.65
20+yr		101	99	1	2	2	2		1	0	1	1	1		1			2.27
25-<30	30-<35	92	89	2	1	2	3	2	3	0	1	1	1	0	2	1	0	3.12
25-<30	30-<35	109	110	2	1	1	3	1	3	0	1	1	2	1	2	2	1	3.27
=35+	=35+	85		2	2	2	2	2	3	0	1	1	1		1			4.01
<20y	<20y	99	85	1	2	2	2	2	1	0	2	1	0	1	1	2	1	3.9
30-<35	25-<30	97	87		2	2	1	2	2	0	2	1	1	0	1	2	1	1.495
<20y	25-<30	101	90	2	2	1	2	2	2	0	1	1	2	1	1	2	0	3.7
20+yr	<20y	100	101	2		2			2	2	2	2	0	1	1	2	0	3.32
<20y	25-<30	104	89	1	1	2	3	3	1	0	1	1	2	1	1	2	0	3.83
25-<30	30-<35	103	91	2	2	1	2	2	2	0	1	1	2	1	1	3	1	3.56
<20y	25-<30	89	94	2	2	1	1	1	3	0	1	1	0	0	1	2	1	2.74
<20y	25-<30	115	85	1	1	1	2	2	2	0	1	1	2	1	1	2	1	3.91
<20y	<20y	110		1	2	2	2	2	2	2	1	1	0	1	1	2	0	3.61
<20y	<20y	96	87	2	2	1	1	3	3	0	1	1	2	1	1	2	1	3.78
25-<30	25-<30	107	86	2	1	1	2	3	3	0	1	1	1	1	1	2	0	3.65
25-<30	25-<30	106	101	1	1	1	1	2	1	2	2	1	1		2			3.41
<20y	25-<30	102	94	1	2	2	2	1	2	0	1	1	2	1	1	2	0	2.83
30-<35	=35+	111		2	1	2	2	2	1	2	1	1	1	1	1	2	0	3.43
20+yr	<20y	85	88	1	2	2	2	2		1	1	1	1		1			3.37

<20y	<20y	107	101	2	2	2	3	1	2	0	2	1	2		1			3.88
<20y	=35+	105			1	2	3	3	3	0	1	1	2	1	1	2	0	3.7
20+yr	<20y	109	95	2	1	1	2	1	2	0	1	1	2	1	1	2	1	3.87
30-<35	25-<30	106	82	2	2	1	3	2	1	2	1	1	0	1	1	2	1	4.155
25-<30	25-<30	120	95	1		1	2	2	2	0	1	1	1	1	1		0	1.905
20+yr	20+yr	118	101	1	2	1	2	2	3	0	1	1	0	1	1	2	0	3.61
<20y	25-<30	117	110	2	1	2	1	2	3	2	1	1	2	1	1	2	0	3.38
25-<30	=35+	98	93	2	2	2	2	1	1	0	1	1	0	1	1	1	1	4.04
25-<30	30-<35	92	100		2	1	2	1	3	2	1	1	2	0	2	2	0	3.9
25-<30	30-<35	104	114	2	1	2	2	2	3	0	1	1	2	1	1	2	1	3.93
<20y		102	96	2	1	1	2	2	2	0	2	1	2	1	1	2	0	3.65
25-<30	25-<30	69	79	1	1	1	1	2	2	2	1	1	0	1	1	2	0	2.93
25-<30	25-<30	105	88	1	1	1	2	1	2	0	2	1	2	1	1	2	1	3.09
25-<30	25-<30	105	97	2	1	1	2	2	2	0	1	1	2	1	1			2.665
<20y	25-<30	98	91	2	2	2	2	2	3	2	2	1	1	1	1	2	1	3.15
25-<30	=35+	101	101	2	1	2	2	2	1	0	1	1	2	1	1	2	1	3.59
25-<30	25-<30	109	85	1	1	1	1	3	2	0	1	1	1	1	1	3	0	3.95
25-<30	=35+	94	101	1	1	2	3	2	2	0	1	1	2	1	2	2	0	3.7
20+yr	<20y	118	102	2	2	2	3	2	3	0	2	1	2	1	1			2.955
25-<30	30-<35	98		2	1	2	1	1	3	0	1	1	0	0	1	2	1	3.13
<20y	<20y	95		1	1	2	2	2	3	0	1	1	1		1			3.31
25-<30	25-<30	112	112	2	2	2	2	3	3	0	1	1	2	1	1	3	1	3.33
=35+	<20y	90	91	2	2	1	1	1	1	0	1	1	1	1	2	3	0	3.38
30-<35	=35+	97	98	1	1	2	2	2	1	2	1	1	0	1	2	2	1	3.62
25-<30	30-<35	105	93	1	1	1	2	2	2	0	1	1	1	1	1	2	0	3.45
=35+	=35+	102		2	1	1	1	3	1	0	1	1	1	1	1			3.36
<20y	25-<30	108	100	1		1	2	2	2	1	1	1	1	1	1	2	0	3.22
25-<30	30-<35	106	85	2	2	2	2	2	3	0	1	1	2	0	1	3	1	3.27

30-<35	25-<30	97		2	1	1	1	2	2	2	1	1	1		1			3.11
<20y	<20y	87	90	2	1	2	2	2	2	0	1	1	2	1	1	2	1	3.92
25-<30	30-<35	95	76	2	2	1	2	1	1	0	2	1	2		2			3.5
30-<35		105	109	2	1	1	3	3	3	0	1	1	2		1			3.18
30-<35	=35+	110	89	2	1	2	2	3	3	0	1	1	1	1	1	1	0	2.91
=35+	=35+	91	70	2	2	1	3	2	2	0	1	1	1	1	1	1	1	2
<20y	<20y	118	114	2	1	2	2	3	2	1	1	1	2	1	1	2	1	2.38
<20y		114	103	1	1	2	2	2	3	0	1	1	2	0	1			3.89
25-<30		112	90	1	2	1	2		2	2	2	1	1	1	1	2	0	3.72
<20y	<20y	103	103	1	2	2	3	2	1	0	1	1	2	1	1	2	0	4.375
<20y	25-<30	105	112	1	1	1	1	2	2	2	1	2	2	0	1	2	0	2.9
20+yr	<20y	92	85	2	2	2	2	3	1	1	2	1	2	1	1			3.46
20+yr	<20y	100	96	1	2	2	2	2	3	2	1	1	0		1		0	2.72
<20y		104	95		2	1	2		1	0	2	1	1	1	2	2	0	1.57
20+yr	<20y	107	109		1	1	2	3	2	2	2	1	2	1	1	2	0	4.09
25-<30	30-<35	112	92	2	1	2	2	3	3	0	1	1	2	1	1	2	1	3.23
30-<35	25-<30	94	84	2	1	1	1	1	1	0	1	1			1			3.45
<20y	30-<35	92	81	1		2	2	2	3	0	1	1						3.55
20+yr	<20y	79		2	1	1	3	2	2	2	2	1		1	1	2	0	3.49
<20y	<20y	99	102		2	1	2	1	3	0	1	1	2		1			2.7
<20y	<20y	122	93	2	1	2	2	2	1	2	1	1	1	1	1	2	1	3.32
30-<35	=35+	115	92	2	2	2	2	2		0	1	1	0		1			3.605
<20y	30-<35	104	90	1	1	1	3	3	3	0	1	1	2		1	2	0	3.56
25-<30	30-<35	120	88	2	1	1	2	1	2	0	1	1	0	1	2	2	1	3.19
25-<30	25-<30	88		2	1	2	1	1	1	0	1	1	0	0	1	2	0	2.51
20+yr	20+yr	95	97	2	2	1	1	2	2	2	1	1	0	1	1	2		3.15
25-<30	30-<35	89	88		1	1	1	1	2	2	2	1						3.855
<20y	25-<30	115	103	2	1	1	2	2	3	0	1	1	2		1	3	0	3.38

<20y	25-<30	86			2	2	2	2		2	2	1	2		1			3.66
20+yr	<20y	107	98	2	1	2	2	2	2	2	1	1	1	1	2	2	0	3.21
<20y	<20y	99	101	2	2	2	2	2	2	0	1	1		1		2	0	1.83
<20y	<20y	97	94	1	1	2	3	2	2	0	1	1	2	1	1	1	0	3.76
<20y	<20y	110	108	2	1	1			2	0	1	1	0	0	1	3	0	4.28
25-<30	25-<30	88		1	2	2	2	2	2	0	1	1	1		1			3.21
<20y	<20y	107	89	2	2	1	3	2	3	1	1	1	2	0	1	2	0	3.65
<20y	25-<30	102	88	2	1	1	1	1	1	0	1		1	1	2	2	1	3.97
<20y	<20y	93	86	2	2	2	1	2	1	1	1	1	0	1	1	2	1	1.71
25-<30	25-<30	105		1	2	1	3	3	2	1	1	1	2	1	1	2	0	3.575
25-<30	30-<35	94	96	2		1	1	1	2	0	2	1	1	1	1	2	1	3.12
<20y	<20y	86	84		1	1	1	2	3			1	0		1			3.26
30-<35	30-<35	108		2	1	1	1	1	2	0	1	1	1	0	1	2	1	3.94
25-<30	25-<30	111	88	2	1	2	1	2	1	1	2	1	1	0	1	2	0	3.58
=35+	30-<35	105	95	2	2	2	1	1		2	2	1	0	1	1		0	1.93
<20y	<20y	109	95	1	2	2	2	2	2	1	1	1	1	1	1	2	1	3.88
30-<35	25-<30	111	117	2	2	2	3	3	2	0	1	1	2	1	1	2	0	4.31
<20y	<20y	101	101	2	1	1	2	2	2	0	1	1	1	1	1	2	0	3.9
25-<30	30-<35	105	103	2	2	1	2	2	3	1	1	1	2	1	1	2	1	4.33
<20y	25-<30	106	87	2	1	2	1	1	2	0	1	1	1	1	1		0	3.18
25-<30		99		1	2	2	2	3	1	2	2	1	1	1	1	1	0	3.39
<20y	25-<30	90	113	2	1	2	2	2	2	2	1	1	1	1	1	2	1	4.065
<20y	<20y	102	96	1	2	1	3	3	3	0	1	1	1		1	2	0	3.06
<20y	25-<30	109		1	2	1	3	3	1	0	1	1	2	0	1	2	0	3.66
25-<30	25-<30	118	108	1	1	2	2	3	2	2	2	1	2	1	1	3	1	3.04
<20y	25-<30	101	91	2		1	3	3	3	1	1	1	2	0	1	3	0	2.62
30-<35	30-<35	94	91	1	2	1	1	2	3	0	1	1	1	1	1			3.06
<20y	<20y	103	101	1	2	2	1	1	1	2	2	1	1	0	1	2	0	3.03

<20y	<20y	79	86	2	1	2	2	2	2	0	1	1	0	1	1	2	1	3.71
25-<30	30-<35	103	106	2	1	2	2	1	3	0	1	1	2	0	1	3	1	3.78
30-<35	30-<35	97	106	1	2	2	3	3	3	0		1	1	0	1	3	0	3.9
<20y	25-<30	120	100	2	2	2	1	2	2	0	1	1	1	1	2	2	1	3.4
25-<30	=35+	105	104	2	1	2	3	3	3	0	1	1	1	1	1			2.27
25-<30	25-<30	104	81	2	1	2	2	2	1	1	1	1	1	0	1		0	3.53
30-<35	=35+	95	99	2	1	2	2	1	2	0	1	1	1	0	2	3	0	3.57
30-<35	30-<35	125	124	2	2	1	2	3	3	0	1	1	2	1	1			3.73
25-<30	25-<30	105	100	1	2	2	3	2	3	0	1	1	2	1	1	2	0	3.12
30-<35	30-<35	96	100	2	2	1	2	2	3	0	1	1	2	1	1	3	0	3.27
25-<30	25-<30	110		2	2	1	2	2	1	0	1	1	1		1			4.11
25-<30	25-<30	123	88	2	1	1	2	3	2	0	1	1	1		2			2.66
<20y	<20y	92	89	2	1	2	2	1	2	0	1	1	1		1			3.72
25-<30	25-<30	99	94		1	1	2	2	1	0	1	1	1	0	1	3	1	4.26
25-<30	30-<35	101	90	1	1	2	2	2	1	0	1	1	1	1	1			3.46
25-<30	25-<30	99	89	2	1	2	2	2	2	0	1	1	2	1	1	2	1	2.94
<20y	25-<30	108	103	1	2	2	2	2	1	0	1	1	2	1	1	2	0	3.14
<20y	25-<30	98	98	1	1	2	3	3	2	0	1	1	1	1	1	2	0	3.56
30-<35	30-<35	123	88	2	1	2	1	3	2	0	1	1	1	1	1	2	0	3.86
30-<35	30-<35	114	87	2	1	2	1	2	3	0	1	1	1	0	2	1	1	3.7
25-<30	=35+	85	83	2	1	2	2	2	3	0	1		1	0	1	2	0	3.755
30-<35	=35+	104	107	2	1	2	3	2	2	0	1	1	2	0	2	3	1	3.21
<20y	<20y	89	93	2	2	1	2	1	2	0	1	1	0	1	1	2	1	3.5
25-<30	25-<30	123	95	2	2	1	2	2	1	2	1	2	0	0	2	2	1	3.78
25-<30	30-<35	107	98	2		1	3	2	2	0	2	1	1	0	1	2	1	3.74
25-<30	25-<30	110	97	2	1	2	2	2	3	1	1	1	2	0	1	3	1	3.145
<20y	30-<35	112	101	2	1	1	2	3	3	0	1	1	1	1	1		0	3.61
25-<30	25-<30	111		2	2	2	3	3	3	0	1	1	2	0	1	2	0	3.06

25-<30	30-<35	82	96		2	1	3	3	3	0	1	1	1		1			3.61
20+yr	<20y	90		1	1	1	1	1	1	2	1	1	0		1			3.13
25-<30	25-<30	123	112	2	1	1	2	2	2	0	1	1	2	0	1	2	0	4
=35+	25-<30	88	97		1	1	2	2		0	1	1	0		1			2.62
25-<30	30-<35	103	94	2	2	2	1	2	3	2	1	1	1	0	1	3	0	3.265
25-<30	25-<30	108	108	2	1	1	3	3	1	0	2	1	2	1	1	2	0	3.45
=35+	30-<35	112	93		2	1	1	1	2	0	1	1	1	0	1	2	1	4.08
<20y	25-<30	104	98	1	1	2	2	2	3	0	1	1	1	0	1	2	0	3.91
30-<35	25-<30	114	112	2	1	1	3	3	3	0	1	1	2	0	1	2	1	4.2
<20y	25-<30	111	97	2	1	1	2	3	3	0	1	1	1	0	1	2	0	4
30-<35	30-<35	108	99	2	2	2	2	1	2	0	1	1	0	1	2	3	0	2.88
25-<30	25-<30	111	90	1	1	2	2	1	2	0	1	1	2	0	2	3	0	3.93
<20y	<20y	114	92	2	2	2	2	2		0	2	1	1		1			2.425
<20y	20+yr	107		1	2	2	2	2		0	2	1	0	1	1	2	0	3.11
<20y	<20y	102	94	1	2	1	2	2	1	2	2	1	0		1			3.18
<20y	=35+	110	97	2	1	2	3	3	3	0	1	1	2	0	1	3	0	4.02
25-<30	25-<30	114	98	2	2	1	2	2	3	0	1	1	1	1	1	2	0	4.68
<20y	<20y	99	88		2	1	2	2	3	0	1	1	1	1	1	2	0	3.91
<20y	25-<30	109	100	2	1	1	2	2	3	0	1	1	1	0	1	3	0	2.44
<20y	25-<30	99	98	2	2	2	2	1	2	0	1	1	1	0	2	2	1	3.33
<20y	25-<30	95	103	1	1	2	2	2	3	0	2	1	1	0	1	2	1	3.13
<20y	<20y	109	93	2	1	2	2	2	2	1	1	1	1	0	2	2	0	3.65
<20y	<20y	92	90	1	1	1	2	2	1	0	1	1	1	1	1	1		3.42
25-<30	30-<35	95	89	2	2	1	2	2	3	0	1	1	2	1	1	2	1	2.78
<20y	<20y	100	91	2	1	2	2	2	1	2	1	1	0	1	2	2	1	2.73
<20y	30-<35	88	85	2	1	1	1	2	3	0	2	1	2	1	2		0	3.28
30-<35	25-<30	104			1	2	2	2	2	0	1	1	1	1	1	3	0	4.03
25-<30	25-<30	115	119	2	1	2	2	3	3	0	1	1	2	1	1	3	0	3.45

25-<30	30-<35	97	84		2	2	3	2	1	1	1	1	1	1	1	1	0	2.99
20+yr		79	89	2	2	1	2	1	3	0	1	1	1	0	1	2	1	4.02
25-<30	30-<35	89	108	1	2	1	3	2	3	1	1	1	2	1	1	2	1	3.76
25-<30	25-<30	109		2	1	1	3	2	2	0	1	1	2	0	1	2	0	3.88
20+yr	<20y	86	97	2	2	2	1	1	1	2	1	1	1	0	2	2	0	3.9
=35+	30-<35	107	93	2	1	2	1	3	3	2	1	1	2	1		2	0	2.865
<20y	<20y	83	86	2		2	2	2	3	0	1	1	2	0	2	1	0	3.36
<20y	25-<30	97	95	2	1	2	2	2	2	2	2	1	0	0	1		0	2.88
20+yr	<20y	99			2	2	2	2	3	0	1	1	0		1			3.64
25-<30	30-<35	89	79		2	2	2	2	1	1	1	1	2	0	2	2	1	3.235
<20y	30-<35	118	103	2	2	1	2	2	3	0	1	1	1	1	2	2	1	2.87
<20y	<20y	94	88	2	1	1	2	2	1	2	1	1	1	0	1	2	0	2.9
=35+		115	79	2	2	2						1	2	0	1		0	2.92
25-<30	30-<35	115		2	1	2	2	2	3	0	1	1	1	1	1		0	2.98
30-<35	=35+	110	91	2	2	2	3	3	1	0	1	1	1	0	2	2	1	2.72
<20y	<20y	111	97	2	2	2	2	2	3	0	1	1	2	0	1	2	1	3.94
<20y	25-<30	103	109	2	2	1	1	2	3	0	1	1	0	0	1	2	0	4.02
25-<30	30-<35	103	112	1	2	2	2	3	3	0	1	1	2	1	1	2	1	3.67
=35+	30-<35	96	107	2	2	2	2	2	3	1	2	1	2	1	1	2	1	3.42
<20y	<20y	103	91		1	1	2	2	1	0	1	1	0		1			3.8
<20y	25-<30	85	82	2	1	2	3	1	3	0	1	1	1	0	1	2	0	3.8
25-<30	30-<35	115	100	2	2	2	3	3	3	0	1	1	2	1	1	3	0	3.6
20+yr	<20y	95	92	2	2	2	1	1	1	1	1	1	0	0	1	2	1	2.63
25-<30	25-<30	107	104	2	1	1	2	2	2	1	2	1	2	0	2	2	0	3.59
25-<30	30-<35	105	112	2	1	2	2	2	3	2	2	1	0	0	1	2	0	3.085
<20y	<20y	103	107	2	2	2	2	2	2	0	1	1	1	0	1			2.58
25-<30	25-<30	100	90	2	1	1	2	1	3	0	1	1	0	0	1	2	1	3.98
<20y	25-<30	108	85	2	1	1	2	2	3	1	1	1	0	0	1	2	0	3.225

25-<30	30-<35	107	112	2	2	2	3	1	3	1	1	1	2	0	1	2	0	3.48
30-<35	30-<35	106			1	2	2	2		0	1	1	2		1			3.18
<20y	<20y	112	103	2	2	2	2	2	3	0	1	1	2		1			3.515
<20y	<20y	92	93	2	1	1	2	2	2	2	1	1	0	0	2	2	1	3.745
25-<30		114	110	2	2	2	2	2	2	0	1	1	0	0	1			3.3
20+yr	<20y	90		2	2	1	2	2		0	1	1	1	1	2	2	1	2.96
<20y	<20y	127	119	2	2	1	3	3	3	0	1	1	1	0	1	2	1	3.92
30-<35	30-<35	108	89	2	1	2	2	2	2	1	2	1	1	1	2	3	1	3.39
20+yr	20+yr	92	85	1	2	1	2	2	1	0	1	1	1		1			3.61
<20y	<20y	73	93	2	1	1	1	1	2	0	1	1	0	0	1	2	1	3.585
<20y	25-<30	102	94	2	2	1	2	2	2	0	1	1	2	0	1	2	0	3.82
25-<30	30-<35	102	96	1	1	2	2	1	2	1	1	1		0		2	0	3.21
25-<30	30-<35	123		2	1	1	2	2	3	0	1	1	0	0	1	2	0	3.74
<20y	<20y	103	104	2	1	2	2	1	2	0	1	1	2	0	1	2	0	3.43
25-<30	25-<30	99	95	2	1	2	3	2	3	0	1	1	1	0	1	3	0	3.64
<20y	30-<35	103		1	2	1	2	2	3	2	1	1	1	0	1	3	0	3.29
<20y	25-<30	93	80	2	2	1	2	2	2	1	2	1	1	1	1	2	1	3.48
<20y	<20y	115	95		2	1	2	2	2	0	1	1	2	1	1	2	0	3.795
25-<30	25-<30	115	97	2	1	1	3	3	2	0	1	1	2	1	1	2	0	3.09
<20y	=35+	103	88	1	1	2	3	1	3	1	2	1	2	0	1		1	3.5
<20y	30-<35	94	87	1	1	1	2	3	2	0	1	1	1	0	1	2	1	3.92
<20y	30-<35	108	94	2	2	1	2	1	3	0	1	1	1	0	1	2	0	4.42
20+yr		90	105	1	2	1	2		1	1	1	1	2	0	1		0	3.77
25-<30	25-<30	96	93	2	1	1	2	2	2	0	1	1	2	0	1	2	1	4.42
25-<30	25-<30	103	86	2	1	1	2	2	2	0	2	1	1	0	2	2	0	3.56
25-<30	25-<30	103	110	2	1	1	2	3	3	1	1	1	1	1	1	2	1	3.47
30-<35	30-<35	111	93	2	1	2	1	3	2	0	1	1	2	0	1	2	0	3.29
25-<30	25-<30	97	97	2	1	1	2	3		1	1	1	1	0	1	3	0	3.5

25-<30	30-<35	105	95	2	1	1	3	3	2	1	1	1	2	0	1	3	0	2.93
20+yr	20+yr	100	101	1	1	1	1	2	1	2	1	1	1	1	1	3	0	3.7
25-<30	25-<30	101		1	1	2	2	2	1	1	1	1	2	0	1	3	0	3.15
20+yr	20+yr	94	87	2	2	2	1	2	1	1	1	1	1	0	2	2	0	3.41
<20y	25-<30	92	90	2	1	2	2	2	2	0	2	1	1		1			3.37
<20y	25-<30	94	96	2	1	1	2	1	3	2	1	1	1	0	1	3	1	3.53
<20y	<20y	94	103	2	1	1	2	3	1	1	1	1	2	0	1	2	1	4
=35+	=35+	118	117	1	1	1	3	3	1	0	1	1	2	1	1			3.49
25-<30	25-<30	90	110	2	2	1	2	2	2	0	2	1	1	0	2	2	1	3.59
20+yr	<20y	103	128	2	2	1	3	2	2	0	1	1	0	0	1	2	1	2.42
25-<30	25-<30	112	94	2	1	1	2	2	3	0	1	1	2	0	2	1	0	3.71
<20y	25-<30	108	91	2	1	1	2	2	3			1	0	0	2	2	1	4.19
<20y	<20y	105		1	2	2	2	2	3	0	1	1	2	0	1	2	0	3.76
25-<30	25-<30	106	100	2		1	2	2	3	1	2	1	1	0	1	2	0	3.13
<20y	<20y	102	85	2	1	2	2	2	3	0	1	1	1		1			3.34
25-<30	30-<35	82	84	2	1	1	1	1	2	0	1	1	0	0	1			3.22
25-<30	25-<30	115	106	2	1	1	3	3	2	0	1	1	1	0	1		1	3.61
<20y	<20y	89	100	1	1	2	2	2	2		1	1	1	0	1	3	0	3
20+yr	<20y	94	105	1	2	1	2	2	2	1	1	1	2	0	1	2	0	3.71
25-<30	30-<35	88	104		2	2	2	3	2	2	1	1	2	0	2	2	1	2.67
25-<30	25-<30	106	100	2	1	1	2	2	2		1		2	0	2	2	1	4.1
25-<30	30-<35	86	87	2	1	2	1	2	2	0	2	1	0		1			3.52
<20y	25-<30	90	93	2	1	2	1	2	3	0	2	1	2	0	1	2	1	3.62
25-<30		92	96	1	2	1	1		1	0	1	1	2		1			3.4
25-<30	30-<35	123	112	1	1	1	3	2	3	0	1	1	2	1	1	2	0	2.89
25-<30	25-<30	92	98	2	2	1	2	2	3	1	1	1	2	0	1	2	1	3.76
25-<30	<20y	92	94	2	2	2	2	2		0	2	1	1	0	1	2	0	3.3
<20y		103	86	1	2	1	2			2	2	1	1	0	1	2	0	3.26

25-<30	30-<35	122	98	2	1	2	3	3	2	0	1	1	2	0	1	2	1	3.31
25-<30	30-<35	93	87	2	1	2	2	2	2	0	1	1	2		1			3.045
30-<35	<20y	86	84	2	1	1	1	1	2	2	2	1	2	1	1		0	3.21
20+yr		86	92	1	2	2	2		3	0	1	1	0	0	1	1		2.98
25-<30	30-<35	114	89	2	2	1	1		2	2	1	1	0		1	2	0	1.81
<20y	25-<30	111	112	1	1	1	3	1	1	0	1	1	2	0	1	2	1	3.24
25-<30	30-<35	107	96	2	1	1	2	2	2	2	2	1	1	0	2	2	1	3.58
30-<35	=35+	106	101	2	2	1	2	1	3	2	1	1	2		1			3.35
25-<30	25-<30	106	103	2	1	2	2	2	3	2	1	1	2	0	1		0	3.11
30-<35	=35+	97			1	1	2	2	2	1	2	1	0	0	1	2	1	3
25-<30	30-<35	102	90	2	1	1	2	1	2	0	1	1	2	0	1	3	1	2.645
<20y	<20y	115	98	2	1	2	3	3	2	0	1	1	2	1	1			3.38
25-<30	30-<35	109	104	2	1	1	2	2	2	2	1	1	1	0	1	2	0	2.16
25-<30	25-<30	110	114	2	1	1	3	3	3	0	1	1	2	0	1	3	1	3.09
25-<30	25-<30	115	95	2	1	1	1	2	3	0	1	1	2	0	1			3.76
<20y	<20y	120	119		2	1	3	2	2	0	1	1	2		1			2.88
20+yr	25-<30	80	102	2	2	2	2	2	1	1	1	1		0	1	3	1	3.045
<20y	<20y	86	101	2	1	2	1	1	2	2	1	1	0	0	1	2	0	2.89
25-<30	=35+	105			1	2	3	1	3	0	1	1	2		2			3.76
<20y		100	93		2	1	2	2	2	2	1	1	1	0	1	3	0	3.72
<20y	25-<30	92	86	2	2	2	2	3	3	0	1	1	2	0	1	2	0	3.25
25-<30	25-<30	100	106	2	1	2	2	3	3	0	1	1	1		1	2	1	3.51
=35+	30-<35	97	102	1	1	2	2	2	3	0	1	1	2	0	1	3	1	3.29
<20y	<20y	84	84	1	1	2	2	1	2	2	1	1	1	0	1	2	1	2.84
25-<30	30-<35	114		1	2	2	3	2	3	0	1	1	0	0	2	1	0	3.48
25-<30	30-<35	110	83	2	1	1	1	3	3	0	1	1	0		1			3.39
30-<35	=35+	110	114	2	2	1	3	3	3	1	2	1	2	1	1	2	0	3.11
25-<30	25-<30	107	122	1	1	1	2	2	2	2	2	1	1		1			3.45

25-<30	25-<30	105		1	2	1	1	2	2	0	1	1	2	0	1	1		3.16
25-<30	25-<30	93		1	2	2	2	2	1	2	1	1	0	0	1	2	0	3.25
<20y	=35+	110	117	1	2	2	3	2	3	0	1	1	2		1			4.21
30-<35	=35+	88		1	2	2	2	1	2	1	1	1	1	0	1	1		2.68
25-<30	25-<30	132	117	1	2	2	3	3	2	0	1	2	2	0	2	2	0	3.66
<20y	<20y	83	95	2	1	1	2	2	2	1	1	1	2	0	1	2	0	3.66
=35+	=35+	110	61	2	1	2	1	2	1	0	1	1	0	1	1	3	1	3.53
=35+	=35+	109	89	2	2	1	2	2	3	0	1	1	2	0	1	2	1	3.17
20+yr	<20y	109	105	1		1	3	2	2	2	1	1	1	0	1	2	0	2.61
=35+	=35+	91		1		1	2	2	3	0	1	1	0	0	1	2	0	4.03
<20y	<20y	118	102	1	1	1	2	2	3	0	1	1	2	0	1	2	0	3.925
25-<30	=35+	109	106	2	1	2	3	2	1	2	1	1	1	0	1	2	0	2.8
<20y	25-<30	90	92	2	1	1	2	2	2	0	1	1	0	0	1	2	0	3.72
=35+	=35+	103	112	2	2	2	2	3	2	2	2	1	1	0	1	2	0	2.98
<20y	25-<30	82	82	2	1	2	2	2	3	0	2	1	2	0	1			2.86
<20y	<20y	86	90	2	2	1	2	2	2	2	2	1	0	0	1	2	1	3.49
30-<35	=35+	109			1	2	3	1	1	0	2	1	1	0	1	2	1	3.04
25-<30	25-<30	111	112	2	2	1	3	3	3	0	1	1	2	0	1	3	0	3.05
<20y	25-<30	105		1	1	1	2	3	1	0	1	1	0		1			3.25
<20y	25-<30	117	101	2	1	2	3	2	2	0	1	1	2	0	1		0	4.41
=35+	=35+	94		1	2	1	3	3	3	0	1	1	0	0	1	2	1	3.47
=35+	30-<35	111	117	2	1	1	2	2	3	0	1	1	2	0	1	3	1	3.033
<20y	25-<30	101	98	1	1	1	2	2	3	0	1	1	2	0	1	2	0	3.425
25-<30	25-<30	98	94	2	1	2	2	2	1	0	2	1	2	0	2	2	1	2.42
=35+	=35+	94	102	2	1	1	2	2	3	0	1	1	1	0	1	3	0	3.595
25-<30	25-<30	92	96	2	2	1	2	2	1	0	1	1	0	0	2	2	1	2.42
=35+	=35+	108	120	2	1	1	3	3	2	1	2	1	2	0	1	3	1	3.67
25-<30	25-<30	81	77	2	2	1	1	2	1	0	1	1	1	0	1	2	1	4.25

<20y	25-<30	103	104	1	2	1	2	2	1	0	1	1	0	0	2	3	0	4.21
25-<30	25-<30	104	117	2	2	1	2	2	2	0	1	1	1	0	1	2	0	3.74
<20y	25-<30	115	103	2	2	1	3	2	2	2	1	2	2	0	1	2	0	4.485
30-<35	30-<35	104	103	2	1	2	2	3	3			1	2	0	1	3	0	3.61
25-<30	30-<35	97	93	2	1	1	2	1	2	2	1	1	1		1			3.89
25-<30	25-<30	110			1	1	1	3	3	0	1	1	0		1			3.06
30-<35	30-<35	89	90	2	1	2	2	2	2	1	1	1	2	0	1	3	1	3.21
<20y	<20y	111	85	2	2	1	2	2	1	2	1	1	1	0	1	2	1	3.75
25-<30	30-<35	103	108	1	2	2	2	2	1	0	1	1	1		1			3.62
<20y	<20y	110	87	2	1	1	2	2	2	0	1	1	1	0	1			3.28
20+yr		100	99	1	2	1	2	2	2	2	1	1	1		2			3.5
30-<35	30-<35	109	97	2	1	2	2	2	3	1	1	1	2	0	1	2	1	3.495
<20y	30-<35	88	84	2	1	1	2	2	3	0	1	1	2	0	1	2	1	4.65
<20y		114	101	1	2	2	3	2	1	1	1	1	2	1	1	2	0	3.245
25-<30	25-<30	120		2	1	1	3	2	2	0	1	1	2	0	1	2	0	3.2
25-<30	25-<30	123	112	2	2	1	2	2	3	0	1	1	2	0	2	2	0	3.37
25-<30	30-<35	102	93	2	1	2	2	2	3	1	1	1	1	0	1	2	1	3.105
30-<35	25-<30	109	93	1	1	1	2	1	2	0	1	1	2	0	1	2	0	3.52
25-<30	30-<35	114	97	2	2	2	2	3	3	0	1	1	2	0	2	2	0	3
=35+	=35+	123		1	1	1	1	1	2	0	1	1	2	0	1	2	1	3.905
25-<30	30-<35	101	96	2	2	1	2	2	2	0	1	1	2	0	2	2	0	4.23
30-<35	25-<30	94	86		1	2	2	1	3	0	2	1	0	0	1		0	3.35
<20y	25-<30	105	99	1	2	2	3	3	3	0	1	1	2	0	1	2	0	3.29
20+yr		88	95		2	2	1		3	0	1	2	0	0	1	2	0	2.09
=35+	=35+	112	78		2	1	2	2	1	0	1	1	2	0	2	2	1	3.545
20+yr	<20y	108	90	2	2	1	2	2	1	2	1	1	1	0	1	2	0	3.67
<20y	25-<30	111	98	1	2	1	2	2	2	0	1	1	1	0	2			2.825
<20y	25-<30	96	91	2	2	1	2	1	2	1	1	1	1		1			3.84

25-<30	25-<30	95	82	2		1	3		3	0	1	1	1	0	1	2	1	2.99
<20y	25-<30	93	96	1	1	1	2	1	2	0	1	1	0					3.4
25-<30	25-<30	81	87	2	1	2	2	2	2	2	2	1	0	0	1	2	1	3.18
<20y	25-<30	108	94	2	1	1	2	3	2	0	1	1	2	0	1	2	1	4.23
<20y	25-<30	102	92	2	1	2	2	3	3	0	1	1	2		1			3.92
<20y	25-<30	101	101	1	1	1	2	1	3	0	1	1	2	0	1	2	1	3.63
25-<30	30-<35	110	125	2		1	2	2	3	0	1	1	2	0	1	2	0	3.47
25-<30	25-<30	118		2	1	2	2	2		0	1	1	0	0	1	2	1	3.26
<20y	<20y	117	98	1	1	1	2	2	3	0	2	1	2	0	1	2	0	3.66
30-<35	30-<35	118	96	1	1	2	2	2	1	0	1	1	2	1	1	3	0	3.81
30-<35	30-<35	115	98	2	2	1	3	3	2	0	1	1	2	0	2	2	0	3.28
<20y		89	76		2	2	3	1		1	1	2	1	0	1		1	3.18
20+yr	<20y	102	102	1	2	1	2	1	1	0	2	1	1	0	1	2	0	3.21
25-<30		110	91	2	2	2	3	3	3	0	2	1	2	0	1	3	0	3.46
<20y	25-<30	85			1	2	2	2	1	1	1	1	2		1			3.565
30-<35	30-<35	120	125	2	1	2	3	3	3	0	1	1	2	0	1	2	1	4.325
<20y	<20y	97	100	2	2	1	1	2	2	1	1	1	0		1			3.19
<20y	25-<30	97	95	1	1	2	2	1	1	0	1	1	2	0	1	3	0	3.06
<20y	25-<30	95		1	2	2	2	2	3	0	1	1	2	0	1	2	1	2.82
25-<30	30-<35	110	91	2	1	2	2	3	3	0	1	1	2	0	1	2	1	3.495
20+yr	20+yr	95	87	1	2	1	2	2		2	2	1	0	0	1	2	1	3.53
<20y	<20y	90	90	2	1	2	2	2	3	0	2	1	2	0	1	2	1	3.21
<20y	25-<30	114	100	2	1	2	2	2	1	0	1	1	2	0	2	2	0	3.56
25-<30	25-<30	101	94	2	2	1	2	2	2	0	1	1	2	0	1		0	2.81
25-<30	30-<35	112	92	2	2	1	2	2	2	0	1	1	1	0	1	3	1	3.39
20+yr	<20y	103	117		2	1	2	2	1	0	1	1	1	0	1	2	0	4.4
25-<30	25-<30	94	90	2	1	2	2	2	2	0	1	1	2	0	1	2	0	3.57
<20y	25-<30	93	94	2	2	1	2	3	2	0	1	1	2	0	1	2	0	3.34

30-<35	30-<35	118		1	1	2	3	3	3	0	1	1	2	0	1	3	0	3.64
30-<35	=35+	102	79	2		1	2	2	3	2	1	1	0	0	2	1	0	2.69
30-<35	30-<35	108	103	2	1	1	2	2	3	0	2	1	1	0	1	2	0	4.17
<20y	30-<35	90	80	2	2	1	2		3	2	1	2	0		2			3.44
<20y	<20y	115	100	2	1	2	2	1	3	2	1	1	2	0	1	2	1	4.01
20+yr	<20y	98			1	2	1	2	1	1	2	1	2	0	1	2	0	3.46
<20y		109	97	1	2	1	2		2	1	1	1	0	0	1	2	1	3.27
25-<30	30-<35	100	96	2	2	1	2	2	3	2	1	1	1	0	1	3	0	3.31
30-<35	30-<35	106	100	1	1	2	2	3	1	0	1	1	2	0	1	3	0	3.85
25-<30	25-<30	105	97	1	1	2	2	2	2	0	2	1	1	0	1	2	0	3.79
30-<35	30-<35	111	107	2		1	3	2	3	0	1	1	1	0	1	3	0	3.61
20+yr	20+yr	83	98	1	2	1	2	2	1	1	1	1	1	0	1		0	3.15
<20y	<20y	107	81	2	2	1	2	1	3	0	1	1	1	0	1	2	1	3.58
25-<30	30-<35	112	100	2	1	1	2	2	3	0	2	1	1	0	1	1	0	3.44
<20y	<20y	106	101	2	1	1	2	2	2	2	1	1		0		2	0	1.886
25-<30	25-<30	106	91	2	1	2	2	1	3	2	1	1	1	1	1	2	0	3.75
=35+	25-<30	90	87	1	1	2	1	2	3	0	1	1	0	0	1	2	0	4.23
30-<35	30-<35	100	84	2	1	2	3	3		2	1	1	0	1	1	2	1	3.49
<20y	<20y	104	88	2	2	2	2	3	3	1	1	1	1	0	1	3	1	3.97
25-<30	25-<30	118	110	2	1	2	3	3	2	0	2	1	2	0	2	2	1	3.08
25-<30	25-<30	88	77	2	2	2	2	2	2	1	2	1	2	0	1		1	3.935
25-<30	25-<30	109	81	1	2	1	2	2	2	2	1	1	2	0	1	2	1	3.298
30-<35	30-<35	102	112	2	1	2	2	2	3	2	2	1	2	0	1		0	2.48
25-<30	30-<35	97	88	2	1	1	2	2	2	0	1	1	2	0	2			4.01
25-<30	30-<35	96	96	2		1	3	3	2	2	1	2	0	1	1	2	1	2.93
20+yr	<20y	102	87	1	1	2	2	1	1	0	2	1	2		1			3.775
30-<35	30-<35	89	93	2	2	2	3	2	1	0	1	1	2	0	1	2	0	2.415
30-<35	=35+	109	88	2	1	1	1	1	3	0	1	1	1	0	1	1	0	3.8

<20y	25-<30	99	98	2	1	1	2	2	3	0	2	1	2	0	2	2	0	3.8
25-<30	30-<35	111	94	2	2	1	2	2	3	0	1	1	1	0	1			3.33
30-<35	30-<35	120	105	2	2	1	3	3	3	0	1	1	2	1	1	2	1	4.415
<20y	<20y	97	101	2	1	2	3	2	3	0	1	1	1		1			2.53
25-<30	=35+	104	101	2	1	1	2	3	1	2	1	1	1	0	1	2	1	3.33
<20y	<20y	114	110	2	2	2	2	2	1	2	1	1	1	1	1	2	0	3.37
20+yr	<20y	97	92	1	2	2	2	2	2	0	1	1	2	1	1	2		3.26
<20y	<20y	99	97	1	2	2	2	2	3	1	1	1	1	0	1	1	0	3.25
<20y	25-<30	103	98	2	1	1	2	2	3	0	1	1	1		1			4.1
30-<35	=35+	114	97	2	1	2	1	2	2	2	1		2	0	1	2	0	2.79
25-<30	30-<35	102	98	2	1	1	2	1	3	0	1	1	2	0	1	2	1	3.3
30-<35	25-<30	97	107	2	2	2	2	2	3	2	2	1	0	0	1	2	1	3.85
30-<35	30-<35	105	93	2	2	2	2	2	2	0	1	2	1	0	2	2	0	3.54
<20y	<20y	89	88		2	2	1	2	1	2	1	1	0		1			3.15
<20y	25-<30	99	103	2	1	1	2	2	3	0	1	1	2	0	2	2	1	4.59
25-<30	30-<35	106	93	2	2	2	3	2	2	0	2	1	2	0	2	2	1	3.13
25-<30	30-<35	94	97	2	2	2	2	2	3	2	2	1	1	0	1	2	1	3.01
30-<35	25-<30	118	117	2	2	1	3	3	2	1	1	1	2	0	1	3	0	2.69
30-<35	30-<35	90		1	1	1	2	2	2	0	2	1	2	0	1	2	1	3.72
<20y	<20y	92	87	2	1	2	2	2	2	0	1	1	0		2			2.96
30-<35	30-<35	111	117	2	1	1	2	2		0	1	1	2	0	1	2	1	3.74
<20y	=35+	109		1	2	1	2	3	2	0	1	1	0	1	1	2	0	3.53
<20y	25-<30	99	89	2	2	2	2	2	3	0	1	1	1	1	1	2	0	4
25-<30	=35+	111	95	1	2	1	1	2	1	0	1	1	1	0	1	2	1	3.9
25-<30	25-<30	79	80	1	2	1	2	3	1	0	1	1	1	1	1	1	1	4.36
<20y	<20y	114	97	1	1	2	2	2	2	0	1	1	2	0	2	2	0	3.7
<20y	25-<30	118		2	1	2	2	2	1	2	1	1	2	0	1	2	0	3.27
<20y	<20y	101	113	2	2	2	2	1	3	1	1	1	1	0	1	2	0	4.06

20+yr	<20y	99	94		2	2	2	2	1	0	1	1	0	0	2	2	0	3.3
<20y	25-<30	112	93	2	1	1	2	2	2	0	1	1	2	0	1	2	1	3.45
<20y	<20y	108	108	2	1	1	1	2	3	0	1	1	1	0	1	2	0	3.9
<20y	<20y	125	90	2	2	1	2	2	1	2	1	1		0		2	1	2.88
<20y	25-<30	98	111	2	1	2	2	2	2	1	1	2		0		2	0	3.01
<20y	25-<30	110	106	2	2	2	2	2	2	2	2	1	0	0	1	3	1	3.77
25-<30	25-<30	100	108	1	1	1	3	3	3	0	1	1	1	1	1	2	0	3.96
25-<30	25-<30	112	100	2	1	2	2	2	2	0	1	1	2	0	1	2	1	3.47
30-<35	30-<35	101	95	1	1	2	1	2	2	2	2	1	0	1	1		0	4.57
30-<35	30-<35	108	88	1	1	2	2	2	1	0	1	1	2	0	1		1	3.06
30-<35	30-<35	102	90	1	1	1	2	2	2	0	1	1	2	0	1	3	0	5.39
<20y	<20y	88	94	2	2	1	3	3	1	0	1	1	1	0	1	2	0	3.56
<20y	<20y	101		2	2	2	2	2	3	0	1	1	1	0	1	2		3.49
30-<35	30-<35	90	109	2	2	2	2	2	1	2	2	1	2	1	1	2	1	2.7
<20y	<20y	101		2	2	1	2	2	3	0	2	1	1		2			3.16
<20y	<20y	108	92	1	1	2	3	2	3	1	1	1	1		1			3.69
25-<30	30-<35	117	91	1	2	1	2	2	1	2	1	1	1	1	1	2	1	3.615
25-<30	=35+	115	100	2	1	1	3	3	3	0	1	1	2	0	1	2	0	4.29
25-<30	25-<30	108	114	2	2	2	2	2	3	2	1	1	2	0	1	2	0	2.66
30-<35	25-<30	105	104	2	1	2	2	2	2	1	1	1	2	1	1	2	0	3.19
20+yr	<20y	101	92	1	2	1	2	2	3	0	1	1	2	0	1	2	0	3.63
25-<30	30-<35	112	103	2	2	1	2	3	3	0	1	1	1	0	1	2	0	1.495
25-<30		97			1	1	2					1	0		1	2	0	3.9
25-<30	30-<35	104	68	2	1	2	3	2	2	0	1	1	1	1	1	3	1	3.245
20+yr		100	102	1	2	1	2		1	0	1	1	1	0	1	2	0	3.27
25-<30	=35+	112	110	2	1	1	1	1	2	0	1	1	2	1	1	3	1	3.78
<20y	<20y	110	98	1	1	1	2	3	2	0	1	1	2	1	1	2	0	4.13
20+yr	<20y	112	95	1	2	2	2	3	1	2	1	1						3.61

<20y	=35+	101	89	2	1	1	1	1	2	2	1	1	1	0	1		1	3.1
20+yr	<20y	102	93	1	1	1	3	2	2	2	2	1	1	0	1	2	0	3.37
<20y	<20y	99			1	2	2	2	1	0	1	1	1	1	1	2	1	3.03
25-<30	25-<30	102	104	1		2	2	2	3	0	1	1	1	0	1	2	0	3.51
25-<30	30-<35	109	108	2		2	2	1	1	0	1	1	1	0	1	1	0	2.45
20+yr	<20y	120	98	2	2	1	2	3	2	0	1	1	2	1	1		0	3.74
25-<30	25-<30	101	92	2	1	2	2	3	3	1	1	1	1	1	1	3	1	2.63
<20y	<20y	88	88	2	1	1	2	1	1	0	1	1	2	0	1	2	0	3.46
<20y	25-<30	101	96	1	1	2	2	1	3	0	1	1	1	0	1	2	0	2.99
<20y	25-<30	125		2	1	2	3	2	3	0	1	1	2	0	1	3	0	3.715
<20y	25-<30	105	82	2	1	2	3	2	2	0	1	1	2	0	1		1	3.9
<20y	25-<30	98	93	1	2	1	1	2	1	2	1	2	1	0	1	2	0	2.85
25-<30	30-<35	94		2	1	1	2	2	2	0	1	1	1	1	1			4.19
25-<30	30-<35	102	49	2	2	1	3	2	1	0	1	1	0	0	2	3	1	2.98
=35+	=35+	104	93	1	1	2	1	2	3	0	1	1	0	0	1	3	0	3.5
=35+	=35+	120	120	2	2	2	3	3	3	1	1	1	2	1	1	2	0	3.98
20+yr	<20y	120	105	1	2	2	1	2	3	1		1	0	0	1	3	0	3.715
20+yr	25-<30	97		1	1	1	2	2	2	2	1	1	1		1			3.92
30-<35	25-<30	117	117	2	1	1	2	2	3	2	1	1	1	0	1	2	0	2.99
25-<30	30-<35	97	86	2	1	1	2	2	3	0	2	1	2	0	1	2	0	3.6
20+yr	<20y	112	99	2	2	2	2	2		0	1	1	1	1	1	2	0	3.21
30-<35	25-<30	110		1	2	1	3	2	2	0	1	1	2	0	1	3	0	3.15
30-<35	25-<30	137	98	1	1	2	3	2	3	0	1	1	2	1	1	3	0	4.4
20+yr	25-<30	96	82	2	2	2	2	1	3	0	1	1	1		1			3.53
<20y	<20y	107	119	2	1	2	3	1	1	1	1	1	1		1			3.29
20+yr	<20y	115	98	2	2	2	1	2	1	1	1	1						3.47
=35+	=35+	115	97	2	1	1	2	1	2	0	1	1		0		3	1	2.97
<20y	25-<30	115			2	1	3	2	2	2	1	1	1	0	1	2	0	3.26

<20y	25-<30	103	106	2	2	1	2	2	3	0	2	1	1	0	1	1	0	3.67
25-<30	30-<35	102	87	2	1	2	2	2	2	2	2	1	0	0	1		0	2.93
20+yr	25-<30	105	93	2	1	1	2	2	2	1	2	1		1		2	0	3.73
25-<30	<20y	93	97	2	2	2	3	3	1	2	1	1	2	0	1	2	1	3.04
<20y	<20y	109	104	2	1	2	2	3	2	0	1	1	1	0	1			2.37
<20y	30-<35	92	88	2	2	2	2	2	3	2	1	1	2	0	2		0	2.98
30-<35	25-<30	97	107	1	2	2	3	2	2	0	1	2	2	1	1	2	0	3.32
25-<30	30-<35	100	86	2	1	1	1	2	2	1	2	1	1	0	1		0	3.78
25-<30	<20y	98	103	2	1	2	3	3	2	0	2	1	2	0	1	2	1	3.58
30-<35	<20y	123	107	2	2	2	3	2	2	0	1	1	2		1			3.18
<20y	25-<30	112	101	2		1	3	2	2	2	1	1	2	0	1	2	0	3.52
25-<30	20+yr	97	80	2	1	2	2	1	1	2	2	1	0		1			3.69
<20y	<20y	95	87	1	1	2	2	2	2	0	1	1	1	1	1		0	3.78
20+yr	20+yr	110	117	1	1	1	2	2	1	1	1	1	0		1			2.96
25-<30	25-<30	111	112	1	1	2	3	3	3	0	1	1	2		1			2.81
<20y	25-<30	95	101	1	2	1	2	2	2	1	1	1	1	0	1	2	1	4.12
25-<30	25-<30	112	100	2	1	1	2	2	3	0	2	1	0	0	1	2	1	3.41
25-<30		106	108	2	2	1	2	2	2	0	1	1	2	0	1	1	0	3.69
<20y	<20y	108	97	2	2	1	2	2	3	2	1	1	2		1			3.26
=35+	=35+	107		2		1	2	1	2	0	1		0	0	1	1	0	2.16
30-<35	30-<35	108	110	2	1	2	2	3	3	0	1	1	2	0	1	2	1	3.06
30-<35	30-<35	96	95	2	2	2	2	2	3	0	1	1	1	0	1	2	1	3.465
<20y	<20y	107	102	1	2	1	3	2	1	1	1	1	2	1	1	1	0	3.29
30-<35	30-<35	94	104	2	1	1	2	2	2	0	1	1	2	0	2	3	1	4.51
20+yr	<20y	106	101	2	2	1	2	2	2	0	1	1	1		1			3.65
25-<30	=35+	112		1	1	1	2	3	1	0	1	2	0	0	1	2	0	3.39
20+yr	=35+	106	93	2	2	1	1	2	1	0	1	1	1	0	1	2	0	2.71
25-<30	30-<35	132		1	1	1	2	3	3	0	2	1	2		1			3.21

<20y	25-<30	105	93	2	2	2	3	1	3	1	1	1	0	0	1	2	0	3.25
20+yr	<20y	114	95	2	2	2	2	3		0	1	1	1	0	1	3	0	3.8
<20y	25-<30	110	100	2	1	1	2	2		0	1	1	2	0	1	2	0	4.22
<20y	<20y	105	100	1	2	2	2	2	2	2	1	1	2	1	1	3	1	3.41
25-<30	25-<30	90	89	2	1	2	2	2	3	0	1	1	2	1	1	2	0	3.57
30-<35	30-<35	96	93	2	1	1	2	2	3	2	1	1	0	0	1	2	1	3.32
20+yr	<20y	109		1	2	1	1	2	1	2	1	2	2	0	1	2	0	4.14
<20y	<20y	100	99	1	1	1	3	2	3	0	1	1	1	0	1	1	1	3.58
25-<30	25-<30	104	100	2	1	1	2	2	2	0	1	1	2	0	1	2	0	3.5
30-<35	30-<35	107		2	2	2			2	0	1	1	2		2			3.47
25-<30	30-<35	100	103	1	2	1	2	2	2	0	1	1	2	0	1		0	2.98
25-<30	30-<35	111		2	1	1	3	2	2	0	2	1	2	0	2	2	0	3.38
<20y	<20y	100	102	2	1	1	1	2	2	1	1	1	1	0	1	2	0	2.58
<20y	<20y	95	82	1	2	1	2	2		1	1	1	0	0	2	2	1	3.76
<20y	25-<30	92	97	1	2	1	2	2	2	0	1	1				1	0	3.96
<20y	<20y	114	100	2	1	2	2	2	3	2	1	1	1	1	1	3	1	3.43
20+yr	<20y	101	85	2	1	1	2	2		2	1	2	0	0	1	2	0	3.355
25-<30	30-<35	95	80	2	2	2				2	1	2						3.725
<20y	25-<30	109	101	2	2	1	2	2	2	1	1	1	2	1	1	3	0	3.33
=35+	=35+	122	128	2	1	1	3	3	3	0	1	1	2	0	1	2	0	3.6
<20y	<20y	91	99	1	1	2	2	1	1	2	1	1	1	0	1	2	0	2.91
25-<30	30-<35	110	112	2	1	2	3	3	3	0	1	1	2	0	1	2	1	3.22
25-<30	30-<35	104	103	1	1	1	3	2	1	1	1	1	1	0	1	2	0	3.61
<20y	<20y	115	122	2	2	2	3	3	3	2	1	1	2	0	2	2	0	3.83
25-<30	30-<35	109	103	1	1	1	2	2	3	0	2	1	2		1			3.49
=35+	=35+	118	83	2	2	2	2	2	2	1	1	1	1		1			3.27
25-<30	25-<30	114	95	2	1	2	2	2	1	1	1	1	0	1	2	2	0	1.81
20+yr	<20y	102	102	2	2	1	2	2	2	0	2	1	0	0	2	3	0	3.36

30-<35	=35+	98	128	2	1	1	2	3	3	0	1	1	1	0	1	2	1	3.78
<20y	25-<30	112	115	2	2	1	2	3	2	0	1	1	2	1	1	2	0	3.31
<20y	<20y	106		2	1	2	2	2	2	0	1	1	2	0	1	2	1	3.13
25-<30	<20y	102	114	2	1	1	2	2	1	0	1	1	2	0	1	3	1	3.48
<20y	<20y	110	93	1	2	1	3	2	3	2	1	1	1	0	1	2	0	3.31
30-<35	30-<35	102	93	1	1	2	2	2	2	0	1	1	0	0	2		0	3.43
30-<35	=35+	98	104	2	1	2	2	2	2	0	1	1	2	0	1	2	0	2.225
25-<30	25-<30	99	97	2	1	2	2	2	3	1	1	1	1		2			3.63
<20y		100	119	1	2	1	2		1	0	1	1	1	0	1	2	0	4.6
20+yr		120	87	2	2	1	1		3	2	2	1	1	0	1		1	3.13
30-<35	=35+	115	77	2	1	1	2	3	3	0	1	1	1	0	1	1	0	3.62
25-<30	=35+	108	100	2	1	2	2	2	2	2	1	1	1	0	2	2	0	2.9
25-<30	30-<35	100	87	1	1	1	1	2	2	2	2	2	1	0	1	2	1	3.9
25-<30	25-<30	103	101	2	1	1	3	2		1	1	2	1	0	1	1	0	3.01
25-<30	30-<35	98	93	2	1	1	2	2	2	0	1	1	2	0	1	2	0	3.68
25-<30	<20y	98	87	1	2	2	2	1	3	0	1	1	0	0	1		0	2.74
<20y	25-<30	114	101	2	1	1	2	2	3	2	2	1	1		1			3.95
25-<30	30-<35	120	112	2	2	1	2	2	3	2	2	1	0	0	1	3	0	3.34
25-<30	25-<30	105	96	2	2	2	3	2	2	0	1	1	1	0	1	2	0	2.92
25-<30	25-<30	100		1	1	2	2	2	2	1	1	1	2	0	1	2	0	4.06
<20y	<20y	95		1	1	2	2	2		0	2	1	1	0	1	2	1	3.32
<20y	<20y	110	95	2	1	1	2	2	3	0	1	1	2	0	1	2	0	3.46
<20y	25-<30	120	100	1	1	1	2	1	1	0	1	1	2		1			2.186
25-<30	30-<35	96	87	2	1	2	2	3	3	0	1	1	1	0	1		0	3.135
30-<35	=35+	99	77	2	2	2	2	3	2	2	1	2	0	1	2	2	1	3.13
<20y	<20y	93	101	2	1	2	2	2	3	1	1	1	2	0	1		1	3.63
25-<30	25-<30	100	93	2	1	1	2	2		0	1	1	2	0	1	1	0	3.68
<20y	<20y	108		1	2	2	1	2	1	2	2	1		0		2	1	3.35

<20y	<20y	120	100	1	2	1	2	1	1	1	1	1	1	0	1	2	0	3.71
20+yr		96		1	2	2	2	1	1	2	1	1	0	1	1	2	0	2.95
<20y	25-<30	103	101	2		1	2	2	3	1	1	1	2		2			3.57
<20y	25-<30	106	103	2	2	1	2	2	3	0	1	1	2	0	1	2	1	4.15
25-<30	25-<30	102	85	2		1	1	1	1	2	1	1	2	0	1	3	0	3.46
20+yr	<20y	91	104	2	1	2	2	2	1	0	1	1	1	0	1	2	0	3.425
<20y	25-<30	102	91	2	1	2	1	2	3	0	1	1	1	0	1	2	0	2.92
=35+	=35+	94	94	1	1	2	1	1	3	2	1	1	1	1	1	2	0	3.13
25-<30	25-<30	111	98	1	2	1	2	2	1	0	1	1	1		1			3.78
<20y	30-<35	99	96	2	1	2	2	1	3	0	1	1	1	0	1	3	0	3.42
<20y	25-<30	93	88	2	1	2	1	2	1	0	2	1	1	0	2	1	1	3.39
30-<35	=35+	102	91			1	2	2	3	1	2	1	2	0	1	2	1	3.16
20+yr	20+yr	100		2	1	1	2	2	1	0	1	1	2		1			3.83
<20y	<20y	107	105	1	2	2	3	2	3	2	1	1	2	1	1		0	3.49
<20y	25-<30	103	106	2	1	1	2	2	3	0	1	1	2	0	2	2	1	3.125
<20y	<20y	75		2	1	2	2	2	2	1	2	1	1	0	2	2	1	2.75
30-<35	30-<35	110	98	2	1	1	2	2	1	0	1	1	0	0	1			3.33
20+yr	25-<30	120	100	2	1	1	2	3	2	0	1	1	2	1	1	2	0	4.29
30-<35	30-<35	107		2	1	2	2	2	3	0	1	1	1	0	1	2	1	3.08
=35+	=35+	109	92	1	2	1	2	2		1	1	1	1	1	1	2	1	3.42
30-<35	=35+	83	95	2	1	1	1	1		0	1	1	2	0	1	2	1	3.5
<20y	25-<30	101	84	1	1	2	1	2	3	0	1	1	1	0	1	2	1	2.9
25-<30	30-<35	110	106	2	2	2	3	3	3	0	1	1	2	0	1	2	1	3.7
<20y	<20y	97	89	2	2	2	2	2	3	0	1	1		0		2	1	2.09
<20y	<20y	114	106	1	1	2	3	3	2	0	1	1	2	0	1	2	0	3.38
20+yr	<20y	110	90	1	2	2	2	2	3	0	1	1	1		1			3.14
25-<30	30-<35	107	114	2	1	2	3	2	3	2	1	1	2	0	1	3	1	2.3
25-<30	30-<35	110	110	2	2	1	2	2	3	0	1	1	2	0	1	2	1	3.99

20+yr	<20y	106	104	2	1	1	2	2	2	0	2	1	2	0	1			3.96
<20y	25-<30	104	96	2	1	1	1	2	2	0	1	1	2	0	2	2	0	3.495
30-<35	=35+	92	85	1	1	1	1	2	1			1	2	0	1	2	1	3.77
20+yr	<20y	106	97	2	2	1	2	2	2	2	1	1	1	0	1	2	0	2.1
25-<30	30-<35	97	90	2	2	1	2	2		1	1	1			1			1.014
25-<30	30-<35	110		2	1	1	2	3	1	0	1	1	2	0	1	1	0	3.79
25-<30	25-<30	94	114	2	1	2	2	2	1	0	1	1	1	0	1	2	1	2.86
<20y	25-<30	118	95	2	1	1	1	3	2	2	1	1	2	0	1	2	0	3.275
25-<30	25-<30	104	92	2	1	1	3	3	3	0	1	1	2	0	1	2	1	3.795
25-<30	=35+	129	95	2	1	1	2	2	2	0	1	1	2	0	1		1	3.42
30-<35	30-<35	109	102	2	2	1	2	1	3	0	1	1	2	0	1	3	0	3.25
<20y	25-<30	90	92	1	1	2	3	2	1	0	1	1	1	1	2	1		3.03
25-<30	25-<30	92		2	1	2	1	1	2	2	1	1	2	1	1	2	1	4.08
25-<30	<20y	93	93	2	1	1	2	2	2	0	1	1	2	0	2	2	0	3.57
=35+	=35+	114	87	2	1	1	2	3	2	1	1	2	0	0	1	3	0	4.03
20+yr		108		1	2	1	1			1	1	1	1	0	2	2	1	3.61
20+yr	<20y	115	96	2	2	1	2	2		0	1	1	0	0	1	2	0	2.81
25-<30	25-<30	99	80	2	2	1	2	2	3	1	2	1	1	1	1	2	1	3.18
25-<30	=35+	123	96	2	1	1	2	2	2	0	2	1	1	0	1	2	1	3.57
=35+	=35+	100	113	2	2	2	1	1	2	0	1	1	1	1	1	3	0	3.04
20+yr	<20y	118	102	1	2	1	2	1		0	1	1	1		2			4.28
25-<30	30-<35	95	108	1	2	1	2	2	2	2	1	1	2	0	1	3	0	3.025
<20y	25-<30	109	86	2	2	1	2	2	2	0	1	1	2	0	2		1	4.26
25-<30	25-<30	106	90	2	1	2	2	2	2	0	1	1	2	0	1	2	1	3.08
20+yr		106	102	1	2	1	1		1	2	2	1	1	0	2	2		3.07
<20y	25-<30	102	114	2	2	1	2	2	3	0	1	1	2	0	1	2	1	4.23
<20y	=35+	99	97	2	2	2	2	2	3	0	2	1	2		1			3.59
=35+		92	79	1	2	2	1	2	3	2	2	1	0	0	1	2	1	2.78

=35+	=35+	122	88	1	1	1	3	1	1	0	1	1	2		1		1	3.43
25-<30	<20y	115	93	2	1	1	1	2	2	0	1	1	2	0	1		0	3.4
25-<30	30-<35	107	117	1	1	2	3	3	3	0	1	1	2	0	1	3	0	2.77
<20y	25-<30	101	91	2	1	2	2	2	1	0	1	1	1	0	1	2	1	4.005
20+yr	<20y	112	97	2	2	2	2	2	1	2	1	1	1	0	1		1	2.84
<20y	<20y	109	101	1	1	2	2	2	3	0	1	1	1	0	1	1	0	4.55
30-<35	30-<35	92			2	1	1	1	2	0	1	1	0	1	1			3.03
<20y	<20y	110	81	2	2	2	2	2	2	2	1	1	0		1			2.77
25-<30	=35+	84	96	1	1	1	2	1	2	0	1	1	2	0	1	2	0	4.01
25-<30	30-<35	97	103	1	2	1	2	2	2	0	1	1	1	0	1	2	0	3.925
20+yr	<20y	104		1	2	2	2	2	2	1	1	1	1	0	1	2	1	3.73
30-<35	=35+	125	128	2	1	1	2	3	3	0	1	1	2	0	1	2	0	3.72
20+yr	<20y	95	88	2	2	1	3	2	3	0	1	1	2	0	1	2	0	3.28
<20y	25-<30	109	108	2	1	2	3	2	3	0	1	1	1	0	1	2	0	3.07
=35+	=35+	99	103	2	2	2	2	1	2	0	1	1	2	0	1	2	0	3.92
20+yr		86	90	1	2	2	2		3	0	1	1	0	0	1	2	1	3.74
<20y	25-<30	91	98	2	2	2	2	2	2	0	1	1	1	0	1	2	0	3.5
25-<30	30-<35	109	112	2	2	1	3	2	2	0	1	1	1	0	1	2	1	3.31
25-<30	=35+	100		2	2	1	2	2	2	0	1	1	2	0	2	2	1	3.41
30-<35	30-<35	118	103	2	1	2	3	2	2	0	1	1	2	0	2	3	0	3.17
25-<30	25-<30	99		2	1	2	2	3	3	2	2	1	0	1	1	2	0	3.19
<20y	25-<30	112	101	2	1	1	3	3	3	0	1	1	2	1	1	2	0	3.16
<20y	<20y	98	93	2	1	1			3	0	1	1	0	1	1	1	0	3.83
25-<30	30-<35	104	94	2	1	2	3	3	2	0	1	1	2	0	1	3	0	3.36
<20y		99		1	2	2	2		3	2	1	1	1	0	1	2	0	3.55
=35+	25-<30	95	90	1	2	2	2	2	1	1	2	1	0	0	1	1	1	3.76
30-<35	30-<35	100	100		1	2	2	2	2	2	2	1	0	0	1	2	0	3.91
25-<30	25-<30	110		2	1	2	2	2	3	0	2	1	2	0	1	3	0	3.83

<20y	25-<30	104		1	1	2	2	3	2	1	1	1	2	1	1	1	1	3.7
30-<35		115	97	2	2	1	2	3	1	2	2	1	2	0	1	3	0	3.555
<20y	25-<30	96	92	2	2	2	2	3	2	1	2	1	2	0	1	2	0	3.05
20+yr		107		1	1	1	3		2	0	1	1	1	1	1	2	1	2.94
<20y	25-<30	111	104	1	2	2	2	1	1	0	1	1	2		1			2.66
20+yr	20+yr	114	93	2	2	2	2	2	1	1	1	1	1		1			3.47
<20y	25-<30	104	106	2	2	2	2	2	2	0	1	1	2	0	1	2	0	3.415
25-<30	25-<30	111	108	2	1	1	1	3	3	2	1	1	1		1			3.25
<20y	<20y	94	85	2	2	1	2	1	3	1	2	1	2		1	2	1	3.08
25-<30	30-<35	101	112		2	1	2	2	2	2	1	1	0	0	1	2	0	3.67
<20y	25-<30	100	110	2	2	1	2	2	3	0	1	1	1	1	1	2	1	3.5
<20y	<20y	96		2	2	2	3	3	2	0	1	1	1	1	1	2	1	3.29
<20y	25-<30	111	98	2	2	2	2	2	3	0	1	1	2	0	1	2	0	3.8
20+yr	30-<35	109		1	2	2	1	3		2	1	2		1		2	1	2.89
25-<30	30-<35	110	89	2	1	2	3	3	2	1	2	1	2	0	2	2	0	3.7
=35+	=35+	94	98	1	2	2	2	2	2	0	1	1	2	0	2	2	1	3.84
<20y	<20y	99	97	2	2	2	1	2	1	2	1	1	1	0	1	2		3.74
<20y	25-<30	95		2	1	2	2	2	3	0	1	1	2	0	1	2	0	3.45
<20y	<20y	108	103	1	2	2	2	2	2	0	1	1	2	0	1	3	0	3.24
25-<30	30-<35	111		2	2	1	2	1	3	2	1	1	1	0	1	2	0	2.99
<20y	<20y	118		2	1	2	2	2	2	1	1	1	1	0	1	2	0	2.29
<20y		91	79	2	2	2	1		1	1	1	1	0	1	1	1	1	2.64
<20y	<20y	92	110	1	2	1	2	2	1	0	1	1	2	0	1	2	0	3.29
=35+	=35+	96	81	1	2	2	2	2	2	0	1	1		0	2	1	1	3.23
=35+	30-<35	122	100	1	2	1	2	3	3	0	1	1	2	0	1	2	1	4.125
25-<30	30-<35	100		1	2	2	3	3	1	0	1	1	2		1			3.475
25-<30	25-<30	101	106	1	2	2	3	2	3	1	1	1	2	0	1	2	1	3.01
=35+	30-<35	111	84	2	2	1	1	2	1	2	1	1	2	0	1	2	1	3.7

<20y	30-<35	97			2	2	2	3	2	1	1	1	2	0	1	2	0	2.87
20+yr	<20y	115	92	1	2	2	2			2	1	1	1	1	1	2	0	2.795
20+yr	25-<30	90		1	1	1	2	2	2	2	2	1	0	0	1	2	0	3.42
25-<30	30-<35	107	101	2	1	2	2	2	3	0	1	1	1	0	1	3	0	3.36
25-<30	<20y	85	88	1	1	1	1	2	2	1	1	1	2	1	2	2	1	2.355
25-<30	25-<30	111	101	2	2	2	2	3	3	0	1	1	2	0	1	1	0	3.67
25-<30	25-<30	103	98	2	1	1	2	2		1		1	1	1	1	2	0	3.48
25-<30	=35+	122	81	1	2	2	2	2	1	0	1	1	2	1	1		0	3.67
20+yr	<20y	99	93	2	2	2	3	2	1	0	2	1	2	0	1	2	0	3.87
25-<30	25-<30	103	90	2	1	1	1	2	3	0	1	1	2	0	1	2	1	3.42
25-<30	30-<35	115	104	2	1	2	2	3	2	2	1	1	1	0	1	2	0	3.085
<20y	25-<30	111	97	2	2	2	2	2	1		2	1	2	0	1	2	0	3.71
30-<35	=35+	99	103	2	2	2	2	2	3	0	1	1	2	0	1	2	0	2.78
30-<35	30-<35	118		2	1	2	3	2	3	0	1	1	2	0	1	3	0	2.55
<20y	25-<30	97		2	1	1	1	1	1	0	1	1	1	0	1	1	0	3.1
=35+	=35+	97	97	1	2	1	3	2	1	2	1	1	1	0	1	2	0	2.63
<20y	25-<30	84	96	1		1	2	2	2	0	1	1	2	0	1	2	0	3.75
<20y	25-<30	110	93	1	2	1	2	3	1	0	2	1	2	0	2	2	0	3.98
25-<30	25-<30	112	86	1	1	1	2	2		0	2	1	1	0	1	2	0	3.885
25-<30	25-<30	100	97	2		1	2	2	3	0	1	1	0	1	2	2	0	3.13
25-<30	25-<30	122	104	2	1	2	2	3	3	2	1	1	1	0	1	2	0	3.53
20+yr	<20y	97	90	1	2	2	2	2	1	2	1	1	1		1			2.93
25-<30	25-<30	90	95	2	2	1	1	2	2	2	1	1	1		1			2.552
30-<35	25-<30	104	103	2	1	2	3	3	2	0	1	1	2	1	1	1		3.64
<20y	25-<30	111	104	2	2	1	2	1	3	0	2	2		1			0	3.98
<20y	<20y	111	88		1	1	2	2	2	0	1	1	2		1			3.84
<20y	=35+	100	92	2	2	2	1	2	2	2	2	1	2	0	1	1	1	2.87
<20y	30-<35	104		2	1	2	3	1	1	1	2	1	0	1	1	2	0	4.03

<20y	<20y	99	88	1	1	1	2	2	2	0	1	1	2	0	1	2	0	4.23
25-<30	30-<35	109	101	1	1	2	2	2	2	0	1	1	1	0	1		0	3.76
=35+	=35+	115	81	1	2	2	1	2	2	0	1	1	2	1	1	2	1	2.965
25-<30	25-<30	97	101	1	2	1	2	2	2	0	1	1	2	0	1	2	0	3.51
20+yr	<20y	99	86	1	2	2	1	2	2	1	1	1	2	1	1	3	1	3.79
=35+	=35+	109	105	2	2	1	2	3	2	0	1	1	2	1	1	3	0	3.6
25-<30	30-<35	112	122	1	2	1	3	2	1	0	1	1	2	0	1	2	0	3.56
25-<30	25-<30	107	100		1	2	3	3	2	0	1	1	2	1	1	2	1	3.91
25-<30	25-<30	116	98	2	2	1	2	3	3	0	1	1	1	0	1	2	1	3.665
25-<30	25-<30	106	85	2	1	2	2	2	2	1	1	1	1	0	1	3	0	3.52
<20y	25-<30	108		1	1	2	2	2	2	2	2	1		0		2	1	3.53
<20y	25-<30	83	86	2	1	2	2	2	3	0	1	1	2	0	1	2	1	3.6
25-<30		110	125	2	1	1	2	3	2	0	1	1	2	1	1	2	0	3.89
20+yr	<20y	93	102	1	2	2	2	1	1	0	2	1	0	0	1	2		3.15
25-<30	<20y	95	97	2	1	1	2	2	1	1	1	1	1	0	1	2	1	3
25-<30	25-<30	88	82	2	1	1	1	3	2	0	1		1	0	1	2	1	3.26
30-<35	30-<35	104	114	2	2	1	3	2	3	2	2	2	2		1			3.26
30-<35	30-<35	95	101	2	1	2	1	2	3	2	2	1	0	1	1	3	0	4.34
<20y	<20y	90	87	2	2	1	1	2		0	2	1	1	0	1	2	1	3.59
30-<35	30-<35	103	93	2	1	2	2	1	1	0	2	1	2	0	1			2
20+yr	<20y	86	91	2	1	2	2	2	2	0	1	1	0		1			3.57
<20y	<20y	102	91	2	1	1	2	2	3	0	1	1	0	1	1	1	0	3.54
<20y	30-<35	110	83	2	1	1	2	1	3	0	1	1	0		2			4.35
25-<30	30-<35	97	98	2	1	2	2	2	2	2	2	1	2	0	1	2	0	3.41
<20y	25-<30	112	112	2	1	2	2	2	3	2	1	1	2	0	1	2	1	3.18
20+yr		112	98	1	2	1	2		1	2	1	1	2		1			2.54
20+yr	<20y	83	89	2	2	1	1	2	1	2	1	1	0	0	1	2	1	4.02
25-<30	=35+	97		1	1	1	3	2		0	1	1	2	1	1	2	1	3.73

<20y	<20y	114	100	2	2	2	2	2	2	0	1	1	2	0	1	2	0	3.31
<20y	30-<35	108	101	1	2	1	2	3	1	0	1	1	2	1	1	3	1	2.91
25-<30	25-<30	107	95	2	1	2	2	2	3	0	1	1	2	1	1	3	0	3.565
30-<35	=35+	111	104	2	1	2	3	2	3	0	1	1	2	0	1	3	1	2.64
30-<35	30-<35	115	99	1	2	2	2	2	3	0	1	1	2	0	1	3	0	3.49
<20y	<20y	107	110	2	2	2	2	3	2	0	2	1	2	0	1	2	0	3.68
<20y	=35+	110		1	1	1	3	1	2	0	1	1	2	0	1	2	1	3.56
<20y	25-<30	92	80	2	1	1	2	2	2	0	1	1	2	0	1	2	1	3.43
=35+	=35+	104		1	1	2	1	1	2	2	2	1	1		2			2.96
25-<30	=35+	99			2	2	1	1	3	0	1	1	1	0	1	3	1	3.69
25-<30	25-<30	94	100	2	2	2	2	2	2	0	1	1	2	0	1	2	0	3.67
25-<30	30-<35	114	96	1	1	2	3	3	1	0	1	1	2	0	1	2	0	3.65
25-<30	30-<35	98	94	2	2	2	2	2	3	0	1	1	2	0	1	2	0	3.45
25-<30	25-<30	115	95	1		1	2	1	2	0	1	1	2	0	1	2	1	3.64
25-<30	30-<35	99		2	1	2	2	1	2	1	1	1	0	1	1	3	0	3.95
20+yr	20+yr	86	92	1	2	1	3	1	1	0	1	1	1	1	1	2	1	4.31
<20y	<20y	113		1	2	2	2	3	2	0	1	1	2	0	1	2	1	3.54
25-<30	30-<35	94	103	2	2	2	3	3		0	2	1	2	0	1		1	3.3
20+yr		99	95	1	2	1	2	2	2	0	1	2	0	0	1	2	0	3.28
<20y	25-<30	101	108	1	1	2	2	2	2	0	1	1	1		1			2.96
<20y	<20y	117	111	2	1	1	3	3	1	0	1	1	2	1	1	2	0	3.135
20+yr	<20y	106	105	1	1	2	1	1	1	0	1	1	0	1	1	2	1	3.85
<20y	25-<30	87	80		1	1	2	2	1	2	2	1	0		2			2.52
<20y	25-<30	93	103	2	2	2	2	2	2	1	2	1	2	1	1	2	1	2.82
25-<30	30-<35	111	81	2	2	2	3	3	3	0	1	1	1	0	2	2	1	3.65
<20y	25-<30	88	90	2	1	1	2	2	2	1	2	1	1	0	1	2	0	2.216
<20y	25-<30	111	112	2	1	1	2	2	2	0	1	1	2	0	1	2	0	3.84
20+yr	<20y	88	86	2	1	2	2	2	1	0	1	1	0	0	2	2	1	3.02

<20y	30-<35	95	103	2	2	2	3	3	2	0	1	1	1	0	1	2	0	3.145
<20y	25-<30	106	85	2	2	2	2	3	2	0	1	1		0	1	3	1	1.695
<20y	25-<30	107		1	2	2	2	2	2	0	1	1	0	1	1	1	1	2.62
<20y	<20y	108		1	2	1	2	2		0	1	1	1	1	1	2	0	3.54
<20y	25-<30	97	103	1	1	1	3	2	2	0	1	1	1	1	1	2	1	4.27
<20y	25-<30	97	89	1	1	2	2	2	2	0	1	1	2	0	1	2	0	3.08
<20y	25-<30	88	86		1	2	1	2	2	0	1	1	1	0	1	2	1	4.09
<20y	25-<30	101	94	2	2	1	2	2	1	2	1	1	1	0	1	2	0	3.66
<20y	30-<35	104	92	1	1	2	2	2	2	0	1	1	1	1	2	3	0	2.61
<20y	25-<30	105	99	1	2	1	2	2	2	2	1	1	1	1	1	3	1	3.73
<20y	<20y	99	88	2	2	1	2	2	2	2	1	2	2	1	1	2	0	3.65
25-<30	25-<30	98	104	2	1	1	2	1	3	0	1	1	0	0	1	2	1	3.17
25-<30	25-<30	110	88	2	1	2	2	2	3	0	2	1	0	0	2	2	1	2.94
<20y	25-<30	127	108	1	1	1	2	2	2	1	1		2		1			3.35
30-<35	=35+	112	112	2	2	1	3	3	3	0	1	1	2	0	2	2	0	2.58
30-<35	<20y	105		2	2	1	3	2	3	0	1	1	2	0	1	3	1	4.32
<20y	25-<30	104	98		2	1	2	2	3	0	1	1	2	0	1	2	1	3.23
<20y	<20y	92	88	2	2	1	2	2	1	0	1	1	1	1	1	2	0	4.08
<20y	25-<30	108	108	1	2	1	2	2	1	2	1	1	1	0	1	2	1	3.08
<20y	<20y	107		2	1	1	3	2	2	0	1	1	1	0	1	2	0	3.49
25-<30	30-<35	107	107	2	2	1	2	2	2	0	1	1	1	0	1	2	1	3.69
=35+	=35+	88	95	2	1	1	2	2	2	0	1	1	2	1	1	1		3.7
25-<30	30-<35	98	112	2	1	1	2	2	3	0	1	1	1	0	2		1	3.8
25-<30	30-<35	108	87	2	1	2	2	2	2	0	1	1	2	1	2	2	1	3.27
<20y		102	87	2	2	2	2	2		2	2	1	1	0	1			3.6
<20y	<20y	92		1		2	3	1	2	1	1	1	0	0	1	2	0	2.68
<20y	<20y	106	88	2	1	2	2	3	1	1	1	1	1	0	1	1	0	4.08
25-<30	30-<35	120	133	2	2	1	2	3	2	0	1	1	1	1	2	2	1	3.45

30-<35	=35+	104	107	2	1	1	2	2		0	1	1	2	1	2	3	1	4.03
25-<30	=35+	100	117	1	2	1	3	3	1	0	1	1	2	0	2	3	0	3.41
25-<30	25-<30	107		1	2	2	2	2	2	0	1	1	1	0	1	2	0	3.53
25-<30	25-<30	107		1	2	1	3	3	2	0	1	1	2	0	1	3	1	4
30-<35	25-<30	100	91	2	2	2	2	2	1	2	2	2	1	1	1	3	1	3.25
=35+	=35+	82	89			1	1	1	1	0	1	1	1	1	1	2	1	3.97
<20y	<20y	122	111	2	1	1	2	1	3	1	1	1	1	1	1	2	0	3.62
<20y	<20y	94			2	1	2	1	2	2	1	1	2	1	1	2	1	3.46
25-<30	25-<30	92	85		1	2	2	2	3	2	1	1	0	0	2	2	1	3.3
30-<35	30-<35	118	108	2	2	1	3	3	3	0	1	1	2		1			3.215
25-<30	30-<35	99	92	2	1	1	2	3	3	0	1	1	2	0	1	2	0	4
=35+	=35+	115	128	2	2	2	3	2		0	1	1	2	0	1	3	1	2.85
<20y	<20y	96	88	2	2	1	2	2	1	2	1	1	1	0	1	2	0	3.07
<20y		101	88	1	1	2	3	2	1	2	1	1	2		1			3.78
<20y	25-<30	103	110	1	1	1	2	3	2	0	1	1	2	0	1	2	0	3.255
<20y	<20y	85	76	2	2	2	2	2	1	2	1	1	1	1	1	2	1	2.35
<20y	<20y	90		2	2	2	2	2		0	1	1	1	0	1	3	1	2.42
<20y	<20y	110	101	1	2	2	2	2	3	0	1	1	2	1	1	2	0	3.46
<20y	25-<30	102	102	2	1	1	2	2	1	2	1	1	2	0	1	2	0	3.76
25-<30	30-<35	88	80	1	1	1	1	2	2	0	1	1	1	0	1			3.9
25-<30	=35+	114	106		2	1	2	1	1	2	2	1	1	1	1	2	0	4.3
30-<35	=35+	115	110	2	1	2	1	3	2	0	1	1	2	1	1	2	0	4.1
<20y	30-<35	102	88	2	2	2	2	3	3	0	1	1	1	0	1	2	0	2.995
25-<30	<20y	99	95	2	1	1	2	2	3	1	2	1	2	1	1	3	0	3.57
20+yr	<20y	83	98		2	1	2	2	3	1	1	1	1	0	1	2	1	3.51
<20y	<20y	78	79	2	1	1	2	2		2	1	1	2	0	1	2	1	3.4
<20y	<20y	102	104	2	1	1	2	2	3	0	1	1	2	1	1	2	1	4.36
25-<30	30-<35	103	96	2	2	2	2	3	1	0	1	1	2	1	1	2	0	3.01

<20y	<20y	101	111	2	2	2	3	3	3	0	1	1	1	0	2	2	0	3.11
25-<30	30-<35	111	97	2	1	2	2	2	3	0	1	1	1	0	1	2	0	3
<20y	<20y	102	96	2	2	1	2	2	1	0	1	1	1	0	2	2	1	3.71
<20y	20+yr	89		1	2	1	2	1	3	0	1	1	0	1	1	2	1	3.004
25-<30	25-<30	86	88	2	2	1	2	2	2	0	1	1	2	0	2	1	0	4.41
25-<30	25-<30	99	89	2	1	2	2	2	2	0	1	1	0		1			3.51
20+yr	<20y	101	86	2	2	2	1	2		0	1	1		1		2		3.37
25-<30	=35+	103	98	2	2	1	2	2	3	0	1	1	2	0	1	2	0	2.835
25-<30	30-<35	105		1	1	1	2	3	3	0	1	1	1	1	1		0	4.47
25-<30		96	86	2	1	2	2	2	2	1	2	1	2		1	2	1	3.59
<20y	<20y	110	87	2	2	1	1	1		2	1	1	0		1			3.49
<20y	<20y	85		1	1	1	1	2	2	1	1	1	1	0	1	2	0	3.4
25-<30	25-<30	115	110	2	2	2	2	3	1	0	1	1	1	0	1	2	1	4.27
<20y	25-<30	107	90	1	1	1	2	2	3	1	1	1	1	0	1	2	0	2.98
30-<35	=35+	109	97	2	1	1	2	1	1	0	1	1	2	0	1	2	1	3.195
25-<30	30-<35	110	95	2	2	1	2	2	2	0	1	1	2	1	1	1	0	3.12
<20y	25-<30	88	80	2	1	2	1	2	3	1	2	1	2	1	1			4.415
25-<30	=35+	96	90	1	2	2	3	2	2	2	1	1	2	1	2	2	0	2.68
<20y	<20y	96	85		1	2	2	2	2	1	1	1	2		1			2.66
25-<30	25-<30	105	96	2	1	1	2	2	3	0	1	1	2	1	1	2	0	3.48
<20y	<20y	98	93	2	2	2	2	1	2	0	1	1	0	0	1	2	1	3.79
25-<30	30-<35	106	92	2	2	2	2	3	3	0	1	1	2	1	1	3	0	4.09
20+yr	=35+	101	96	1	2	2	2	3	3	0	1	1	0	0	1	2	1	2.95
<20y	<20y	96	91	2	1	2	1	1	1	2	1	1	2	0	1	2	1	3.21
30-<35	=35+	125	114	2	1	2	3	3	2	0	2	1	2	1	2	2	0	4.02
<20y	25-<30	113	109	2	1	2	3	1	1	0	1	1	1	0	1		0	3.57
30-<35	=35+	102	98	2	1	1	2	2	3	0	1	1	0	0	1	1	0	3.68
20+yr		90	88	1	2	2	1		1	2	2	1	1	1	2	2	1	3.54

25-<30	30-<35	96	92	2	1	1	2	2	2	0	1	1	2	1	1	2	1	3.77
30-<35	30-<35	117	128	2	2	1	2	2	3	0	2	1	2	0	1	2	1	3.51
20+yr	<20y	103	85	2	2	2	1	2	1	0	1	1	0	1	1	2	0	4.03
30-<35	=35+	104	105	2	1	1	2	2	3	0	1	1	2	0	2	2	1	4.1
<20y	<20y	90	100	1	1	2	2	1	1	0	2	1	1	1	2	3	0	3.15
20+yr	<20y	94	84		1	2	2	2	1	1	2	2	2		2			3.465
=35+	=35+	79		1	2	1	1	2		2	2	1	0	0	1	2	0	2.76
<20y	<20y	111	110	2		1	2	1	3	0	1	1	1	0	1	2	0	4.11
25-<30	30-<35	103	98	2	1	2	1	2	2	0	1	1	2	0	2	2	0	3.29
<20y		109	88	2	1	2	3	1	2	2	1	1	1	0	1	2	0	2.73
25-<30	25-<30	92	86		1	1	2	3	3	0	1	1	0	0	1	1		3.35
<20y	<20y	110	122	2	2	2	3	2	3	0	1	1	2		1	2	1	2.64
<20y	30-<35	107	103	2	2	1	2	2	3	0	1	1	2	0	1	1	1	3.3
<20y	25-<30	94	83	2	1	2	2	2	2	0	1	1	2	0	1	2	0	3.36
25-<30	=35+	97	101	2	2	2	1	1	3	2	1	1	0		1			3.18
<20y	25-<30	122	91	2	2	1	3	3	3	0	1	1	2	0	1	2	1	3.06
30-<35	30-<35	107	93	2	2	2	2	2	3	0	1	1	2	0	1	3	0	4.11
<20y	30-<35	94		1	2	1	2	2		0	2	2	1	0	1	2	1	3.63
25-<30	25-<30	78	85	2	1	2	1	2	3	2	1	1	0	0	1	2	1	3.39
20+yr	25-<30	104	104	1	2	1	1	2	3	2	2	2	0	0	1	3	0	2.93
<20y	=35+	92	97	2	2	2	3	2	3	0	1	1	1		1			3.21
<20y	30-<35	85		2	2	2	2	2	3	2	1	1	1	1	1	2	1	2.91
25-<30	=35+	120	92	2	2	1	3	2	3	0	1	1	2	0	1	2	0	3.275
<20y	25-<30	92	94	2	2	2	2	3		0	1	1	0	0	1	2	1	4.14
<20y	25-<30	88	89	2	1	1	2	2	3	0	2	1	2	0	1	2	0	3.83
25-<30	30-<35	104	101	2	1	1	2	2	2	2	1	1	0	0	1	2	1	3.47
30-<35	=35+	106	90	2		2	2	3		0	2		2	0	1	1	0	2.67
<20y	<20y	91	92	2	2	1	3	3	3	0	2	1	1	1	1	2	1	3.4

25-<30	=35+	114	91	1	2	1	1	3	1	0	1	1	2	0	1	2	0	2.74
25-<30	25-<30	84	98	2	1	1	3	3	3	0	1	1	2	0	1	2	1	3.02
30-<35	30-<35	117	99	1	2	1	3	3	3	0	1	1	1		1			4.075
<20y	25-<30	114	104	2	2	2	2	3	1	2	1	1	1	0	1	2	1	4.03
25-<30	25-<30	129	101	2	1	1	2	2	3	0	1	1	1	0	1	2	0	3.28
<20y	20+yr	102	91	1	2	1	2	2	1	2	2	1	1	0	1	2	1	3.4
<20y	<20y	114	109	2	1	1	2	3	3	0	1	1	1	0	1	2	0	2.95
<20y	=35+	127		2	2	2	1	3		0	1	1	2	1	1	2	1	3.01
<20y	30-<35	97	89	2	1	2	2	2	2	0	1	1	1	1	1	2	0	2.84
25-<30	30-<35	102	80	2	1	2			3	1	1	1	0	0	1		1	3.62
25-<30	30-<35	92	110	1	1	1	2	2	2	2	2	1	1	0	1	2	1	2.64
<20y	25-<30	124	101	2	2	2	3	2	3	0	2	1	2	0	1	2	1	4.15
25-<30	25-<30	91	119	2	2	2	2	3	3	0	1	1	2	0	1	3	0	3.38
20+yr	25-<30	103	101	2	2	2	2	2	2	0	1	1	1	0	1	2	0	3.1
25-<30		95	95	2	1	2	2	2		2	2	1	1	1	1	2	0	3.54
=35+	=35+	97	86	2	2	1	1	1	3	2	2	1	0	0	1			2.3
<20y		96	100	1	2	2	2		1	1	2	1	2		1	2	1	3.99
<20y	25-<30	81	103	2	1	1	2	2	3	1	2	1	0	1	1	2	1	3.8
<20y	25-<30	88		2	1	2	2	3	1	0	1	1	1	0	1	2	1	3.16
20+yr	<20y	89	99		2	1	2	1	1	1	1	1	1	0	1	2	0	3.725
<20y	25-<30	104	104	2	1	2	2	2	3	0	1	1	2	0	2	2	0	3.71
<20y	<20y	118	95	1	2	2	2	3	3	0	1	1	2	0	1	2	0	3.26
20+yr	<20y	103	91	2	2	1	2	2	1	1	1	1	2	0	1	1	1	2.95
30-<35	25-<30	98	102	2	1	1	2	1		0	1	1	2	0	2	2	1	3.18
25-<30	<20y	109	104	2	1	1	2	2	3	0	1	1	1	1	1	2	1	3.22
<20y	25-<30	117	101	2	1	1	2	2	3	0	1	1	2	0	1	2	0	3.965
25-<30	25-<30	111	108	2	1	1	2	2	2	0	1	1	2	1	1	2	1	2.685
25-<30	25-<30	97	80	2	1	1	2	2	2	0	2	1			2			3.05

=35+	=35+	111	89	2	2	1	2	1	2	0	1	1	1	1	1	2	0	2.32
25-<30	30-<35	112	117	1	2	1	3	3	3	2	1	2	1	0	1	2	1	3.61
<20y	<20y	100	115	2	2	1	2	2	3	0	1	1	1	1	2	2	0	2.747
30-<35	30-<35	98	90	2	2	2	2	3	3	0	1	1	2	0	1		0	3.11
<20y	<20y	117	89	1	2	1	3	2	1	0	2	2	2	0	1	2	1	3.2
25-<30	30-<35	108	97	2	1	1	3	2		1	1	1	2	0	1		0	3.39
25-<30	=35+	103	87	2	2	2	1	2	3	0	1	1	2	0	1	2	0	3.23
=35+	=35+	94	90	2	2	1	2	2	2	0	1	1	2		1			3.955
<20y		110	94	2	2	2	1	2	2	2	2	1	1	0	1	1	0	3.575
25-<30	30-<35	102		2	2	2	3	3	3	1	1	1	2	0	1	3	0	3.36
=35+	25-<30	112	89	2		2	1	3	3	1			0	1	1		0	2.86
25-<30	25-<30	103	106	1	1	2	2	2	2	0	2	1	1	1	2	2	0	2.81
<20y	<20y	106	92	1	1	2	2	2	2	0	2	1	0	0	2	1	1	3.64
<20y	30-<35	84		1	2	1	2	2	3	0	1	1	0	0	2	2	1	2.94
25-<30	30-<35	91		2	2	1	2	2		0	2	1	2	0	2	2	0	3.95
<20y	30-<35	108	98	2	2	2	2	3	2	0	1	1	2	0	2	2	1	3.66
30-<35	30-<35	90	104	2	1	2	3	3	2	0	1	1	2	1	1	1	0	2.585
<20y	25-<30	125	125	2	2	1	2	2	3	1	1	1	2	0	1	2	1	4.015
<20y	<20y	108	83	1	2	2	2	2	2	0	1	1	2	0	1	3	0	3.48
<20y	25-<30	115	104	2	2	2	2	2	3	1	1	1	2	0	2	2	0	2.96
30-<35	30-<35	100		1	1	1	2	1	1	1	1	2	1	1	1	2	0	3.54
25-<30	30-<35	79	97		1	1	3	2	1	0	1	1	2	0	1	3	0	3.47
<20y	25-<30	107		2	2	1	2	2	2	2	2	1	2		2			4.04
25-<30	25-<30	120	139	2	1	1	3	3	2	0	1	1	2	0	2	2	1	3.75
25-<30	30-<35	107	98	2	2	1	3	1	2	0	1	1	2	0	1		0	4.035
<20y	25-<30	106	90	1	1	2	3	2	1	0	2	1	1	0	1	2	0	3.51
<20y	30-<35	94	91	1	2	2	2	2	3	0	1	1	1	0	1	2	1	3.72
30-<35		96	85	2	2	1				0	1	1	2	0	1	3	1	4.05

25-<30	25-<30	104	100	1	1	2	2	1	3	1	1	1	0	0	1	2	0	3.35
25-<30	25-<30	134	103	2	2	1	2	2	1	0	1	1	2	0	2	2	0	2.795
30-<35	=35+	111	96	1	2	1	3	3	3	0	1	1	2	0	1	2	0	4.12
25-<30	25-<30	99	100	2	1	1	2	2	3	0	1	1	2	0	2	2	0	3.18
20+yr	<20y	101	81		2	1	2	2	2	0	1	1	1	0	1	2		2.89
<20y	25-<30	114	96	2	1	1	2	2	3	0	1	1	1	0	1	3	0	3.76
<20y	25-<30	105	89	2	1	1	3	3	2	0	1	1	1	0	2	2	1	4.2
30-<35	=35+	108	105	1	1	2	3	3	3	1	1	1	2	0	1		0	4
=35+	=35+	88		2	2	2	2	2	1	0	1	1	1		1			3.46
25-<30	30-<35	117	94	2	1	2	3	2	2	1	1	1	0	1	1	1	0	2.77
25-<30	30-<35	114	108	1	2	2	3	3	3	0	1	1	2	1	1	3	0	3.18
20+yr	<20y	107	102	1	1	2	2	2	2	1	1	1	0		2			2.63
<20y	<20y	97	86	2	1	1	2	2	3	2	1	1	0	0	1	1	0	4.595
<20y	25-<30	93	76	2	1	1	1	1	3	0	1	1	1	1	1	2	1	2.495
25-<30	30-<35	106	97	2	1	2	3	3	3	0	1	1	2	0	1	2	0	3.8
<20y	25-<30	99			1	1	2	2	2	1	1	1	2	0	1	2	0	3.26
25-<30	30-<35	110	117	1	1	2	2	2	3	1	1	1	1	1	1	2	0	3.96
30-<35	30-<35	99	84	2	2	2	1	1	2	0	1	1	1	0	1	2	0	3.35
30-<35	25-<30	118	117	2	2	1	3	3	1	0	1	1	2	0	1	3	1	3.99
<20y	25-<30	106	109	1	2	1	2	2	1	1	2	2	2	0	1	2	0	3.7
20+yr	<20y	107	87	1	2	2	2	1	1	2	1	1	0	0	1	2	1	3.14
20+yr	<20y	90		1	1	2	2	2	1	0	1	1		0		2	0	2.9
<20y	25-<30	119	129	2	1	1	3	1	3	0	1	1	2	0	1	2	0	3.35
20+yr	<20y	82		1	2	1	2	2		0	2	1	1	0	1	2	1	3.3
25-<30	25-<30	106	112	2	2	1	2	3	3	0	1	1	2	0	1	3	0	3.375
20+yr	<20y	86		1	2	2	2	2	1	2	1	1	1	0	1	2	1	3.08
25-<30	25-<30	109		1	2	1	2	3	1	0	1	1	1	0	1	3	0	3.715
<20y	<20y	113	103	2	1	2	3	2	2	0	1	1	2	0	1	3	0	4.585

20+yr	<20y	82	88	1	1	2	2	2	2	1	1	1	1	0	1	2	1	2.57
<20y	25-<30	108	99	2	2	2	1	3	3	0	1	1	2	0	1	2	0	3.155
<20y	25-<30	95	100		2	1			2	2	1	1						3.84
<20y	<20y	96	101	2	1	2	2	2	3	0	1	1	1	0	1	2	0	2.98
<20y	25-<30	122		1	2	1	3	3	3	0	1	1	2		1			3.21
25-<30	25-<30	97		2	2	1	2	2	2	0	1	1	2	0	1	2	1	3.3
30-<35	30-<35	97	81	1	1	2	1	3	2	0	1	1	2	0	1	2	0	3.4
30-<35	<20y	83	98		1	2	2	2		1	1	1	1	0	1	1		2.9
30-<35	=35+	114	109	2	1	2	3	3	2	2	1	1	2	0	1	3	0	3.8
<20y	25-<30	105	90	2	1	2	2	2	2	1	1	1		0		2	0	3.83
<20y	<20y	97		2	2	2	2	2		0	1	1	1	1	2	2	1	4.12
=35+	=35+	112	103	2	2	2	2	2	3	1	1	1	1	0	1	3	0	4.09
25-<30	<20y	111	108	2	2	1	2	1	2	0	1	1	2	0	1	2	0	3.245
<20y	<20y	97	84		1	2	2	3	3	0	1	1	2		1			3.68
25-<30	25-<30	93	101	1	1	2	2	3	3	0	1	1	0	0	1	2	1	3.43
<20y	25-<30	114	109	2	2	1	2	2	2	1	1	1	2		1			3.71
<20y	<20y	103	89	1		2	2	2	2	2	2	1	1	0	1	2	0	3.965
30-<35	30-<35	118	117	2	1	1	2	3	3	0	1	1	2	1	1	2	1	4.87
<20y	25-<30	106	94	2	2	2	3	3	1	0	1	1	2	0	1	3	1	3.655
20+yr	<20y	118		2	2	2	3	3	1	0	1	2	1		1			3.75
<20y	<20y	104	100	1	2	1	2	3	3	0	1	1	0		1	1	0	4.62
30-<35		97	88	2	1	1	2	1	2	0	1	1	2	0	1	2		3.83
25-<30	30-<35	89	98	2	1	1	3	3	3	0	2	1	2	0	1	1	0	3.88
30-<35	30-<35	111	99	1	2	2	1	2	1	2	2	1	0	0	1		1	2.146
20+yr	<20y	107	87	1	2	1	2	2	2	2	1	1		1		3	1	1.928
<20y	30-<35	102				2	2	2	2	1	1	1	0		1			3.28
<20y	25-<30	114	100		2	2	2	3	3	1	1	1	1	0	1	1	1	3.18
20+yr	20+yr	83		1	2	2	1	2	1	0	1	1	1	0	1		0	3.06

<20y	<20y	83	97	1	1	2	3	2	2	2	1	1	0		1			3.4
<20y	25-<30	122	111	2	2	1	3	2	2	0	1	1	2	0	1	2	0	3.02
<20y	<20y	101		1	2	2	2	2	2	2	2	1	0	0	2	2	1	4
<20y	25-<30	105	114	2	1	2	2	3	1	0	1	1	2	0	1	2	0	3.26
<20y	<20y	101	114	2	1	2	2	3	2	0	2	1	2	1	1	2	0	1.165
<20y		93		1	2	1						1	0	0	1	2	0	4
30-<35	=35+	109	109	2	2	1	2	2		2	2	1	1	0	1	2	1	3.57
<20y	25-<30	115	108	2	2	1	2	2	2	0	1	1	2	0	2	2	0	4.245
<20y	=35+	99	85	2	1	2	2	2	2	0	1	1	0	1	1	2	1	3.405
30-<35	=35+	125	103	2		1	2	1	2	0	1	1	2	0	1	2	1	3.68
30-<35	=35+	104		1	2	1	2	1	3	0	1	2	0	0	1	2		3.41
30-<35	=35+	117	75	2	1	1	1	1	2	0	1	1	0	0	1	3	1	3.38
25-<30	25-<30	96	96	2	1	2	3	3	3	0	1	1	2	0	1	3	0	3.4
<20y	<20y	94	95	1	1	2	2	2		0	1	1	0	0	1	2	0	2.83
25-<30	30-<35	118	110	1	2	2	3	3	2	0	1	1	2	0	1	3	1	3.11
=35+	=35+	99	95	2	2	1	2	2	2	2	1	1	1	0	2	2	1	2.77
<20y	25-<30	113	96	2	1	2	2	2	3	1	1	1	2	0	1	2	0	3.98
20+yr	<20y	117	97	2	2	1	2	2	1	1	1	1	1	0	1	2	1	2.89
25-<30	25-<30	103	98	2	1	2	2	2	2	2	2	1	1	0	1	2	0	2.62
25-<30	30-<35	87	92	2	1	2	2	2	3	0	1	1	1	0	1	2	0	4.32
<20y	25-<30	94	105	1	1	1	2	1	3	0	1	1	0	0	1	2	0	3.66
<20y	<20y	93	105	2	1	1	1	2	2	0	1	1	1	1	1	2	0	3.84
<20y	=35+	117		2	2	2	2	3	1	0	1	1	2	0	2	3	1	3.36
=35+	=35+	104	109	2	1	2	3	3	3	0	1	1	1	0	2	3	0	3.85
30-<35	30-<35	111	95	2	1	1	2	1	3	0	1	1	0	0	1	2	0	3.39
20+yr	<20y	86	105		2	2	2	2	1	2	1	1	0	0	1	3	1	3.51
20+yr	<20y	106		1	2	2	2	2	2	0	2	1	1	0	1	2	1	3.74
<20y	<20y	94	82	1	1	1	2	2	2	2	2	1	0	0	1	1		3.47

25-<30	25-<30	115	97	2	2	1	3	3	2	0	1	1	2	0	1	2	1	2.53
<20y	<20y	108		1	1	1	1	2	1	0	1	1	1	0	1	2	0	3.1
25-<30	30-<35	106	90	2	2	2	2	2	3	0	1	1	0	0	1	2	0	3.86
<20y		98		1	2	2	2		1	2	2	1	0	0	1			3.52
25-<30	<20y	104	93	2	1	2	3	2	1	0	1	1	2		1			3.74
<20y	<20y	104		1	2	1	2	2	1	1	1	1	0	0	1		0	4.33
30-<35	=35+	110	117	2	1	2	3	3	3	0	1	1	2	0	1	2	0	3.19
<20y	<20y	108		2	2	1	1	1	2	2	2	1	0	0	1	2	1	3.045
25-<30	25-<30	94	85	1	2	1	1	1	2	2	2	1	1	0	1	2	1	2.38
25-<30	25-<30	132	114	2	2	1	3	3	3	0	1	1	2	0	1	3	1	3.38
<20y	<20y	87	87	2	2	1	1	2		0	1	1	0	0	2	1	0	3.94
=35+	=35+	107	82	2	2	2	3	1	3	0	1	1	0	0	1	2	0	3.56
